# Skeletal editing of pyridines through atom-pair swap from CN to CC

**DOI:** 10.1038/s41557-023-01428-2

**Published:** 2024-01-18

**Authors:** Qiang Cheng, Debkanta Bhattacharya, Malte Haring, Hui Cao, Christian Mück-Lichtenfeld, Armido Studer

**Affiliations:** 1 Organisch-Chemisches Institut, Universität Münster, Münster, Germany; 2 College of Chemistry and Molecular Sciences, Wuhan University, Wuhan, P. R. China

**Keywords:** Diversity-oriented synthesis, Synthetic chemistry methodology

## Abstract

Skeletal editing is a straightforward synthetic strategy for precise substitution or rearrangement of atoms in core ring structures of complex molecules; it enables quick diversification of compounds that is not possible by applying peripheral editing strategies. Previously reported skeletal editing of common arenes mainly relies on carbene- or nitrene-type insertion reactions or rearrangements. Although powerful, efficient and applicable to late-stage heteroarene core structure modification, these strategies cannot be used for skeletal editing of pyridines. Here we report the direct skeletal editing of pyridines through atom-pair swap from CN to CC to generate benzenes and naphthalenes in a modular fashion. Specifically, we use sequential dearomatization, cycloaddition and rearomatizing retrocycloaddition reactions in a one-pot sequence to transform the parent pyridines into benzenes and naphthalenes bearing diversified substituents at specific sites, as defined by the cycloaddition reaction components. Applications to late-stage skeletal diversification of pyridine cores in several drugs are demonstrated.

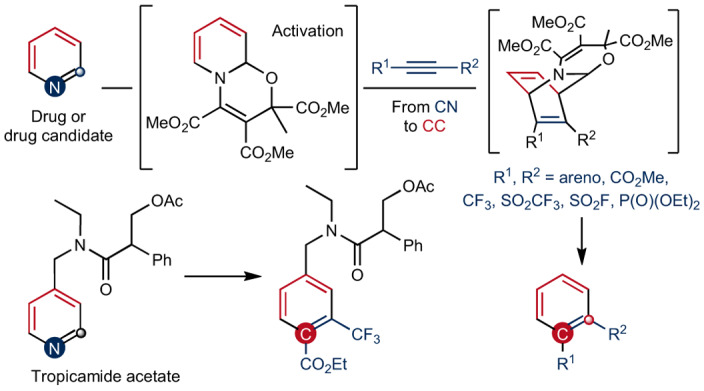

## Abstract (web summary)

Skeletal editing enables diversification of compounds not possible by applying peripheral editing strategies. Now, a catalyst-free atom-pair swap strategy for pyridine editing has been developed via one-pot sequential dearomatization, cycloaddition and rearomative retrocyclization. Benzenes and naphthalenes with precisely installed functional groups are produced, and the mild conditions enable late-stage skeletal diversification of pyridine cores.

## Main

The pyridine ring is a ubiquitous structural core in pharmaceuticals and materials^1–3^. Its reactivity and, in turn, its selective chemical modification mainly rely on the intrinsic electron-deficiency of the *π*-system and the *σ*-donating ability of the nitrogen atom^4^. Peripheral editing of pyridines has enabled regioselective functionalization at specific C–H bonds under mild conditions, offering powerful tools for modification of the pyridine moiety in drugs and materials^5–13^. An equally important approach for pyridine functionalization is the precise skeletal editing, where one or more atoms of the six-membered nitrogen-containing aromatic ring system is substituted to produce other (hetero)arenes^14,15^. Such a scaffold-hopping strategy is of high interest to the field of medicinal chemistry^16^. However, compared with the rather well-established peripheral C–H functionalization, direct skeletal editing of pyridines is even more challenging, with transformations proceeding through generally higher energy barriers caused by the necessary dearomatization step. Moreover, the cleavage of strong C–N or C–C bond is required and general synthetic methods for pyridine skeletal editing are currently not available.

**Fig. 1:**
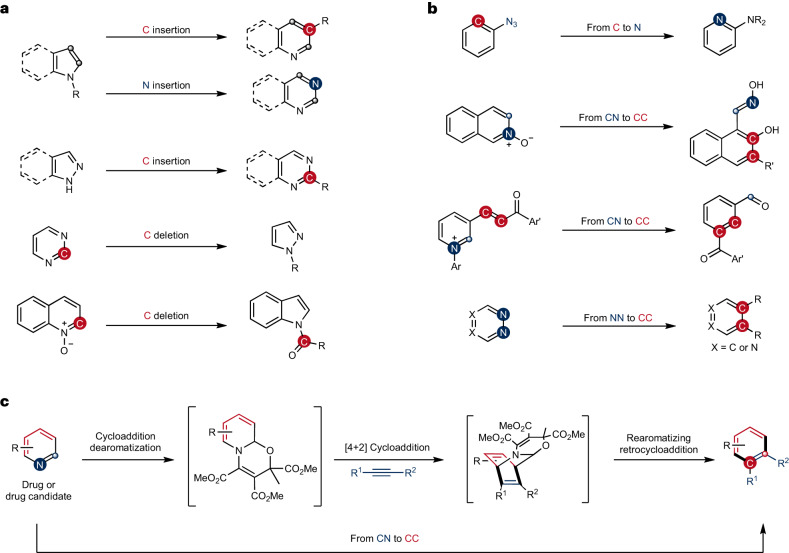
Various strategies for skeletal editing of arenes and heteroarenes. **a**, Heteroarene skeletal editing through single-atom insertion and deletion that can be used at a late-stage, yet not reported for pyridines. **b**, Atom swap in arenes is challenging, with existing methods showing limited substrate scope and therefore application to late-stage modification is not possible. **c**, Our developed strategy through sequential dearomatization, cycloaddition and rearomatizing retrocyclization enabling an atom-pair swap from CN to CC in pyridines. The method is a one-pot modular approach for pyridine editing with a broad substrate scope that is applicable to late-stage modification.

Due to the extra energy compensation of aromaticity, reactive reagents are required for precise skeletal editing of arenes (Fig. 1). Along these lines, carbenes have been investigated for a long time, as early realized in the Buchner reaction^17^ and the Ciamician–Dennstedt rearrangement^18^. Despite their potential, surprisingly, these reactions have not been broadly applied to skeletal diversification of (hetero)arenes until recently. For example, single carbon atom insertion into pyrroles or pyrazoles to produce substituted pyridines or pyrimidines following a reaction with diazirines as carbene precursors has been achieved^19,20^. A similar single atom editing logic has also been realized through nitrene insertion to indoles to form quinazolines or quinoxalines^21^. Conversion of pyrimidines into pyrazoles has been achieved with a formal carbon atom deletion through triflation of the pyrimidine core followed by a hydrazine-mediated rearrangement^22^. Following photoirradiation, quinoline *N*-oxides have been shown to undergo one carbon deletion to afford *N*-acyl indoles^23^. Analysis of the existing strategies reveals that most of the recently developed skeletal-editing reactions of aromatic compounds proceed through single-atom insertion or deletion, producing products with a different ring size (Fig. 1a). Chemical modification of arenes through an atom swap to generate the corresponding skeletal-edited congeners with the same ring size is less well explored, probably because the substrate scope is limited for most reported methods, rendering it difficult to apply these reactions to late-stage diversification of more complex systems (Fig. 1b)^24–38^. As an example, an intriguing rearrangement from aryl azides to aminopyridines has been developed^25^, whereas application to late-stage arene modification is unattainable, as preinstallation of an azide group in the aromatic substrate is required. Gold-catalysed benzannulation of isoquinoline *N*-oxide generates naphthols through sequential [4+2] cycloaddition and fragmentation^26^. Similar reactivity applies also to pyridinium salts^27^, yet both of these reactions show limited substrate scope. Another example applies Zincke salts to benzannulation through streptocyanine intermediates^28–30^, which though transforms pyridine to benzene in two to three steps, requires specific substitution patterns on pyridines rendering late-stage application tedious. Other strategies include sequential Diels–Alder and retro-Diels–Alder reactions with heteroarenes such as pyridazines, triazines, tetrazines and thiophenes, or with 2-pyrones and cyclopentadienones as reactive dienes, whereas specialized substrates prevent them from application to late-stage chemical core structure modification^31–38^. So far, none of the reported methods allow direct skeletal editing of pyridines with broad scope, and more generally the late-stage skeletal modification of pyridines through atom swap has not yet been developed.

Here we introduce a method for precise skeletal editing of pyridines to generate substituted benzenes and naphthalenes in a modular way (Fig. 1c). We use sequential cyclization dearomatization of pyridines, followed by [4+2] cycloaddition with dienophiles, and rearomatizing retrocycloaddition, to transmute a C=N pair of the pyridine core into a C=C pair under mild conditions. Synthetically useful functional groups such as carboxylate, phosphonate, sulfonyl fluoride and trifluoromethyl groups can be introduced at defined positions with excellent regioselectivity to the arene moiety through this skeletal-editing approach. Arenes with such substitution patterns are difficult to access via other methods. We also show that our method can be readily applied in late-stage to transform pyridine cores into the corresponding substituted benzenes and naphthalenes. During revision of our manuscript, a paper describing C=C pair exchange of arenes with dienophiles via a dearomatization–cycloaddition–retrocycloaddition process was published^39^.

## Results and discussion

Our editing sequence relies on the reactivity of oxazino pyridines functioning as electron-rich dienes in cycloaddition reactions (Fig. 1c). Importantly, such oxazino pyridines are stable compounds and readily obtained in large scale from the parent pyridines. Their nucleophilic reactivity was previously exploited by us for *meta*-C–H functionalization of pyridines^8^, yet their reaction profiles towards dienophiles are underexplored. Here, the dienamine intermediates are used as enophiles in [4+2] cycloaddition reactions with electrophilic alkynes or arynes as dienophiles. The bridged cycloadducts will undergo rearomatizing retrocycloaddition to form stable benzenes or naphthalenes. Including the initial dearomatization of pyridines, the whole process can be performed in one pot, representing a direct transformation of pyridines into benzenes or naphthalenes through CN to CC atom–pair swap.

The dearomative cyclization of pyridines with acetylenedicarboxylates and pyruvates to give oxazino pyridines is highly efficient in various solvents^8^, which allows running the overall editing as a one-pot sequence, as the cycloadditions with different dienophiles require different solvents. When heating such oxazino pyridine intermediates with in situ-generated arynes (from *ortho*-trimethylsilylaryl triflates^40^) in acetonitrile, and with alkynes in 1,4-dioxane or toluene at 80 °C, [4+2] cycloaddition and rearomatizing retrocyclization occur sequentially as one-pot processes to provide substituted naphthalenes and benzenes without any detectable cycloaddition intermediates. A lower or higher reaction temperature will decrease the yield. For the reaction with arynes, acetonitrile is essential as solvent due to poor solubility of caesium fluoride in less polar solvents (see Supplementary Tables 1–5 for detailed reaction optimization). The whole process is catalyst free, robust and not sensitive to air as well as moisture (Supplementary Fig. 1).

We evaluated the one-pot reaction for skeletal editing of different substituted pyridines with benzyne as dienophile (Table 1). Both electron-withdrawing esters (**2**), nitriles (**3**) and ketones (**13**) and electron-donating phenoxy, methyl groups (**4,** **7**) are well tolerated, affording the corresponding naphthalenes in moderate to good yields. The iodo substituent remains untouched in the cascade (**5**), affording building blocks for further coupling transformations. Other potential dienophiles such as alkenes (**6**) and alkynes (**11,** **21,** **23**) are also tolerated in the dearomatization, cycloaddition and retrocyclization sequence. Importantly, the pyridine editing occurs chemoselectively with substrates bearing different heteroarenes as substituents, including pyrazoles (**9**), benzofurans (**10**), pyridines (**14**), indoles (**15**) and thiophenes (**20**), albeit pyridines, pyrazoles and benzofurans have been reported to engage in addition reactions with arynes under similar conditions^40^. In the case of 2,4′-bipyridine, monodearomatization occurs exclusively at 4-substituted pyridine moiety for steric reasons, and **14** was obtained as the only product in 45% yield. For bipyridines showing the same chemical environment, both pyridine rings are transformed into naphthalenes (**12**). Both *para*- and *ortho*-substituted pyridines can be edited under standard conditions to produce the corresponding naphthalenes. As *ortho*-substituents on pyridines increase the steric hindrance both in the initial dearomative cyclization and also in the subsequent [4+2] cycloaddition, lower yields are achieved in these cases (**19**–**23**). For such more challenging substrates, it is recommended to isolate the oxazino pyridine intermediates prior to cycloaddition, and the naphthalenes can be obtained in improved overall yields. For *meta*-substituted pyridines, the regioselectivity of the initial dearomatization step transfers to the final products, producing naphthalenes with two constitutional isomers (**16**–**18**). Although the first two steps of the sequence are sensitive towards steric hindrance, disubstituted pyridines are still eligible substrates (**8,** **22,** **23**).

**Table 1:**
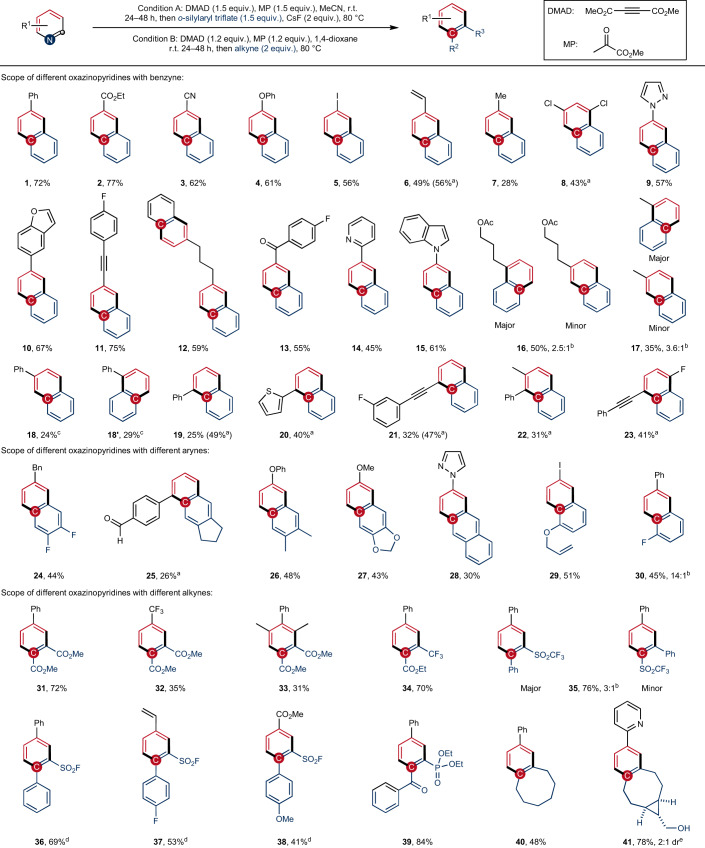
Scope of pyridine skeletal editing

Compounds **1**–**30** are obtained under condition A, in which *ortho*-trimethylsilylaryl triflates are used as aryne precursors. Under condition B, multisubstituted benzenes **31**–**41** are generated from reactions with activated alkynes. All yields are isolated yields based on pyridines in a one-pot process, unless stated otherwise. ^a^Yield based on the isolated oxazino pyridine intermediates. ^b^Combined yield of two constitutional isomers. ^c^Two isolable constitutional isomers, with a ratio of 1.2:1. ^d^Applying condition B, using toluene as a solvent instead of 1,4-dioxane. ^e^The cyclooctyne was used as a 2:1 diastereoisomeric mixture (see page 77 of the Supplementary Information). DMAD, dimethyl acetylenedicarboxylate; MP, methyl pyruvate.

Pyridine editing reactions with symmetrically disubstituted arynes bearing fluoro, alkyl and O-substituents provide multisubstituted naphthalenes (**24**–**27**). The aromatic *π*-system can be further extended to anthracene (**28**) with naphthalene-derived arynes as dienophiles. By using unsymmetrical arynes with 3-alkoxyl (**29**) or 3-fluoro (**30**) substituents, the reaction occurs highly regioselectively, with the nucleophilic δ-carbon of the dienamine intermediates attacking the aryne carbon that is more distorted toward linearity^41^. Activated alkynes (**31**–**41**) are also suitable dienophiles, contributing to the modular CN to CC atom-pair swap strategy that transmutes pyridines into multisubstituted benzenes; however, no reaction occurs with unactivated alkynes or terminal alkynes (see Supplementary Fig. 3).

Electronically activated alkynes such as dimethyl acetylenedicarboxylates^42,43^ also undergo cycloaddition with the in situ-generated oxazino pyridine intermediates to afford benzenes bearing dicarboxylate substituents (**31**–**33**). Penta-substituted benzenes with varied substituents are rarely accessible from step-wise synthetic methods, yet they are important structures for expanding the chemical space of benzenoid pharmaceutical compounds^44^. In this work we obtain a penta-substituted benzene **33** (attached with alkyl, aryl and carboxyl groups) directly from easily accessible trisubstituted pyridines. Moreover, an unsymmetrical alkyne bearing an ester and a trifluoromethyl group reacts regioselectively under optimized conditions to give the trisubstituted benzene **34** in 70% yield. Excellent regioselectivity can also be obtained for acetylenes that are activated by phenyl and fluorosulfonyl groups (**36**–**38**), as well as by acyl and phosphoryl groups (**39**), generating multisubstituted benzenes with defined introduction of diverse functional groups. This skeletal modification method provides a general platform to install pharmacologically valuable functional groups into aromatic compounds at specific positions. The trifluoromethylsulfonyl-substituted phenylacetylene also engages in the editing sequence, though with a moderate selectivity (**35**). Along with electronic activation of the triple bond, strain-induced activation of the alkyne entity can also accelerate the cycloaddition step with the dienamine intermediates—as shown in the transformation with cyclooctynes^45^—to provide substituted benzocyclooctenes in moderate to good yields (**40,** **41**). Thus, our atom-pair swap strategy provides a direct synthetic method for construction of multisubstituted benzenes with medical relevant functionalities from readily available pyridines.

**Fig. 2:**
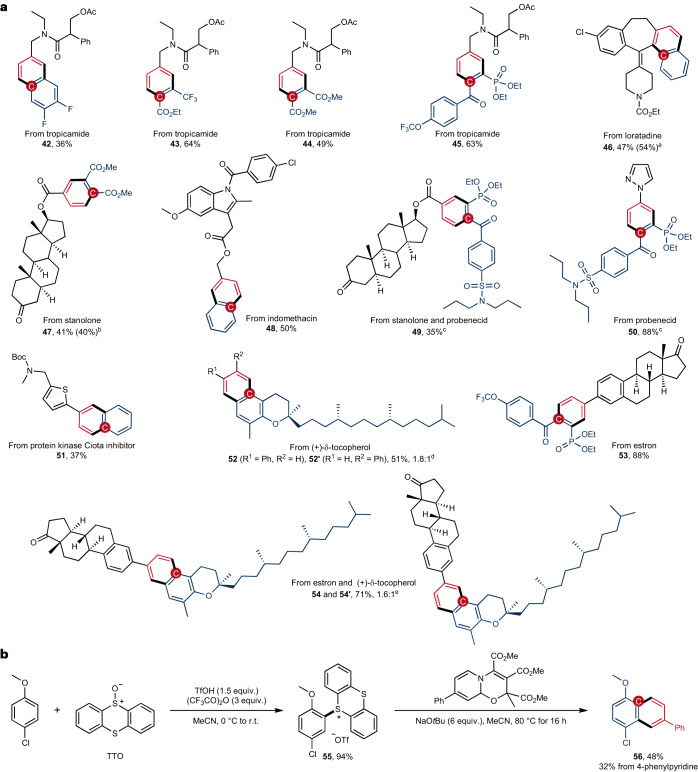
Application of the skeletal editing strategy. **a**, Skeletal editing of pyridine cores in drugs and drug derivatives. Compounds **42,** **46,** **48,** **51,** **52,** **54** are obtained under condition A, whereas others are synthesized under condition B. All yields are isolated yields based on pyridine in a one-pot process unless stated otherwise. ^a^The gram-scale yield (in parenthesis) by using condition A. ^b^The gram-scale yield (in parenthesis) by using condition B under air. ^c^Applying condition B with probenecid-derived alkyne (1.2 equiv.); the yield is based on the pyridine. ^d^With condition A using (+)-δ-tocopherol derived aryne precursor (1 equiv.), 4-phenylpyridine (1.5 equiv.), DMAD (1.5 equiv.), MP (1.5 equiv.) and caesium fluoride (1.5 equiv.); the yield is based on the aryne precursor. ^e^Combined yield of the two constitutional isomers. **b**, Application of aryne formation from aryl thianthrenium salt in pyridine editing.

To illustrate the potential of the developed pyridine editing strategy, we applied the reaction to late-stage modification of drugs and drug derivatives (Fig. 2a). As exemplified with acyl-protected tropicamide (**42**–**45**), the pyridine scaffold is precisely edited in a one-pot process by using different dienophiles to generate diverse benzene and naphthalene derivatives. The internal double bond in loratadine (**46**) and the thiophene ring in a protein kinase inhibitor^46^ (**51**) are well tolerated. By simple derivatization of pyridine–stanolone (**47,** **49**), pyridine–indomethacin (**48**) and pyridine–estron (**53**) conjugates, we can use the atom swap strategy to introduce medicinally and agrochemically relevant functionalities such as carboxylate, phosphonate and trifluoromethoxy groups. On the other hand, owing to the modularity of the method, drugs can also be modified in the other direction by transforming them into dienophiles. Along this line, the probenecid derived alkynes can function as cycloaddition partner in pyridine-editing reactions, producing a phosphonate and pyrazole-incorporated benzene derivative of probenecid in 88% yield (**50**). Likewise, with a (+)-δ-tocopherol derived aryne precursor, *π*-extension to the corresponding substituted naphthalene derivatives is achieved by the reaction with substituted pyridines, though with two constitutional isomers being formed (**52,** **54**). Most of the presented products are difficult to construct from de novo synthesis.

The rational design of hybrid molecules containing two different drugs is a quick and cheap method in drug discovery^47,48^. Here we demonstrate the practicality of the modular pyridine-editing method with fragment coupling of two different drug derivatives (**49,** **54**). The well-known transition-metal-catalysed coupling reactions often require prefunctionalized precursors for C–C bond formation, yet in our cases, the corresponding precursors of one or both of the coupling partners are difficult to access via traditional methods. We therefore provide an alternative strategy for fragment coupling through the introduction of a pyridine moiety as an unconventional connecting unit. Moreover, to illustrate the robustness and potential application of the method in both medicinal and process chemistry, we realized the one-pot pyridine editing reaction in gram scale with two different drugs and drug derivatives (**46,** **47**). Both reactions proceed smoothly without compromising the product yield upon upscaling, even though the reaction with stanolone derivative was performed under air (**47**).

We also applied a recently introduced method for the generation of arynes through formal dehydrogenation of arenes^49^ (Fig. 2b). By using aryl thianthrenium salts as aryne precursors, readily accessible substituted arenes can be used as starting material, which further expands the scope with respect to the aryne component. Thus, reaction of 4-methoxy-chlorobenzene with the thianthrene *S*-oxide in the presence of triflic acid and trifluoroacetic anhydride gave thianthrenium salt **55**. This readily isolable salt was reacted with 4-phenylpyridine-derived oxazino pyridine in acetonitrile using NaO*t*Bu as the base at 80 °C for 16 h, and the targeted naphthalene **56** was obtained in 48% yield as a single constitutional isomer. Moreover, the one-pot reaction starting from 4-phenylpyridine and **55** also provided the product **56**, though in a lower yield (32%).

**Fig. 3:**
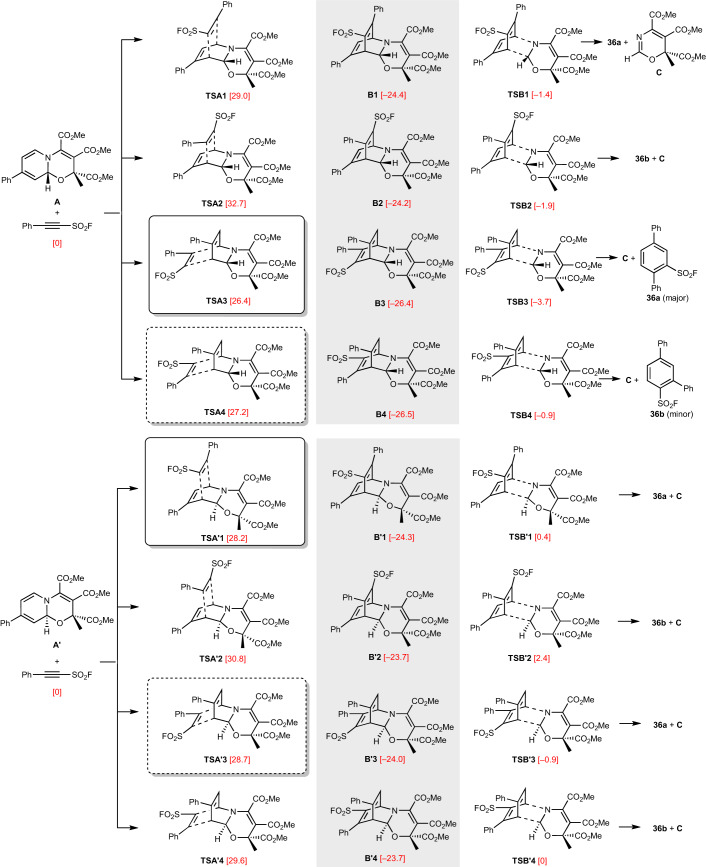
Reaction of the 4-phenylpyridine-derived oxazino pyridine with 2-phenylethinylsulfonylfluoride to give 36. Calculated transition states for the cycloaddition and retrocycloaddition, as well as the structures of the intermediate cycloadducts. The numbers in brackets represent the free energies Δ*G*_s_(353 K), including solvation (PW6B95-D3//TPSS-D3+COSMO-RS/1,4-dioxane), in kilocalories per mole. The calculation is in agreement with the experimental results, which indicates the origin of the regioselectivity and the thermaldynamically favoured rearomatization process.

Finally, we studied the [4+2] cycloaddition and the subsequent retrocycloaddition using density functional theory (PW6B95-D3/TPSS-D3; Fig. 3). The reaction of the 4-phenylpyridine-derived enophile with 2-phenylethinylsulfonylfluoride, which gives **36** with high regioselectivity, was selected. Details on the calculations can be found in page 50 of the Supplementary Information. The two diastereoisomers (**A** and **A′**) of the starting oxazino pyridine differ by only 0.6 kcal mol^–1^, showing why both isomers are formed in the initial dearomatization step (ratio 4:1). In the [4+2] cycloaddition with an unsymmetrical alkyne, eight different transition states are possible and all of them were calculated for the selected transformation. For enophile **A** (less stable isomer), *syn*-addition with respect to the acetal-O-atom of the oxazino pyridine is favoured and all four potential cycloadditions are strongly exothermic (24.2 to 26.5 kcal mol^–1^). Barriers for the two lowest regioisomeric transition states are 26.4 (**TSA3**) and 27.2 kcal mol^–1^ (**TSA4**), respectively. In agreement with the experimental finding that the cycloadducts **B3** and **B4** directly further react under the applied conditions, activation barriers for the retrocycloaddition are lower (22.7 kcal mol^–1^ for **TSB3** and 25.6 kcal mol^–1^ for **TSB4**). For the major oxazino pyridine diastereoisomer, **A′**, the two lowest transition states for the initial cycloaddition (28.2 kcal mol^–1^ for **TSA′1** and 28.7 kcal mol^–1^ for **TSA′3**) lead—following rearomatization—to the experimentally observed regioisomer product. The barrier for **TSA′4** that will eventually afford the regioisomeric product is 29.6 kcal mol^–1^. Again, the activation energies for the retrocycloaddition are lower in energy in all cases (see **TSB′1,** **TSB′3** and **TSB′4**). These calculations reflect the experimentally observed results, albeit at slightly lower regioselectivity than predicted by theory.

A closer inspection of the transition structures of the first cycloaddition reveals an asynchronous C–C bond formation with the bond adjacent to the saturated carbon atom of the dihydropyridine being formed earlier. This simultaneously requires a larger deformation of the alkyne at this reaction centre. Dissecting the energetic barriers into reactant deformation and interaction contributions, we note that the bending of the alkyne fluorosulfonyl bond (**TSA1/3**, **TSA′1/3**) is preferred over the deformation of the phenylethinyl group (**TSA2/4**, **TSA′2/4**). The latter is more destabilizing and leads to larger barriers in the formation of 1,3-diphenyl regioisomers. The only exception is **TSA2**, in which a notably larger interaction energy between alkyne and diene lowers the barrier of this pathway enough to make it a competitive with **TSA3**. Details of the analysis are presented in Supplementary Tables 7 and 8.

## Conclusion

We have developed a general atom-pair swap strategy for pyridine editing through a one-pot sequential dearomatization, cycloaddition and rearomative retrocyclization process. These reactions are robust, occur in the absence of any catalyst and show broad substrate scope for both the pyridine and dienophile components. Substituted benzenes and naphthalenes are formed in a modular fashion with different functional groups precisely installed. The practicality of the method has been illustrated by successful late-stage application in both drug modification and fragment coupling of complex molecules.

## Methods

### General procedure for pyridine editing with arynes as dienophiles (GP1)

A 10 ml oven-dried Schlenk tube equipped with a magnetic stirring bar was subjected to three cycles of vacuum/argon backfill, and charged with pyridine substrates (0.200 mmol, 1.00 equiv.), methyl pyruvate (30.6 mg, 0.300 mmol, 1.50 equiv.) and acetonitrile (1 ml, 0.2 M). Dimethyl acetylenedicarboxylate (42.6 mg, 0.300 mmol, 1.50 equiv.) was then added to the stirred reaction mixture. The reaction mixture was allowed to stir at room temperature for 24 to 48 h. After the reaction was complete, as monitored by TLC, aryne precursors (0.300 mmol, 1.50 equiv.) and CsF (60.5 mg, 0.400 mmol, 2.00 equiv.) were added to the reaction tube. The reaction mixture was stirred at 80 °C for 24 h under argon atmosphere. After the reaction was complete, as monitored by TLC, the solvent was removed on a rotary evaporator under reduced pressure and the residue was subjected to flash column chromatography over silica gel to give the corresponding product.

### General procedure for pyridine editing with activated alkynes as dienophiles (GP2)

A 10 ml oven-dried Schlenk tube equipped with a magnetic stirring bar was subjected to three cycles of vacuum/argon backfill, and charged with pyridine substrates (0.200 mmol, 1.00 equiv.), methyl pyruvate (30.6 mg, 0.300 mmol, 1.50 equiv.), and dioxane or toluene (1 ml, 0.2 M). Dimethyl acetylenedicarboxylate (42.6 mg, 0.300 mmol, 1.50 equiv.) was then added to the stirred reaction mixture. The reaction mixture was allowed to stir at room temperature for 24–48 h. After the reaction was complete, as monitored by TLC, activated alkynes (0.400 mmol, 2.00 equiv.) were added to the reaction tube. The reaction mixture was stirred at 80 °C for 24–48 h under argon atmosphere. After the reaction was complete, as monitored by TLC, the solvent was removed on a rotary evaporator under reduced pressure and the residue was subjected to flash column chromatography over silica gel to give the corresponding product.

### General procedure for pyridine editing through two-pot process (GP3)

The corresponding pyridines (2 mmol, 1 equiv.), methyl pyruvate (0.4 g, 4 mmol, 2 equiv.) and acetonitrile (4 ml, 0.5 M) were added to a 25 ml round-bottom flask with a magnetic stirring bar under air atmosphere. Dimethyl acetylenedicarboxylate (568 mg, 4 mmol, 2 equiv.) was then added dropwise to the stirred reaction mixture. The reaction mixture was allowed to stir at room temperature for 2–48 h. After the reaction was complete, as monitored by TLC, the solvent was removed with a rotary evaporator under reduced pressure and the residue was subjected to flash column chromatography over silica gel to give the corresponding oxazino pyridine intermediates.

A 10 ml oven-dried Schlenk tube equipped with a magnetic stirring bar was subjected to three cycles of vacuum/argon backfill, and charged with oxazino pyridine intermediates (0.200 mmol, 1.00 equiv.), aryne precursors (0.300 mmol, 1.50 equiv.), acetonitrile (1 ml, 0.2 M) and CsF (60.5 mg, 0.400 mmol, 2.00 equiv.). The reaction mixture was stirred at 80 °C for 24–48 h under argon atmosphere. After the reaction was complete, as monitored by TLC, the solvent was removed on a rotary evaporator under reduced pressure and the residue was subjected to flash column chromatography over silica gel to give the corresponding product.

## Online content

Any methods, additional references, Nature Portfolio reporting summaries, source data, extended data, supplementary information, acknowledgements, peer review information; details of author contributions and competing interests; and statements of data and code availability are available at 10.1038/s41557-023-01428-2.

### Supplementary information

Supplementary Information Experimental procedures, product characterization, mechanistic studies, Supplementary Figs. 1–6 and Tables 1–8.

Supplementary Data 1 DFT-optimized structures (Cartesian coordinates).

## Data Availability

All experimental and spectroscopic data are included in the Supplementary Information.
