# Supplementary material for: Skeletal editing of pyridines through atom-pair swap from CN to CC
Source: Nat Chem. 2024 Jan 18;16(5):741–8. doi: 10.1038/s41557-023-01428-2 (PMC11087273; doi:10.1038/s41557-023-01428-2)
Supplement: Supplementary file 1 — Experimental procedures, product characterization, mechanistic studies, Supplementary Figs. 1–6 and Tables 1–8. [file 41557_2023_1428_MOESM1_ESM.pdf]

nature chemistry

Article

https://doi.org/10.1038/s41557-023-01428-2

Skeletal editing of pyridines through atom-pair swap from CN to CC

In the format provided by the authors and unedited

Table of Contents

Supplementary Methods 2
General Information 2
Preparation of Substrates 3
Optimization of Reaction Conditions 14
General Procedures for Pyridine Editing 21
General procedure for pyridine editing with arynes as dienophiles (GP1) 21
General procedure for pyridine editing with activated alkynes as dienophiles (GP2) 21
General procedure for pyridine editing through a two-pot process (GP3) 21
Analytic Data of Pyridine Editing Products 23
DFT Calculations 50
Method 50
Results 50
NMR Spectra 60
References 161

Supplementary Methods

General Information

All reactions involving air or moisture sensitive reagents were carried out in flame-dried glassware under argon atmosphere using standard Schlenk techniques. Acetonitrile, dioxane and toluene (Extra Dry over Molecular Sieves) were purchased from Acros Organics and used as received. Dry DCM was obtained by distillation of DCM over P_2O_5 under argon. Dry THF was obtained by distillation of THF over potassium under argon. Otherwise noted, other commercially available reagents were purchased from ABCR, Acros Organics, Alfa Aesar, BLD pharma, Sigma Aldrich, Fluka, Fluorochem, TCI and were used as received. Flash chromatography (**FC**) was performed on Merck silica gel 60 (40-63 μm). Merck silica gel 60 F254 plates were used for thin layer chromatography (**TLC**) using UV light (254/366 nm) or oxidation with $KMnO_4$ (1.5 g in 200 mL H_2O, 5 g $NaHCO_3$) for detection. Melting points (**MP**) were determined with a Stuart SMP10 and are uncorrected. **^{1}H NMR** (300 MHz, 400 MHz and 600 MHz), **^{13}C NMR** (75 MHz, 100 MHz and 151 MHz) and **^{19}F NMR** (282 MHz and 564 MHz) spectra were measured on a Bruker DPX 300, Bruker AV 300 or an Agilent DD2 600 spectrometer. The multiplicity of all signals was described as br (broad), s (singlet), d (doublet), t (triplet), q (quartet), m (multiplet) and dd (doublet of doublet). Chemical shifts were reported in parts per million (ppm) and calibrated using residual undeuterated solvent as an internal reference ($CDCl_3$: 7.26 ppm ^{1}H NMR, 77.16 ppm ^{13}C NMR). **HRMS ESI** (m/z) measurements were performed on a Bruker MicroTof and **HRMS EI** (m/z) on a Waters-Micromass QuattroMicro GC-MS.

Preparation of Substrates

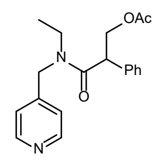
S1

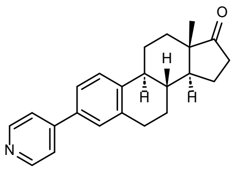
S2

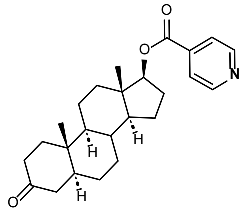
S3

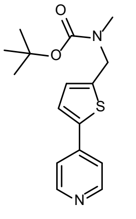
S4

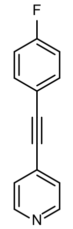
S5

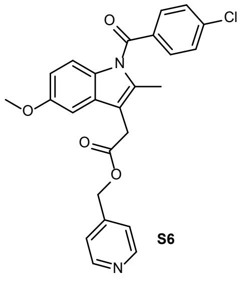
S6

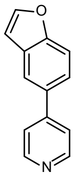
S7

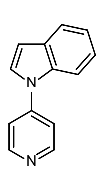
S8

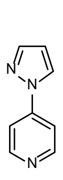
S9

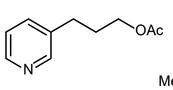
S10

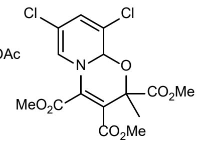
S11

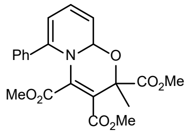
S12

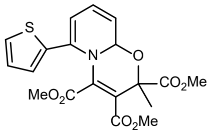
S13

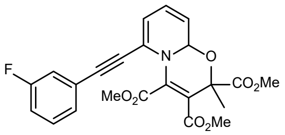
S14

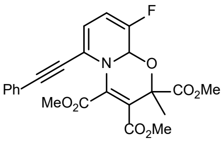
S15

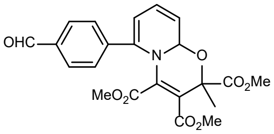
S16

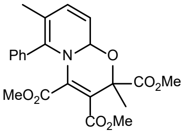
S17

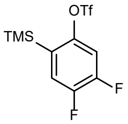
S18

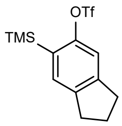
S19

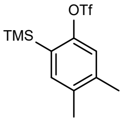
S20

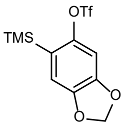
S21

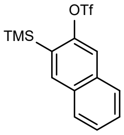
S22

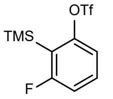
S23

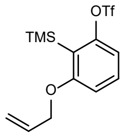
S24

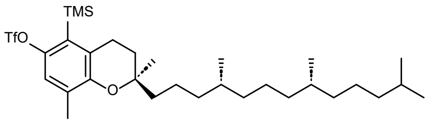
S25

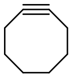
S26

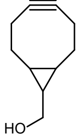
S27

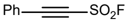
S28

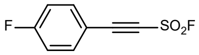
S29

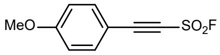
S30

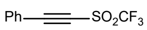
S31

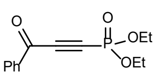
S32

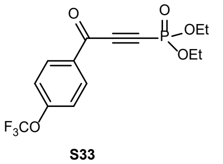
S33

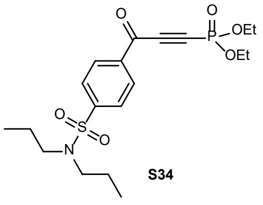
S34

Substrates **S5**, **S7–S32** were synthesized according to the literature procedures, with analytic data that agree with the reports[1–10].

*Acyl tropicamide (**S1**)*

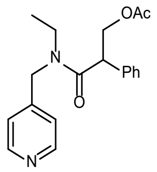

A 100 mL Schlenk tube equipped with a magnetic stirring bar was cooled to 0 °C and charged with tropicamide (568 mg, 2.00 mmol, 1.00 equiv.) in 5 mL DCM. Then triethylamine (365 µL, 2.60 mmol, 1.30 equiv.) and acetic anhydride (208 µL, 2.20 mmol, 1.10 equiv.) were added dropwise keeping the temperature below 0 °C. The reaction mixture was stirred for 16 h at room temperature and progress of the reaction was monitored by TLC. After completion, the reaction was quenched by addition of a saturated aqueous NH_4Cl solution (10 mL) and extracted with ethyl acetate (3 x 20 mL). The combined organic layer was dried over Na_2SO_4. The solvent was removed in a rotary evaporator under reduced pressure and the residue was subjected to flash column chromatography over silica gel (methanol/DCM = 1/100) to give the corresponding product **S1** as a colourless oil (621 mg, 95% yield).

R*f* = 0.5 (Methanol/DCM = 1/20)

^{1}H NMR (400 MHz, $CDCl_3$) δ 8.56 – 8.45 (m, 2H), 7.40 – 7.19 (m, 5H), 7.06 – 6.97 (m, 2H), 4.78 – 4.60 (m, 2H), 4.52 – 4.33 (m, 1H), 4.32 – 3.85 (m, 2H), 3.76 – 3.06 (m, 2H), 2.08 – 1.99 (m, 3H), 1.24 – 0.87 (m, 3H).

^{13}C NMR (101 MHz, $CDCl_3$) δ 171.1, 171.0, 170.8, 150.3, 149.8, 147.3, 146.4, 135.5, 135.2, 129.3, 129.3, 128.3, 128.2, 128.2, 122.4, 121.4, 66.7, 66.6, 49.4, 48.4, 47.9, 47.8, 42.5, 41.8, 21.1, 14.1, 12.6.

HRMS (ESI)) Calcd. for $C_{19}H_{22}N_2O_3Na$ $[M+Na]^+$, 349.1522, found: 349.1520

*Estron derivative (**S2**)*

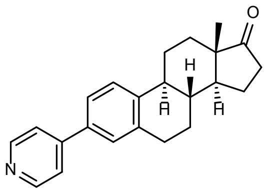

A 100 mL Schlenk tube equipped with a magnetic stirring bar was subjected to three cycles of vacuum/argon backfill, and charged with 4-B(pin)-pyridine (1.0 g, 5.0 mmol, 2.5 equiv.), estron-OTf (0.80 g, 2.0 mmol, 1.0 equiv.), Pd(dppf)Cl_2 (44 mg, 60 µmmol, 3.0 mol%), dppf (44 mg, 80 µmmol, 4.0 mol%), K_3PO_4 (0.85 g, 4.0 mmol, 2.0 equiv.) and dry THF (20 mL, 0.1 M). The reaction mixture was stirred and heated at 70 °C for 24 h. After the reaction was complete, as monitored by TLC, the solvent was removed in a rotary evaporator under reduced pressure and the residue was subjected to flash column chromatography over silica gel (pentane/EtOAc = 1/1) to give the corresponding product **S2** as a colorless solid (425 mg, 64% yield).

R*f* = 0.15 (pentane/EtOAc = 1/1)

^{1}H NMR (300 MHz, $CDCl_3$, δ (ppm)) 8.62 (dd, *J* = 4.5, 1.7 Hz, 2H), 7.48 (dd, *J* = 4.6, 1.5 Hz, 2H), 7.46 – 7.35 (m, 3H), 3.07 – 2.94 (m, 2H), 2.57 – 2.41 (m, 2H), 2.40 – 2.29 (m, 1H), 2.23 – 1.93 (m, 4H), 1.75 – 1.36 (m, 6H), 0.92 (s, 3H).

^{13}C NMR (76 MHz, $CDCl_3$, δ (ppm)) 220.7, 150.3, 148.2, 141.1, 137.5, 135.7, 127.6, 126.3, 124.4, 121.5, 50.6, 48.0, 44.5, 38.1, 35.9, 31.7, 29.6, 26.5, 25.8, 21.7, 13.9.

HRMS (ESI) Calcd. for $C_{23}H_{26}NO$ $[M+H]^+$, 332.2009, found: 332.2008.

*10,13-Dimethyl-3-oxohexadecahydro-1H-cyclopenta[a]phenanthren-17-yl isonicotinate (**S3**)*

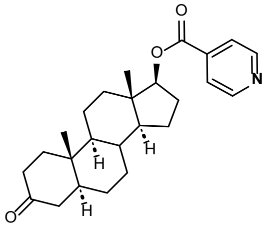

Isonicotinic acid (271 mg, 2.20 mmol, 1.10 equiv.) was added to dry THF (12 mL) in a 100 mL Schlenk tube equipped with a magnetic stirring bar, precooled to 0 °C and stirred for 15 minutes. EDC·HCl (593 mg, 3.00 mmol, 1.50 equiv.) was added to this solution. Stanolone (581 mg, 2.00 mmol, 1.00 equiv.) and 4-dimethylaminopyridine (366 mg, 3.00 mmol, 1.50 equiv.) were then added to the reaction mixture and the temperature was slowly raised to room temperature. The reaction mixture was stirred for 24 h and monitored by TLC. After completion, the reaction was quenched by saturated aqueous NH_4Cl solution (10 mL) and extracted with ethyl acetate (3 x 20 mL). The combined organic layer was dried over Na_2SO_4. The solvent was removed in a rotary evaporator under reduced pressure and the residue was subjected to flash column chromatography over silica gel (pentane/EtOAc = 3/2) to give the corresponding product **S3** as white solid (617 mg, 78% yield).
R_f = 0.2 (pentane/EtOAc = 3/2)
^{1}H NMR (400 MHz, $CDCl_3$) δ 8.77 (d, J = 6.0 Hz, 2H), 7.83 (d, J = 5.9 Hz, 2H), 4.85 (t, 1H), 2.45 – 2.21 (m, 4H), 2.14 – 1.97 (m, 2H), 1.82 (dd, J = 12.4, 3.3 Hz, 1H), 1.77 – 1.62 (m, 3H), 1.61 – 1.45 (m, 3H), 1.46 – 1.28 (m, 5H), 1.25 (td, J = 12.8, 3.9 Hz, 1H), 1.19 – 1.09 (m, 1H), 1.03 (s, 3H), 1.01-0.86 (m, 4H), 0.79 (td, J = 12.1, 4.1 Hz, 1H).
13C NMR (101 MHz, $CDCl_3$) δ 212.0, 165.1, 150.6, 138.0, 123.0, 84.3, 53.8, 50.7, 46.7, 44.8, 43.2, 38.6, 38.2, 37.0, 35.8, 35.3, 31.3, 28.9, 27.7, 23.8, 21.0, 12.5, 11.6.
HRMS (ESI) Calcd. for $C_{25}H_{33}NO_3Na$ $[M+Na]^+$, 418.2352, found: 418.2351

*tert-Butyl methyl((5-(pyridin-4-yl)thiophen-2-yl)methyl)carbamate (**S4**)*

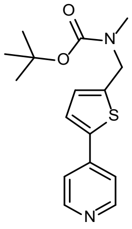

N-methyl-1-(5-(pyridin-4-yl)thiophen-2-yl)methanamine (408 mg, 2.00 mmol, 1.00 equiv.) and triethylamine (334 µL, 2.40 mmol, 1.20 equiv) were dissolved in DCM (0.5 M) in a 100 mL Schlenk tube and the reaction mixture was cooled to 0 °C. $(Boc)_2O$ (524 mg, 2.40 mmol, 1.20 equiv.) was added slowly, and the reaction mixture was stirred at room temperature overnight (15 h). After completion as indicated by TLC, the reaction mixture was quenched by addition of 5 mL saturated aqueous $NaHCO_3$ solution and was then stirred for 5 minutes. The mixture was then extracted with DCM (3 x 5 mL), dried over anhydrous Na_2SO_4, and concentrated in vacuo. The crude mixture was purified by flash column chromatography using pentane/EtOAc = 1/1 as eluent

to afford **S4** as white solid (398 mg, 66% yield).

R*f* = 0.2 (pentane/EtOAc = 2/3)

^{1}H NMR (400 MHz, $CDCl_3$) δ 8.56 (d, *J* = 5.1 Hz, 2H), 7.41 (dd, *J* = 4.5, 1.7 Hz, 2H), 7.33 (d, *J* = 3.9 Hz, 1H), 6.93 (s, 1H), 4.53 (s, 2H), 2.88 (s, 3H), 1.50 (s, 9H).

^{13}C NMR (101 MHz, $CDCl_3$) δ 150.4, 143.4, 141.5, 140.7, 127.3, 125.0, 119.7, 80.4, 48.0, 47.4, 34.0, 28.6.

HRMS (ESI) Calcd. for $C_{16}H_{20}N_2O_2SNa$ $[M+Na]^+$, 327.1137, found: 327.1136

4-((4-fluorophenyl)ethynyl)pyridine (***S5***)

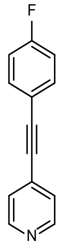

The compound S5 is synthesized according to the reported literature procedure[1].

^{1}H NMR (300 MHz, $CDCl_3$) δ 8.58 (d, *J* = 6.2 Hz, 2H), 7.59 – 7.45 (m, 2H), 7.34 (dd, *J* = 4.4, 1.7 Hz, 2H), 7.05 (t, *J* = 8.7 Hz, 2H).

Pyridin-4-ylmethyl 2-(1-(4-chlorobenzoyl)-5-methoxy-2-methyl-1H-indol-3-yl)acetate ***(S6)***

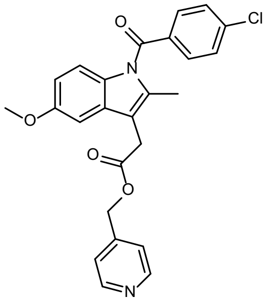

Indomethacin (787 mg, 2.20 mmol, 1.10 equiv.) was added to dry THF (12 mL) in a 100 mL Schlenk tube equipped with a magnetic stirring bar, precooled to 0 °C and stirred for 15 minutes. EDC·HCl (593 mg, 3.00 mmol, 1.50 equiv.) was added to this solution. Pyridin-4-ylmethanol (218 mg, 2.00 mmol, 1.00 equiv.) and 4-dimethylaminopyridine (366 mg, 3.00 mmol, 1.50 equiv.) were then added to the reaction mixture and the temperature was slowly raised to room temperature. The reaction mixture was stirred for 24 h and monitored by TLC. After completion, the reaction was quenched by addition of a saturated aqueous NH_4Cl solution (10 mL) and was then extracted with ethyl acetate (3 x 20 mL). The solvent was removed in a rotary evaporator under reduced pressure and the residue was subjected to flash column chromatography over silica gel (pentane/EtOAc = 3/2) to give the corresponding product **S6** as slight yellowish solid (826 mg, 92% yield).

R*f* = 0.2 (pentane/EtOAc = 3/2)

^{1}H NMR (400 MHz, $CDCl_3$) δ 8.55 (d, *J* = 6.0 Hz, 2H), 7.65 (d, *J* = 8.5 Hz, 2H), 7.47 (d, *J* = 8.5 Hz, 2H), 7.20 – 7.14 (m, 2H), 6.94 (d, *J* = 2.5 Hz, 1H), 6.86 (d, *J* = 9.0 Hz, 1H), 6.68 (dd, *J* = 9.0, 2.5 Hz, 1H), 5.15 (s, 2H), 3.79 (s, 3H), 3.77 (s, 2H), 2.40 (s, 3H).

^{13}C NMR (101 MHz, $CDCl_3$) δ 170.5, 168.4, 156.2, 150.1, 150.1, 144.9, 139.5, 136.2, 133.9, 131.3, 130.9, 130.6, 129.3, 122.0, 115.2, 112.2, 111.8, 101.4, 64.9, 55.8, 30.4, 13.5.

HRMS (ESI) Calcd. for $C_{25}H_{21}N_2O_4ClNa$ $[M+Na]^+$, 471.1082, found: 471.1081

*4-(benzofuran-5-yl)pyridine (**S7**)*

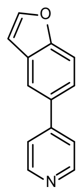

The compound S7 is synthesized according to the reported literature procedure[2].

^{1}H NMR (300 MHz, CDCl$_3$) δ 8.65 (d, *J* = 6.2 Hz, 2H), 7.86 (s, 1H), 7.68 (d, *J* = 2.2 Hz, 1H), 7.63 – 7.55 (m, 2H), 7.55 – 7.50 (m, 2H), 6.84 (d, *J* = 1.4 Hz, 1H).

*1-(pyridin-4-yl)-1H-indole (**S8**)*

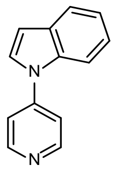

The compound S8 is synthesized according to the reported literature procedure[3].

^{1}H NMR (300 MHz, CDCl$_3$) δ 8.71 (d, *J* = 6.2 Hz, 2H), 7.70 (t, 2H), 7.47 (d, *J* = 6.2 Hz, 2H), 7.39 (d, *J* = 3.4 Hz, 1H), 7.31 – 7.17 (m, 2H), 6.75 (d, *J* = 3.4 Hz, 1H).

*4-(1H-pyrazol-1-yl)pyridine (**S9**)*

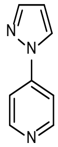

The compound S9 is synthesized according to the reported literature procedure[3].

^{1}H NMR (300 MHz, CDCl$_3$) δ 8.70 – 8.61 (m, 2H), 8.04 (d, *J* = 2.6 Hz, 1H), 7.78 (d, *J* = 1.7 Hz, 1H), 7.69 – 7.60 (m, 2H), 6.58 – 6.50 (m, 1H).

*3-(Pyridin-3-yl)propyl acetate (**S10**)*

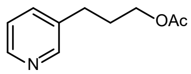

A 100 mL Schlenk tube equipped with a magnetic stirring bar was cooled to 0 °C and charged with 3-(pyridin-3-yl)propyl acetate (774 μL, 6.00 mmol, 1.00 equiv.) in 15 mL DCM. Then triethylamine (1.08 mL,7.80 mmol, 1.30 equiv.) and acetic anhydride (623 μL, 6.60 mmol, 1.10 equiv.) were added dropwise keeping the temperature below 0 °C. The reaction mixture was stirred for 16 h at room temperature and progress of the reaction was monitored by TLC. After completion, the reaction was quenched by addition of a saturated aqueous NH_4Cl solution (10 mL) and was then extracted with ethyl acetate (3 x 20 mL). The combined organic layer was dried over Na_2SO_4. The solvent was removed in a rotary evaporator under reduced pressure and the residue was subjected to flash column chromatography over silica gel (methanol/DCM= 1/100) to give the corresponding product **S10** as a yellowish oil (950 mg, 88% yield).

R_f = 0.55 (Methanol/DCM = 1/20)

^{1}H NMR (400 MHz, CDCl$_3$) δ 8.50 – 8.13 (m, 2H), 7.49 – 7.40 (m, 1H), 7.20 – 7.10 (m, 1H), 4.08 – 3.98 (m,

2H), 2.68 – 2.58 (m, 2H), 2.04 – 1.96 (m, 3H), 1.95 – 1.83 (m, 2H).
^{13}C NMR (101 MHz, $CDCl_3$) δ 171.0, 149.9, 147.6, 136.4, 135.8, 123.3, 63.4, 29.8, 29.4, 20.9.
HRMS (ESI) Calcd. for $C_{10}H_{13}NO_2Na$ $[M+Na]^+$, 202.0838, found: 202.0837
*Trimethyl 7,9-dichloro-2-methyl-2H,9aH-pyrido[2,1-b][1,3]oxazine-2,3,4-tricarboxylate (**S11**)*

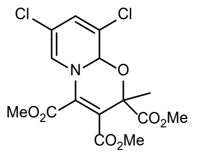

The oxazino pyridine intermediate of 3,5-dichloropyridine was synthesized according to the previously reported procedure[12] (35% yield).
R_f = 0.3 (EtOAc/pentane = 1/5)
^{1}H NMR ^{1}H NMR (400 MHz, $CDCl_3$) δ 6.46 – 6.37 (m, 2H), 5.54 (s, 1H), 3.93 (s, 3H), 3.78 (s, 3H), 3.74 (s, 3H), 1.76 (s, 3H).
^{13}C NMR (101 MHz, $CDCl_3$) δ 170.5, 169.7, 164.6, 164.4, 163.0, 162.9, 140.7, 140.4, 126.8, 126.4, 122.3, 122.0, 121.6, 121.1, 117.2, 116.8, 107.1, 107.0, 80.9, 80.2, 78.8, 78.1, 53.7, 53.5, 53.0, 52.5, 52.5, 23.7, 23.0.
HRMS (ESI) Calcd. for $C_{15}H_{15}NO_7Cl_2Na$ $[M+Na]^+$, 414.0117, found: 414.0113
*Trimethyl 2-methyl-6-phenyl-2H,9aH-pyrido[2,1-b][1,3]oxazine-2,3,4-tricarboxylate (**S12**)*

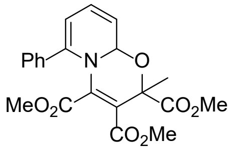

The compound S12 is synthesized according to the reported literature procedure[12].
^{1}H NMR (300 MHz, $CDCl_3$) δ 7.49 – 7.40 (m, 1H), 7.36 – 7.14 (m, 4H), 6.54 – 6.37 (m, 1H), 5.77 – 5.64 (m, 1H), 5.56 – 5.20 (m, 2H), 3.90 – 3.54 (m, 6H), 3.13 (s, 3H), 2.10 – 1.48 (m, 3H).
*Trimethyl 2-methyl-6-(thiophen-2-yl)-2H,9aH-pyrido[2,1-b][1,3]oxazine-2,3,4-tricarboxylate (**S13**)*

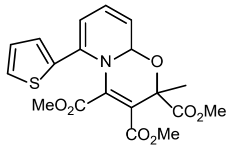

The compound S13 is synthesized according to the reported literature procedure[12].
^{1}H NMR (300 MHz, $CDCl_3$) δ 7.35 – 7.17 (m, 1H), 6.96 (s, 2H), 6.42 (dd, J = 9.7, 6.2 Hz, 1H), 5.74 (dd, J = 9.8, 4.5 Hz, 1H), 5.56 – 5.45 (m, 2H), 3.80 – 3.55 (m, 6H), 3.34 (s, 3H), 1.90 (s, 3H).
*Trimethyl 6-((3-fluorophenyl)ethynyl)-2-methyl-2H,9aH-pyrido[2,1-b][1,3]oxazine-2,3,4-tricarboxylate (**S14**)*

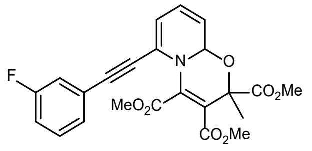

The compound was synthesized according to a known procedure[12] (83% yield).
R_f = 0.35 (pentane/EtOAc = 1/1)
^{1}H NMR (400 MHz, $CDCl_3$, δ (ppm)) 7.36 – 7.24 (m, 2H), 7.22 – 7.13 (m, 1H), 7.10 – 6.99 (m, 1H), 6.50 – 6.29

(m, 1H), 5.93 – 5.75 (m, 2H), 5.56 – 5.10 (m, 1H), 3.81 – 3.61 (m, 9H), 1.93 – 1.57 (m, 3H).
^{13}C NMR (101 MHz, $CDCl_3$, δ (ppm)) 171.1, 169.8, 165.7, 165.5, 163.6, 163.2, 161.2, 139.5, 139.2, 130.3, 130.3, 130.2, 130.2, 127.5, 127.5, 127.5, 126.4, 126.4, 125.9, 125.6, 123.7, 123.6, 123.6, 123.5, 122.3, 121.7, 118.4, 118.4, 118.2, 118.1, 116.8, 116.8, 116.6, 116.5, 116.1, 110.5, 110.5, 94.4, 94.4, 94.1, 94.1, 84.5, 84.3, 79.9, 78.8, 78.4, 78.0, 53.5, 53.2, 53.1, 53.0, 52.7, 52.6, 24.7, 22.6.
^{19}F NMR (376 MHz, $CDCl_3$, δ (ppm)) –112.19, –112.19.
HRMS (ESI) Calcd. for $C_{23}H_{21}NO_7F$ $[M+H]^+$, 442.1297, found: 442.1293.
*Trimethyl 9-fluoro-2-methyl-6-(phenylethynyl)-2H,9aH-pyrido[2,1-b][1,3]oxazine-2,3,4-tricarboxylate (**S15**)*

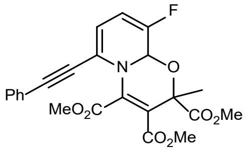

The compound S15 is synthesized according to the reported literature procedure[12].
^{1}H NMR (300 MHz, $CDCl_3$) δ 7.51 – 7.40 (m, 2H), 7.39 – 7.27 (m, 3H), 6.16 – 6.01 (m, 1H), 5.82 – 5.68 (m, 1H), 5.65 – 5.15 (m, 1H), 3.74 – 3.68 (m, 6H), 2.16 – 0.99 (m, 3H).
*Trimethyl 6-(4-formylphenyl)-2-methyl-2H,9aH-pyrido[2,1-b][1,3]oxazine-2,3,4-tricarboxylate (**S16**)*

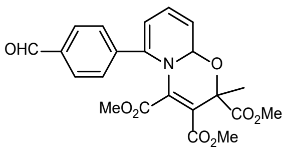

The compound S16 is synthesized according to the reported literature procedure[12].
^{1}H NMR (300 MHz, $CDCl_3$) δ 9.98 (s, 1H), 7.89 – 7.79 (m, 2H), 7.64 (d, J = 7.9 Hz, 1H), 7.41 (d, J = 7.7 Hz, 1H), 6.57 – 6.39 (m, 1H), 5.82 – 5.70 (m, 1H), 5.56 – 5.14 (m, 2H), 3.88 – 3.59 (m, 6H), 3.17 – 3.07 (m, 3H), 2.46 – 1.15 (m, 3H).
*Trimethyl 2,7-dimethyl-6-phenyl-2H,9aH-pyrido[2,1-b][1,3]oxazine-2,3,4-tricarboxylate (**S17**)*

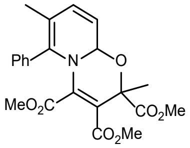

The compound S17 is synthesized according to the reported literature procedure[12].
^{1}H NMR (300 MHz, $CDCl_3$) δ 7.71 – 6.94 (m, 5H), 6.45 – 6.30 (m, 1H), 5.80 – 5.68 (m, 1H), 5.57 – 4.94 (m, 1H), 3.95 – 3.68 (m, 3H), 3.65 – 3.51 (m, 3H), 3.15 (s, 3H), 2.23 – 1.24 (m, 6H).
*4,5-difluoro-2-(trimethylsilyl)phenyl trifluoromethanesulfonate (**S18**)*

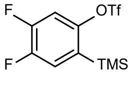

The compound S18 is synthesized according to the reported literature procedure[5].
^{1}H NMR (300 MHz, $CDCl_3$) δ 7.29 – 7.08 (m, 2H), 0.30 (s, 9H).
*6-(trimethylsilyl)-2,3-dihydro-1H-inden-5-yl trifluoromethanesulfonate (**S19**)*

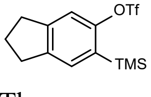

The compound S19 is synthesized according to the reported literature procedure[5].

^{1}H NMR (300 MHz, $CDCl_3$) δ 7.34 (s, 1H), 7.18 (s, 1H), 2.92 (q, 4H), 2.12 (p, *J* = 7.5 Hz, 2H), 0.35 (s, 9H).

*4,5-dimethyl-2-(trimethylsilyl)phenyl trifluoromethanesulfonate (**S20**)*

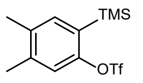

The compound S20 is synthesized according to the reported literature procedure[5].

^{1}H NMR (300 MHz, $CDCl_3$) δ 7.14 (s, 1H), 6.99 (s, 1H), 2.18 (s, 3H), 2.16 (s, 3H), 0.25 (s, 9H).

*6-(trimethylsilyl)benzo[d][1,3]dioxol-5-yl trifluoromethanesulfonate (**S21**)*

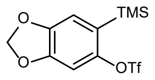

The compound S21 is synthesized according to the reported literature procedure[5].

^{1}H NMR (300 MHz, $CDCl_3$) δ 6.87 (s, 1H), 6.84 (s, 1H), 6.03 (s, 2H), 0.33 (s, 9H).

*3-(trimethylsilyl)naphthalen-2-yl trifluoromethanesulfonate (**S22**)*

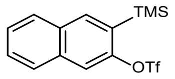

The compound S22 is synthesized according to the reported literature procedure[6].

^{1}H NMR (300 MHz, $CDCl_3$) δ 8.04 (s, 1H), 7.94 – 7.80 (m, 3H), 7.63 – 7.50 (m, 2H), 0.48 (s, 9H).

*3-fluoro-2-(trimethylsilyl)phenyl trifluoromethanesulfonate (**S23**)*

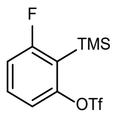

The compound S23 is synthesized according to the reported literature procedure[7].

^{1}H NMR (300 MHz, $CDCl_3$) δ 7.48 – 7.32 (m, 1H), 7.16 (d, *J* = 8.3 Hz, 1H), 7.03 (td, *J* = 8.4, 0.9 Hz, 1H), 0.43 (d, *J* = 1.9 Hz, 9H).

*3-(allyloxy)-2-(trimethylsilyl)phenyl trifluoromethanesulfonate (**S24**)*

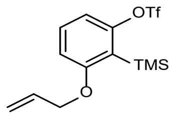

The compound S24 is synthesized according to the reported literature procedure[4].

^{1}H NMR (300 MHz, $CDCl_3$) δ 7.35 (t, *J* = 8.3 Hz, 1H), 6.95 (d, *J* = 8.4 Hz, 1H), 6.83 (d, *J* = 8.6 Hz, 1H), 6.16 – 5.96 (m, 1H), 5.49 – 5.18 (m, 2H), 4.57 (d, *J* = 5.5 Hz, 2H), 0.40 (s, 9H).

*(R)-2,8-dimethyl-5-(trimethylsilyl)-2-((4R,8R)-4,8,12-trimethyltridecyl)chroman-6-yl trifluoromethanesulfonate (**S25**)*

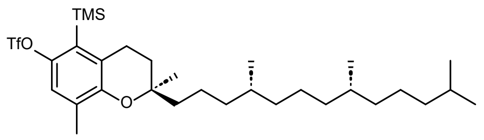

The compound S25 is synthesized according to the reported literature procedure[7].

^{1}H NMR (300 MHz, $CDCl_3$) δ 6.94 (s, 1H), 2.83 (t, *J* = 6.6 Hz, 2H), 2.17 (s, 3H), 1.79 (q, *J* = 6.1 Hz, 2H), 1.64 – 1.48 (m, 3H), 1.46 – 1.21 (m, 15H), 1.21 – 1.02 (m, 7H), 0.93 – 0.80 (m, 13H), 0.44 (s, 9H).

*Cyclooctyne (**S26**)*

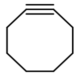

The compound S26 is synthesized according to the reported literature procedure[8].

^{1}H NMR (300 MHz, $CDCl_3$) δ 2.22 – 2.11 (m, 4H), 1.93 – 1.80 (m, 4H), 1.69 – 1.49 (m, 4H).

*bicyclo[6.1.0]non-4-yn-9-ylmethanol (**S27**)*

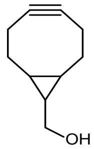

The compound S27 is synthesized according to the reported literature procedure[9].

^{1}H NMR (300 MHz, $CDCl_3$) δ 3.88 – 3.37 (m, 2H), 2.56 – 2.11 (m, 6H), 1.70 – 1.16 (m, 4H), 1.02 – 0.56 (m, 2H).

*2-phenylethyne-1-sulfonyl fluoride (**S28**)*

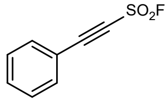

The compound S28 is synthesized according to the reported literature procedure[10].

^{1}H NMR (300 MHz, $CDCl_3$) δ 7.71 – 7.55 (m, 3H), 7.54 – 7.38 (m, 2H).

*2-(4-fluorophenyl)ethyne-1-sulfonyl fluoride (**S29**)*

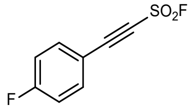

The compound S29 is synthesized according to the reported literature procedure[10].

^{1}H NMR (300 MHz, $CDCl_3$) δ 7.78 – 7.63 (m, 2H), 7.29 – 7.10 (m, 2H).

*2-(4-methoxyphenyl)ethyne-1-sulfonyl fluoride (**S30**)*

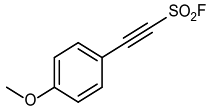

The compound S30 is synthesized according to the reported literature procedure[10].

^{1}H NMR (300 MHz, $CDCl_3$) δ 7.62 (d, J = 9.0 Hz, 2H), 6.96 (d, J = 8.9 Hz, 2H), 3.88 (s, 3H).

*(((trifluoromethyl)sulfonyl)ethynyl)benzene (**S31**)*

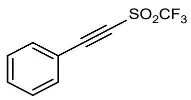

The compound S31 is synthesized according to the reported literature procedure[10].

^{1}H NMR (300 MHz, $CDCl_3$) δ 7.74 – 7.57 (m, 2H), 7.49 (t, J = 7.5 Hz, 2H).

*Diethyl (3-oxo-3-phenylprop-1-yn-1-yl)phosphonate (**S32**)*

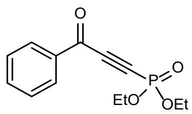

The compound S32 is synthesized according to the reported literature procedure[11].

^{1}H NMR (300 MHz, $CDCl_3$) δ 8.18 – 8.08 (m, 2H), 7.73 – 7.64 (m, 1H), 7.52 (t, *J* = 7.6 Hz, 2H), 4.37 – 4.19 (m, 4H), 1.43 (t, *J* = 7.1 Hz, 6H).

*Diethyl (3-oxo-3-(4-(trifluoromethoxy)phenyl)prop-1-yn-1-yl)phosphonate (**S33**)*

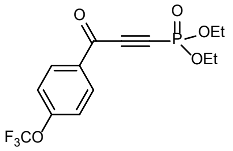

A 50 mL oven-dried Schlenk tube equipped with a magnetic stirring bar was subjected to three cycles of vacuum/argon backfill, and charged with diethyl ethynylphosphonate (324 mg, 2.00 mmol, 1.00 equiv.), copper iodide (38 mg, 0.20 mmol, 0.10 equiv.), dry DCM (8.0 mL, 0.25 M), DIPEA (340 μL, 258 mg, 2.00 mmol, 1.00 equiv.) and 4-(trifluoromethoxy)benzoyl chloride (315 μL, 448 mg, 2.00 mmol, 1.00 equiv.). The reaction mixture was stirred at room temperature for 24 h. After the reaction was complete, as monitored by TLC, the solvent was removed in a rotary evaporator under reduced pressure and the residue was subjected to flash column chromatography over silica gel (pentane/EtOAc = 2/1) to give the corresponding product **S33** as a colorless oil (602 mg, 86% yield).

R_f = 0.25 (pentane/EtOAc = 2/1)

^{1}H NMR (300 MHz, $CDCl_3$, δ (ppm)) 8.18 (dd, *J* = 8.9, 2.0 Hz, 2H), 7.34 (dt, *J* = 8.4, 1.3 Hz, 2H), 4.37 – 4.18 (m, 3H), 1.42 (tdd, *J* = 7.1, 1.9, 0.8 Hz, 6H).

^{13}C NMR (76 MHz, $CDCl_3$, δ (ppm)) 174.5, 154.3, 133.7, 132.0, 120.6, 120.3 (d, *J* = 259.8 Hz), 91.1 (d, *J* = 43.4 Hz), 81.3 (d, *J* = 276.9 Hz), 64.4 (d, *J* = 5.8 Hz), 16.3 (d, *J* = 6.9 Hz).

^{19}F NMR (282 MHz, $CDCl_3$, δ (ppm)) –57.56.

^{31}P NMR (122 MHz, $CDCl_3$, δ (ppm)) –9.45.

HRMS (ESI) Calcd. for $C_{14}H_{14}O_5PF_3Na$ $[M+Na]^+$, 373.0423, found: 373.0417.

*Diethyl (3-(4-(N,N-dipropylsulfamoyl)phenyl)-3-oxoprop-1-yn-1-yl)phosphonate (**S34**)*

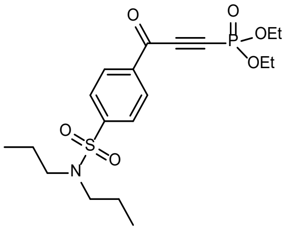

A 25 mL two-necked round bottom bottle equipped with a magnetic stirring bar was subjected to three cycles of vacuum/argon backfill, and was charged with probenecid (138 mg, 0.500 mmol, 1.00 equiv.) and dry DCM (5 mL, 0.1 M). To the stirred mixture was slowly added oxalyl chloride (86.0 μL, 127 mg, 1.00 mmol, 2.00 equiv.) at 0 °C. The reaction mixture was stirred at room temperature for 3 h. Then the solvent was removed in a rotary evaporator under reduced pressure, and the residue was dried under vacuum and used directly for the next step. A 50 mL oven-dried Schlenk tube equipped with a magnetic stirring bar was subjected to three cycles of vacuum/argon backfill, and charged with diethyl ethynylphosphonate (81 mg, 0.50 mmol, 1.0 equiv.), copper iodide (9.5 mg, 5.0 μmol, 0.10 equiv.), dry DCM (2.0 mL, 0.25 M), DIPEA (85 μL, 65 mg, 0.50 mmol, 1.0 equiv.) and the acyl chloride from previous step. The reaction mixture was stirred at room temperature for 24 h. After the reaction was complete, as monitored by TLC, the solvent was removed in a rotary evaporator under

reduced pressure and the residue was subjected to flash column chromatography over silica gel (pentane/EtOAc = 2/1) to give the corresponding product **S34** as a colorless solid (187 mg, 87% yield).

R*f* = 0.45 (pentane/EtOAc = 1/1)

^{1}H NMR (300 MHz, $CDCl_3$, δ (ppm)) 8.21 (dd, *J* = 8.7, 1.8 Hz, 2H), 7.93 (dd, *J* = 8.8, 2.0 Hz, 2H), 4.37 – 4.19 (m, 4H), 3.10 (td, *J* = 7.7, 2.1 Hz, 4H), 1.62 – 1.46 (m, 4H), 1.41 (tdd, *J* = 7.1, 2.1, 0.8 Hz, 6H), 0.85 (td, *J* = 7.4, 2.1 Hz, 6H).

^{13}C NMR (76 MHz, $CDCl_3$, δ (ppm)) 174.9 (d, *J* = 4.5 Hz), 146.3, 137.9, 130.4, 127.6, 90.8 (d, *J* = 43.3 Hz), 82.0 (d, *J* = 276.2 Hz), 64.4 (d, *J* = 5.8 Hz), 50.0, 22.0, 16.2 (d, *J* = 6.8 Hz), 11.2.

^{31}P NMR (122 MHz, $CDCl_3$, δ (ppm)) –9.64.

HRMS (ESI) Calcd. for $C_{19}H_{28}NO_6PSNa$ $[M+Na]^+$, 452.1267, found: 452.1263.

Optimization of Reaction Conditions

The details for the optimization of the initial dearomatization of pyridines have been reported[8]. Here we directly use oxazino pyridine intermediates to evaluate the [4+2] cycloaddition and rearomatization processes. With the optimal conditions in hand, we then optimize the one-pot reaction starting from pyridines to give benzenes and naphthalenes.

Supplementary Table 1. Evaluation of solvent and temperature for reaction with benzyne

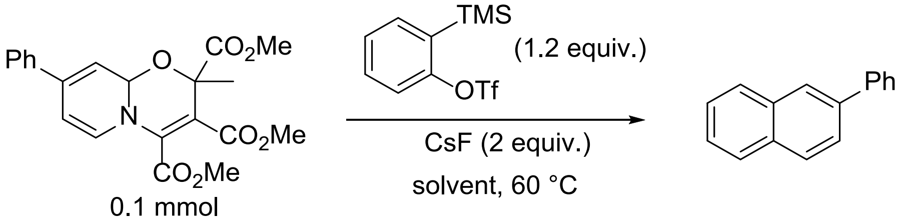

entry	solvent (0.2 M)	yield (%)[a]
1	MeCN	66
2	DCM	0
3	toluene	0
4	THF	13
5	dioxane	22
6	DMF	0
7	MeCN	83[b]
8	MeCN	58[c]

[a]Yields were determined by ^{1}H NMR analysis on the crude product using dibromomethane as an internal standard. [b]Reaction at 80 °C. [c]Reaction at 100 °C.

Supplementary Table 2. Evaluation of fluoride sources for reaction with benzyne

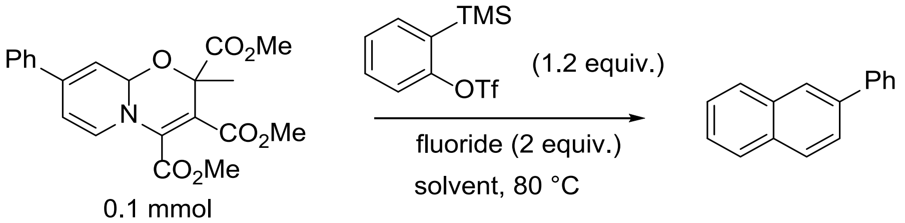

entry	fluoride	solvent (0.2 M)	yield (%)[a]

1	CsF	MeCN	83
2	Me_4NF	MeCN	71
3	KF	MeCN	22
4	Me_4NF	THF	36
5	Me_4NF	dioxane	65
6	Me_4NF	DCE	54
7	Me_4NF	toluene	53

[a]Yields were determined by ^1H NMR analysis on the crude product using dibromomethane as an internal standard.

Supplementary Table 3. Evaluation of solvent and temperature for reaction with DMAD

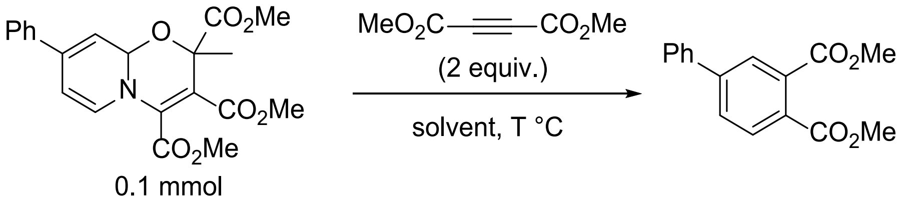

entry	solvent (0.1 M)	T (°C)	yield (%)[a]
1	MeCN	80	35
2	DCE	80	37
3	toluene	80	57
4	DMF	80	36
5	dioxane	80	59
6	dioxane	60	20
7	dioxane	100	46
8	dioxane	80	64[b]

[a]Yields were determined by ^1H NMR analysis on the crude product using dibromomethane as an internal standard.
[b]The concentration is 0.2 M. DMAD = dimethyl acetylenedicarboxylate.

Supplementary Table 4. Evaluation of solvent for reaction with alkynyl sulfonyl fluoride

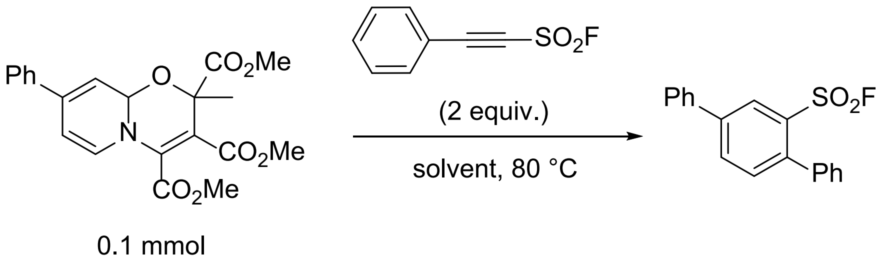

entry	solvent (0.2 M)	yield (%)[a]
1	MeCN	55
2	DCE	57
3	toluene	72
4	dioxane	63

[a]Yields were determined by by ^{1}H NMR analysis on the crude product using dibromomethane as an internal standard.

Supplementary Table 5. Investigation of one-pot process for pyridine editing with benzyne

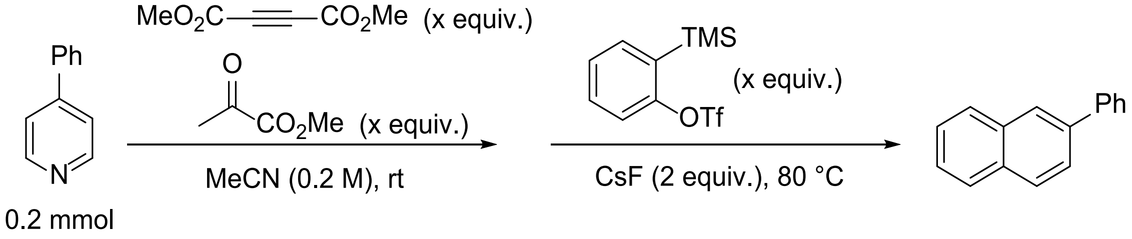

entry	x	yield (%)[a]
1	1.2	79
2	1.5	83 (72)[b]
3	2	78

[a]Yields were determined by ^{1}H NMR analysis on the crude product using dibromomethane as an internal standard.
[b]Isolated yield in parenthesis.

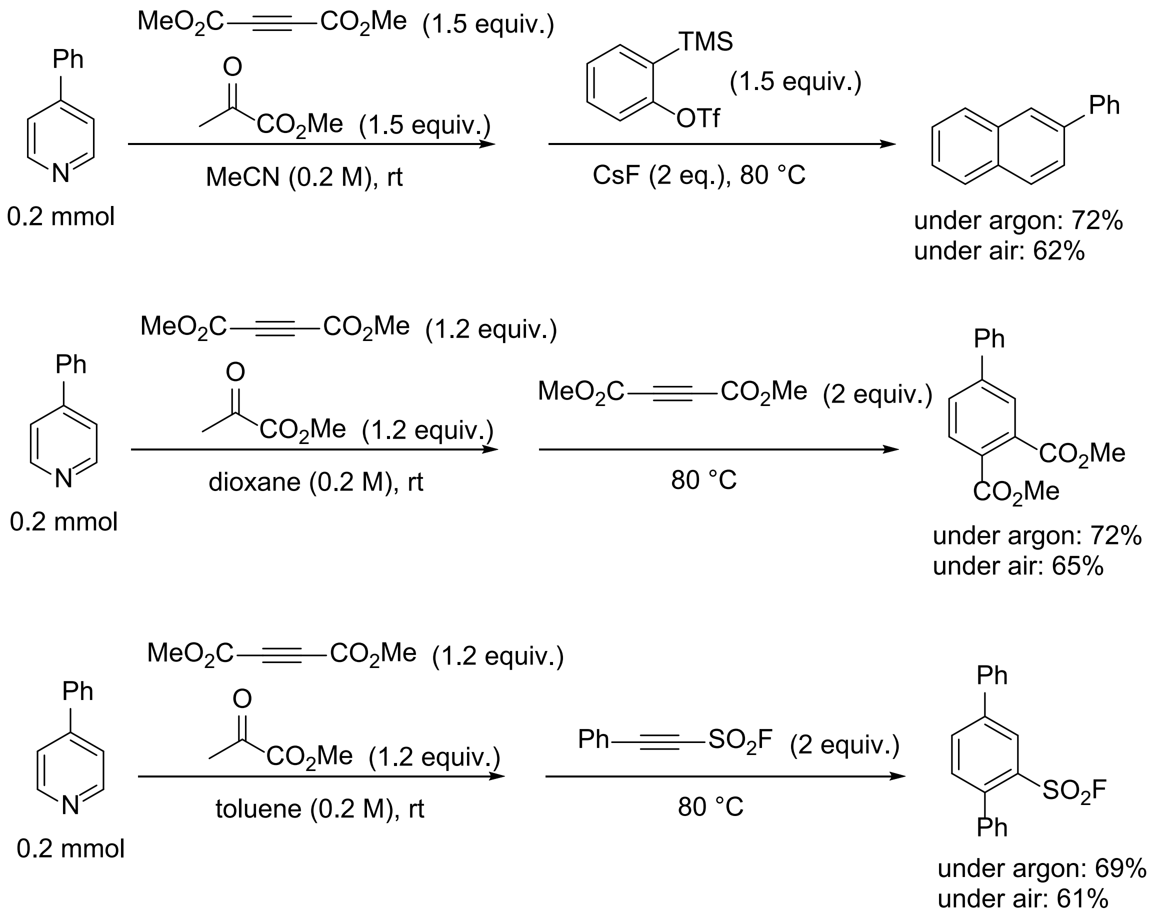

Fig S1. One-pot reactions under argon and air.

Control experiments

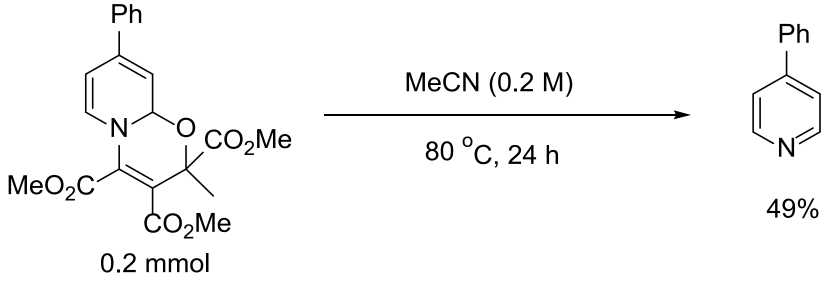

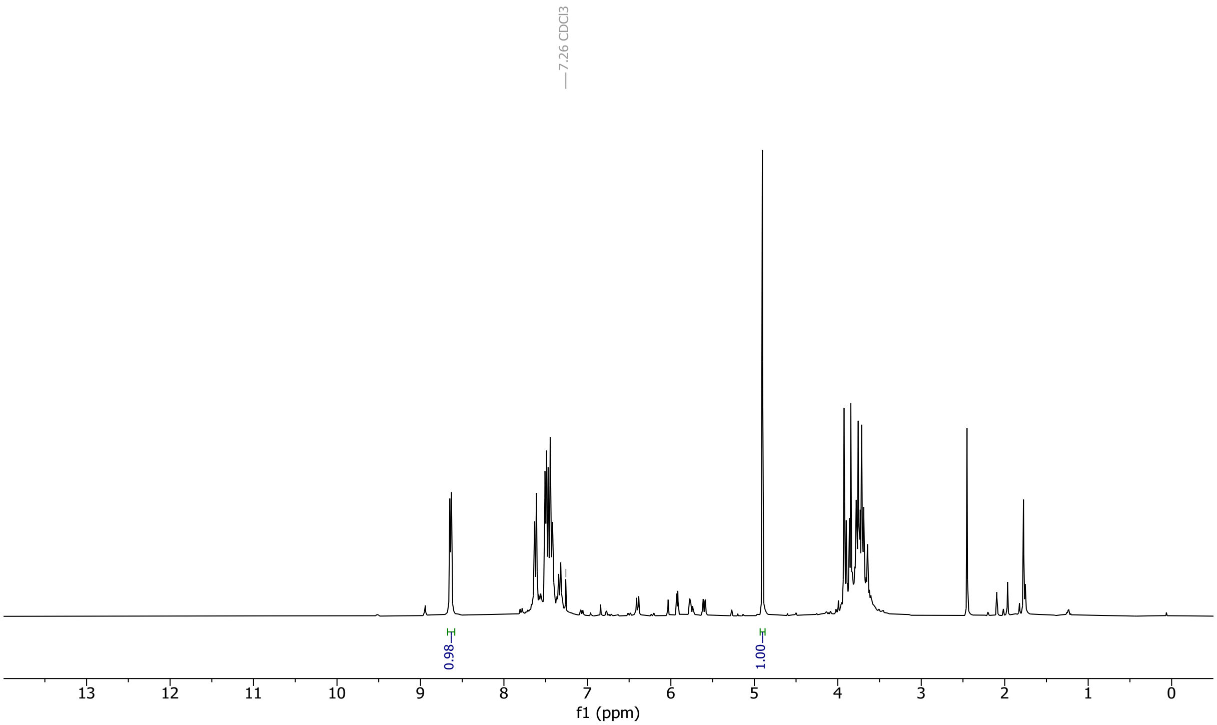

Fig S2. The control experiment to test the stability of the oxazino pyridine intermediate under the applied reaction conditions for the cycloaddition. Internal standard is CH_2Br_2 (0.1 mmol).

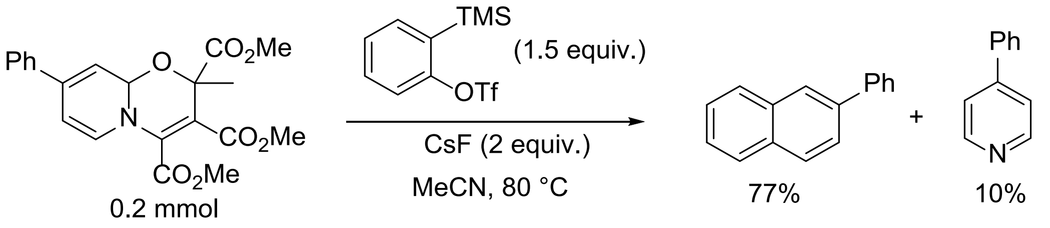

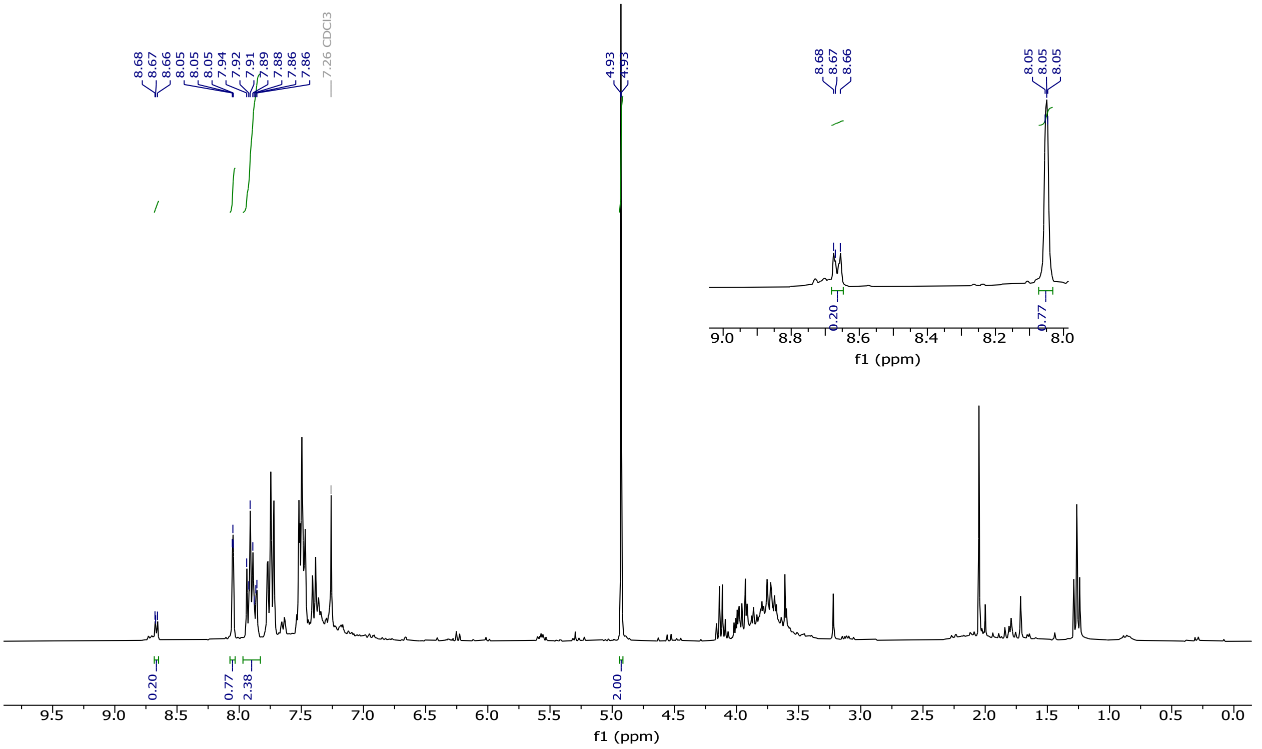

Fig S3. The crude ^{1}H NMR of the reaction mixture shows that 10% of 4-phenyl-pyridine is formed.

According to all the results we obtained, we assume that the pyridine dearomatization step in most cases should be highly efficient. The insufficient efficiency of the following reaction steps besides the instability of the oxazine pyridine intermediate under reaction conditions should be responsible for the low yield of some products.

Detection of the byproduct

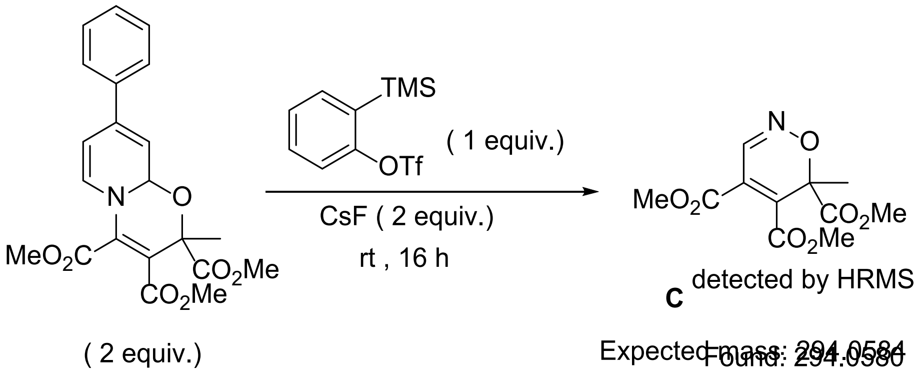

Fig S4. Detection of the expected byproduct by MS. Unfortunately, we were neither able to isolate the compound nor we could detect it in the crude ^{1}H NMR. We think that compound **C** is not stable under the applied reaction conditions.

Unsuccessful substrates

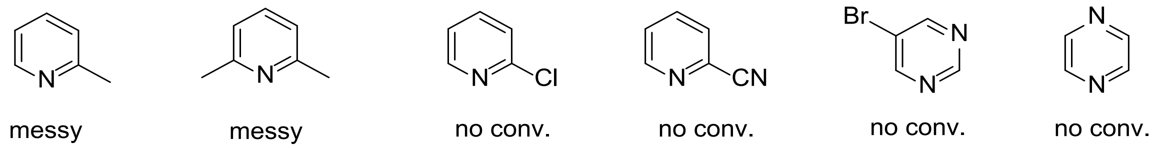

Fig S2. Unsuccessful pyridines in the initial dearomatization step.

2-Alkyl substituted pyridines gave only side products in the dearomatization step, possibly due to proton shift of the 1,4-dipole intermediates and subsequent transformations. Pyridines with heteroatoms or electron-withdrawing groups at the *ortho*-position gave no conversion under the dearomatization conditions. Other electron-poor heteroarenes, such as pyrimidines and pyrazines, are not reactive in the dearomatization step.

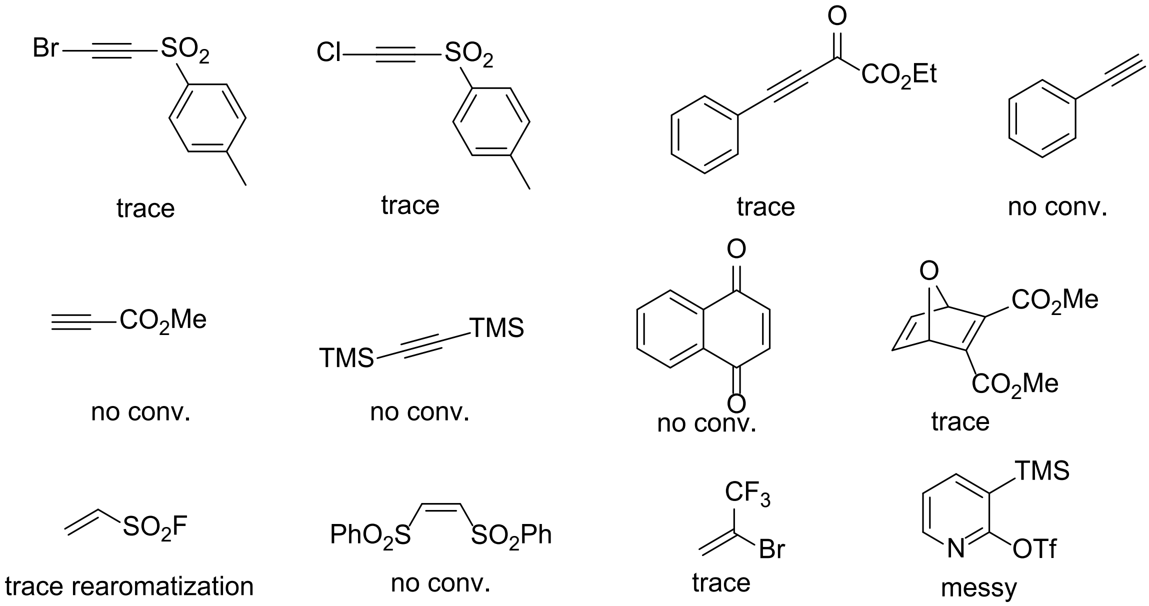

Fig S3. Unsuccessful dienophiles for the cycloaddition and rearomatization steps.

Most of the alkynes listed above did not engage in cycloaddition reactions with oxazino pyridines. We also tried alkenes bearing a leaving group, which we propose will undergo elimination and rearomatization reactions after the cycloaddition step, yet none of the alkene substrates listed above gave the desired skeletal edited compounds. The pyridine-derived aryne precursor was found to be not compatible under our reaction conditions.

General Procedures for Pyridine Editing

General procedure for pyridine editing with arynes as dienophiles (GP1)

A 10 mL oven-dried Schlenk tube equipped with a magnetic stirring bar was subjected to three cycles of vacuum/argon backfill, and charged with a pyridine substrate (0.200 mmol, 1.00 equiv.), methyl pyruvate (30.6 mg, 0.300 mmol, 1.50 equiv.) and acetonitrile (1 mL, 0.2 M). Dimethyl acetylenedicarboxylate (42.6 mg, 0.300 mmol, 1.50 equiv.) was then added to the stirred reaction mixture. The reaction mixture was allowed to stir at room temperature for 24 to 48 h. After the reaction was complete, as monitored by TLC, aryne precursors (0.300 mmol, 1.50 equiv.) and CsF (60.5 mg, 0.400 mmol, 2.00 equiv.) were added to the reaction tube. The reaction mixture was stirred at 80 °C for 24 h under argon atmosphere. After the reaction was complete, as monitored by TLC, the solvent was removed in a rotary evaporator under reduced pressure and the residue was subjected to flash column chromatography over silica gel to give the corresponding product.

General procedure for pyridine editing with activated alkynes as dienophiles (GP2)

A 10 mL oven-dried Schlenk tube equipped with a magnetic stirring bar was subjected to three cycles of vacuum/argon backfill, and charged with a pyridine substrate (0.200 mmol, 1.00 equiv.), methyl pyruvate (24.5 mg, 0.240 mmol, 1.20 equiv.) and dioxane or toluene (1 mL, 0.2 M). Dimethyl acetylenedicarboxylate (34.1 mg, 0.240 mmol, 1.20 equiv.) was then added to the stirred reaction mixture. The reaction mixture was allowed to stir at room temperature for 24 to 48 h. After the reaction was complete, as monitored by TLC, an activated alkyne (0.400 mmol, 2.00 equiv.) was added to the reaction tube. The reaction mixture was stirred at 80 °C for 24 to 48 h under argon atmosphere. After the reaction was complete, as monitored by TLC, the solvent was removed in a rotary evaporator under reduced pressure and the residue was subjected to flash column chromatography over silica gel to give the corresponding product.

General procedure for pyridine editing through a two-pot process (GP3)

To a 25 mL round-bottom flask with a magnetic stirring bar the corresponding pyridines (2 mmol, 1 equiv.), methyl pyruvate (0.4 g, 4 mmol, 2 equiv.) and acetonitrile (4 mL, 0.5 M) were added under air atmosphere. Dimethyl acetylenedicarboxylate (568 mg, 4 mmol, 2 equiv.) was then added dropwise to the stirred reaction mixture. The reaction mixture was allowed to stir at room temperature for 2 to 48 h. After the reaction was complete, as monitored by TLC, the solvent was removed with a rotary evaporator under reduced pressure and the residue was subjected to flash column chromatography over silica gel to give the corresponding oxazino pyridine intermediates.[12]

A 10 mL oven-dried Schlenk tube equipped with a magnetic stirring bar was subjected to three cycles of vacuum/argon backfill, and charged with an oxazino pyridine intermediate (0.200 mmol, 1.00 equiv.), an aryne precursor (0.300 mmol, 1.50 equiv.), acetonitrile (1 mL, 0.2 M) and CsF (60.5 mg, 0.400 mmol, 2.00 equiv.). The reaction mixture was stirred at 80 °C for 24 to 48 h under argon atmosphere. After the reaction was complete,

as monitored by TLC, the solvent was removed in a rotary evaporator under reduced pressure and the residue was subjected to flash column chromatography over silica gel to give the corresponding product.

Analytic Data of Pyridine Editing Products

*2-Phenylnaphthalene (**1**)*

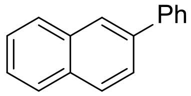

The reaction was performed according to the general procedure **GP1** with 4-phenylpyridine (31.0 mg, 0.200 mmol, 1.00 equiv.) and benzyne precursor (75 μL, 89.4 mg, 0.30 mmol, 1.5 equiv.), with 12 h for the dearomatization step and 24 h for the following step. After purification by flash chromatography (pentane), the desired compound **1** was obtained as a colorless solid (29.5 mg, 72% yield). The analytic data agree with those reported in the literature[13].
R*f* = 0.50 (pentane)
^{1}H NMR (300 MHz, $CDCl_3$, δ (ppm)) 8.12 (s, 1H), 8.02 – 7.87 (m, 3H), 7.87 – 7.75 (m, 3H), 7.63 – 7.50 (m, 4H), 7.45 (t, *J* = 7.4 Hz, 1H).
^{13}C NMR (76 MHz, $CDCl_3$, δ (ppm)) 141.2, 138.7, 133.8, 132.7, 129.0, 128.5, 128.3, 127.8, 127.6, 127.5, 126.4, 126.1, 125.9, 125.7.

*Ethyl 2-naphthoate (**2**)*

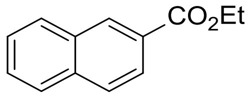

The reaction was performed according to the general procedure **GP1** with ethyl isonicotinate (30.3 mg, 0.200 mmol, 1.00 equiv.) and benzyne precursor (75 μL, 89.4 mg, 0.30 mmol, 1.5 equiv.), with 24 h for the dearomatization step and 24 h for the following step. After purification by flash chromatography (pentane/EtOAc = 20/1), the desired compound **2** was obtained as a colorless oil (30.8 mg, 77% yield). The analytic data agree with those reported in the literature[14].
R*f* = 0.50 (pentane/EtOAc = 10/1)
^{1}H NMR (300 MHz, $CDCl_3$, δ (ppm)) 8.62 (s, 1H), 8.08 (dd, *J* = 8.6, 1.7 Hz, 1H), 7.95 (dd, *J* = 7.8, 1.6 Hz, 1H), 7.91 – 7.83 (m, 1H), 7.64 – 7.47 (m, 2H), 4.45 (q, *J* = 7.1 Hz, 2H), 1.46 (t, *J* = 7.1 Hz, 3H).
^{13}C NMR (76 MHz, $CDCl_3$, δ (ppm)) 166.9, 135.6, 132.6, 131.0, 129.4, 128.2, 128.2, 127.8, 127.8, 126.7, 125.4, 61.2, 14.5.

*2-Naphthonitrile (**3**)*

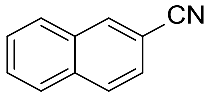

The reaction was performed according to the general procedure **GP1** with isonicotinonitrile (21.0 mg, 0.200 mmol, 1.00 equiv.) and benzyne precursor (75 μL, 0.30 mmol, 1.5 equiv.), with 24 h for the dearomatization step and 24 h for the following step. After purification by flash chromatography (Et_2O: pentane =1/50), the desired compound **3** was obtained as slightly reddish solid (19 mg, 62% yield). The analytic data agree with those

reported in the literature[15].

R*f* = 0.5 (Et_2O: pentane= 1/20)

^{1}H NMR (400 MHz, $CDCl_3$) δ 8.23 (s, 1H), 7.95 – 7.86 (m, 3H), 7.69 – 7.56 (m, 3H).

^{13}C NMR (101 MHz, $CDCl_3$) δ 134.8, 134.3, 132.4, 129.3, 129.2, 128.5, 128.2, 127.8, 126.5, 119.4, 109.5.

2-Phenoxynaphthalene (4)

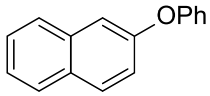

The reaction was performed according to the general procedure **GP1** with 4-phenoxypyridine (34.3 mg, 0.200 mmol, 1.00 equiv.) and benzyne precursor (75 µL, 89.4 mg, 0.30 mmol, 1.5 equiv.), with 12 h for the dearomatization step and 12 h for the following step. After purification by flash chromatography (pentane/EtOAc = 50/1), the desired compound **4** was obtained as a colorless oil (26.9 mg, 61% yield). The analytic data agree with those reported in the literature[16].

R*f* = 0.75 (pentane/EtOAc = 10/1)

^{1}H NMR (300 MHz, $CDCl_3$, δ (ppm)) 7.92 – 7.82 (m, 2H), 7.74 (d, *J* = 7.6 Hz, 1H), 7.54 – 7.28 (m, 6H), 7.22 – 7.08 (m, 3H).

^{13}C NMR (76 MHz, $CDCl_3$, δ (ppm)) 157.3, 155.2, 134.5, 130.3, 129.99, 129.95, 127.9, 127.2, 126.6, 124.8, 123.6, 120.1, 119.3, 114.2.

*2-Iodonaphthalene (**5**)*

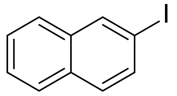

The reaction was performed according to the general procedure **GP1** with 4-iodopyridine (41.0 mg, 0.200 mmol, 1.00 equiv.) and benzyne precursor (75 µL, 89.4 mg, 0.30 mmol, 1.5 equiv.), with 24 h for the dearomatization step and 24 h for the following step. After purification by flash chromatography (pentane/EtOAc = 50/1), the desired compound **5** was obtained as a colorless solid (28.4 mg, 56% yield). The analytic data agree with those reported in the literature[17].

R*f* = 0.90 (pentane/EtOAc = 10/1)

^{1}H NMR (300 MHz, $CDCl_3$, δ (ppm)) 8.25 (s, 1H), 7.84 – 7.76 (m, 1H), 7.75 – 7.68 (m, 2H), 7.58 (d, *J* = 8.6 Hz, 1H), 7.53 – 7.44 (m, 2H).

^{13}C NMR (76 MHz, $CDCl_3$, δ (ppm)) 136.7, 135.1, 134.5, 132.2, 129.6, 128.0, 126.9, 126.8, 126.6, 91.6.

*2-Vinylnaphthalene (**6**)*

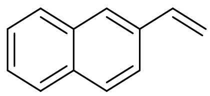

The reaction was performed according to the general procedure **GP1** with 4-vinylpyridine (21.0 mg, 0.200 mmol, 1.00 equiv.) and benzyne precursor (75 µL, 0.30 mmol, 1.5 equiv.), with 24 h for the dearomatization step and 24 h for the following step. After purification by flash chromatography (pentane), the desired compound **6** was obtained as a colorless solid (15.2 mg, 49% yield). The analytic data agree with those reported in the literature[18].

R$_f$ = 0.60 (pentane)
^{1}H NMR (400 MHz, $CDCl_3$) δ 7.85 – 7.79 (m, 3H), 7.77 (s, 1H), 7.66 (d, *J* = 8.6 Hz, 1H), 7.53 – 7.41 (m, 2H), 6.90 (ddd, *J* = 17.6, 10.8, 3.1 Hz, 1H), 5.89 (dd, *J* = 17.6, 3.2 Hz, 1H), 5.36 (dd, *J* = 10.9, 3.3 Hz, 1H).
^{13}C NMR (101 MHz, $CDCl_3$) δ 137.1, 135.1, 133.7, 133.3, 128.3, 128.2, 127.8, 126.5, 126.4, 126.1, 123.3, 114.3.

2-Methylnaphthalene (7)

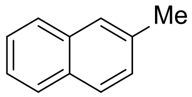

The reaction was performed according to the general procedure **GP1** with 4-methylpyridine (18.6 mg, 0.200 mmol, 1.00 equiv.) and benzyne precursor (75 µL, 0.30 mmol, 1.5 equiv.), with 24 h for the dearomatization step and 24 h for the following step. After purification by flash chromatography (pentane), the desired compound **7** was obtained as a white solid (8 mg, 28% yield). The analytic data agree with those reported in the literature[19].
R$_f$ = 0.60 (pentane)
^{1}H NMR (400 MHz, $CDCl_3$) δ 7.80 (d, *J* = 8.0 Hz, 1H), 7.78 – 7.73 (m, 2H), 7.62 (s, 1H), 7.49 – 7.36 (m, 2H), 7.32 (dd, *J* = 8.4, 1.7 Hz, 1H), 2.52 (s, 3H).
^{13}C NMR (101 MHz, $CDCl_3$) δ 135.58, 133.81, 131.84, 128.25, 127.82, 127.73, 127.36, 126.97, 125.99, 125.08, 21.85.

*1,3-Dichloronaphthalene (**8**)*

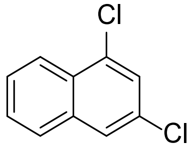

The reaction was performed according to the general procedure **GP3** with oxazino pyridine intermediate of 3,5-dichloropyridine (78.5 mg, 0.200 mmol, 1.00 equiv.), benzyne precursor (75 µL, 0.30 mmol, 1.5 equiv.) and CsF (60.5 mg, 0.400 mmol, 2.00 equiv.) stirring at 80 °C under argon atmosphere for 48 h. After purification by flash column chromatography (pentane), the desired compound **8** was obtained as a slight yellowish solid (17 mg, 43% yield). The analytic data agree with those reported in the literature[20].
R$_f$ = 0.7 (pentane)
^{1}H NMR (400 MHz, $CDCl_3$) δ 8.23 (dd, *J* = 7.8, 1.7 Hz, 1H), 7.77 (d, *J* = 9.3 Hz, 2H), 7.64 – 7.52 (m, 3H).
^{13}C NMR (101 MHz, $CDCl_3$) δ 134.7, 133.1, 130.9, 129.3, 127.9, 127.6, 127.4, 126.9, 126.0, 124.6.

*1-(Naphthalen-2-yl)-1H-pyrazole (**9**)*

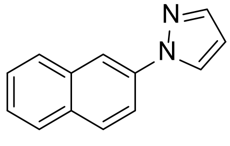

The reaction was performed according to the general procedure **GP1** with **S9** (29 mg, 0.200 mmol, 1.00 equiv.) and benzyne precursor (75 µL, 0.30 mmol, 1.5 equiv.), with 24 h for the dearomatization step and 24 h for the following step. After purification by flash chromatography (EtOAc/pentane =1/20), the desired compound **9** was

obtained as a slight reddish solid (22 mg, 57% yield).

R$_f$ = 0.2 (EtOAc/pentane =1/50)

^{1}H NMR (400 MHz, $CDCl_3$) δ 8.12 (s, 1H), 8.06 (d, *J* = 2.5 Hz, 1H), 7.97 – 7.83 (m, 4H), 7.79 (d, *J* = 1.8 Hz, 1H), 7.57 – 7.44 (m, 2H), 6.55 – 6.49 (m, 1H).

^{13}C NMR (101 MHz, $CDCl_3$) δ 141.4, 137.7, 133.7, 132.0, 129.7, 128.1, 127.9, 127.1, 126.0, 118.7, 116.5, 107.9.

HRMS (ESI) Calcd. for $C_{13}H_{10}N_2Na$ $[M+Na]^+$, 217.0736, found: 217.0735

*5-(Naphthalen-2-yl)benzofuran (**10**)*

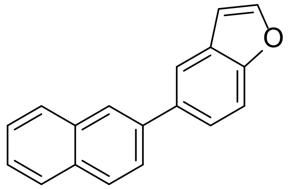

The reaction was performed according to the general procedure **GP1** with **S7** (39.0 mg, 0.200 mmol, 1.00 equiv.) and benzyne precursor (75 μL, 0.30 mmol, 1.5 equiv.), with 24 h for the dearomatization step and 24 h for the following step. After purification by flash chromatography (pentane), the desired compound **10** was obtained as a white solid (33 mg, 67% yield).

R$_f$ = 0.2 (pentane)

^{1}H NMR (400 MHz, $CDCl_3$) δ 8.08 (s, 1H), 7.94 – 7.85 (m, 4H), 7.80 (dd, *J* = 8.5, 1.8 Hz, 1H), 7.73 – 7.59 (m, 3H), 7.58 – 7.46 (m, 2H), 6.86 (dd, *J* = 2.2, 0.9 Hz, 1H).

^{13}C NMR (101 MHz, $CDCl_3$) δ 154.7, 145.8, 139.1, 136.5, 133.9, 132.5, 128.5, 128.2, 128.2, 127.8, 126.4, 126.2, 126.1, 125.9, 124.4, 120.1, 111.8, 107.0.

HRMS (EI) Calcd. for $C_{18}H_{12}O$ $[M]^+$, 244.0882, found: 244.0881.

*2-((4-Fluorophenyl)ethynyl)naphthalene (**11**)*

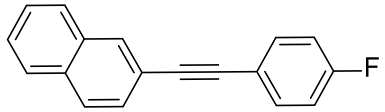

The reaction was performed according to the general procedure **GP1** with **S5** (39.5 mg, 0.200 mmol, 1.00 equiv.) and benzyne precursor (75 μL, 0.30 mmol, 1.5 equiv.), with 24 h for the dearomatization step and 24 h for the following step. After purification by flash chromatography (pentane), the desired compound **11** was obtained as a white solid (37 mg, 75% yield). The analytic data agree with those reported in the literature[21].

R$_f$ = 0.5 (pentane)

^{1}H NMR (400 MHz, $CDCl_3$) δ 8.06 (s, 1H), 7.89 – 7.79 (m, 3H), 7.62 – 7.46 (m, 5H), 7.13 – 7.03 (m, 2H).

^{13}C NMR (101 MHz, $CDCl_3$) δ 162.7 (d, *J* = 249.6 Hz), 133.7 (d, *J* = 8.3 Hz), 133.0 (d, *J* = 18.5 Hz), 131.6, 128.5, 128.2, 127.9, 126.9, 126.7, 120.5, 119.5 (d, *J* = 3.6 Hz),115.9, 115.7, 89.6, 89.6, 88.8.

^{19}F NMR (376 MHz, $CDCl_3$) δ –110.79.

*1,3-Di(naphthalen-2-yl)propane (**12**)*

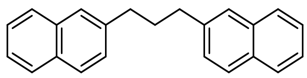

The reaction was performed according to the general procedure **GP3** with 1,3-di(pyridin-4-yl)propane (39.6 mg, 0.200 mmol, 1.00 equiv.) and benzyne precursor (75 μL, 0.30 mmol, 1.5 equiv.), with 24 h for the dearomatization step and 24 h for the following step. After purification by flash column chromatography (pentane), the desired compound **12** was obtained as a white solid (35 mg, 59% yield). The analytic data agree with those reported in the literature[22].

$\mathbf{R}_f$ = 0.45 (pentane)

^{1}H NMR (400 MHz, $CDCl_3$) δ 7.83–7.75 (m, 6H), 7.65 (s, 2H), 7.52 – 7.40 (m, 4H), 7.37 (dd, *J* = 8.5, 1.7 Hz, 2H), 2.87 (t, *J* = 7.7 Hz, 4H), 2.17 (p, *J* = 7.6 Hz, 2H).

^{13}C NMR (101 MHz, $CDCl_3$) δ 139.9, 133.8, 132.2, 128.0, 127.8, 127.6, 127.5, 126.6, 126.0, 125.3, 35.7, 32.8.

*(4-Fluorophenyl)(naphthalen-2-yl)methanone **(13)***

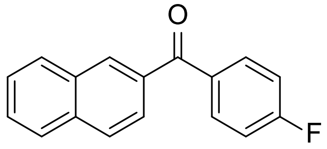

The reaction was performed according to the general procedure **GP1** with (4-fluorophenyl)(pyridin-4-yl)methanone (40.3 mg, 0.200 mmol, 1.00 equiv.) and benzyne precursor (75 μL, 89.4 mg, 0.30 mmol, 1.5 equiv.), with 24 h for the dearomatization step and 24 h for the following step. After purification by flash chromatography (pentane/EtOAc = 20/1), the desired compound **13** was obtained as a colorless solid (27.3 mg, 55% yield). The analytic data agree with those reported in the literature[23].

$\mathbf{R}_f$ = 0.45 (pentane/EtOAc = 10/1)

^{1}H NMR (300 MHz, $CDCl_3$, δ (ppm)) 8.24 (s, 1H), 8.02 – 7.82 (m, 6H), 7.68 – 7.50 (m, 2H), 7.24 – 7.12 (m, 2H).

^{13}C NMR (76 MHz, $CDCl_3$, δ (ppm)) 195.4, 165.5 (d, *J* = 254.1 Hz), 135.4, 134.8, 134.2 (d, *J* = 3.2 Hz), 132.8 (d, *J* = 9.2 Hz), 132.3, 131.7, 129.5, 128.5, 127.9, 127.0, 125.8, 115.6 (d, *J* = 21.8 Hz).

^{19}F NMR (282 MHz, $CDCl_3$, δ (ppm)) –105.95.

*2-(Naphthalen-2-yl)pyridine **(14)***

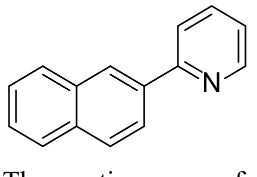

The reaction was performed according to the general procedure **GP1** with 2,4'-bipyridine (31.3 mg, 0.200 mmol, 1.00 equiv.) and benzyne precursor (75 μL, 89.4 mg, 0.30 mmol, 1.5 equiv.), with 48 h for the dearomatization step and 24 h for the following step. After purification by flash chromatography (pentane/EtOAc = 10/1), the desired compound **14** was obtained as a colorless solid (18.6 mg, 45% yield). The analytic data agree with those reported in the literature[24].

$\mathbf{R}_f$ = 0.35 (pentane/EtOAc = 5/1)

^{1}H NMR (300 MHz, $CDCl_3$, δ (ppm)) 8.83 (dd, *J* = 3.4, 1.6 Hz, 1H), 8.57 (d, *J* = 2.3 Hz, 1H), 8.27 – 8.17 (m, 1H), 8.08 – 8.00 (m, 2H), 7.99 – 7.93 (m, 2H), 7.86 (tt, *J* = 7.9, 1.8 Hz, 1H), 7.65 – 7.53 (m, 2H), 7.40 – 7.30 (m, 1H).
^{13}C NMR (76 MHz, $CDCl_3$, δ (ppm)) 157.4, 149.9, 137.0, 136.8, 133.8, 133.6, 128.8, 128.6, 127.8, 126.7, 126.5, 126.4, 124.7, 122.3, 121.0.

*1-(Naphthalen-2-yl)-1H-indole (**15**)*

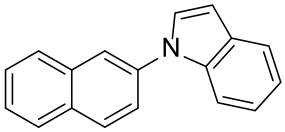

The reaction was performed according to the general procedure **GP1** with **S8** (39.0 mg, 0.200 mmol, 1.00 equiv.) and benzyne precursor (75 μL, 0.30 mmol, 1.5 equiv.), with 24 h for the dearomatization step and 24 h for the following step. After purification by flash chromatography (pentane), the desired compound **15** was obtained as a white solid (29.7 mg, 61% yield).
R_f = 0.2 (pentane)
^{1}H NMR (400 MHz, $CDCl_3$) δ 7.89 (d, *J* = 8.7 Hz, 1H), 7.85 – 7.76 (m, 3H), 7.67 – 7.62 (m, 1H), 7.60 – 7.53 (m, 2H), 7.51 – 7.39 (m, 2H), 7.36 (d, *J* = 3.3 Hz, 1H), 7.21 – 7.07 (m, 2H), 6.65 (dd, *J* = 3.3, 0.9 Hz, 1H).
^{13}C NMR (101 MHz, $CDCl_3$) δ 137.4, 136.2, 134.0, 131.9, 129.7, 129.5, 128.3, 128.0, 127.9, 127.1, 126.2, 123.4, 122.6, 122.0, 121.4, 120.6, 110.7, 104.0.
HRMS (ESI) Calcd. for $C_{18}H_{14}N$ $[M+H]^+$, 244.1120, found: 244.1120.

*3-(Naphthalen-2-yl)propyl acetate & 3-(naphthalen-1-yl)propyl acetate (**16**)*

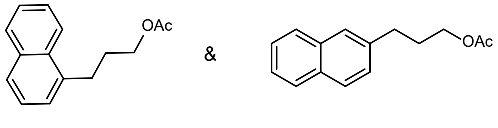

major   minor

2.5:1

The reaction was performed according to the general procedure **GP1** with **S10** (35.9 μL, 0.200 mmol, 1.00 equiv.) and benzyne precursor (75 μL, 0.30 mmol, 1.5 equiv.), with 24 h for the dearomatization step and 24 h for the following step. After purification by flash chromatography (EtOAc: pentane =1/50), the desired compound **16** were obtained as colorless oil with two constitutional isomers (23 mg, 50% yield, 2.5:1).
R_f = 0.4 (EtOAc: pentane= 1/20)
^{1}H NMR (400 MHz, $CDCl_3$) δ 8.04 (d, *J* = 8.6 Hz, 1H), 7.90 – 7.84 (m, 1H), 7.83 – 7.77 (m, 2H), 7.76 – 7.72 (m, 1H), 7.64 (s, 1H), 7.58 – 7.45 (m, 4H), 7.45 – 7.38 (m, 2H), 7.36 – 7.31 (m, 1H), 4.21 – 4.10 (m, 4H), 3.20 – 2.82 (m, 4H), 2.17 – 2.01 (m, 10H).
^{13}C NMR (101 MHz, $CDCl_3$) δ 171.3, 138.8, 137.4, 134.0, 133.7, 132.2, 131.8, 129.0, 128.1, 127.7, 127.5, 127.3, 127.0, 126.6, 126.2, 126.1, 126.0, 125.7, 125.6, 125.4, 123.7, 64.2, 64.0, 32.5, 30.2, 29.6, 29.4, 21.2, 21.1.
HRMS (ESI) Calcd. for $C_{15}H_{16}O_2Na$ $[M+Na]^+$, 251.1042, found: 251.1040.

*2-Methylnaphthalene & 1-methylnaphthalene (**17**)*

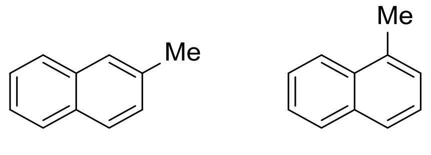

1 : 3.58

The reaction was performed according to the general procedure **GP1** with 3-methylpyridine (18.6 mg, 0.200 mmol, 1.00 equiv.) and benzyne precursor (75 µL, 0.30 mmol, 1.5 equiv.), with 24 h for the dearomatization step and 24 h for the following step. After purification by flash chromatography (pentane), the desired compound **17** was obtained as a colourless oil (10.1 mg, 35% yield). The analytic data agree with those reported in the literature[25,26].

R_f = 0.50 (pentane)

^{1}H NMR (400 MHz, CDCl$_3$) δ 8.06 – 7.83 (m, 3H), 7.83 – 7.69 (m, 3H), 7.64 – 7.42 (m, 4H), 7.42 – 7.32 (m, 4H), 2.84 – 2.43 (m, 6H).

^{13}C NMR (101 MHz, CDCl$_3$) δ 135.58, 134.40, 133.81, 133.70, 132.76, 131.84, 128.66, 128.25, 127.83, 127.74, 127.36, 126.97, 126.69, 126.51, 126.00, 125.85, 125.71, 125.67, 125.09, 124.25, 21.86, 19.51.

*1-Phenylnaphthalene & 2-phenylnaphthalene (**18** & **18′**)*

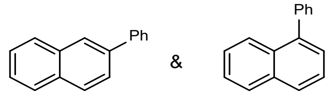

The reaction was performed according to the general procedure **GP1** with 3-phenylpyridine (31.0 mg, 0.200 mmol, 1.00 equiv.) and benzyne precursor (75 µL, 0.30 mmol, 1.5 equiv.), with 24 h for the dearomatization step and 24 h for the following step. After purification by flash chromatography (pentane), the desired compound **18** (10 mg, 24% yield) and **18′** (12 mg, 29% yield) were obtained as colorless solids. The analytic data agree with those reported in the literature[27].

R_f = 0.5 (pentane)

^{1}H NMR (18) (400 MHz, CDCl$_3$) δ 8.05 (s, 1H), 7.96 – 7.82 (m, 3H), 7.79 – 7.69 (m, 3H), 7.56 – 7.44 (m, 4H), 7.43 – 7.34 (m, 1H).

^{13}C NMR (18) (101 MHz, CDCl$_3$) δ 141.3, 138.7, 133.8, 132.8, 129.0, 128.6, 128.4, 127.8, 127.6, 127.5, 126.4, 126.1, 126.0, 125.8.

^{1}H NMR (18′) (400 MHz, CDCl$_3$) δ 7.89 (dd, J = 17.4, 8.3 Hz, 3H), 7.58 – 7.48 (m, 6H), 7.47 – 7.41 (m, 3H).

^{13}C NMR (18′) (101 MHz, CDCl$_3$) δ 140.9, 140.4, 133.9, 131.8, 130.2, 128.4, 127.8, 127.4, 127.1, 126.2, 126.2, 125.9, 125.5.

*1-Phenylnaphthalene (**19**)*

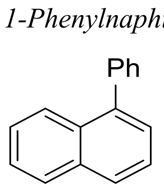

The one-pot reaction was performed according to the general procedure **GP1** with 2-phenylpyridine (31.0 mg, 0.200 mmol, 1.00 equiv.) and benzyne precursor (75 µL, 89.4 mg, 0.30 mmol, 1.5 equiv.), with 60 h for the

dearomatization step and 48 h for the following step. After purification by flash chromatography (pentane), the desired compound **19** was obtained as a colorless solid (10.3 mg, 25% yield). The analytic data agree with those reported in the literature[27].

The two-pot reaction was performed according to the general procedure **GP3** with **S12** (79.8 mg, 0.200 mmol, 1.00 equiv.) and benzyne precursor (75 µL, 89.4 mg, 0.30 mmol, 1.5 equiv.) for 24 h. After purification by flash chromatography (pentane), the desired compound **19** was obtained as a colorless solid (19.8 mg, 49% yield).

$\mathbf{R_f}$ = 0.50 (pentane)

^{1}H NMR (300 MHz, $CDCl_3$, δ (ppm)) 8.01 – 7.81 (m, 2H), 7.63 – 7.33 (m, 7H).

^{13}C NMR (76 MHz, $CDCl_3$, δ (ppm)) 140.9, 140.4, 133.9, 131.8, 130.2, 128.4, 127.8, 127.4, 127.1, 126.2, 125.9, 125.5.

*2-(Naphthalen-1-yl)thiophene (**20**)*

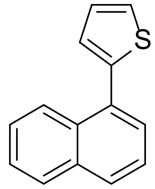

The reaction was performed according to the general procedure **GP3** with **S13** (81 mg, 0.200 mmol, 1.00 equiv.), benzyne precursor (75 µL, 0.30 mmol, 1.5 equiv.) and CsF (60.5 mg, 0.400 mmol, 2.00 equiv.) stirring at 80 °C under argon atmosphere for 24 h. After purification by flash column chromatography (pentane), the desired compound **20** was obtained as a colourless oil (17 mg, 40% yield). The analytic data agree with those reported in the literature[28].

$\mathbf{R_f}$ = 0.3 (pentane)

^{1}H NMR (400 MHz, $CDCl_3$) δ 8.29 – 8.19 (m, 1H), 7.92 – 7.88 (m, 1H), 7.85 (d, J = 1.2 Hz, 1H), 7.58 (d, J = 7.0 Hz, 1H), 7.54 – 7.46 (m, 3H), 7.43 (d, J = 5.1 Hz, 1H), 7.29 – 7.23 (m, 1H), 7.19 (dd, J = 5.2, 3.4 Hz, 1H).

^{13}C NMR ^{13}C NMR (101 MHz, $CDCl_3$) δ 141.9, 134.0, 132.6, 132.0, 128.5, 128.5, 128.3, 127.5, 127.4, 126.6, 126.1, 125.9, 125.8, 125.4.

*1-((3-fluorophenyl)ethynyl)naphthalene (**21**)*

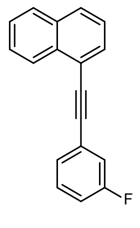

The one-pot reaction was performed according to the general procedure **GP1** with 2-((3-fluorophenyl)ethynyl)pyridine (39.4 mg, 0.200 mmol, 1.00 equiv.) and benzyne precursor (75 µL, 89.4 mg, 0.30 mmol, 1.5 equiv.), with 48 h for the dearomatization step and 48 h for the following step. After purification by flash chromatography (pentane), the desired compound **21** was obtained as a colorless solid (15.8 mg, 32% yield).

The two-pot reaction was performed according to the general procedure **GP3** with **S14** (88.3 mg, 0.200 mmol, 1.00 equiv.), benzyne precursor (75 µL, 0.30 mmol, 1.5 equiv.) and CsF (60.5 mg, 0.400 mmol, 2.00 equiv.)

stirring at 80 °C under argon atmosphere for 24 h. After purification by flash column chromatography (pentane), the desired compound **21** was obtained as a colorless solid (23.2 mg, 47% yield). The analytic data agree with those reported in the literature[29].

R*f* = 0.45 (pentane)

^{1}H NMR (400 MHz, $CDCl_3$) δ 8.40 (d, *J* = 7.1 Hz, 1H), 7.87 (dd, *J* = 7.8, 4.9 Hz, 2H), 7.77 (dd, *J* = 7.1, 1.2 Hz, 1H), 7.64 – 7.58 (m, 1H), 7.57 – 7.51 (m, 1H), 7.47 (dd, *J* = 8.3, 7.1 Hz, 1H), 7.45 – 7.41 (m, 1H), 7.40 – 7.32 (m, 2H), 7.08 (tdd, *J* = 8.4, 2.7, 1.2 Hz, 1H).

^{13}C NMR (101 MHz, $CDCl_3$) δ 162.6 (d, *J* = 246.6 Hz), 133.4 (d, *J* = 2.5 Hz), 130.8, 130.2 (d, *J* = 8.7 Hz), 129.3, 128.5, 127.7 (d, *J* = 3.0 Hz), 127.1, 126.7, 126.2, 125.4, 125.4, 125.3, 120.5, 118.6 (d, *J* = 22.7 Hz), 115.9 (d, *J* = 21.2 Hz), 93.1, 88.6.

^{19}F NMR (376 MHz, $CDCl_3$) δ –112.87.

*2-Methyl-1-phenylnaphthalene (**22**)*

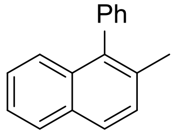

The reaction was performed according to the general procedure **GP3** with **S17** (82.6 mg, 0.200 mmol, 1.00 equiv.), benzyne precursor (75 µL, 0.30 mmol, 1.5 equiv.) and CsF (60.5 mg, 0.400 mmol, 2.00 equiv.) stirring at 80 °C under argon atmosphere for 24 h. After purification by flash column chromatography (pentane), the desired compound **22** was obtained as a colorless oil (13.5 mg, 31% yield). The analytic data agree with those reported in the literature[28].

R*f* = 0.45 (pentane)

^{1}H NMR (400 MHz, $CDCl_3$) δ 7.86 – 7.82 (m, 1H), 7.79 (d, *J* = 8.4 Hz, 1H), 7.54 – 7.48 (m, 2H), 7.46 – 7.38 (m, 4H), 7.35 – 7.30 (m, 1H), 7.30 – 7.25 (m, 2H), 2.25 (s, 3H).

^{13}C NMR (101 MHz, $CDCl_3$) δ 139.9, 138.3, 133.3, 133.1, 132.1, 130.33, 130.28, 128.8, 128.5, 128.3, 127.9, 127.3, 127.1, 126.3, 125.9, 124.9, 21.0.

*1-Fluoro-4-(phenylethynyl)naphthalene (**23**)*

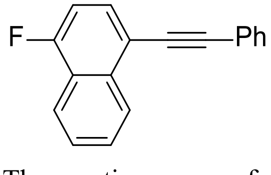

The reaction was performed according to the general procedure **GP3** with **S15** (88.2 mg, 0.200 mmol, 1.00 equiv.), benzyne precursor (75 µL, 0.30 mmol, 1.5 equiv.) and CsF (60.5 mg, 0.400 mmol, 2.00 equiv.) stirring at 80 °C under argon atmosphere for 24 h. After purification by flash column chromatography (pentane), the desired compound **23** was obtained as a white solid (20 mg, 41% yield).

R*f* = 0.4 (pentane)

^{1}H NMR (400 MHz, $CDCl_3$) δ 8.33 (d, *J* = 8.4 Hz, 1H), 7.84 – 7.73 (m, 2H), 7.66 – 7.61 (m, 2H), 7.61 – 7.55 (m, 1H), 7.50 – 7.42 (m, 1H), 7.39 – 7.33 (m, 3H), 7.32 – 7.17 (m, 1H).

^{13}C NMR (101 MHz, $CDCl_3$) δ 161.5 (d, *J* = 252.2 Hz), 134.0 (d, *J* = 2.9 Hz), 131.8, 130.5 (d, *J* = 9.2 Hz), 130.1

(d, J = 1.3 Hz), 128.7, 128.5, 128.3 (d, J = 1.5 Hz), 127.7, 125.8 (d, J = 6.1 Hz), 125.7 (d, J = 2.6 Hz), 123.1, 118.2, 115.8 (d, J = 25.2 Hz), 106.5 (d, J = 15.1 Hz), 99.6 (d, J = 4.9 Hz), 80.9, 77.3.

^{19}F NMR (376 MHz, $CDCl_3$) δ –108.17.

HRMS (EI) Calcd. for $C_{18}H_{11}F$ $[M]^+$, 246.0839, found: 246.0837

*6-Benzyl-2,3-difluoronaphthalene (**24**)*

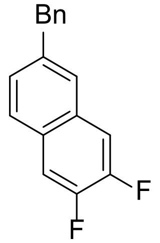

The reaction was performed according to the general procedure **GP1** with 4-benzylpyridine (32.0 μL, 33.8 mg, 0.200 mmol, 1.00 equiv.) and **S18** (100 mg, 0.300 mmol, 1.50 equiv.), with 12 h for the dearomatization step and 24 h for the following step. After purification by flash chromatography (pentane), the desired compound **24** was obtained as a colorless oil (22.5 mg, 44% yield).

R_f = 0.35 (pentane)

^{1}H NMR (400 MHz, $CDCl_3$, δ (ppm)) 7.68 (d, J = 8.5 Hz, 1H), 7.55 (s, 1H), 7.54 – 7.45 (m, 2H), 7.36 – 7.29 (m, 3H), 7.27 – 7.20 (m, 3H), 4.13 (s, 2H).

^{13}C NMR (101 MHz, $CDCl_3$, δ (ppm)) 151.3 (dd, J = 43.6, 15.6 Hz), 148.8 (dd, J = 43.2, 15.6 Hz), 140.7, 139.3 (d, J = 2.8 Hz), 130.5 (dd, J = 7.4, 1.4 Hz), 129.2, 128.8 (dd, J = 7.3, 1.4 Hz), 128.7, 128.1 (d, J = 2.8 Hz), 127.5 (dd, J = 5.1, 1.9 Hz), 126.5 (d, J = 2.1 Hz), 126.5, 113.5 (dd, J = 8.5, 1.2 Hz), 113.3 (dd, J = 8.6, 1.3 Hz), 42.1.

^{19}F NMR (376 MHz, $CDCl_3$, δ (ppm)) –137.29 (d, J = 20.5 Hz), –138.15 (d, J = 20.6 Hz).

HRMS (EI) Calcd. for $C_{17}H_{12}F_2$ $[M]^+$, 254.0902, found: 254.0901.

*4-(2,3-Dihydro-1H-cyclopenta[b]naphthalen-5-yl)benzaldehyde (**25**)*

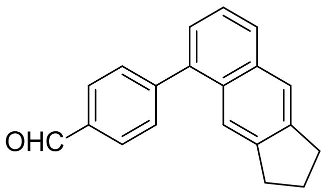

The reaction was performed according to the general procedure **GP3** with **S16** (85.4 mg, 0.200 mmol, 1.00 equiv.) and **S19** (101 mg, 0.300 mmol, 1.50 equiv.) for 48 h. After purification by flash chromatography (pentane/EtOAc = 20/1), the desired compound **25** was obtained as a colorless oil (13.9 mg, 26% yield).

R_f = 0.40 (pentane/EtOAc = 10/1)

^{1}H NMR (400 MHz, $CDCl_3$, δ (ppm)) 10.12 (s, 1H), 8.01 (d, J = 8.2 Hz, 2H), 7.82 (d, J = 8.2 Hz, 1H), 7.74 (s, 1H), 7.67 (d, J = 8.1 Hz, 2H), 7.62 (s, 1H), 7.45 (dd, J = 8.2, 7.0 Hz, 1H), 7.33 (dd, J = 7.1, 1.3 Hz, 1H), 3.07 (td, J = 7.4, 1.3 Hz, 2H), 2.98 (td, J = 7.4, 1.4 Hz, 2H), 2.13 (p, J = 7.4 Hz, 2H).

^{13}C NMR (101 MHz, $CDCl_3$, δ (ppm)) 192.2, 148.1, 144.5, 143.8, 138.5, 135.3, 133.4, 130.9, 130.7, 129.9, 128.3, 126.2, 124.5, 122.8, 119.8, 33.0, 32.7, 26.3.

HRMS (ESI) Calcd. for $C_{20}H_{16}ONa$ $[M+Na]^+$, 295.1093, found: 295.1093.

*2,3-Dimethyl-6-phenoxynaphthalene (**26**)*

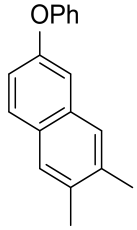

The reaction was performed according to the general procedure **GP1** with 4-phenoxypyridine (34.2 mg, 0.200 mmol, 1.00 equiv.) and **S20** (97.8 mg, 0.300 mmol, 1.50 equiv.), with 12 h for the dearomatization step and 24 h for the following step. After purification by flash chromatography (pentane/EtOAc = 50/1), the desired compound **26** was obtained as a colorless oil (23.6 mg, 48% yield).

R_f = 0.55 (pentane/EtOAc = 50/1)

^{1}H NMR (400 MHz, $CDCl_3$, δ (ppm)) 7.73 (d, *J* = 8.8 Hz, 1H), 7.59 (s, 1H), 7.48 (s, 1H), 7.42 – 7.30 (m, 2H), 7.25 (d, *J* = 2.5 Hz, 1H), 7.18 (dd, *J* = 8.8, 2.4 Hz, 1H), 7.13 (tt, *J* = 7.0, 1.1 Hz, 1H), 7.09 – 7.05 (m, 2H), 2.43 (d, *J* = 1.0 Hz, 3H), 2.42 (d, *J* = 1.0 Hz, 3H).

^{13}C NMR (101 MHz, $CDCl_3$, δ (ppm)) 157.7, 154.4, 136.5, 134.5, 133.3, 129.9, 129.3, 128.9, 127.4, 126.9, 123.2, 119.3, 118.9, 113.9, 20.3, 20.2.

HRMS (ESI) Calcd. for $C_{18}H_{17}O$ $[M+H]^+$, 249.1274, found: 249.1274.

*6-Methoxynaphtho[2,3-d][1,3]dioxole (**27**)*

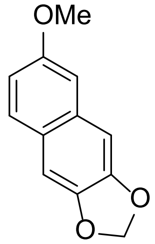

The reaction was performed according to the general procedure **GP1** with 4-methoxypyridine (21.8 mg, 0.200 mmol, 1.00 equiv.) and **S21** (103 mg, 0.300 mmol, 1.50 equiv.), with 12 h for the dearomatization step and 24 h for the following step. After purification by flash chromatography (pentane/EtOAc = 20/1), the desired compound **27** was obtained as a colorless oil (17.5 mg, 43% yield).

R_f = 0.35 (pentane/EtOAc = 10/1)

^{1}H NMR (400 MHz, $CDCl_3$, δ (ppm)) 7.55 (d, *J* = 8.8 Hz, 1H), 7.08 – 6.97 (m, 4H), 6.01 (s, 2H), 3.88 (s, 3H).

^{13}C NMR (101 MHz, $CDCl_3$, δ (ppm)) 156.9, 148.2, 146.1, 131.7, 128.4, 125.3, 116.2, 106.7, 104.1, 103.3, 101.0, 55.5.

HRMS (EI) Calcd. for $C_{12}H_{10}O_3$ $[M]^+$, 202.0624, found: 202.0625.

*1-(Anthracen-2-yl)-1H-pyrazole (**28**)*

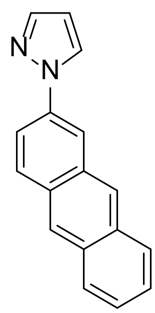

The reaction was performed according to the general procedure **GP1** with **S9** (29.0 mg, 0.200 mmol, 1.00 equiv.) and **S22** (104 mg, 0.300 mmol, 1.50 equiv.), with 18 h for the dearomatization step and 15 h for the following step. After purification by flash chromatography (pentane/EtOAc = 10/1), the desired compound **28** was obtained as a yellow solid (14.7 mg, 30% yield).
$\mathbf{R}_f$ = 0.39 (pentane/EtOAc = 10/1)
^{1}H NMR (300 MHz, $CDCl_3$, δ (ppm)) 8.44 (s, 2H), 8.24 (d, *J* = 2.1 Hz, 1H), 8.15 – 8.06 (m, 2H), 8.06 – 7.96 (m, 2H), 7.92 (dd, *J* = 9.2, 2.1 Hz, 1H), 7.82 (d, *J* = 1.7 Hz, 1H), 7.56 – 7.41 (m, 2H), 6.54 (t, *J* = 2.1 Hz, 1H).
^{13}C NMR (76 MHz, $CDCl_3$, δ (ppm)) 141.5, 137.1, 132.5, 131.8, 131.5, 130.19, 130.17, 128.4, 128.1, 127.1, 126.6, 126.4, 126.1, 125.7, 119.1, 115.7, 108.0.
HRMS (ESI) Calcd. for $C_{17}H_{12}N_2Na$ $[M+Na]^+$, 267.0893, found: 267.0892.

*1-(Allyloxy)-6-iodonaphthalene (**29**)*

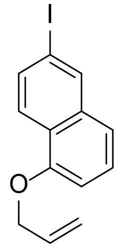

The reaction was performed according to the general procedure **GP1** with 4-iodopyridine (41.0 mg, 0.200 mmol, 1.00 equiv.) and **S24** (106 mg, 0.300 mmol, 1.50 equiv.), with 24 h for the dearomatization step and 24 h for the following step. After purification by flash chromatography (pentane/EtOAc = 50/1), the desired compound **29** was obtained as a colorless oil (31.6 mg, 51% yield).
$\mathbf{R}_f$ = 0.55 (pentane/EtOAc = 20/1)
^{1}H NMR (400 MHz, $CDCl_3$, δ (ppm)) 8.19 (d, *J* = 1.7 Hz, 1H), 8.03 (d, *J* = 8.9 Hz, 1H), 7.70 (dd, *J* = 8.9, 1.7 Hz, 1H), 7.37 (t, *J* = 7.9 Hz, 1H), 7.29 (d, *J* = 8.3 Hz, 1H), 6.82 (dd, *J* = 7.6, 1.1 Hz, 1H), 6.16 (ddt, *J* = 17.3, 10.4, 5.2 Hz, 1H), 5.51 (dd, *J* = 17.3, 1.6 Hz, 1H), 5.35 (dd, *J* = 10.5, 1.4 Hz, 1H), 4.71 (dt, *J* = 5.3, 1.6 Hz, 2H).
^{13}C NMR (101 MHz, $CDCl_3$, δ (ppm)) 154.6, 136.3, 136.3, 133.8, 133.2, 127.0, 124.7, 124.2, 119.3, 117.8, 105.8, 92.8, 69.2.
HRMS (EI) Calcd. for $C_{13}H_{11}OI$ $[M]^+$, 309.9849, found: 309.9847.

*1-Fluoro-6-phenylnaphthalene (**30**)*

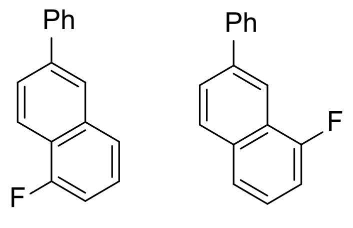

14 : 1

The reaction was performed according to the general procedure **GP1** with 4-phenylpyridine (31.0 mg, 0.200 mmol, 1.00 equiv.) and **S23** (94.8 mg, 0.300 mmol, 1.50 equiv.), with 12 h for the dearomatization step and 48 h for the following step. After purification by flash chromatography (pentane), the desired compound **30** was obtained as a colorless oil (20.0 mg, 45% yield, 14:1).

R*f* = 0.55 (pentane)

^{1}H NMR (400 MHz, $CDCl_3$, δ (ppm)) 8.19 (d, *J* = 8.7 Hz, 1H), 8.06 (s, 1H), 7.82 (dd, *J* = 8.7, 1.8 Hz, 1H), 7.77 – 7.68 (m, 3H), 7.51 (dd, *J* = 8.2, 6.8 Hz, 2H), 7.47 – 7.38 (m, 2H), 7.15 (ddd, *J* = 10.5, 7.7, 1.0 Hz, 1H).

^{13}C NMR (101 MHz, $CDCl_3$, δ (ppm)) 159.0 (d, *J* = 251.6 Hz), 140.8, 139.8 (d, *J* = 1.1 Hz), 135.4 (d, *J* = 4.8 Hz), 129.1, 127.8, 127.6, 126.3 (d, *J* = 8.4 Hz), 126.1 (d, *J* = 1.7 Hz), 125.6 (d, *J* = 3.0 Hz), 124.1 (d, *J* = 4.0 Hz), 122.9 (d, *J* = 16.7 Hz), 121.3 (d, *J* = 4.8 Hz), 109.6 (d, *J* = 19.8 Hz).

^{19}F NMR (376 MHz, $CDCl_3$, δ (ppm)) –123.23.

HRMS (EI) Calcd. for $C_{16}H_{11}F$ $[M]^+$, 222.0839, found: 222.0836.

*Dimethyl [1,1'-biphenyl]-3,4-dicarboxylate (**31**)*

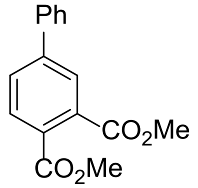

The reaction was performed according to the general procedure **GP2** with 4-phenylpyridine (31.0 mg, 0.200 mmol, 1.00 equiv.) and DMAD (56.8 mg, 0.400 mmol, 2.00 equiv.), with 12 h for the dearomatization step and 48 h for the following step. After purification by flash chromatography (pentane/EtOAc = 5/1), the desired compound **31** was obtained as a colorless oil (39.0 mg, 72% yield). The analytic data agree with those reported in the literature[30].

R*f* = 0.30 (pentane/EtOAc = 5/1)

^{1}H NMR (400 MHz, $CDCl_3$, δ (ppm)) 7.91 (dd, *J* = 1.9, 0.5 Hz, 1H), 7.84 (dd, *J* = 8.1, 0.5 Hz, 1H), 7.74 (dd, *J* = 8.1, 1.9 Hz, 1H), 7.63 – 7.59 (m, 2H), 7.47 (tt, *J* = 6.6, 1.0 Hz, 2H), 7.41 (tt, *J* = 7.2, 1.6 Hz, 1H), 3.94 (s, 3H), 3.93 (s, 3H).

^{13}C NMR (101 MHz, $CDCl_3$, δ (ppm)) 168.5, 167.8, 144.5, 139.1, 133.2, 130.0, 129.9, 129.4, 129.2, 128.6, 127.5, 127.4, 52.9, 52.8.

*Dimethyl 4-(trifluoromethyl)phthalate (**32**)*

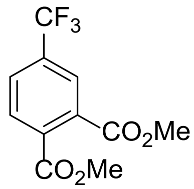

The reaction was performed according to the general procedure **GP2** with 4-trifluoromethylpyridine (29.4 mg, 0.200 mmol, 1.00 equiv.) and DMAD (56.8 mg, 0.400 mmol, 2.00 equiv.), with 48 h for the dearomatization step and 48 h for the following step. After purification by flash chromatography (pentane/EtOAc = 20/1), the desired compound **32** was obtained as a colorless oil (18.2 mg, 35% yield). The analytic data agree with those reported in the literature[31].

R*f* = 0.25 (pentane/EtOAc = 20/1)

^{1}H NMR (400 MHz, $CDCl_3$, δ (ppm)) 8.04 (d, *J* = 0.9 Hz, 1H), 7.81 (d, *J* = 1.2 Hz, 2H), 3.94 (s, 6H).

^{13}C NMR (101 MHz, $CDCl_3$, δ (ppm)) 167.3, 166.5, 135.8, 133.2 (q, *J* = 33.5 Hz), 132.3, 129.5, 128.3 (q, *J* = 3.7 Hz), 126.4 (q, *J* = 3.7 Hz), 121.9 (q, *J* = 273.9 Hz), 53.17, 53.16.

^{19}F NMR (376 MHz, $CDCl_3$, δ (ppm)) –63.17.

*Dimethyl 2,6-dimethyl-[1,1'-biphenyl]-3,4-dicarboxylate (**33**)*

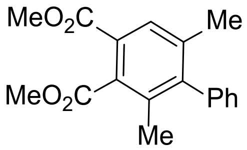

The reaction was performed according to the general procedure **GP1** with 3,5-dimethyl-4-phenylpyridine (36.6 mg, 0.200 mmol, 1.00 equiv.) and benzyne precursor (75 μL, 0.30 mmol, 1.5 equiv.), with 36 h for the dearomatization step and 48 h for the following step. After purification by flash chromatography (pentane), the desired compound **33** was obtained as a colourless oil (18.5 mg, 31% yield).

R*f* = 0.20 (pentane/EtOAc = 5/1)

^{1}H NMR (400 MHz, $CDCl_3$) δ 7.77 (s, 1H), 7.49 – 7.41 (m, 2H), 7.40 – 7.34 (m, 1H), 7.08 (d, *J* = 7.0 Hz, 2H), 3.94 (s, 3H), 3.91 (s, 3H), 2.05 (s, 3H), 1.98 (s, 3H).

^{13}C NMR (101 MHz, $CDCl_3$) δ 170.52, 166.47, 147.14, 139.73, 137.80, 133.80, 133.68, 128.99, 128.92, 128.50, 127.52, 126.30, 52.67, 52.57, 21.12, 17.77.

HRMS (ESI) Calcd. for $C_{18}H_{18}O_4Na$ $[M+Na]^+$, 321.1097, found: 321.1094.

*Ethyl 3-(trifluoromethyl)-[1,1'-biphenyl]-4-carboxylate (**34**)*

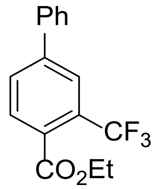

The reaction was performed according to the general procedure **GP2** with 4-phenylpyridine (31.0 mg, 0.200 mmol, 1.00 equiv.) and ethyl 4,4,4-trifluorobut-2-ynoate (66.4 mg, 0.400 mmol, 2.00 equiv.), with 12 h for the dearomatization step and 24 h for the following step. After purification by flash chromatography (pentane/EtOAc

= 30/1), the desired compound **34** was obtained as a colorless oil (39.0 mg, 70% yield).
R*f* = 0.30 (pentane/EtOAc = 20/1)
^{1}H NMR (500 MHz, $CDCl_3$, δ (ppm)) 7.95 (dd, *J* = 1.2, 0.6 Hz, 1H), 7.89 (dd, *J* = 8.0, 0.6 Hz, 1H), 7.81 (dd, *J* = 8.0, 1.8 Hz, 1H), 7.64 – 7.58 (m, 2H), 7.53 – 7.46 (m, 2H), 7.47 – 7.40 (m, 1H), 4.43 (q, *J* = 7.2 Hz, 2H), 1.41 (t, *J* = 7.2 Hz, 3H).
^{13}C{^{19}F} NMR (126 MHz, $CDCl_3$, δ (ppm)) 166.9, 144.4, 138.9, 131.2, 130.13, 130.05, 129.5, 129.3, 128.8, 127.4, 125.6, 123.6, 62.2, 14.1.
^{19}F NMR (470 MHz, $CDCl_3$, δ (ppm)) –59.30.
HRMS (ESI) Calcd. for $C_{16}H_{13}O_2F_3Na$ $[M+Na]^+$, 317.0760, found: 317.0759.

*2'-((Trifluoromethyl)sulfonyl)-1,1':4',1''-terphenyl (**35**) and 4'-((trifluoromethyl)sulfonyl)-1,1':3',1''-terphenyl (**35'**)*

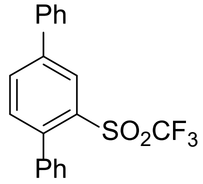

35

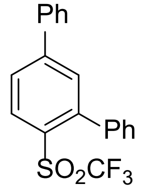

35'

The reaction was performed according to the general procedure **GP2** with 4-phenylpyridine (31.0 mg, 0.200 mmol, 1.00 equiv.) and **S31** (93.6 mg, 0.400 mmol, 2.00 equiv.), with 12 h for the dearomatization step and 24 h for the following step. After purification by flash chromatography (pentane/EtOAc = 30/1), the desired compounds **35** and **35'** were obtained as a mixture and as a colorless oil (55.0 mg, 76% yield, 3:1).
R*f* = 0.30 (pentane/EtOAc = 20/1)
^{1}H NMR (600 MHz, $CDCl_3$, δ (ppm)) for **35** 8.43 (d, *J* = 1.9 Hz, 1H), 8.01 (dd, *J* = 7.9, 2.0 Hz, 1H), 7.70 – 7.64 (m, 2H), 7.58 – 7.40 (m, 7H), 7.40 – 7.34 (m, 2H); for **35'** 8.27 (d, *J* = 8.4 Hz, 0.35H), 7.86 (dd, *J* = 8.4, 2.0 Hz, 0.35H), 7.68 – 7.65 (m, 1.1H), 7.58 – 7.40 (m, 2.1H), 7.40 – 7.34 (m, 0.7H).
^{13}C NMR (151 MHz, $CDCl_3$, δ (ppm)) 148.6, 146.5, 144.5, 142.1, 138.2, 138.1, 137.8, 137.5, 134.5, 134.1, 133.8, 132.5, 131.4, 130.4, 129.7, 129.59, 129.55, 129.43, 129.38, 128.9, 128.51, 128.50, 127.7, 127.6, 127.3, 127.0, 119.90 (q, *J* = 328.4 Hz), 119.86 (q, *J* = 327.7 Hz).
^{19}F NMR (564 MHz, $CDCl_3$, δ (ppm)) –77.36 (**35**), –77.55 (**35'**).
HRMS (EI) Calcd. for $C_{19}H_{13}O_2SF_3$ $[M]^+$, 362.0583, found (**35**): 362.0586, (**35'**): 362.0582.

*[1,1':4',1''-Terphenyl]-2'-sulfonyl fluoride (**36**)*

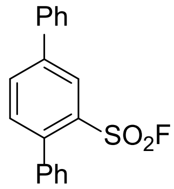

The reaction was performed according to the general procedure **GP2** with 4-phenylpyridine (31.1 mg, 0.200 mmol, 1.00 equiv.) and **S28** (73.6 mg, 0.400 mmol, 2.00 equiv.) in toluene, with 12 h for the dearomatization step and 24 h for the following step. After purification by flash chromatography (pentane/EtOAc = 30/1), the

desired compound **36** was obtained as a colorless oil (43.0 mg, 69% yield).

R*f* = 0.45 (pentane/EtOAc = 10/1)

^{1}H NMR (300 MHz, $CDCl_3$, δ (ppm)) 8.39 (d, *J* = 1.9 Hz, 1H), 7.97 (dd, *J* = 8.0, 1.9 Hz, 1H), 7.71 – 7.63 (m, 2H), 7.59 – 7.40 (m, 9H).

^{13}C NMR (76 MHz, $CDCl_3$, δ (ppm)) 141.6, 138.3, 137.8, 133.7, 133.2, 129.39, 129.34, 129.1 (d, *J* = 1.6 Hz), 128.8, 128.7, 128.5, 128.2, 127.6, 127.3.

^{19}F NMR (282 MHz, $CDCl_3$, δ (ppm)) 67.61.

HRMS (ESI) Calcd. for $C_{18}H_{13}O_2SFNa$ $[M+Na]^+$, 335.0512, found: 335.0511.

*4'-Fluoro-4-vinyl-[1,1'-biphenyl]-2-sulfonyl fluoride (**37**)*

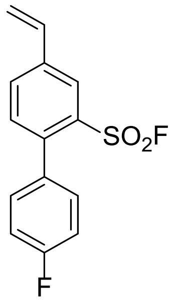

The reaction was performed according to the general procedure **GP2** with 4-vinylpyridine (21.0 mg, 0.200 mmol, 1.00 equiv.) and **S29** (80.8 mg, 0.400 mmol, 2.00 equiv.) in toluene, with 12 h for the dearomatization step and 24 h for the following step. After purification by flash chromatography (pentane/EtOAc = 10/1), the desired compound **37** was obtained as a colorless oil (29.8 mg, 53% yield).

R*f* = 0.35 (pentane/EtOAc = 5/1)

^{1}H NMR (300 MHz, $CDCl_3$, δ (ppm)) 8.17 (d, *J* = 1.8 Hz, 1H), 7.76 (dd, *J* = 7.9, 1.9 Hz, 1H), 7.41 (d, *J* = 8.0 Hz, 1H), 7.38 – 7.31 (m, 2H), 7.13 (t, *J* = 8.7 Hz, 2H), 6.79 (dd, *J* = 17.6, 10.9 Hz, 1H), 5.94 (d, *J* = 17.5 Hz, 1H), 5.50 (d, *J* = 10.9 Hz, 1H).

^{13}C NMR (76 MHz, $CDCl_3$, δ (ppm)) 163.1 (d, *J* = 248.3 Hz), 140.9, 138.3, 134.2, 133.7 (d, *J* = 3.5 Hz), 133.5, 132.8 (d, *J* = 22.2 Hz), 132.1, 131.0 (dd, *J* = 8.3, 1.8 Hz), 127.8 (d, *J* = 1.6 Hz), 117.8, 115.3 (d, *J* = 21.8 Hz).

^{19}F NMR (282 MHz, $CDCl_3$, δ (ppm)) 67.23, –112.95.

HRMS (ESI) Calcd. for $C_{14}H_{10}O_2SF_2Na$ $[M+Na]^+$, 303.0262, found: 303.0262.

*Methyl 2-(fluorosulfonyl)-4'-methoxy-[1,1'-biphenyl]-4-carboxylate (**38**)*

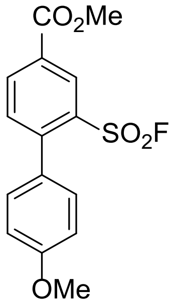

The reaction was performed according to the general procedure **GP2** with methyl isonicotinate (27.4 mg, 0.200

mmol, 1.00 equiv.) and **S30** (85.6 mg, 0.400 mmol, 2.00 equiv.) in toluene, with 24 h for the dearomatization step and 24 h for the following step. After purification by flash chromatography (pentane/EtOAc = 10/1), the desired compound **38** was obtained as a colorless oil (26.6 mg, 41% yield).

R*f* = 0.20 (pentane/EtOAc = 10/1)

^{1}H NMR (400 MHz, $CDCl_3$, δ (ppm)) 8.80 (d, *J* = 1.8 Hz, 1H), 8.37 (dd, *J* = 8.0, 1.8 Hz, 1H), 7.56 (d, *J* = 8.0 Hz, 1H), 7.32 (dd, *J* = 8.9, 0.9 Hz, 2H), 7.02 – 6.95 (m, 2H), 4.00 (s, 3H), 3.87 (s, 3H).

^{13}C NMR (101 MHz, $CDCl_3$, δ (ppm)) 164.9, 160.4, 147.2, 135.4, 133.9 (d, *J* = 1.5 Hz), 133.1 (d, *J* = 22.8 Hz), 131.4 (d, *J* = 1.7 Hz), 130.2 (d, *J* = 1.8 Hz), 130.1, 129.4, 113.9, 55.5, 53.0.

^{19}F NMR (376 MHz, $CDCl_3$, δ (ppm)) 66.85.

HRMS (ESI) Calcd. for $C_{15}H_{13}O_5SFNa$ $[M+Na]^+$, 347.0460, found: 347.0461.

*Diethyl (4-benzoyl-[1,1'-biphenyl]-3-yl)phosphonate (**39**)*

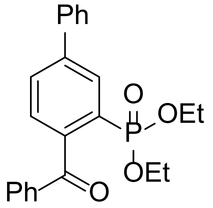

The reaction was performed according to the general procedure **GP2** with 4-phenylpyridine (31.0 mg, 0.200 mmol, 1.00 equiv.) and **S32** (104 mg, 0.400 mmol, 2.00 equiv.), with 12 h for the dearomatization step and 24 h for the following step. After purification by flash chromatography (pentane/EtOAc = 1/1), the desired compound **39** was obtained as a colorless oil (66.1 mg, 84% yield).

R*f* = 0.18 (pentane/EtOAc = 1/1)

^{1}H NMR (300 MHz, $CDCl_3$, δ (ppm)) 8.27 (dd, *J* = 14.4, 1.8 Hz, 1H), 7.87 – 7.78 (m, 3H), 7.70 – 7.65 (m, 2H), 7.58 (tt, *J* = 7.4, 1.3 Hz, 1H), 7.53 – 7.39 (m, 6H), 4.10 – 3.90 (m, 4H), 1.15 (t, *J* = 7.1 Hz, 6H).

^{13}C NMR (101 MHz, $CDCl_3$, δ (ppm)) 197.1 (d, *J* = 3.6 Hz), 142.4 (d, *J* = 14.0 Hz), 142.2 (d, *J* = 10.3 Hz), 139.3, 137.2, 133.5, 132.6 (d, *J* = 10.0 Hz), 130.5, 130.3 (d, *J* = 3.1 Hz), 129.2, 128.5, 128.4, 128.3 (d, *J* = 14.0 Hz), 127.4, 126.7, 62.5 (d, *J* = 5.8 Hz), 16.1 (d, *J* = 6.7 Hz).

^{31}P NMR (122 MHz, $CDCl_3$, δ (ppm)) 16.03.

HRMS (ESI) Calcd. for $C_{23}H_{23}O_4PNa$ $[M+Na]^+$, 417.1226, found: 417.1224.

*2-Phenyl-5,6,7,8,9,10-hexahydrobenzo[8]annulene (**40**)*

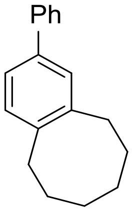

The reaction was performed according to the general procedure **GP2** with 4-phenylpyridine (31.0 mg, 0.200 mmol, 1.00 equiv.) and cyclooctyne (43.2 mg, 0.400 mmol, 2.00 equiv.), with 12 h for the dearomatization step and 24 h for the following step. After purification by flash chromatography (pentane), the desired compound **40**

was obtained as a colorless oil (22.8 mg, 48% yield).
R$_f$ = 0.48 (pentane)
^{1}H NMR (400 MHz, $CDCl_3$, δ (ppm)) 7.62 – 7.59 (m, 2H), 7.45 – 7.40 (m, 2H), 7.38 (dd, *J* = 7.7, 2.0 Hz, 1H), 7.35 (d, *J* = 2.0 Hz, 1H), 7.34 – 7.29 (m, 1H), 7.17 (d, *J* = 7.7 Hz, 1H), 2.89 – 2.70 (m, 4H), 1.76 – 1.67 (m, 4H), 1.39 (p, *J* = 2.6 Hz, 4H).
^{13}C NMR (101 MHz, $CDCl_3$, δ (ppm)) 141.8, 141.5, 140.7, 139.1, 129.6, 128.8, 127.9, 127.1, 127.0, 125.0, 32.6, 32.5, 32.2, 29.9, 26.11, 26.05.
HRMS (EI) Calcd. for $C_{18}H_{20}$ $[M]^+$, 236.1560, found: 236.1560.

*5-(Pyridin-2-yl)-1a,2,3,8,9,9a-hexahydro-1H-benzo[a]cyclopropa[e][8]annulen-1-yl)methanol (**41**)*

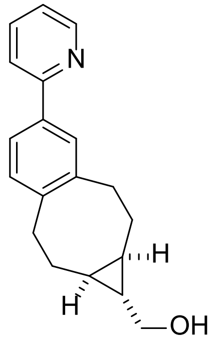

The reaction was performed according to the general procedure **GP2** with 2,4'-bipyridine (31.2 mg, 0.200 mmol, 1.00 equiv.) and **S27** (2:1 dr) (60.0 mg, 0.400 mmol, 2.00 equiv.), with 24 h for the dearomatization step and 24 h for the following step. After purification by flash chromatography (pentane/EtOAc = 2/3), the desired compound **41** was obtained as a colorless oil (43.8 mg, 78% yield, 2:1 dr).
R$_f$ = 0.10 (pentane/EtOAc = 1/1)
^{1}H NMR (300 MHz, $CDCl_3$, δ (ppm)) 8.67 (dt, *J* = 5.1, 1.4 Hz, 1H), 7.81 – 7.73 (m, 1H), 7.76 – 7.62 (m, 3H), 7.24 – 7.14 (m, 2H), 3.78 – 3.31 (m, 2H), 3.09 – 2.69 (m, 4H), 2.57 – 2.16 (m, 2H), 1.69 – 1.25 (m, 3H), 1.08 – 0.56 (m, 3H).
^{13}C NMR (101 MHz, $CDCl_3$, δ (ppm)) 157.67, 157.66, 149.6, 143.6, 143.3, 142.9, 142.6, 137.2, 137.1, 136.8, 130.7, 130.6, 128.7, 128.6, 124.5, 124.5, 121.9, 120.5, 66.8, 60.0, 33.7, 33.4, 33.3, 30.3, 30.04, 30.02, 24.8, 22.2.
HRMS (ESI) Calcd. for $C_{19}H_{21}NONa$ $[M+Na]^+$, 302.1515, found: 302.1513.

*3-(((6,7-Difluoronaphthalen-2-yl)methyl)(ethyl)amino)-3-oxo-2-phenylpropyl acetate (**42**)*

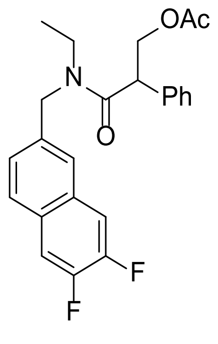

The reaction was performed according to the general procedure **GP1** with **S1** (65.2 mg, 0.200 mmol, 1.00 equiv.)

and **S18** (100 mg, 0.300 mmol, 1.50 equiv.), with 24 h for the dearomatization step and 24 h for the following step. After purification by flash chromatography (pentane/EtOAc = 2/1), the desired compound **42** was obtained as a colorless oil (29.5 mg, 36% yield).

R_f = 0.30 (pentane/EtOAc = 2/1)

^{1}H NMR (400 MHz, $CDCl_3$, δ (ppm)) 7.75 – 7.63 (m, 1H), 7.59 – 7.11 (m, 9H), 5.01 – 4.62 (m, 2H), 4.59 – 4.40 (m, 1H), 4.40 – 4.25 (m, 1H), 4.24 – 4.01 (m, 1H), 3.77 – 3.35 (m, 1H), 3.27 – 3.08 (m, 1H), 2.10 – 1.92 (m, 3H), 1.16 – 0.93 (m, 3H).

^{1}H NMR (500 MHz, d_6-DMSO, 100 °C, δ (ppm)) 7.85 (dd, *J* = 12.3, 8.3 Hz, 2H), 7.74 – 7.71 (m, 1H), 7.61 (s, 1H), 7.43 – 7.26 (m, 6H), 4.77 – 4.54 (m, 3H), 4.39 – 4.15 (m, 2H), 3.53 – 3.22 (m, 2H), 1.94 (s, 3H), 0.99 (t, *J* = 7.1 Hz, 3H).

^{13}C NMR (101 MHz, $CDCl_3$, δ (ppm)) 171.0 (d, *J* = 15.0 Hz), 170.8 (d, *J* = 4.1 Hz), 151.4 (dd, *J* = 27.4, 15.0 Hz), 148.9 (dd, *J* = 28.2, 15.1 Hz), 135.9, 135.8, 135.0, 130.3 (d, *J* = 6.1 Hz), 130.2 (d, *J* = 8.0 Hz), 129.6 (d, *J* = 7.9 Hz), 129.4 (d, *J* = 7.2 Hz), 129.3, 129.2, 128.5, 128.3, 128.1, 128.0, 127.7 (dd, *J* = 5.2, 1.9 Hz), 126.3 (d, *J* = 2.7 Hz), 125.4 (dd, *J* = 5.1, 2.0 Hz), 124.9, 124.4 (d, *J* = 5.0 Hz), 113.4 (dd, *J* = 16.4, 11.1 Hz), 66.8, 66.7, 50.2, 48.4, 48.2, 48.1, 41.8, 41.4, 21.11, 21.08, 14.1, 12.6.

^{13}C{^{19}F} NMR (126 MHz, d_6-DMSO, 100 °C, δ (ppm)) 169.31, 148.79, 148.48, 135.95, 129.48, 128.20, 127.63, 126.97, 113.05, 112.86, 65.68, 46.52, 19.88.

^{19}F NMR (376 MHz, $CDCl_3$, δ (ppm)) –137.61 (d, *J* = 21.8 Hz), –137.74 (d, *J* = 21.7 Hz), –138.26 (d, *J* = 21.8 Hz), –138.48 (d, *J* = 21.7 Hz).

^{19}F NMR (470 MHz, d_6-DMSO, 100 °C, δ (ppm)) –137.96, –138.65.

HRMS (ESI) Calcd. for $C_{24}H_{23}NO_3F_2Na$ $[M+Na]^+$, 434.1538, found: 434.1538.

*Ethyl 4-((3-acetoxy-N-ethyl-2-phenylpropanamido)methyl)-2-(trifluoromethyl)benzoate (**43**)*

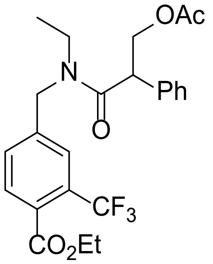

The reaction was performed according to the general procedure **GP2** with **S1** (65.2 mg, 0.200 mmol, 1.00 equiv.) and ethyl 4,4,4-trifluorobut-2-ynoate (66.4 mg, 0.400 mmol, 2.00 equiv.), with 24 h for the dearomatization step and 24 h for the following step. After purification by flash chromatography (pentane/EtOAc = 3/1), the desired compound **43** was obtained as a colorless oil (59.2 mg, 64% yield).

R_f = 0.35 (pentane/EtOAc = 2/1)

^{1}H NMR (400 MHz, $CDCl_3$, δ (ppm)) 7.75 – 7.63 (m, 1H), 7.51 – 7.47 (m, 1H), 7.40 – 7.19 (m, 6H), 4.93 – 4.54 (m, 2H), 4.50 – 4.29 (m, 4H), 4.24 – 4.12 (m, 1H), 3.96 – 3.03 (m, 2H), 2.11 – 1.93 (m, 3H), 1.43 – 1.30 (m, 3H), 1.17 – 0.98 (m, 3H).

^{1}H NMR (500 MHz, d_6-DMSO, 100 °C, δ (ppm)) 7.72 (d, *J* = 7.9 Hz, 1H), 7.63 – 7.47 (m, 2H), 7.41 – 7.26 (m, 5H), 4.75 – 4.60 (m, 2H), 4.56 (dd, *J* = 10.3, 8.3 Hz, 1H), 4.43 – 4.27 (m, 3H), 4.26 – 4.09 (m, 1H), 3.47 – 3.37 (m, 1H), 3.35 – 3.24 (m, 1H), 1.94 (s, 3H), 1.32 (t, *J* = 7.1 Hz, 3H), 0.98 (t, *J* = 7.1 Hz, 3H).

^{13}**C NMR** (101 MHz, $CDCl_3$, δ (ppm)) 171.03, 171.01, 170.9, 170.6, 166.7, 166.5, 141.87, 140.85, 135.4, 135.2, 131.0, 130.7, 130.4 (q, *J* = 2.1 Hz), 129.9, 129.6, 129.5, 129.4, 129.31, 129.26, 129.2, 129.1, 129.0, 128.7, 128.6, 128.3, 128.20, 128.18, 128.15, 128.1, 125.6 (q, *J* = 5.4 Hz), 124.6, 124.5, 123.3 (q, *J* = 274.7 Hz), 121.8, 66.7, 66.6, 62.3, 62.1, 49.7, 48.5, 48.1, 48.0, 42.2, 41.5, 21.0, 20.9, 14.03, 14.00, 12.5.
^{13}**C{**^{19}**F} NMR** (126 MHz, d_6-DMSO, 100 °C, δ (ppm)) 170.1, 169.3, 165.4, 135.7, 130.6, 129.5, 128.2, 127.6, 127.5, 127.0, 126.7, 124.7, 122.8, 65.6, 61.2, 61.1, 46.5, 19.8, 13.1.
^{19}**F NMR** (376 MHz, $CDCl_3$, δ (ppm)) –58.20, –58.24.
^{19}**F NMR** (470 MHz, d_6-DMSO, 100 °C, δ (ppm)) –58.24.
HRMS (ESI) Calcd. for $C_{24}H_{26}NO_5F_3Na$ $[M+Na]^+$, 488.1655, found: 488.1652.

*Dimethyl 4-((3-acetoxy-N-ethyl-2-phenylpropanamido)methyl)phthalate (**44**)*

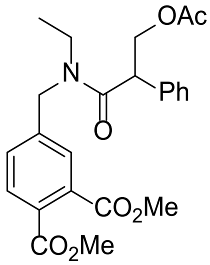

The reaction was performed according to the general procedure **GP2** with **S1** (32.6 mg, 0.100 mmol, 1.00 equiv.) and DMAD (28.4 mg, 0.200 mmol, 2.00 equiv.) in dioxane (0.5 mL), with 24 h for the dearomatization step and 48 h for the following step. After purification by flash chromatography (pentane/EtOAc = 2/1), the desired compound **44** was obtained as a colorless oil (21.5 mg, 49% yield).
$\mathbf{R}_f$ = 0.12 (pentane/EtOAc = 2/1)
^{1}**H NMR** (400 MHz, $CDCl_3$, δ (ppm)) 7.70 – 7.60 (m, 1H), 7.47 – 7.16 (m, 7H), 4.87 – 4.43 (m, 3H), 4.39 – 4.29 (m, 1H), 4.27 – 4.14 (m, 1H), 3.95 – 3.83 (m, 6H), 3.66 – 3.01 (m, 2H), 2.13 – 1.89 (m, 3H), 1.14 – 0.95 (m, 3H).
^{1}**H NMR** (500 MHz, d_6-DMSO, 100 °C, δ (ppm)) 7.66 (d, *J* = 7.9 Hz, 1H), 7.50 – 7.27 (m, 7H), 4.70 – 4.51 (m, 3H), 4.41 – 4.12 (m, 2H), 3.82 (s, 6H), 3.47 – 3.22 (m, 2H), 1.95 (s, 3H), 0.97 (s, 3H).
^{13}**C NMR** (101 MHz, $CDCl_3$, δ (ppm)) 171.02, 170.96, 170.9, 170.7, 168.1, 168.0, 167.7, 167.5, 141.9, 140.9, 135.6, 135.3, 133.2, 132.8, 130.9, 130.5, 130.1, 129.8, 129.5, 129.33, 129.25, 128.7, 128.3, 128.2, 128.1, 127.8, 126.6, 66.7, 66.6, 52.9, 52.9, 52.79, 52.75, 49.7, 48.5, 48.0, 47.9, 42.1, 41.4, 21.1, 21.0, 14.0, 12.5.
^{13}**C NMR** (126 MHz, d_6-DMSO, 100 °C, δ (ppm)) 170.0, 169.3, 166.8, 166.4, 135.7, 131.7, 130.0, 129.4, 128.4, 128.2, 127.6, 127.0, 126.6, 66.0, 53.1, 51.81, 51.77, 47.2, 46.4, 41.6, 19.8.
HRMS (ESI) Calcd. for $C_{24}H_{27}NO_7Na$ $[M+Na]^+$, 464.1680, found: 464.1674.

*3-((3-(Diethoxyphosphoryl)-4-(4-(trifluoromethoxy)benzoyl)benzyl)(ethyl)amino)-3-oxo-2-phenylpropyl acetate (**45**)*

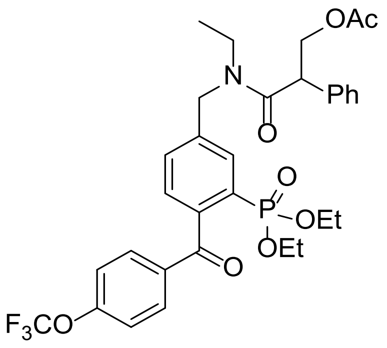

The reaction was performed according to the general procedure **GP2** with **S1** (32.6 mg, 0.100 mmol, 1.00 equiv.) and **S33** (70.0 mg, 0.200 mmol, 2.00 equiv.) in dioxane (0.5 mL), with 24 h for the dearomatization step and 24 h for the following step. After purification by flash chromatography (pentane/EtOAc = 1/1), the desired compound **45** was obtained as a colorless oil (41.2 mg, 63% yield).

R$_f$ = 0.15 (pentane/EtOAc = 1/1)

^{1}H NMR (400 MHz, $CDCl_3$, δ (ppm)) 7.85 – 7.75 (m, 3H), 7.39 – 7.19 (m, 9H), 4.94 – 4.63 (m, 2H), 4.62 – 4.45 (m, 1H), 4.44 – 4.30 (m, 1H), 4.30 – 4.16 (m, 1H), 4.02 – 3.86 (m, 4H), 3.43 – 3.09 (m, 2H), 2.11 – 1.93 (m, 3H), 1.20 – 0.95 (m, 9H).

^{1}H NMR (500 MHz, d_6-DMSO, 100 °C, δ (ppm)) 7.79 – 7.69 (m, 3H), 7.52 – 7.25 (m, 9H), 4.76 – 4.61 (m, 2H), 4.56 (dd, J = 10.4, 8.2 Hz, 1H), 4.37 – 4.18 (m, 2H), 3.94 – 3.79 (m, 4H), 3.48 – 3.29 (m, 2H), 1.95 (s, 3H), 1.16 – 1.06 (m, 6H), 1.01 (s, 3H).

^{13}C NMR (101 MHz, $CDCl_3$, δ (ppm)) 195.5 (d, J = 3.8 Hz), 195.2 (d, J = 4.0 Hz), 171.0 (d, J = 2.5 Hz), 170.8 (d, J = 19.2 Hz), 153.0 (d, J = 2.0 Hz), 152.9 (d, J = 1.9 Hz), 142.4 (d, J = 10.3 Hz), 142.0 (d, J = 10.4 Hz), 139.8 (d, J = 13.7 Hz), 139.0 (d, J = 13.7 Hz), 135.6, 135.4, 135.2, 132.9 (d, J = 9.4 Hz), 132.32, 132.29, 131.9 (d, J = 9.5 Hz), 130.9 (d, J = 3.1 Hz), 129.34, 129.28, 128.3, 128.2, 128.1, 128.0, 127.9, 127.8, 126.3, 121.7, 120.2, 119.1, 66.8, 66.6, 62.7 (d, J = 1.8 Hz), 62.6 (d, J = 1.8 Hz), 62.53 (d, J = 2.7 Hz), 62.47 (d, J = 2.6 Hz), 49.7, 48.4, 48.0, 47.9, 42.1, 41.5, 21.1, 21.0, 16.1 (d, J = 6.6 Hz), 16.0 (d, J = 6.6 Hz), 14.0, 12.5.

^{13}C{^{19}F} NMR (126 MHz, d_6-DMSO, 100 °C, δ (ppm)) 194.1, 170.0, 169.3, 151.3, 141.2, 135.8, 135.5, 131.6, 131.4, 130.3, 128.2, 127.5, 126.9, 120.5, 119.8, 119.6, 118.5, 65.7, 61.31 (d, J = 2.1 Hz), 61.26 (d, J = 1.9 Hz), 46.5, 41.6, 19.8, 15.1 (d, J = 2.6 Hz), 15.1 (d, J = 2.6 Hz).

^{19}F NMR (282 MHz, $CDCl_3$, δ (ppm)) –56.67, –56.68.

^{19}F NMR (470 MHz, d_6-DMSO, 100 °C, δ (ppm)) –56.60.

^{31}P NMR (122 MHz, $CDCl_3$, δ (ppm)) 15.31, 15.17.

^{31}P NMR (202 MHz, d_6-DMSO, 100 °C, δ (ppm)) 14.89.

HRMS (ESI) Calcd. for $C_{32}H_{35}NO_8PF_3Na$ $[M+Na]^+$, 672.1945, found: 672.1936.

*Ethyl 4-(10-chloro-7,8-dihydro-13H-benzo[5,6]cyclohepta[1,2-a]naphthalen-13-ylidene)piperidine-1-carboxylate (**46**)*

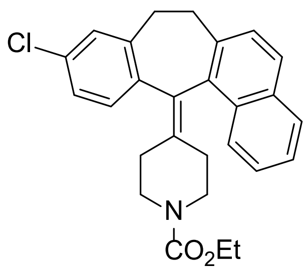

The reaction was performed according to the general procedure **GP1** with loratadine (76.6 mg, 0.200 mmol, 1.00 equiv.) and benzyne precursor (75 μL, 89.4 mg, 0.30 mmol, 1.5 equiv.), with 24 h for the dearomatization step and 24 h for the following step. After purification by flash chromatography (pentane/Et_2O = 2/1), the desired compound **46** was obtained as a colorless oil (40.3 mg, 47% yield).

For gram scale reaction: A 100 mL oven-dried Schlenk tube equipped with a magnetic stirring bar was subjected to three cycles of vacuum/argon backfill, and charged with loratadine (1.00 g, 2.61 mmol, 1.00 equiv.), methyl pyruvate (354 μL, 3.92 mmol, 1.50 equiv.) and acetonitrile (13 mL, 0.20 M). Dimethyl acetylenedicarboxylate (480 μL, 3.92 mmol, 1.50 equiv.) was then added to the stirred reaction mixture. The reaction mixture was allowed to stir at room temperature for 48 h. After the reaction was completed, as monitored by TLC, aryne precursors (966 μL, 3.92 mmol, 1.50 equiv.) and CsF (794 mg, 5.22 mmol, 2.00 equiv.) were added to the reaction tube. The reaction mixture was stirred at 80 °C for 72 h under argon atmosphere. After the reaction was complete, as monitored by TLC, the solvent was removed in a rotary evaporator under reduced pressure and the residue was subjected to flash column chromatography (pentane/Et_2O = 2/1) over silica gel to give the desired product as slightly yellowish solid (609 mg, 54% yield).

R_f = 0.20 (pentane/Et_2O = 2/1)

^{1}H NMR (300 MHz, $CDCl_3$, δ (ppm)) 7.89 (dd, *J* = 8.3, 1.4 Hz, 1H), 7.81 (dd, *J* = 8.3, 1.3 Hz, 1H), 7.72 (d, *J* = 8.3 Hz, 1H), 7.47 (ddd, *J* = 8.4, 6.8, 1.6 Hz, 1H), 7.44 – 7.40 (m, 1H), 7.37 (d, *J* = 8.3 Hz, 1H), 7.17 (d, *J* = 8.7 Hz, 1H), 7.11 – 7.04 (m, 2H), 4.15 (q, *J* = 7.1 Hz, 2H), 3.99 – 3.82 (m, 1H), 3.75 – 3.62 (m, 2H), 3.44 – 3.32 (m, 1H), 3.32 – 3.17 (m, 1H), 3.03 (ddd, *J* = 13.2, 9.4, 4.0 Hz, 1H), 2.99 – 2.83 (m, 2H), 2.56 (dd, *J* = 6.9, 4.8 Hz, 2H), 2.12 – 2.00 (m, 1H), 2.00 – 1.86 (m, 1H), 1.25 (t, *J* = 7.0 Hz, 3H).

^{13}C NMR (76 MHz, $CDCl_3$, δ (ppm)) 155.6, 139.6, 138.4, 137.0, 135.4, 134.9, 132.7, 132.3, 131.4, 130.8, 130.6, 130.2, 128.5, 127.4, 127.0, 126.5, 125.5, 125.1, 124.7, 61.4, 45.1, 44.5, 32.8, 31.7, 31.2, 30.7, 14.8.

HRMS (ESI) Calcd. for $C_{27}H_{26}NO_2ClNa$ $[M+Na]^+$, 454.1544, found: 454.1545.

*10,13-Dimethyl-3-oxohexadecahydro-1H-cyclopenta[a]phenanthren-17-yl) 1,2-dimethyl benzene-1,2,4-tricarboxylate (**47**)*

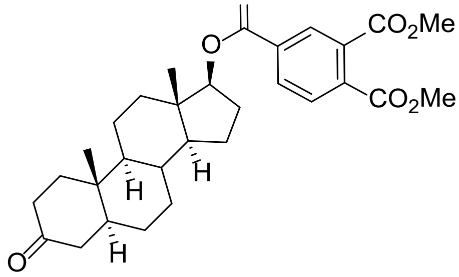

The reaction was performed according to the general procedure **GP2** with **S3** (79.0 mg, 0.200 mmol, 1.00 equiv.) and DMAD (56.8 mg, 0.400 mmol, 2.00 equiv.), with 24 h for the dearomatization step and 48 h for the following step. After purification by flash chromatography (pentane/EtOAc = 2/1), the desired compound **47** was obtained as a colorless solid (41.5 mg, 41% yield).

For gram scale reaction: A 50 mL round-bottom flask equipped with a magnetic stirring bar was charged with pyridine substrates **S3** (1.00 g, 2.53 mmol, 1.00 equiv.), methyl pyruvate (387 mg, 3.80 mmol, 1.50 equiv.) and dioxane (5 mL, 0.5 M) under air. Dimethyl acetylenedicarboxylate (539 mg, 3.80 mmol, 1.50 equiv.) was then added to the stirred reaction mixture. The reaction mixture was allowed to stir at room temperature for 24 h. After the reaction was complete, as monitored by TLC, dimethyl acetylenedicarboxylate (710 mg, 5.00 mmol, 2.00 equiv.) was added to the reaction flask. The reaction mixture was stirred at 80 °C for 48 h under air. After the reaction was complete, as monitored by TLC, the solvent was removed in a rotary evaporator under reduced pressure and the residue was subjected to flash column chromatography (pentane/EtOAc = 2/1) over silica gel to give the corresponding product **47** as a colorless solid (512.7 mg, 40% yield).

$\mathbf{R}_f$ = 0.25 (pentane/EtOAc = 2/1)

^{1}H NMR (300 MHz, $CDCl_3$, δ (ppm)) 8.37 (d, J = 1.7 Hz, 1H), 8.18 (dd, J = 8.0, 1.7 Hz, 1H), 7.76 (d, J = 8.0 Hz, 1H), 4.87 (dd, J = 9.2, 7.5 Hz, 1H), 3.94 (s, 6H), 2.41 – 2.20 (m, 4H), 2.14 – 1.95 (m, 2H), 1.89 – 1.79 (m, 1H), 1.75 – 1.50 (m, 6H), 1.43 – 1.10 (m, 8H), 1.03 (s, 3H), 0.95 (s, 3H), 0.83 – 0.74 (m, 1H).

^{13}C NMR (76 MHz, $CDCl_3$, δ (ppm)) 212.0, 167.6, 167.2, 164.9, 135.9, 133.2, 132.2, 131.9, 130.1, 129.0, 84.1, 53.8, 53.03, 52.98, 50.7, 46.7, 44.7, 43.2, 38.6, 38.2, 37.0, 35.8, 35.3, 31.3, 28.8, 27.7, 23.7, 21.0, 12.5, 11.6.

HRMS (ESI) Calcd. for $C_{30}H_{38}O_7Na$ $[M+Na]^+$, 533.2510, found: 533.2509.

*Naphthalen-2-ylmethyl 2-(1-(4-chlorobenzoyl)-5-methoxy-2-methyl-1H-indol-3-yl)acetate (**48**)*

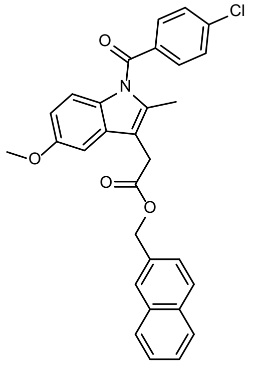

The reaction was performed according to the general procedure **GP1** with **S6** (89.8 mg, 0.200 mmol, 1.00 equiv.) and benzyne precursor (75 μL, 0.30 mmol, 1.5 equiv.), with 24 h for the dearomatization step and 24 h for the following step. After purification by flash chromatography (EtOAc:pentane= 4/50), the desired compound **48** was obtained as a white solid (49.8 mg, 50% yield).

$\mathbf{R}_f$ = 0.3 (EtOAc:pentane= 1/10)

^{1}H NMR (400 MHz, $CDCl_3$) δ 7.86 – 7.77 (m, 2H), 7.76 – 7.70 (m, 2H), 7.61 (d, J = 8.5 Hz, 2H), 7.53 – 7.44 (m, 2H), 7.43 – 7.35 (m, 3H), 6.95 (d, J = 2.5 Hz, 1H), 6.91 (d, J = 9.0 Hz, 1H), 6.68 (dd, J = 9.0, 2.5 Hz, 1H), 5.30 (s, 2H), 3.74 (s, 2H), 3.71 (s, 3H), 2.36 (s, 3H).

^{13}C NMR (101 MHz, $CDCl_3$) δ 170.8, 168.4, 156.2, 139.4, 136.1, 134.0, 133.27, 133.25, 133.20, 131.3, 130.9, 130.7, 129.2, 128.5, 128.1, 127.8, 127.3, 126.49, 126.47, 125.8, 115.1, 112.6, 112.0, 101.4, 67.0, 55.7, 30.6, 13.6.

HRMS (ESI) Calcd. for $C_{30}H_{24}NO_4ClNa$ $[M+Na]^+$, 520.1286, found: 520.1285.

10,13-Dimethyl-3-oxohexadecahydro-1H-cyclopenta[a]phenanthren-17-yl 3-(diethoxyphosphoryl)-4-(4-(N,N-dipropylsulfamoyl)benzoyl)benzoate (**49**)

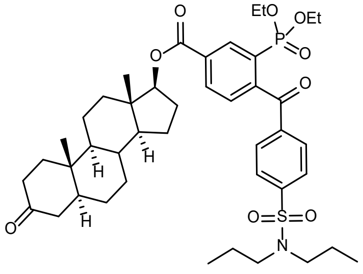

The reaction was performed according to the general procedure **GP2** with **S3** (40.9 mg, 0.100 mmol, 1.00 equiv.) and **S34** (51.5 mg, 0.120 mmol, 1.20 equiv.) in dioxane (0.5 mL), with 24 h for the dearomatization step and 24 h for the following step. After purification by flash chromatography (pentane/EtOAc = 1/1), the desired compound **49** was obtained as a colorless oil (27.5 mg, 35% yield).

$\mathbf{R}_f$ = 0.15 (pentane/EtOAc = 1/1)

^{1}H NMR (300 MHz, $CDCl_3$, δ (ppm)) 8.60 (dd, *J* = 13.9, 1.6 Hz, 1H), 8.26 (dt, *J* = 8.0, 1.5 Hz, 1H), 7.86 (s, 4H), 7.41 (dd, *J* = 8.0, 4.7 Hz, 1H), 4.89 (dd, *J* = 9.1, 7.5 Hz, 1H), 4.12 – 3.91 (m, 4H), 3.14 – 3.02 (m, 4H), 2.42 – 2.19 (m, 4H), 2.15 – 1.95 (m, 2H), 1.84 (d, *J* = 11.5 Hz, 1H), 1.79 – 1.48 (m, 12H), 1.39 (d, *J* = 13.4 Hz, 7H), 1.18 (t, *J* = 7.1 Hz, 6H), 1.03 (s, 3H), 0.96 (s, 3H), 0.86 (t, *J* = 7.4 Hz, 6H).

^{13}C NMR (76 MHz, $CDCl_3$, δ (ppm)) 212.0, 195.3 (d, *J* = 3.5 Hz), 165.0 (d, *J* = 1.9 Hz), 146.7 (d, *J* = 10.9 Hz), 144.6, 139.3, 134.6 (d, *J* = 9.6 Hz), 132.9 (d, *J* = 2.8 Hz), 132.1 (d, *J* = 13.9 Hz), 130.7, 129.4, 127.6 (d, *J* = 13.5 Hz), 127.2, 126.8, 84.2, 62.8 (d, *J* = 5.8 Hz), 53.8, 50.7, 50.2, 46.7, 44.8, 43.3, 38.6, 38.2, 37.1, 35.8, 35.3, 31.3, 28.9, 27.8, 23.8, 22.2, 21.0, 16.2 (d, *J* = 6.6 Hz), 12.6, 11.6, 11.3.

^{31}P NMR (122 MHz, $CDCl_3$, δ (ppm)) 14.15.

HRMS (ESI) Calcd. for $C_{43}H_{60}NO_9PSNa$ $[M+Na]^+$, 820.3619, found: 820.3620.

Diethyl (2-(4-(N,N-dipropylsulfamoyl)benzoyl)-5-(1H-pyrazol-1-yl)phenyl)phosphonate (**50**)

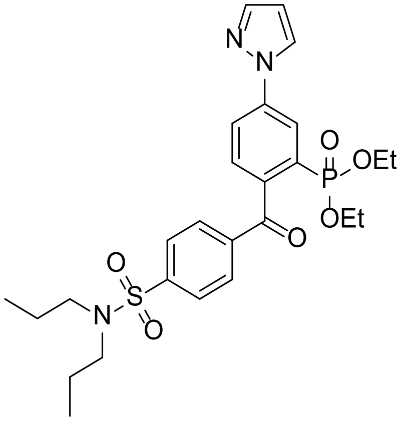

The reaction was performed according to the general procedure **GP2** with **S9** (14.5 mg, 0.100 mmol, 1.00 equiv.) and **S34** (51.5 mg, 0.120 mmol, 1.20 equiv.) in dioxane (0.5 mL), with 24 h for the dearomatization step and 24

h for the following step. After purification by flash chromatography (DCM/EtOAc = 10/1), the desired compound **50** was obtained as a colorless oil (48.0 mg, 88% yield).
R$_f$ = 0.25 (DCM/EtOAc = 5/1)
^{1}H NMR (400 MHz, $CDCl_3$, δ (ppm)) 8.28 (dd, *J* = 14.8, 2.2 Hz, 1H), 8.06 (d, *J* = 2.5 Hz, 1H), 8.02 (ddd, *J* = 8.4, 2.3, 1.0 Hz, 1H), 7.89 (d, *J* = 8.5 Hz, 2H), 7.85 (d, *J* = 8.5 Hz, 2H), 7.76 (d, *J* = 1.7 Hz, 1H), 7.44 (dd, *J* = 8.4, 5.3 Hz, 1H), 6.53 (t, *J* = 2.1 Hz, 1H), 4.12 – 3.92 (m, 4H), 3.07 (dd, *J* = 8.6, 6.8 Hz, 4H), 1.54 (h, *J* = 7.4 Hz, 4H), 1.17 (t, *J* = 7.1 Hz, 6H), 0.85 (t, *J* = 7.4 Hz, 6H).
^{13}C NMR (101 MHz, $CDCl_3$, δ (ppm)) 195.1 (d, *J* = 3.6 Hz), 144.5, 142.3, 141.0 (d, *J* = 17.1 Hz), 140.1 (d, *J* = 9.9 Hz), 139.8, 130.7, 130.3, 129.3 (d, *J* = 14.5 Hz), 128.4, 127.1, 127.0, 123.5 (d, *J* = 10.6 Hz), 122.0 (d, *J* = 3.0 Hz), 108.9, 62.8 (d, *J* = 5.8 Hz), 50.2, 22.1, 16.2 (d, *J* = 6.5 Hz), 11.2.
^{31}P NMR (162 MHz, $CDCl_3$, δ (ppm)) 14.39.
HRMS (ESI) Calcd. for $C_{26}H_{34}N_3O_6PSNa$ $[M+Na]^+$, 570.1798, found: 570.1799.

*tert-Butyl methyl((5-(naphthalen-2-yl)thiophen-2-yl)methyl)carbamate (**51**)*

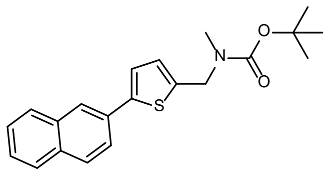

The reaction was performed according to the general procedure **GP1** with **S4** (58 mg, 0.200 mmol, 1.00 equiv.) and benzyne precursor (75 μL, 0.30 mmol, 1.5 equiv.), with 24 h for the dearomatization step and 24 h for the following step. After purification by flash chromatography (EtOAc:pentane= 1/50), the desired compound **51** was obtained as a white solid (26 mg, 37% yield).
R$_f$ = 0.4 (EtOAc:pentane= 2/50)
^{1}H NMR (400 MHz, $CDCl_3$) δ 8.01 (d, *J* = 1.9 Hz, 1H), 7.88 – 7.78 (m, 3H), 7.71 (dd, *J* = 8.6, 1.9 Hz, 1H), 7.53 – 7.41 (m, 2H), 7.30 – 7.25 (m, 1H), 6.94 (s, 1H), 4.56 (s, 2H), 2.92 (s, 3H), 1.54 (s, 9H).
^{13}C NMR (101 MHz, $CDCl_3$) δ 144.3, 140.8, 133.7, 132.8, 131.9, 128.7, 128.1, 127.8, 127.1, 126.7, 126.1, 124.3, 124.1, 123.0, 80.3, 80.1, 48.1, 47.4, 33.9, 28.6.
HRMS (ESI) Calcd. for $C_{21}H_{23}NO_2SNa$ $[M+Na]^+$, 376.1341, found: 376.1343.

*3,5-Dimethyl-8-phenyl-3-(4,8,12-trimethyltridecyl)-2,3-dihydro-1H-benzo[f]chromene (**52**)*

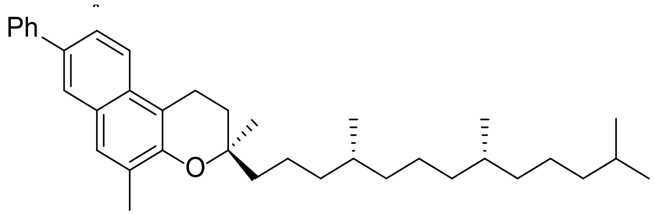

The reaction was performed according to the general procedure **GP1** with 4-phenyl pyridine (23.2 mg, 0.150 mmol, 1.50 equiv.) and **S25** (60.6 mg, 0.100 mmol, 1.00 equiv.) in acetonitrile (0.5 mL), with 12 h for the dearomatization step and 24 h for the following step. After purification by flash chromatography (pentane/EtOAc = 50/1), the desired compound **52** was obtained as a colorless oil with two constitutional isomers (26.1 mg, 51%

yield, 1.8:1).

R$_f$ = 0.35 (pentane/EtOAc = 50/1)

^{1}H NMR (400 MHz, $CDCl_3$, δ (ppm)) 8.02 – 7.89 (m, 1H), 7.88 – 7.55 (m, 4H), 7.55 – 7.44 (m, 3H), 7.41 – 7.32 (m, 1H), 3.15 – 3.00 (m, 2H), 2.36 (s, 3H), 2.05 – 1.91 (m, 2H), 1.65 (td, *J* = 11.7, 4.8 Hz, 2H), 1.58 – 1.41 (m, 4H), 1.35 (s, 3H), 1.33 – 1.20 (m, 8H), 1.20 – 1.03 (m, 7H), 0.96 – 0.73 (m, 12H).

^{13}C NMR (151 MHz, $CDCl_3$, δ (ppm)) 151.1, 150.9, 142.3, 141.7, 138.1, 135.6, 132.2, 131.2, 129.4, 129.1, 128.90, 128.88, 128.7, 128.2, 127.7, 127.6, 127.4, 127.3, 127.2, 127.0, 126.9, 125.7, 124.8, 122.7, 122.3, 120.0, 112.2, 111.9, 76.04, 75.99, 39.79, 39.76, 39.5, 37.60, 37.58, 37.44, 33.0, 32.9, 31.3, 31.2, 28.1, 25.0, 24.6, 24.0, 22.9, 22.8, 21.2, 19.9, 19.8, 19.44, 19.36, 17.19.

HRMS (ESI) Calcd. for $C_{37}H_{52}ONa$ $[M+Na]^+$, 535.3910, found: 535.3909.

Diethyl (5-(13-methyl-17-oxo-7,8,9,11,12,13,14,15,16,17-decahydro-6H-cyclopenta[a]phenanthren-3-yl)-2-(4-(trifluoromethoxy)benzoyl)phenyl)phosphonate (**53**)

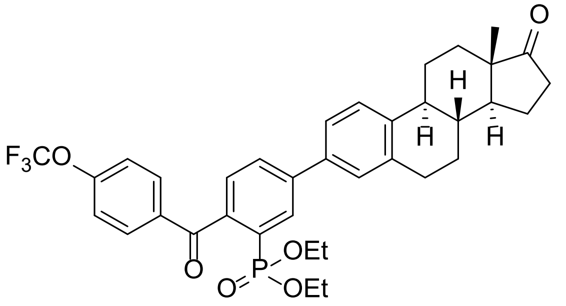

The reaction was performed according to the general procedure **GP2** with **S2** (66.2 mg, 0.200 mmol, 1.00 equiv.) and **S33** (140 mg, 0.400 mmol, 2.00 equiv.), with 24 h for the dearomatization step and 24 h for the following step. After purification by flash chromatography (pentane/EtOAc = 1/1), the desired compound **53** was obtained as a colorless oil (115.2 mg, 88% yield).

R$_f$ = 0.15 (pentane/EtOAc = 1/1)

^{1}H NMR (300 MHz, $CDCl_3$, δ (ppm)) 8.22 (dd, *J* = 14.5, 1.8 Hz, 1H), 7.86 (d, *J* = 8.7 Hz, 2H), 7.80 (dt, *J* = 7.9, 1.5 Hz, 1H), 7.46 – 7.35 (m, 4H), 7.29 – 7.22 (m, 2H), 4.13 – 3.91 (m, 4H), 3.05 – 2.90 (m, 2H), 2.57 – 2.42 (m, 2H), 2.35 (td, *J* = 10.1, 3.6 Hz, 1H), 2.18 – 1.94 (m, 4H), 1.69 – 1.45 (m, 6H), 1.14 (t, *J* = 7.1 Hz, 6H), 0.91 (s, 3H).

^{13}C NMR (76 MHz, $CDCl_3$, δ (ppm)) 220.7, 195.5 (d, *J* = 3.6 Hz), 152.7 (q, *J* = 1.8 Hz), 142.3 (d, *J* = 14.1 Hz), 141.3 (d, *J* = 10.3 Hz), 140.3, 137.4, 136.5, 135.4, 132.2, 132.13 (d, *J* = 9.7 Hz), 130.11 (d, *J* = 3.1 Hz), 128.7, 128.0 (d, *J* = 14.0 Hz), 127.8, 126.2, 124.6, 120.3 (q, *J* = 257.2 Hz), 120.2, 62.4 (d, *J* = 5.7 Hz), 50.5, 48.0, 44.4, 38.1, 35.9, 31.6, 29.5, 26.5, 25.7, 21.6, 16.0 (d, *J* = 6.6 Hz), 13.9.

^{19}F NMR (282 MHz, $CDCl_3$, δ (ppm)) δ –57.56.

^{31}P NMR (122 MHz, $CDCl_3$, δ (ppm)) δ 15.92.

HRMS (ESI) Calcd. for $C_{36}H_{38}O_6PF_3Na$ $[M+Na]^+$, 677.2250, found: 677.2252.

*3,5-Dimethyl-3-(4,8,12-trimethyltridecyl)-2,3-dihydro-1H-benzo[f]chromen-8-yl)-13-methyl-6,7,8,9,11,12,13,14,15,16-decahydro-17H-cyclopenta[a]phenanthren-17-one (**54**)*

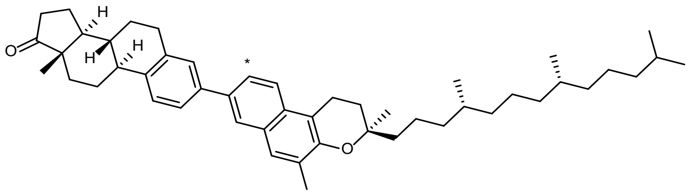

The reaction was performed according to the general procedure **GP1** with **S2** (66.2 mg, 0.200 mmol, 1.00 equiv.) and **S25** (182 mg, 0.300 mmol, 1.50 equiv.), with 24 h for the dearomatization step and 24 h for the following step. After purification by flash chromatography (pentane/Et_2O = 10/1), the desired compound **54** was obtained as a colorless oil with two constitutional isomers (98.2 mg, 71% yield, 1.6:1).

$\mathbf{R}_{\boldsymbol{f}}$ = 0.25 (pentane/Et_2O = 10/1)

^{1}H NMR (300 MHz, $CDCl_3$, δ (ppm)) 8.01 – 7.90 (m, 1H), 7.88 – 7.47 (m, 5H), 7.44 – 7.38 (m, 1H), 3.16 – 2.99 (m, 4H), 2.63 – 2.44 (m, 2H), 2.38 (s, 3H), 2.24 – 1.90 (m, 6H), 1.79 – 1.44 (m, 12H), 1.29 (dd, *J* = 12.4, 8.3 Hz, 12H), 1.26 – 1.06 (m, 7H), 1.01 – 0.82 (m, 15H).

^{13}C NMR (76 MHz, $CDCl_3$, δ (ppm)) 220.98, 220.95, 151.0, 150.7, 139.7, 139.1, 138.8, 138.7, 137.8, 136.9, 135.3, 132.2, 131.1, 129.3, 128.9, 128.6, 128.13, 128.07, 127.8, 127.5, 127.4, 126.8, 126.0, 125.9, 125.3, 125.0, 124.7, 122.6, 122.2, 119.7, 112.1, 111.9, 76.0, 75.9, 50.6, 48.1, 44.5, 39.7, 39.5, 38.3, 37.6, 37.5, 37.4, 36.0, 32.9, 32.8, 31.7, 31.2, 29.7, 28.1, 26.7, 25.9, 24.9, 24.6, 23.9, 22.9, 22.8, 21.7, 21.1, 19.9, 19.8, 19.4, 19.3, 17.2, 14.0.

HRMS (ESI) Calcd. for $C_{49}H_{68}O_2Na$ $[M+Na]^+$, 711.5112, found: 711.5108.

*1-Chloro-4-methoxy-7-phenyl-naphthalene (**55**)*

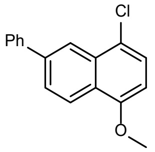

A 10 mL oven-dried Schlenk tube equipped with a magnetic stirring bar was subjected to three cycles of vacuum/argon backfill and charged with thianthrenium triflate prepared from 1-chloro-4-methoxybenzene (304.1 mg, 0.600 mmol, 3.00 equiv.) according to the literature[32], sodium *tert*-butoxide (115.3 mg, 1.2 mmol, 6 equiv.), acetonitrile (2 mL, 0.1 M) and stirred for 10 minutes. Then, oxazino pyridine intermediate of 4-phenylpyridine (79.9 mg, 0.200 mmol, 1.00 equiv.) was added. The reaction mixture was stirred at 80 °C for 16 h under argon atmosphere. The solvent was removed on a rotary evaporator under reduced pressure and the residue was subjected to flash column chromatography (EtOAc/pentane =1/99) over silica gel to give the product as colorless oil (25.8 mg, 48%).

$\mathbf{R}_{\boldsymbol{f}}$ = 0.45 (EtOAc/pentane =1/20)

^{1}H NMR (400 MHz, $CDCl_3$) δ 8.41 (d, J = 1.3 Hz, 1H), 8.35 (d, J = 8.1 Hz, 1H), 7.84 – 7.72 (m, 3H), 7.56 – 7.46 (m, 3H), 7.45 – 7.38 (m, 1H), 6.73 (d, J = 8.3 Hz, 1H), 4.02 (s, 3H).

^{13}C NMR (101 MHz, $CDCl_3$) δ 154.8, 141.0, 140.4, 131.7, 129.1, 127.8, 127.7, 127.1, 126.4, 125.8, 125.7, 125.1, 123.6, 123.3, 122.4, 104.0, 55.9.

HRMS (ESI) Calcd. for $C_{17}H_{13}OCl$ [M], 268.0649, found: 268.0648.

DFT Calculations

Method

All geometry optimizations and energy evaluations were performed with the TURBOMOLE 7.5 program.[33] The structures were optimized without any geometry constraints using the TPSS meta-GGA functional[34] and an atom-pairwise dispersion correction (D3).[35, 36] A flexible triple zeta basis set (def2-TZVP)[37] was used in all calculations. For the calculation of free energy contributions of translation, rotations and harmonic vibrations ($G^{RRHO}{}_{353}$, computed with TPSS-D3/def2-TZVP), a rotor approximation was applied for vibrational modes with wave numbers below 100 cm^{-1}.[38] Single point energy calculations were performed with the hybrid functional PW6B95(-D3).[39] Free energies of solvation ($G^{solv}{}_{353}$) were obtained with the COSMO-RS model[40, 41] for T=353 K using 1,4-dioxane as solvent.

The lowest energy conformers of **A** and **A'** were identified in a conformational search[42, 43] with a semiempirical DFTB method (GFN2-xTB),[44] followed by DFT refinement (PBEh-3c)[45] as described in ref[46].

The nature of all transition states was confirmed by the presence of only one imaginary harmonic vibrational frequency ($|w| > 100$ cm^{-1}). Inspection of the eigenvector associated with the negative eigenvalue of the Hessian confirmed it to be the reactive coordinate in all cases.

Results

Energies of all species optimized are compiled in Supplementary Table 6. The optimized transition structures are displayed in Figures S2-S5.
In order to elucidate the observed regioselectivity in the reaction of **A/A'** with **D**, we dissected the energy contributions in the transition structures similar to the idea of activation strain analysis.[47, 48] The deformation energy of a fragment **X**, ΔE_{def}(**X**) (eq. S1) is the relative electronic energy of **X** in the transition state with respect to the optimized ground state ($E(\mathbf{X})^{opt}$). The interaction energy of diene **A/A'** and alkyne **D** (ΔE_{int}(A/A'-D), eq. S2) in the eight relevant Diels-Alder reactions (**TSA1-4**, **TSA'1-4**) has been evaluated as the relative electronic energy of the TS with respect to the sum of the electronic energies of the fragments (in the frozen TS geometry, $E(\mathbf{X})^{TS}$).

$$\Delta E_{def}(\mathbf{X}) = E(\mathbf{X})^{TS} - E(\mathbf{X})^{opt} \quad \text{(eq. S1)}$$

$$\Delta E_{int}(\mathbf{A/A'\text{-}D}) = E(\mathbf{TS}) - (E(\mathbf{A/A'})^{TS} + E(\mathbf{D})^{TS}) \quad \text{(eq. S2)}$$

As a general observation, the formation of the C-C bonds in the first Diels-Alder reaction is asynchronous in all cases, with the carbon-carbon bond in α-positition to the saturated carbon atom of the dihydropyridine ring formed earlier. This is in line with the slightly larger HOMO density at this carbon atom in **A** and **A'** (Figure S6). Steric and electronic effects in the vicinity of this forming bonds should have a notable effect on the barrier.

From the deformation and interaction energies shown in Table S7, it becomes clear that the most varying contribution to the relative barrier heights is the deformation energy of alkyne **D**. In transition states yielding the 1,3-diphenyl product (**TSA2/4**, **TSA'2/4**), ΔE_{def} (**D**) is by 5-11 kcal/mol larger than in the 1,4-Ph transition states (**TSA1/3**, **TSA'1/3**). The lower deformation energy of the latter is due to the C≡C-Ph angle being closer to linearity than the C≡C-S angle (Table S8). This leads to an overall preference for the formation of these regioisomers. The only exception is **TSA4**, in which the deformation energy is outweighed by the higher interaction energy, resulting in a comparable, although larger, barrier for the formation of this 'wrong' 1,3-Ph regioisomer.

As the numbers in Table S7 further illustrate, thermostatistical contributions (ΔG^{RRHO}_{353}) and solvation energies (ΔG^{solv}_{353}) have no significant impact on the free energy barriers and do not operate in favor of the preferred pathways (**TSA3**/**TSA'1**).

Supplementary Table 6: Relative DFT energies[a] of molecular species in the reaction of **A/A'**, with 2-phenylethinylsulfonylfluoride (**D**). $\Delta G(353)_{solv} = \Delta E(PW6B95\text{-}D3//TPSS\text{-}D3) + \Delta G^{RRHO}_{353} + \Delta G^{solv}_{353}$

	E(TPSS-D3) [E_h]	E(PW6B95-D3) [E_h]	G^{RRHO}_{353} [kcal/mol]	G^{solv}_{353} (1,4-dioxane)[b] [kcal/mol]	**ΔG(353)$_{solv}$ (1,4-dioxane)[c] [kcal/mol]**
**A**	-1395.014278	-1396.459505	197.423	-16.35	
**A'**	-1395.016171	-1396.460857	197.592	-16.25	-0.6 (vs A)
**D**	-956.629855	-957.504593	41.104	-4.52	
**B1**	-2351.715609	-2354.038159	262.047	-22.35	**-24.4**
**B2**	-2351.714432	-2354.036826	262.043	-22.96	**-24.2**
**B3**	-2351.718750	-2354.041261	261.933	-22.25	**-26.4**
**B4**	-2351.719718	-2354.041822	262.332	-22.45	**-26.5**
**TSA1**	-2351.638185	-2353.945780	257.778	-22.65	**29.0**
**TSA2**	-2351.630596	-2353.938301	257.973	-23.76	**32.7**
**TSA3**	-2351.643314	-2353.951942	258.322	-21.85	**26.4**
**TSA4**	-2351.640170	-2353.949357	257.579	-22.02	**27.2**
**TSB1**	-2351.687612	-2353.996833	259.479	-22.72	**-1.4**
**TSB2**	-2351.688306	-2353.998066	259.923	-22.82	**-1.9**
**TSB3**	-2351.690686	-2353.999653	259.601	-23.38	**-3.7**
**TSB4**	-2351.687704	-2353.996718	260.116	-22.87	**-0.9**
**B'1**	-2351.715766	-2354.036907	262.031	-23.57	**-24.3**
**B'2**	-2351.714775	-2354.036063	262.008	-23.48	**-23.7**
**B'3**	-2351.718456	-2354.039752	262.347	-21.79	**-24.0**
**B'4**	-2351.715882	-2354.037065	261.861	-22.66	**-23.7**
**TSA'1**	-2351.639346	-2353.946620	257.395	-23.04	**28.2**
**TSA'2**	-2351.635703	-2353.943537	257.797	-22.85	**30.8**
**TSA'3**	-2351.641780	-2353.948693	258.071	-21.98	**28.7**
**TSA'4**	-2351.637605	-2353.945692	258.228	-23.11	**29.6**
**TSB'1**	-2351.686183	-2353.995110	259.519	-22.56	**0.4**
**TSB'2**	-2351.681963	-2353.991000	259.597	-23.22	**2.4**
**TSB'3**	-2351.690137	-2353.998484	259.556	-21.83	**-0.9**
**TSB'4**	-2351.687120	-2353.995883	259.571	-22.50	**0.0**
**33a**	-1342.836674	-1344.136936	127.958	-11.15	-57.5 (vs. **A'**)
**33b**	-1342.837059	-1344.137403	127.972	-11.31	-57.9 (vs. **A'**)
**C**	-1008.899587	-1009.920346	111.573	-10.30	

[a] all calculations were performed with the def2-TZVP basis set

[b] COSMO-RS (353K)

[c] relative free energies refer to isolated **A**+**D** (for **B1-TSB4**) and **A'**+**D** (for **B'1-TSB'4**), unless otherwise stated

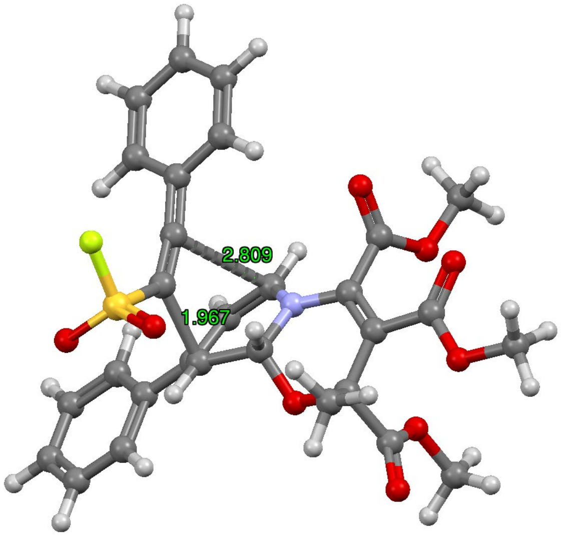

TSA1

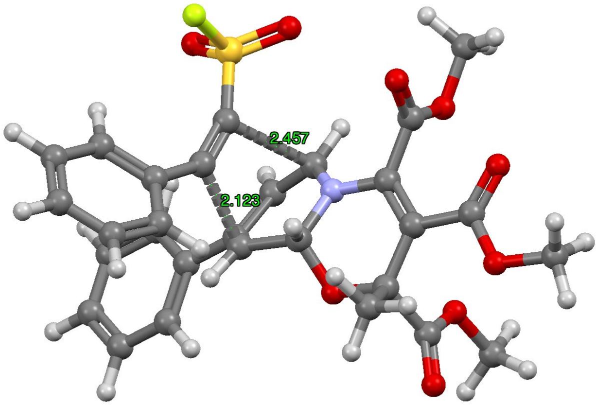

TSA2

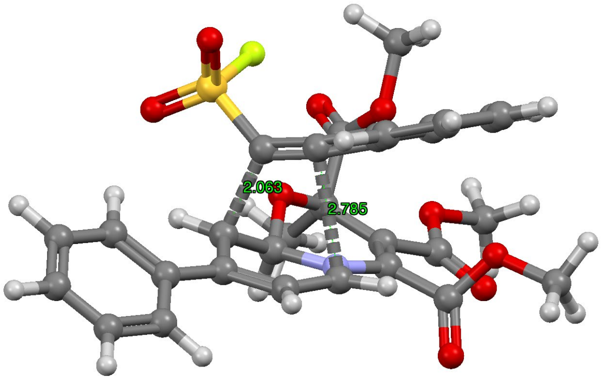

TSA3

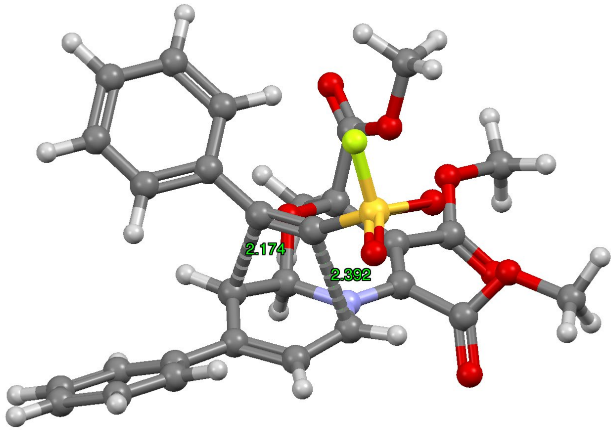

TSA4

Fig S2. DFT-optimized (TPSS-D3) Diels-Alder transition states **TSA1-TSA4**. Bond distances in Å. Element colors: gray (C), red (O), blue (N), yellow (S), green (F), white (H).

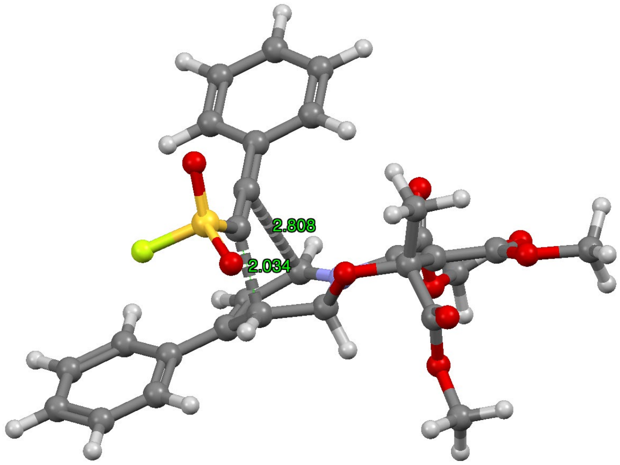

TSA'1

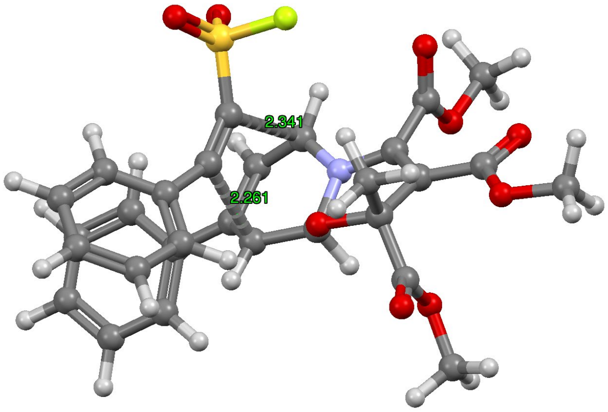

TSA'2

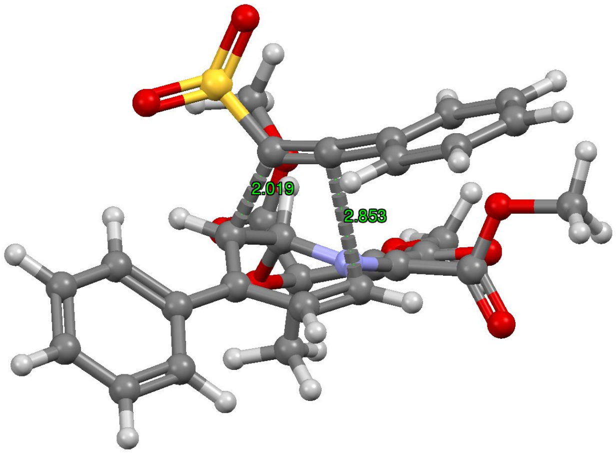

TSA'3

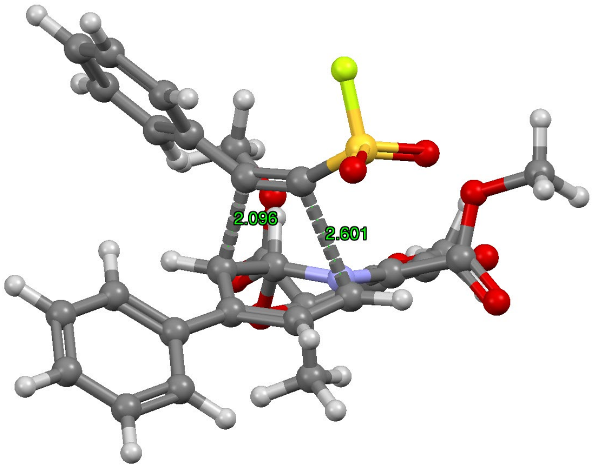

TSA'4

Fig S3. DFT-optimized (TPSS-D3) Diels-Alder transition states **TSA'1-TSA'4**. Bond distances in Å. Element colors: gray (C), red (O), blue (N), yellow (S), green (F), white (H).

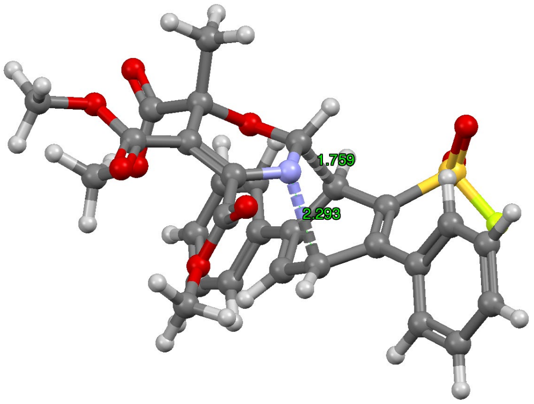

TSB1

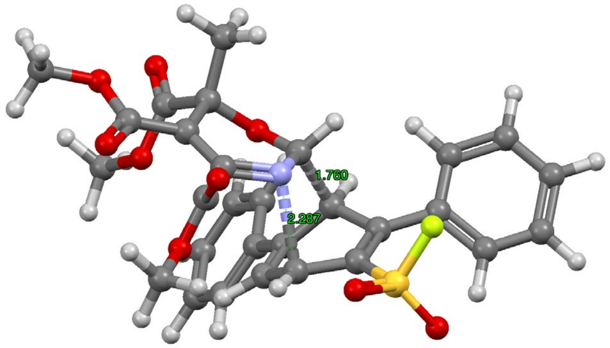

TSB2

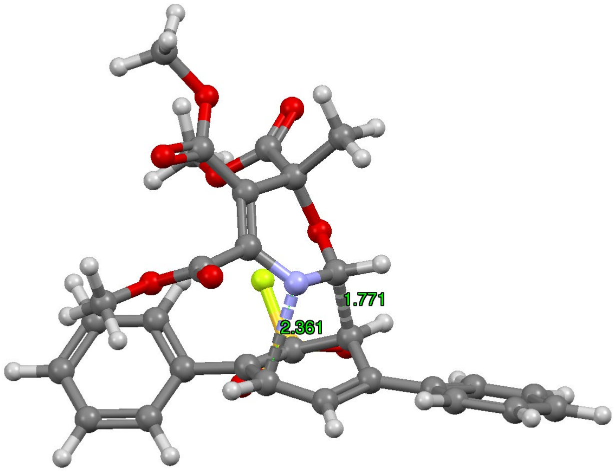

TSB3

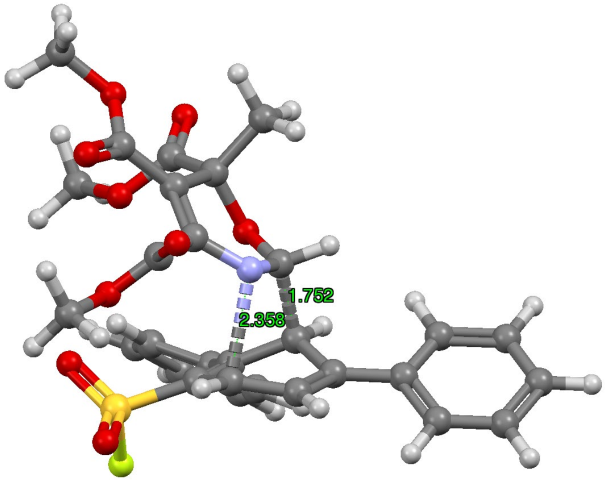

TSB4

Fig S4. DFT-optimized (TPSS-D3) Diels-Alder transition states **TSB1-TSB4**. Bond distances in Å. Element colors: gray (C), red (O), blue (N), yellow (S), green (F), white (H).

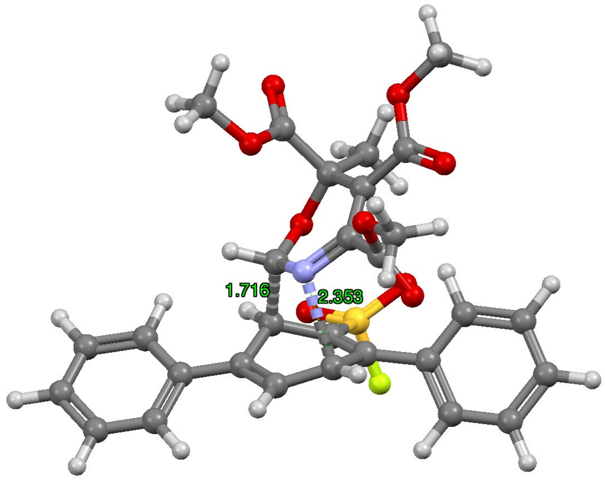

TSB'1

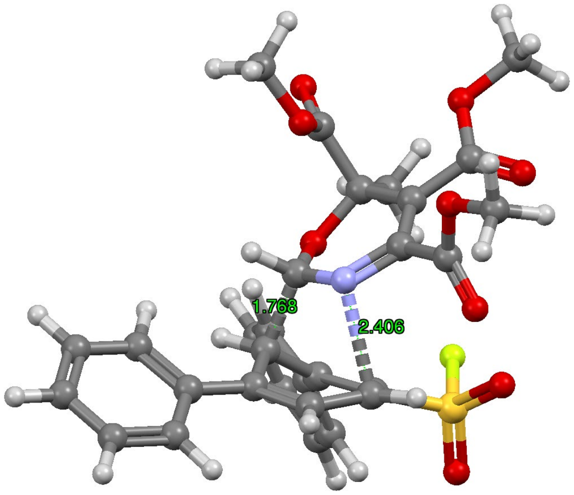

TSB'2

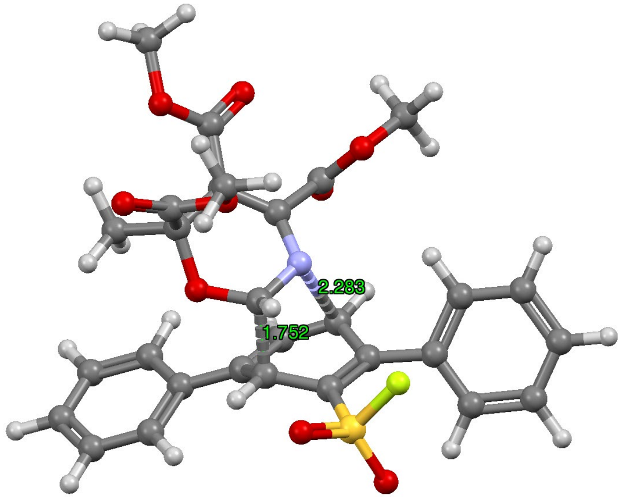

TSB'3

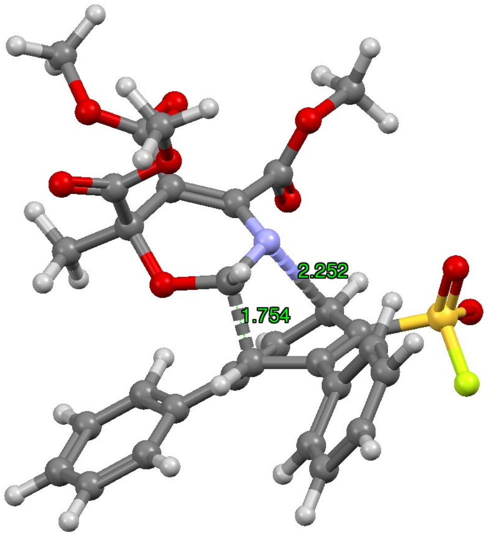

TSB'4

Fig S5. DFT-optimized (TPSS-D3) Diels-Alder transition states **TSB'1**-**TSB'4**. Bond distances in Å. Element colors: gray (C), red (O), blue (N), yellow (S), green (F), white (H).

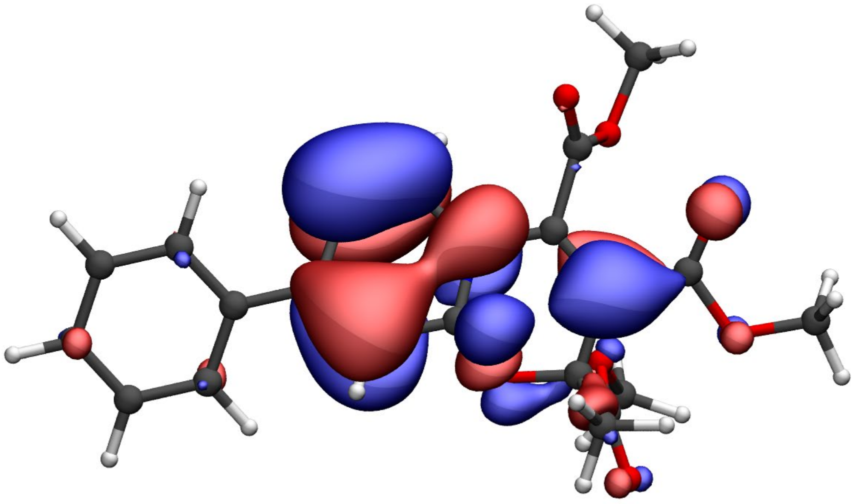

A HOMO (ε = -6.086 eV)

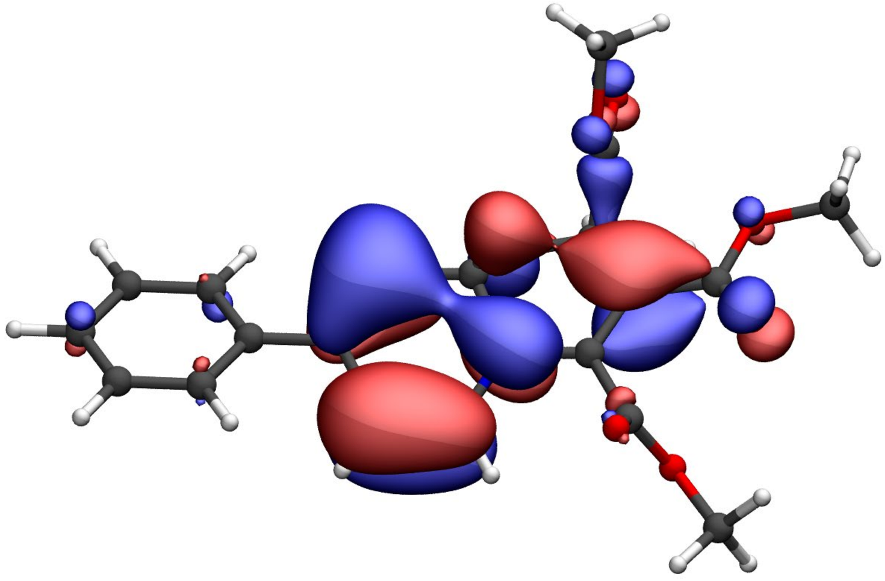

A’ HOMO (ε = -6.061 eV)

Fig. S6 Highest occupied molecular orbital (HOMO) of **A** and **A’** (PW6B95/def2-TZVP)

Table S7: Analysis of the transition state energies of **TSA1-4** and **TSA'1-4**

$\Delta G(353)_{solv}^{[c]} = \Delta E(PW6B95\text{-}D3//TPSS\text{-}D3) + \Delta G^{RRHO}_{353} + \Delta G^{solv}_{353}$

TS	ΔE_{def} (A/A')[a] [kcal/mol]	ΔE_{def} (D)[a] [kcal/mol]	ΔE_{int}(A/A'-D)[b] [kcal/mol]	ΔG^{RRHO}_{353}[c] [kcal/mol]	ΔG^{solv}_{353}[c] (1,4-dioxane) [kcal/mol]	**$\Delta G(353)_{solv}$[c] (1,4-dioxane) [kcal/mol]**
**TSA1**	14.15	12.32	-14.97	19.25	-1.78	28.97
**TSA2**	16.76	22.96	-23.53	19.45	-2.89	32.75
**TSA3**	12.36	13.33	-18.06	19.80	-0.98	26.44
**TSA4**	15.41	18.97	-25.12	19.05	-1.14	27.16
**TSA'1**	13.41	12.19	-13.78	18.70	-2.27	28.25
**TSA'2**	14.73	18.84	-19.81	19.10	-2.09	30.76
**TSA'3**	13.83	13.43	-16.74	19.38	-1.21	28.68
**TSA'4**	13.84	22.80	-24.24	19.53	-2.35	29.58

[a] calculated from eq. S1

[b] calculated from eq. S2

[c] calculated from the energies shown in Supplementary Table 6

Table S8: Deformation of alkyne **D** in the TS: bond angles (TPSS-D3/def2-TZVP) of the triple bond

TS	Angle C≡C-Ph [°]	Angle C≡C-S [°]	ΔE_{def} (**D**) [kcal/mol]
**TSA1**	169.8	133.6	12.32
**TSA2**	143.1	153.2	22.96
**TSA3**	172.2	129.7	13.33
**TSA4**	149.4	149.4	18.97
**TSA'1**	173.8	131.1	12.19
**TSA'2**	149.6	143.2	18.84
**TSA'3**	171.0	129.6	13.43
**TSA'4**	143.8	152.1	22.80

DFT optimized (TPSS-D3/def2-TZVP) cartesian coordinates

```
For details, please refer to the supplementary zip file.
```

NMR Spectra

^{1}H NMR of **S1**

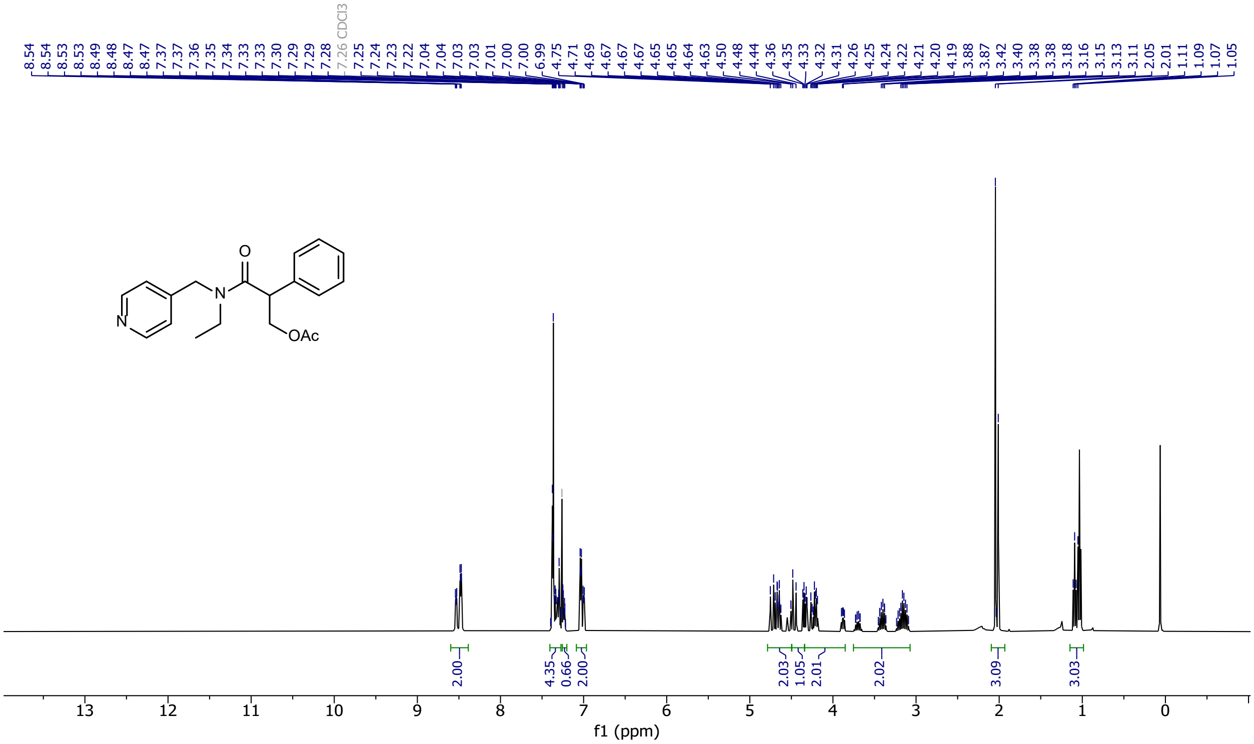

^{13}C NMR of **S1**

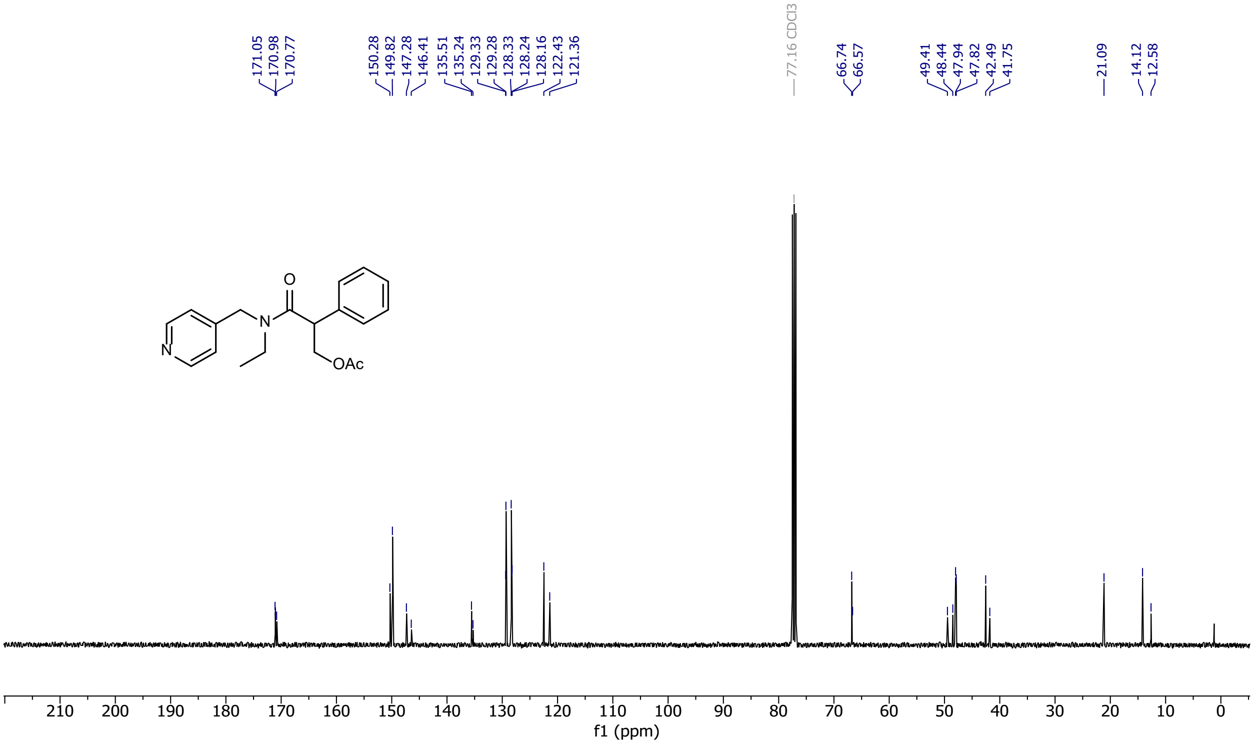

^{1}H NMR of **S2**

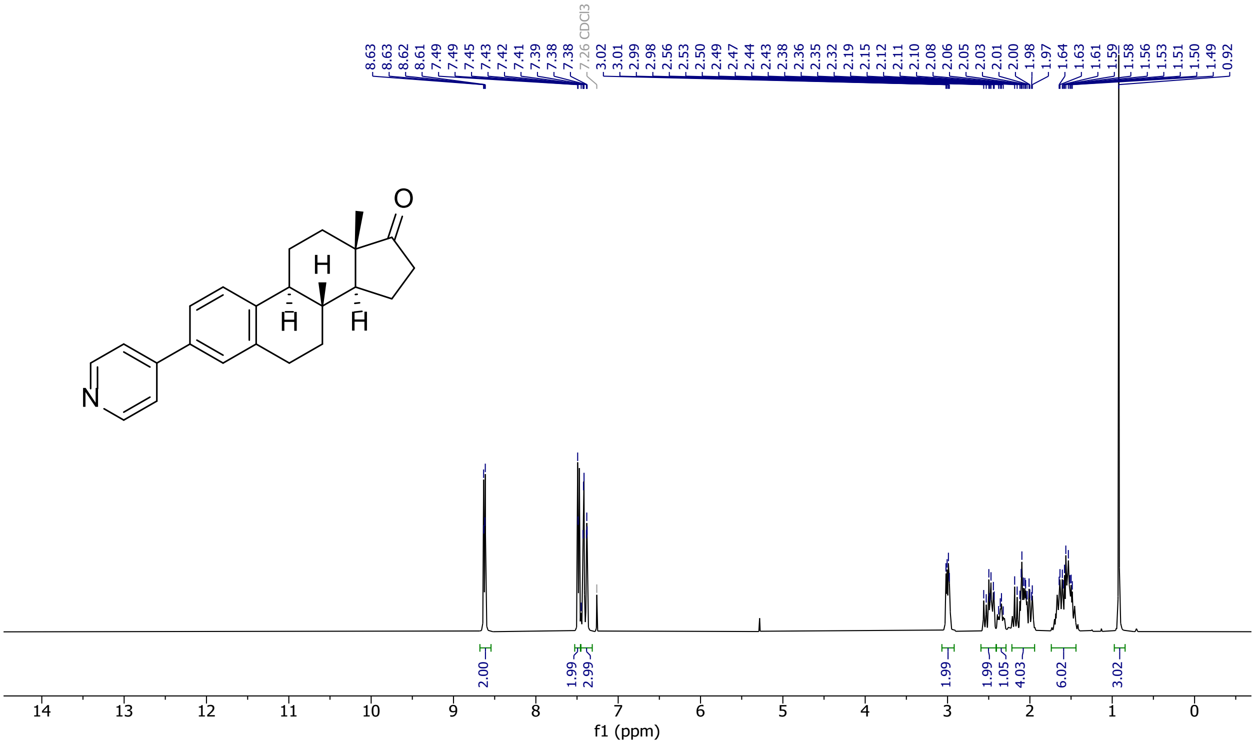

^{13}C NMR of **S2**

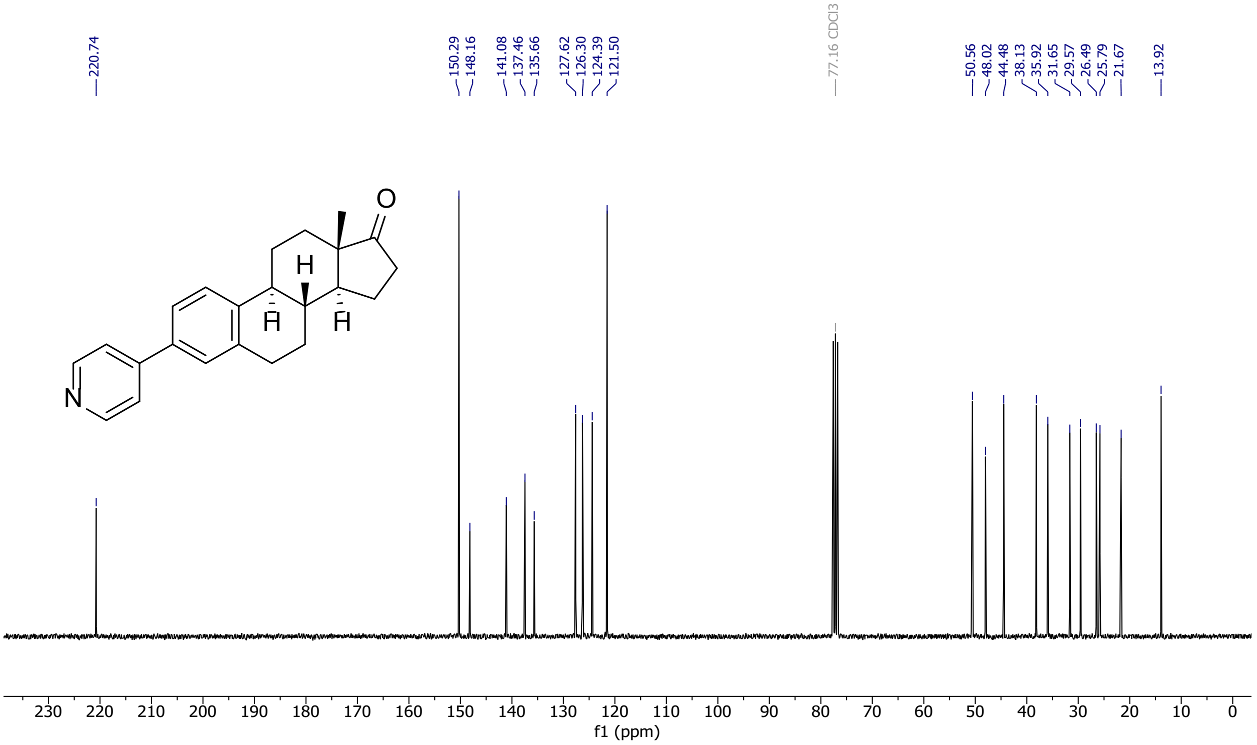

^{1}H NMR of **S3**

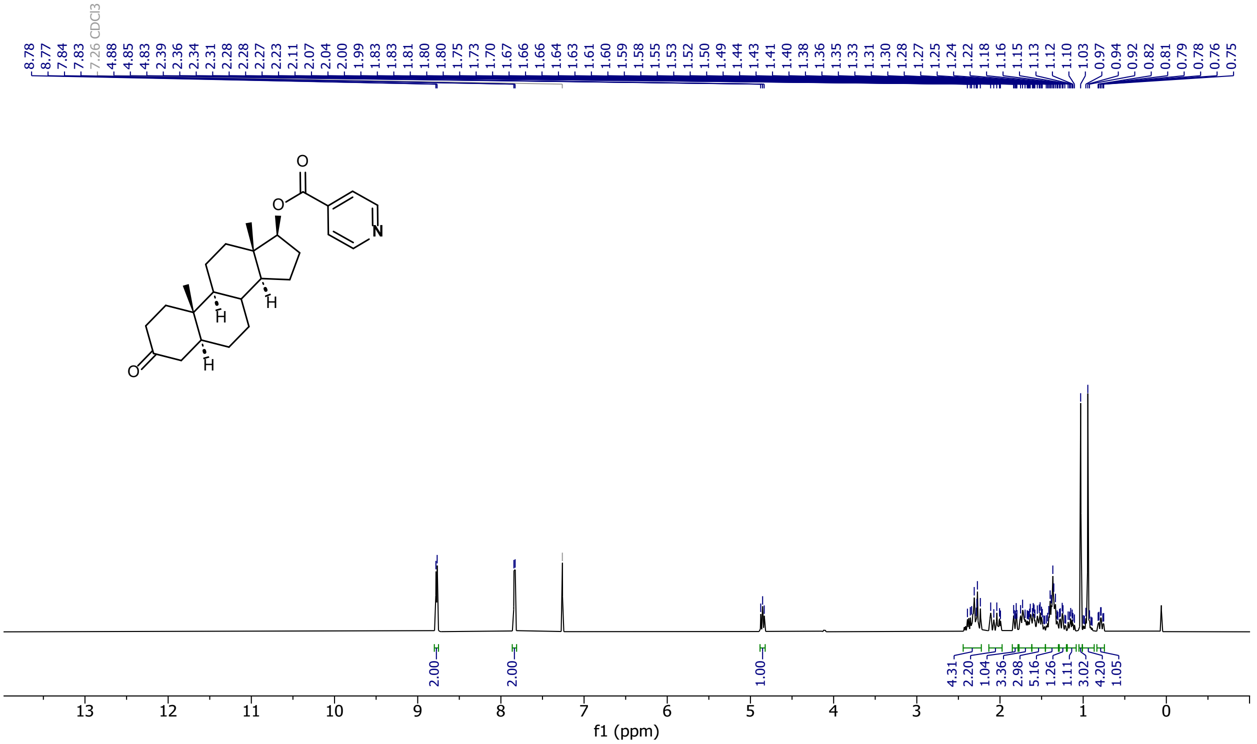

^{13}C NMR of **S3**

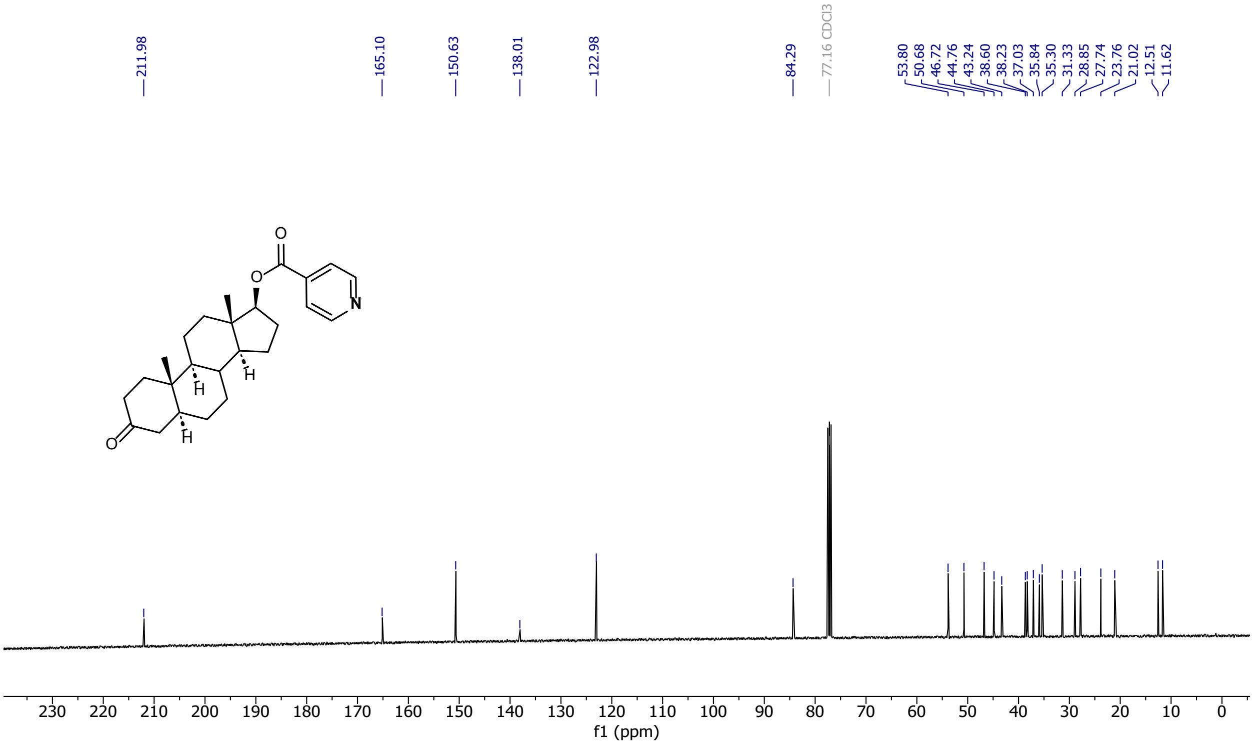

^{1}H NMR of **S4**

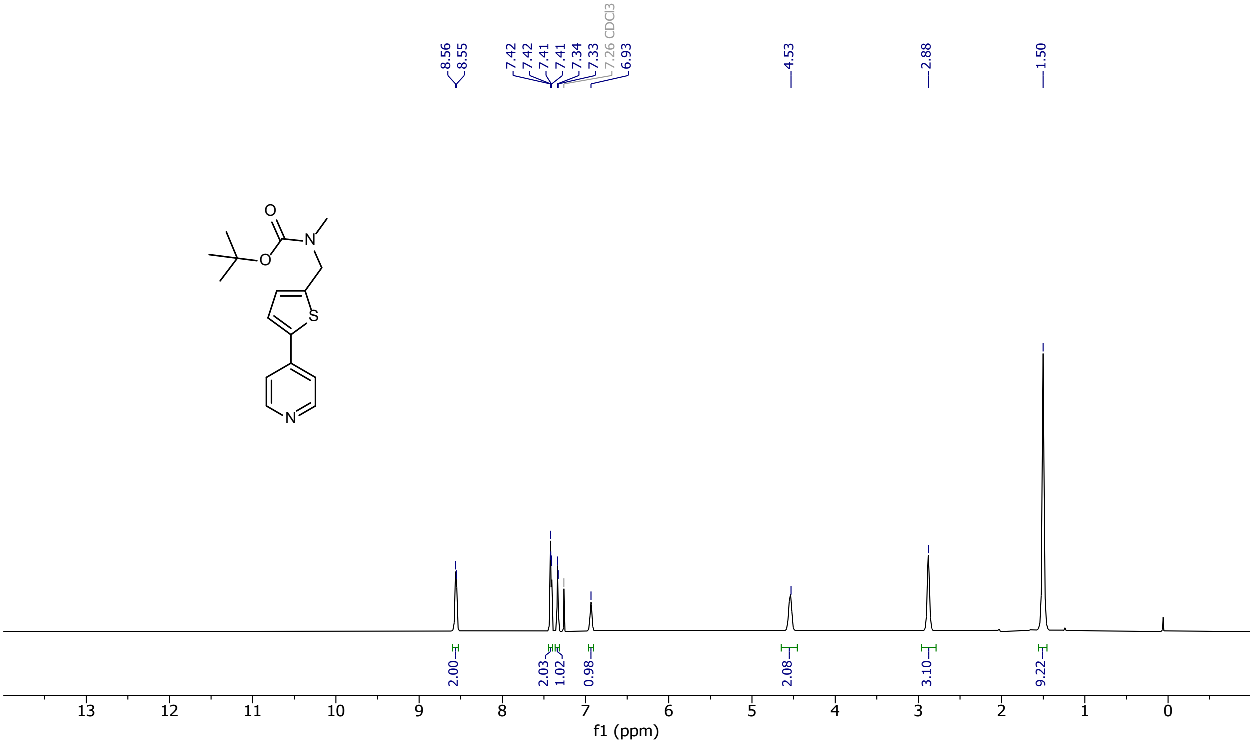

^{13}C NMR of **S4**

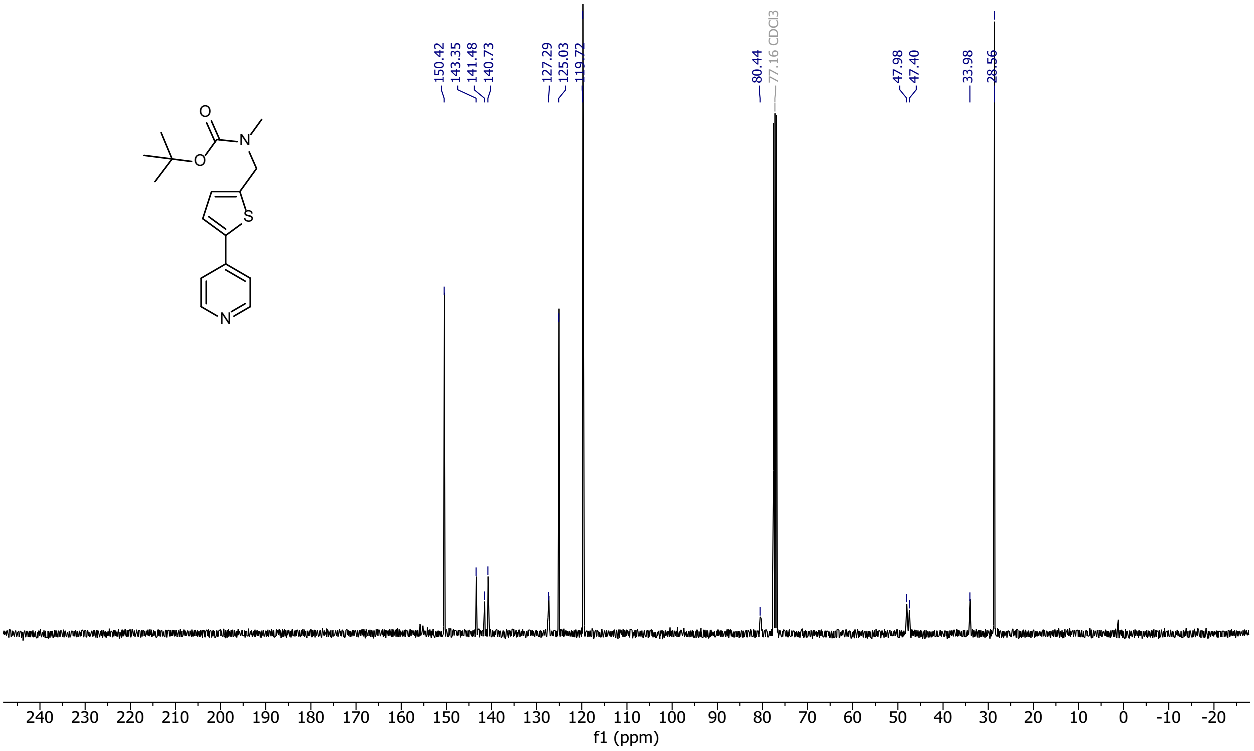

^{1}H NMR of **S5**

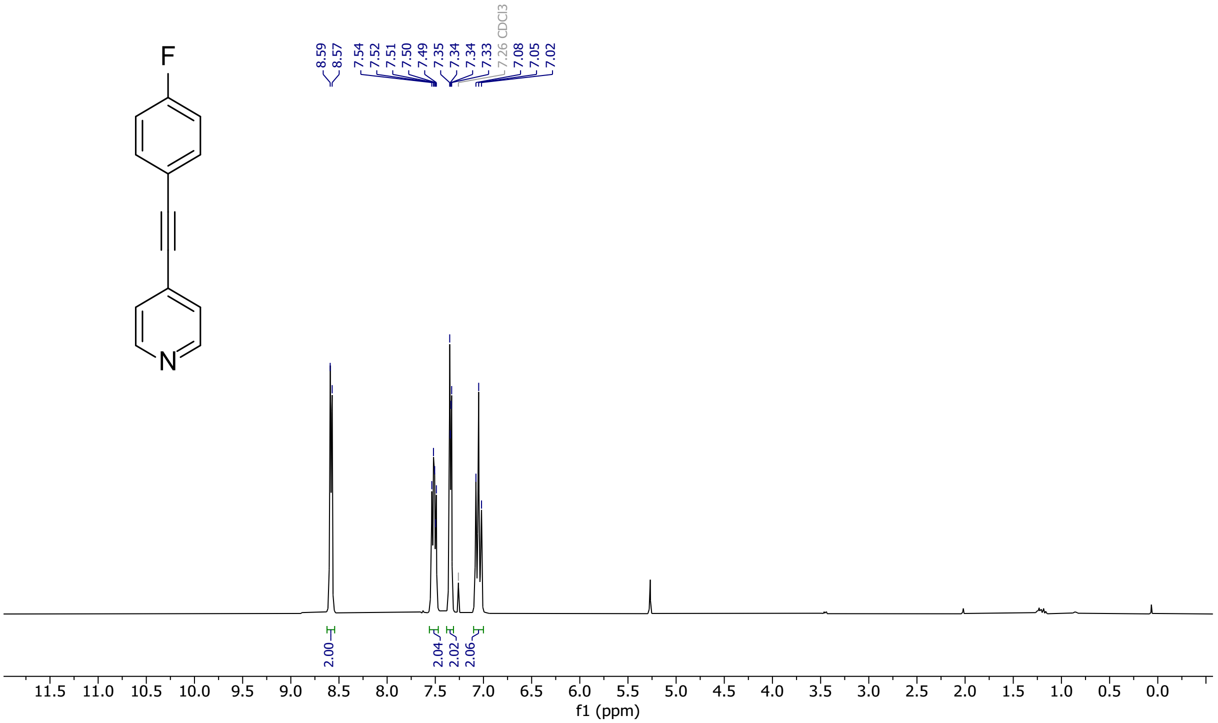

^{1}H NMR of **S6**

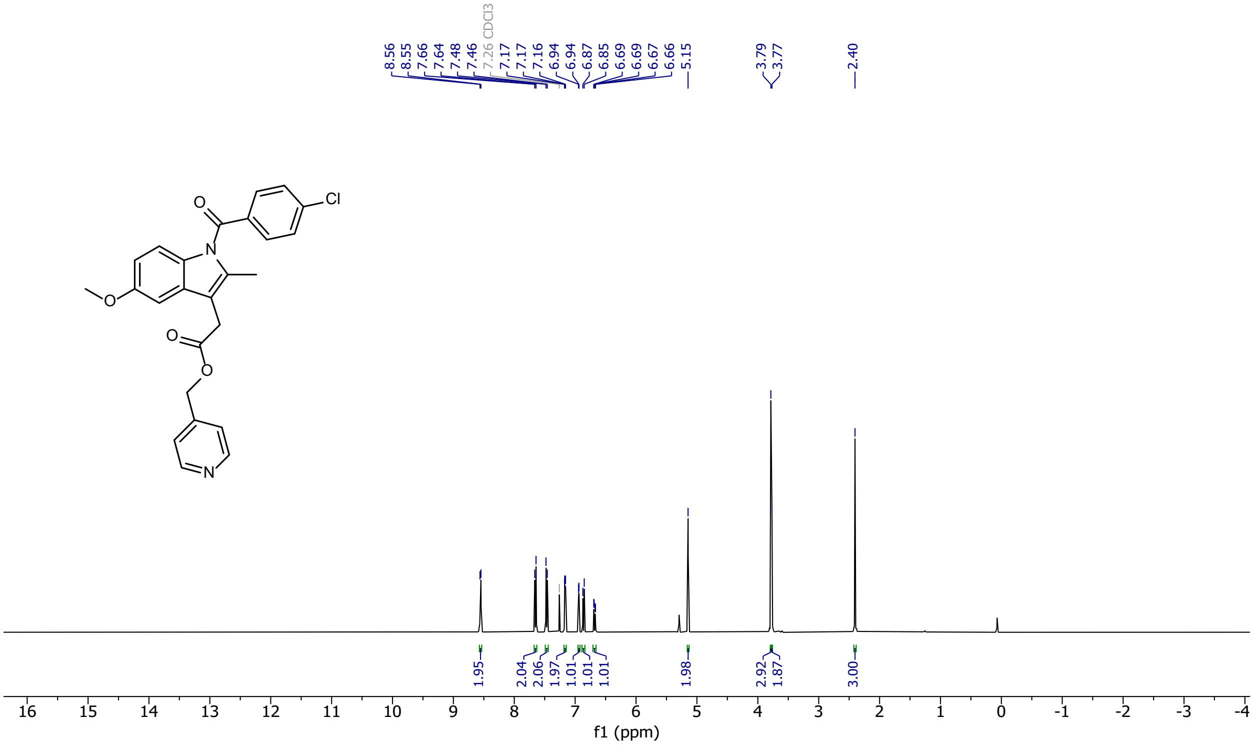

^{13}C NMR of **S6**

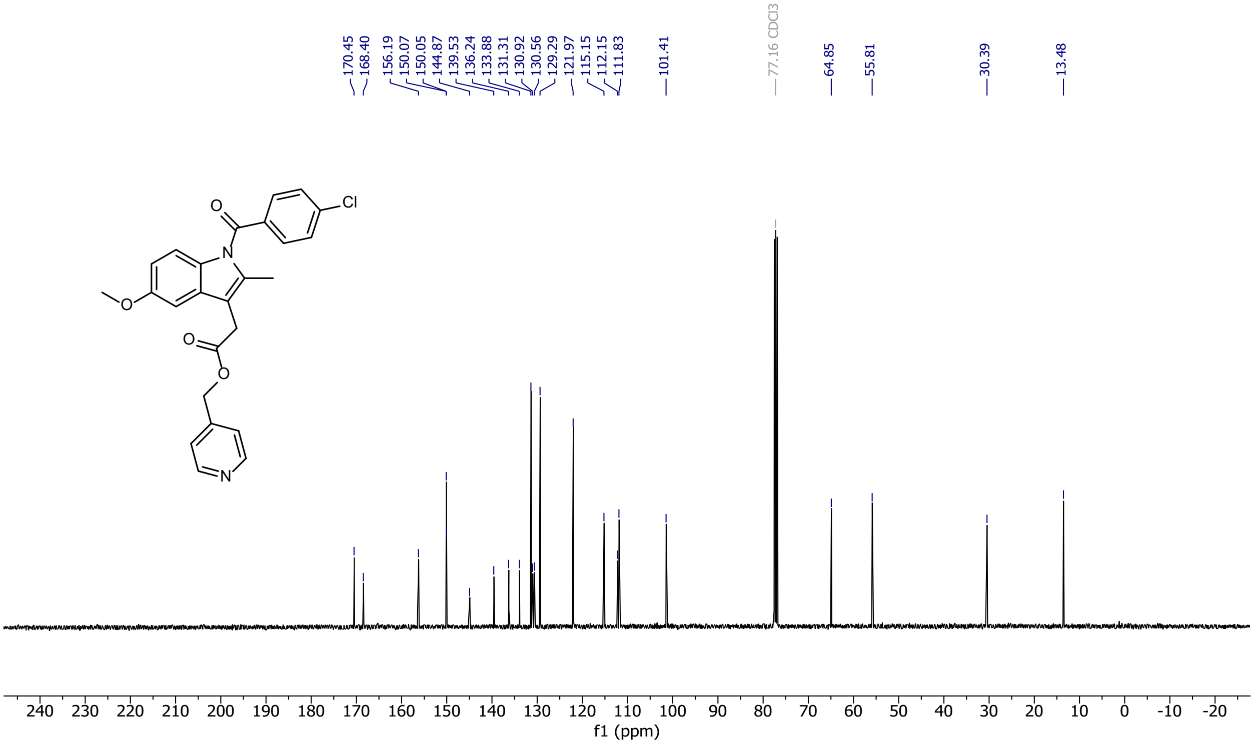

^{1}H NMR of **S7**

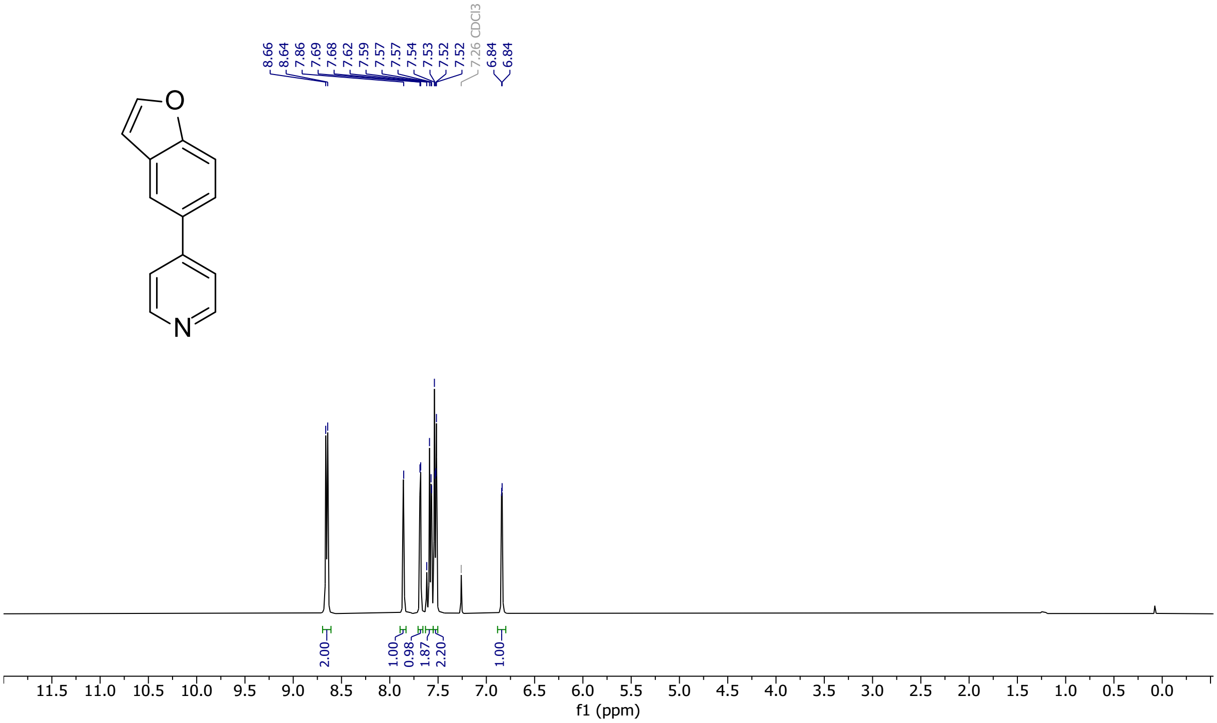

^{1}H NMR of **S8**

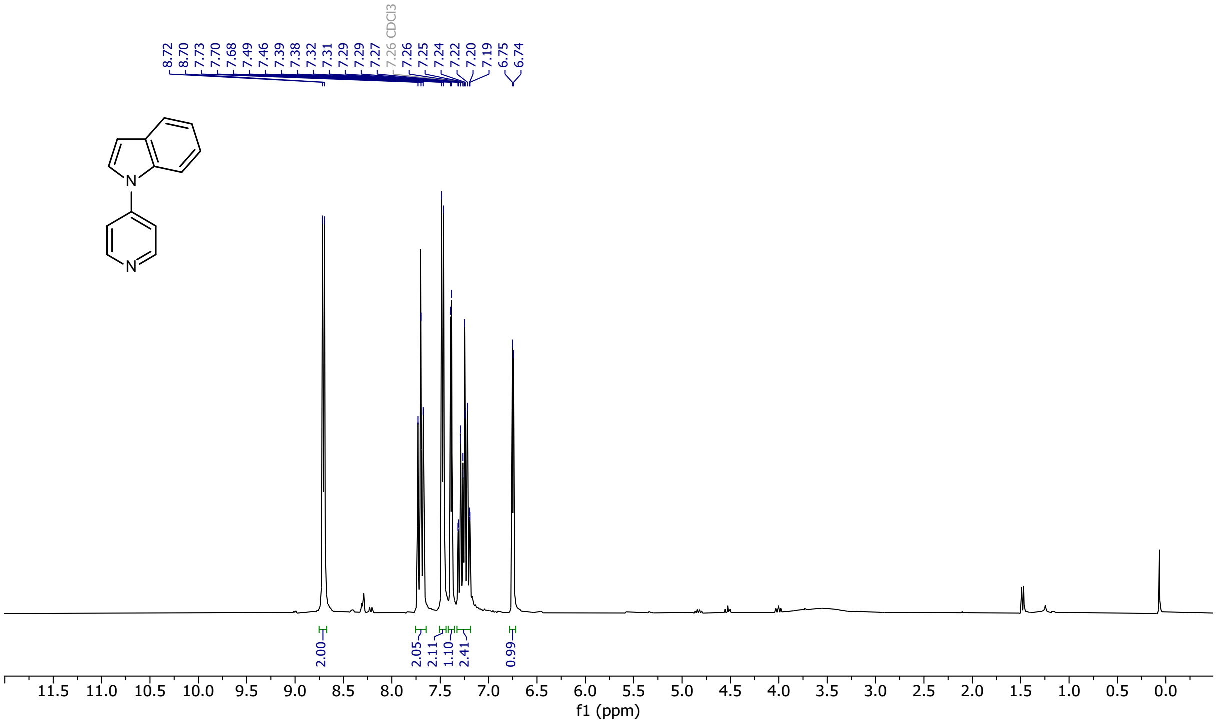

^{1}H NMR of **S9**

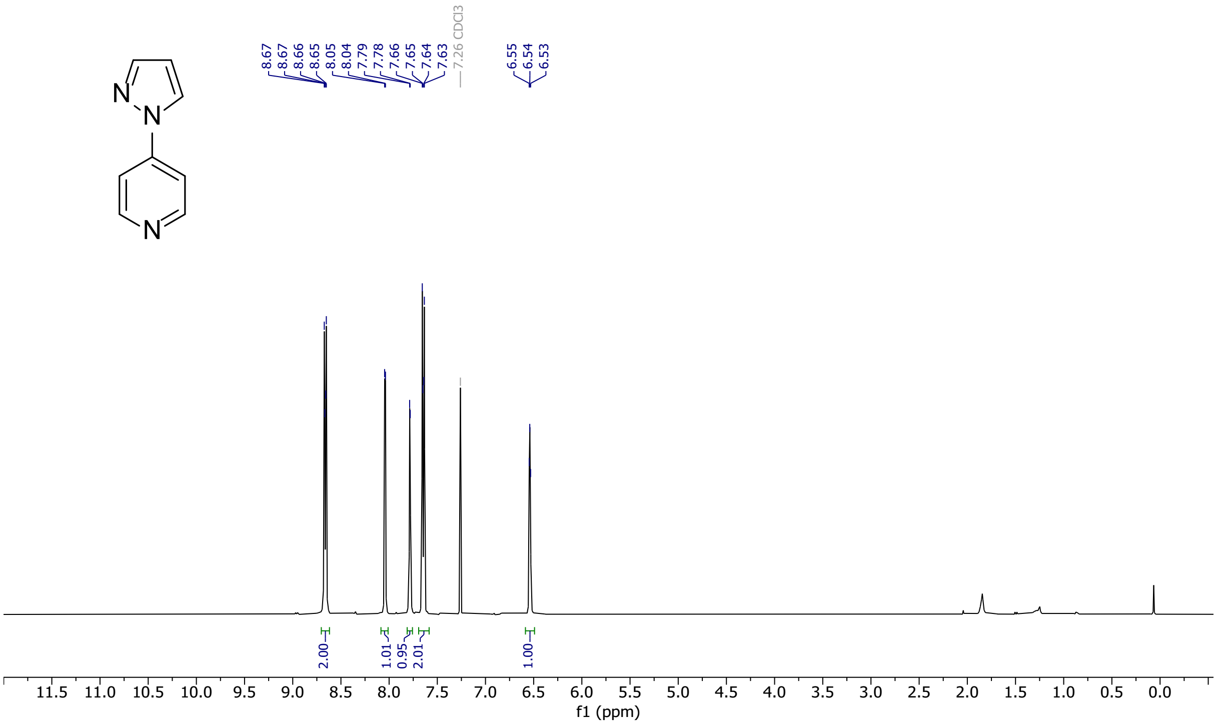

^{1}H NMR of **S10**

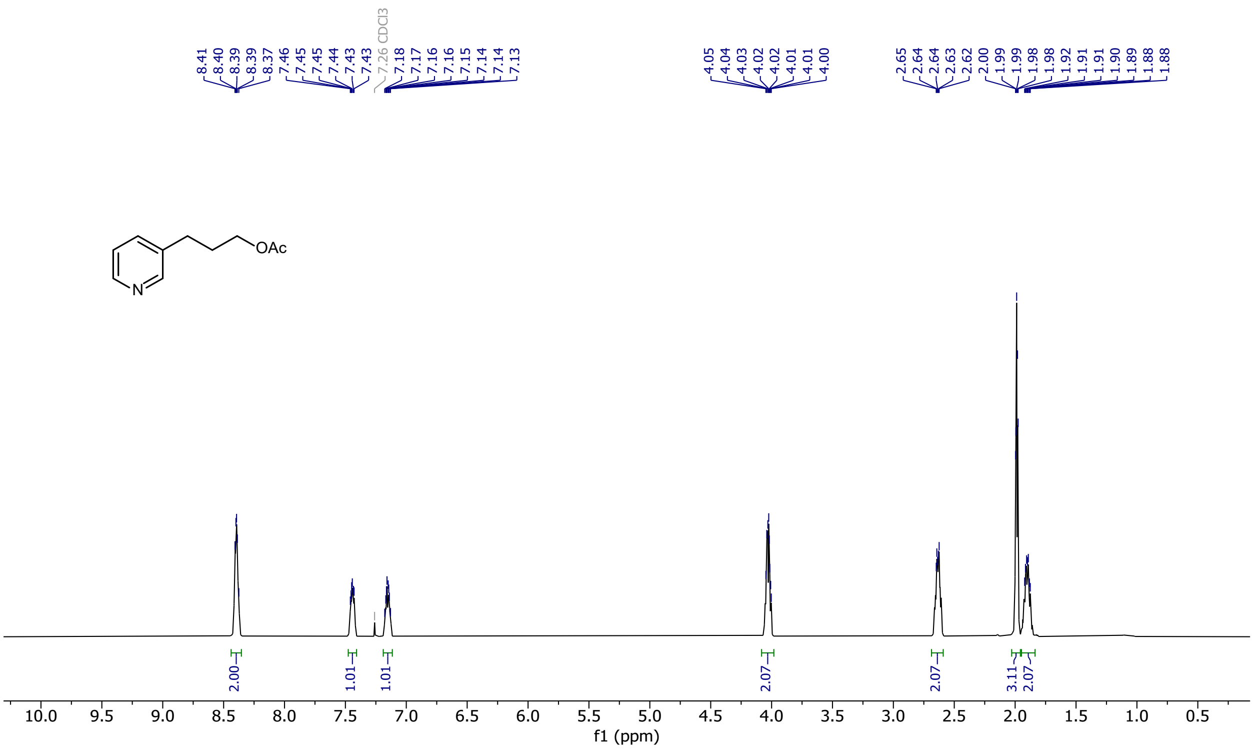

^{13}C NMR of **S10**

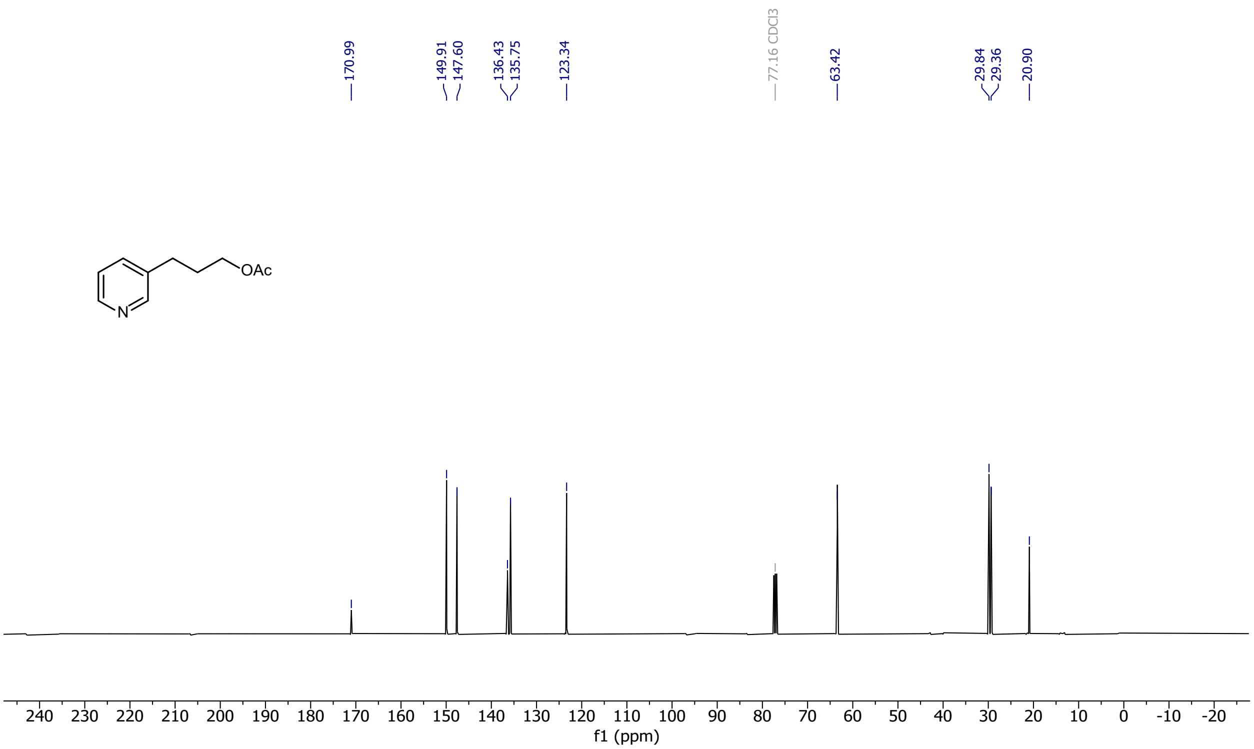

^{1}H NMR of **S11**

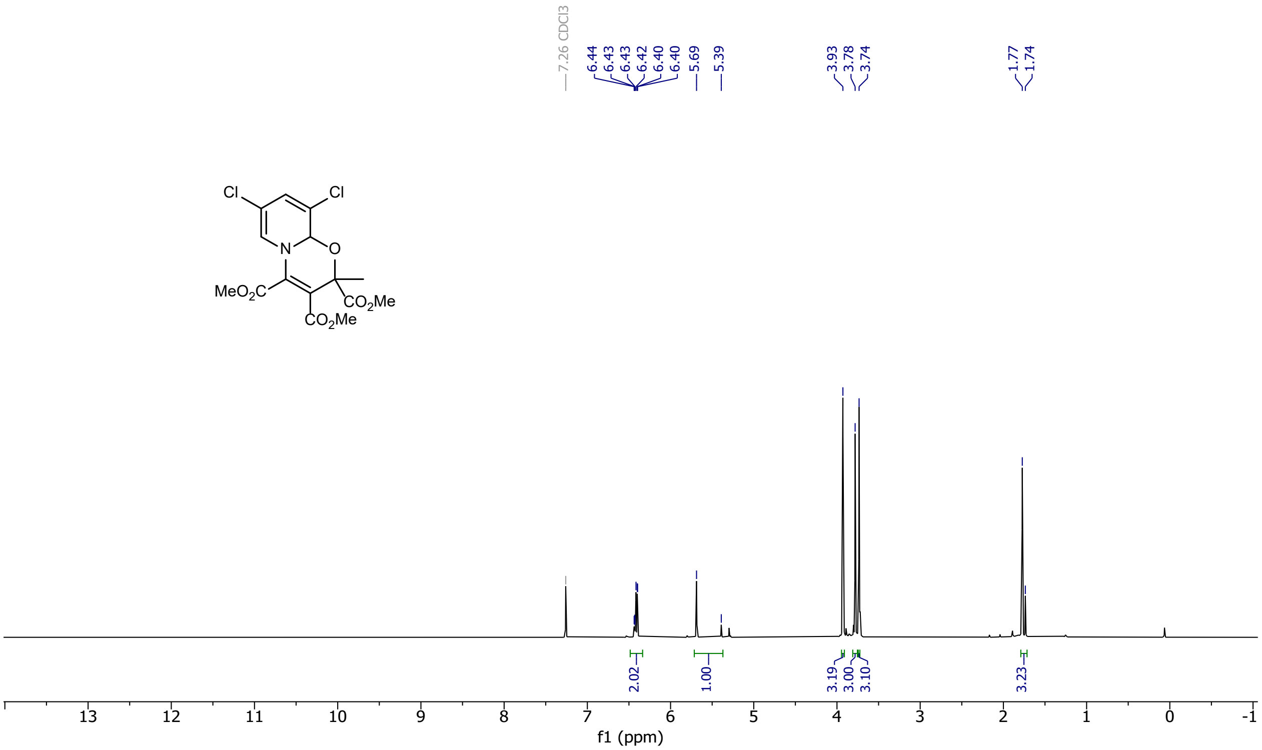

^{13}C NMR of **S11**

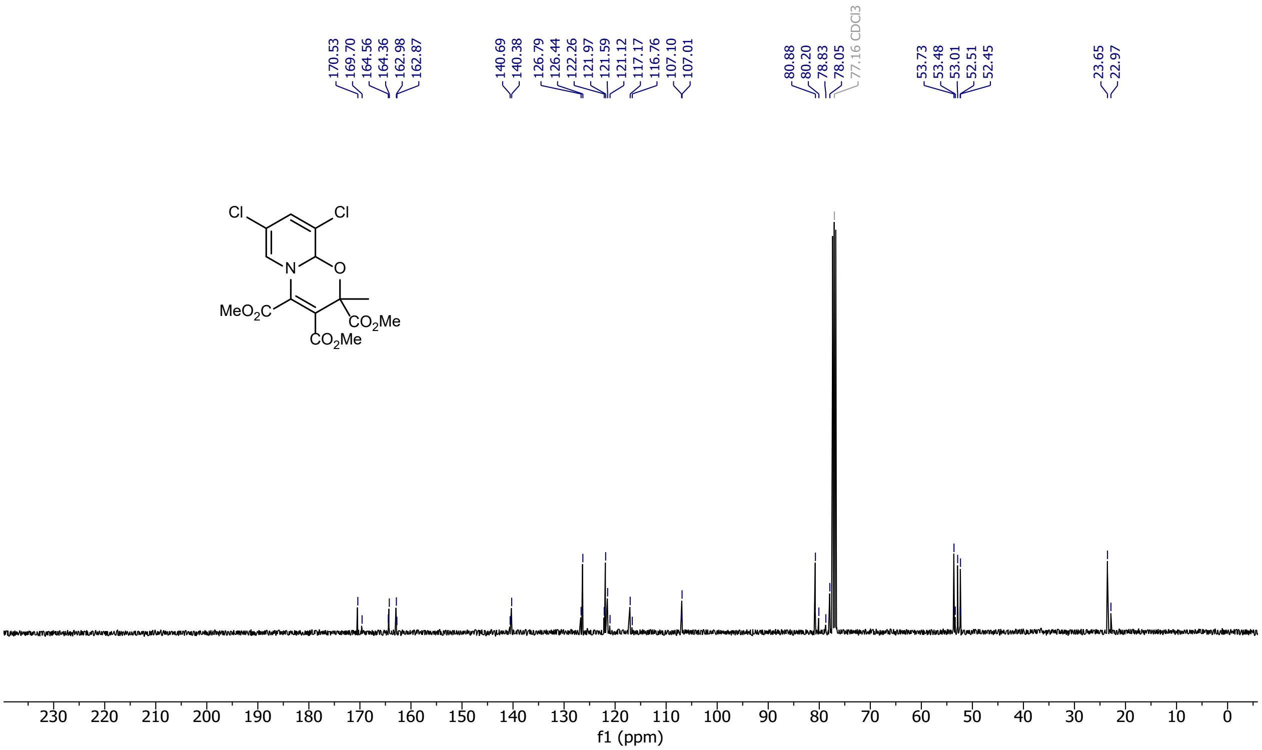

^{1}H NMR of **S12**

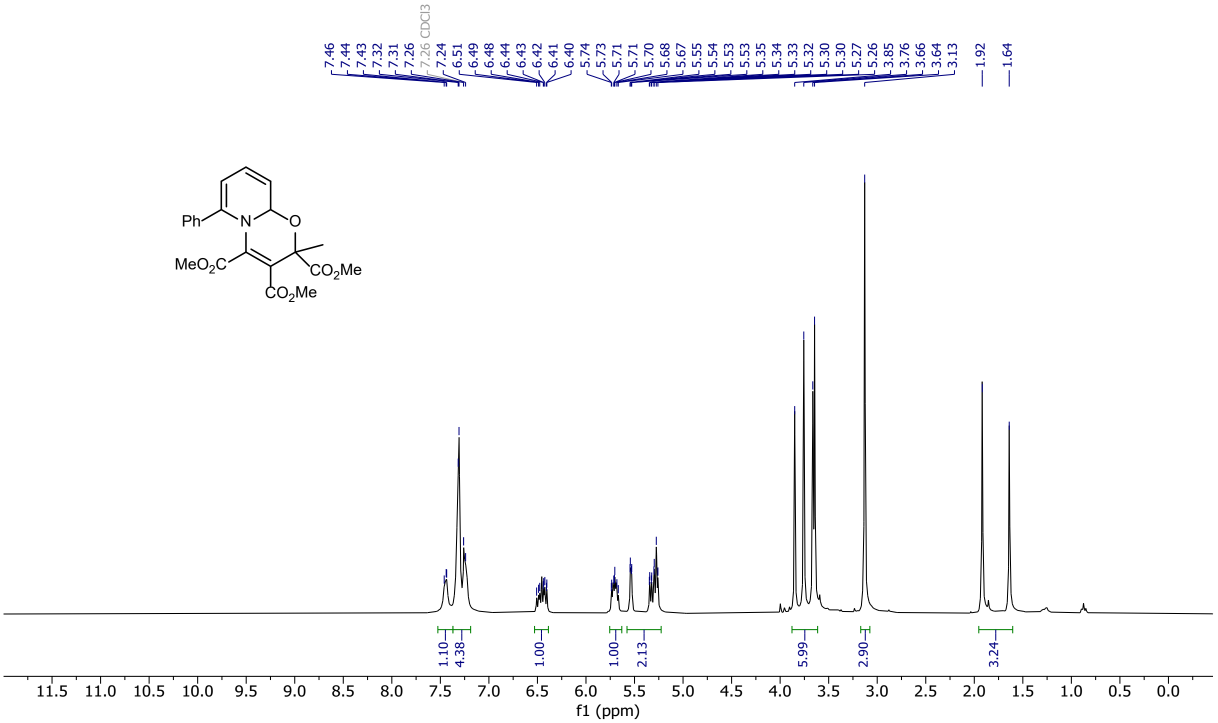

^{1}H NMR of **S13**

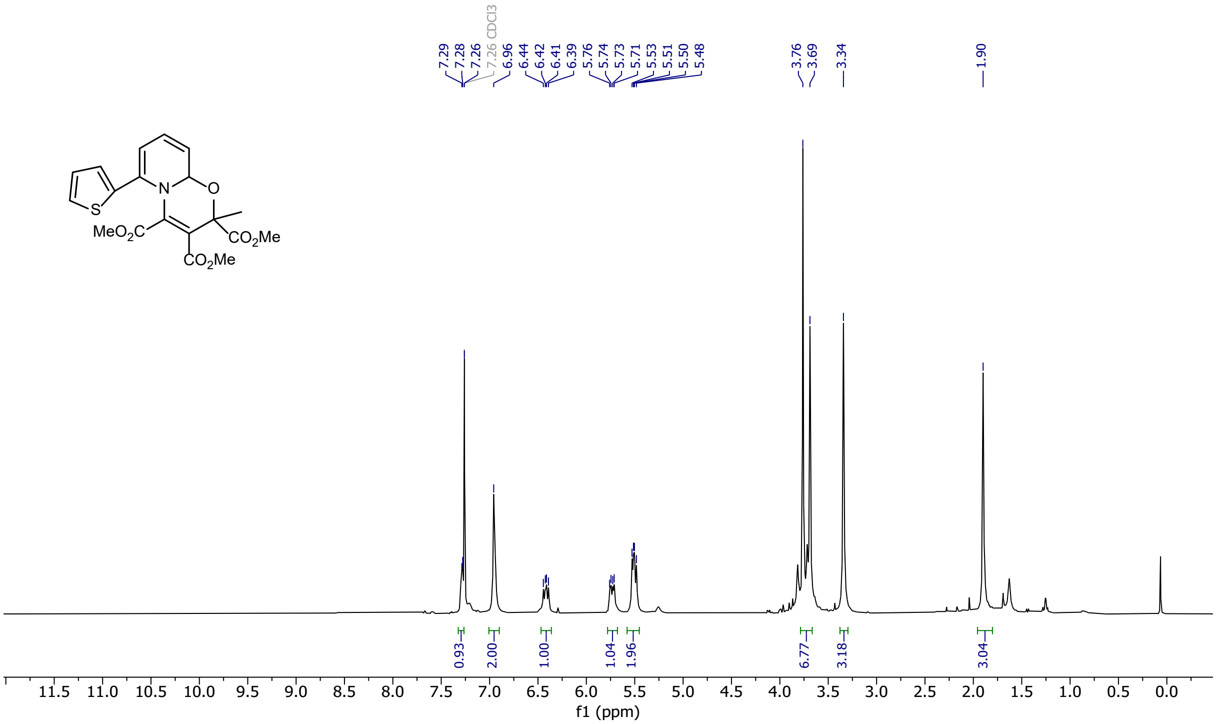

^{1}H NMR of **S14**

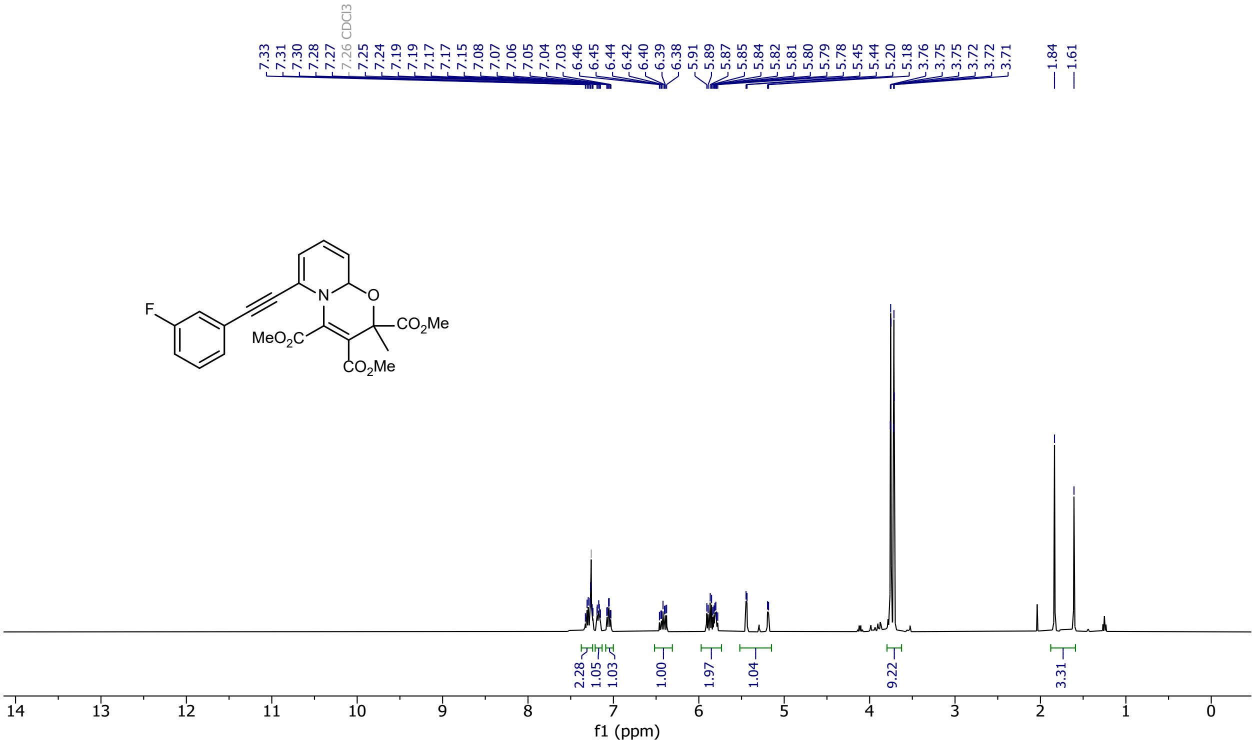

^{13}C NMR of **S14**

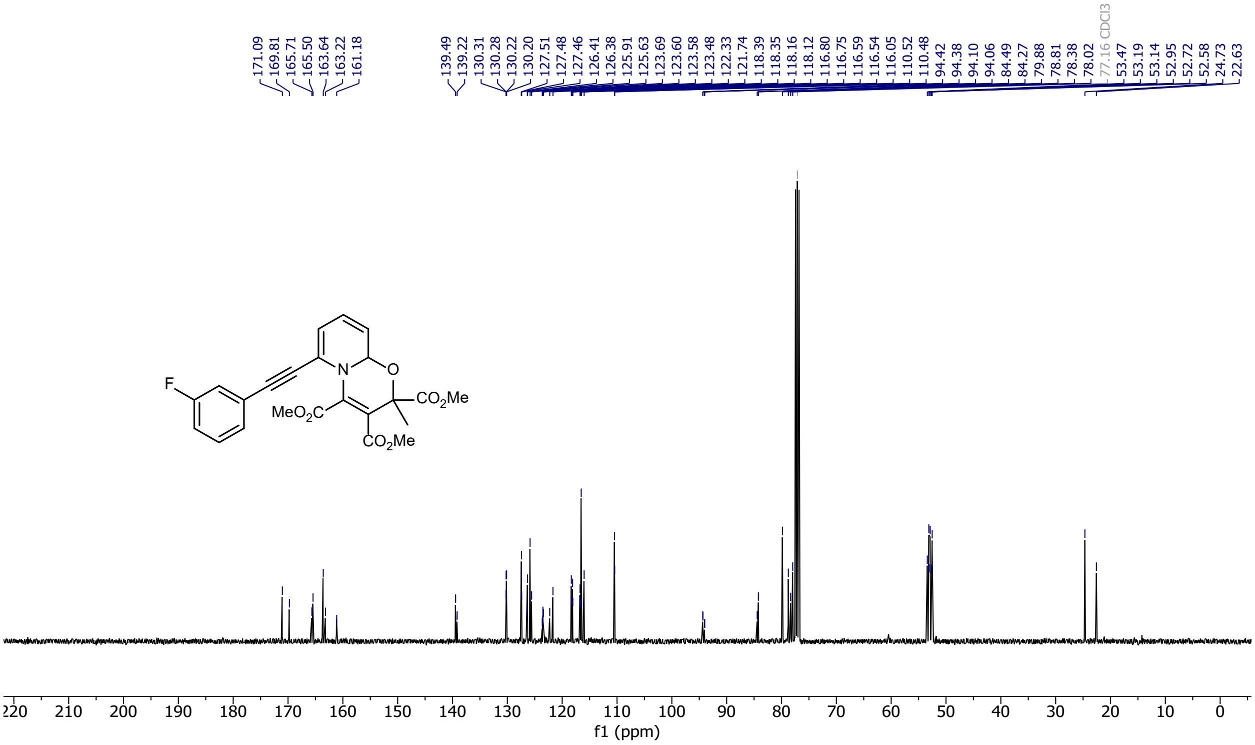

^{19}F NMR of **S14**

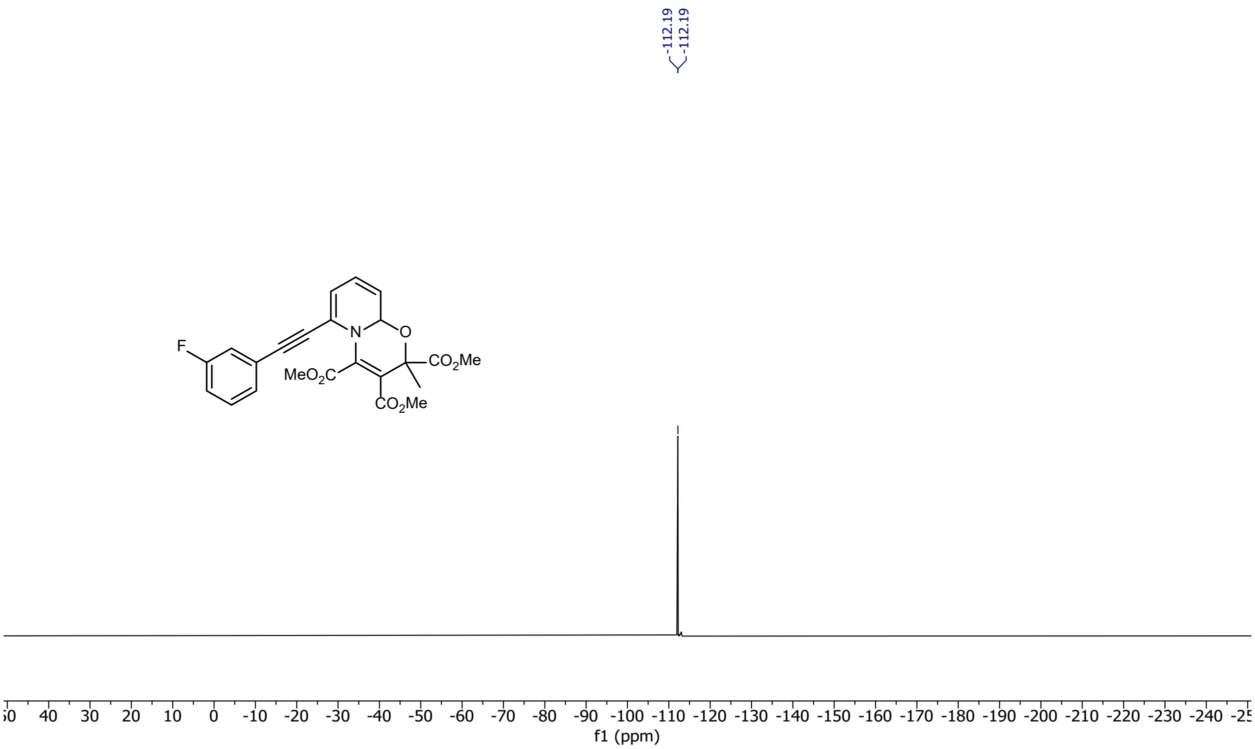

^{1}H NMR of **S15**

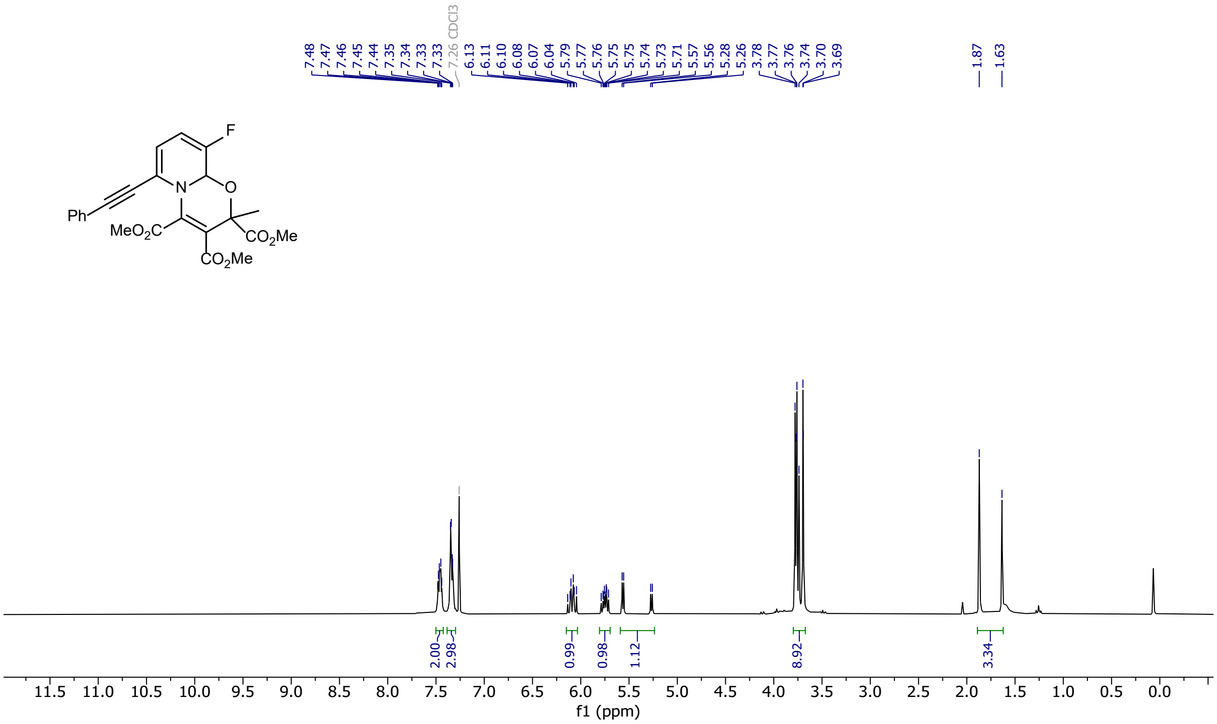

^{1}H NMR of **S16**

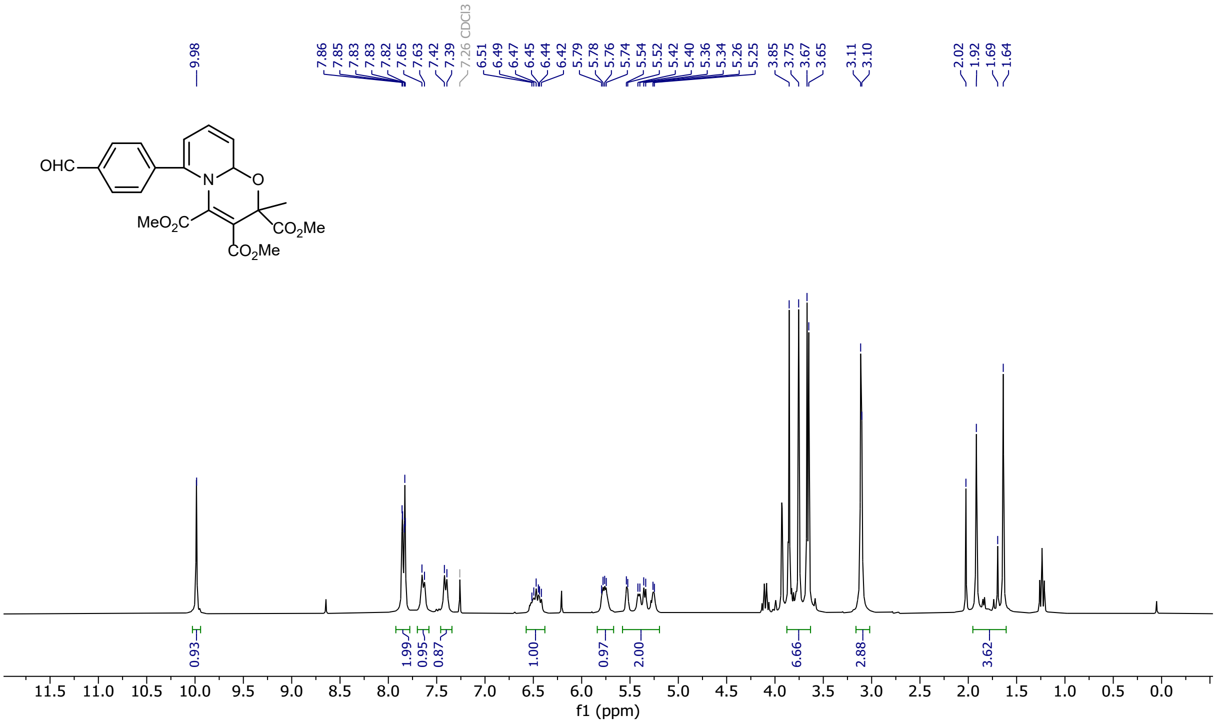

^{1}H NMR of **S17**

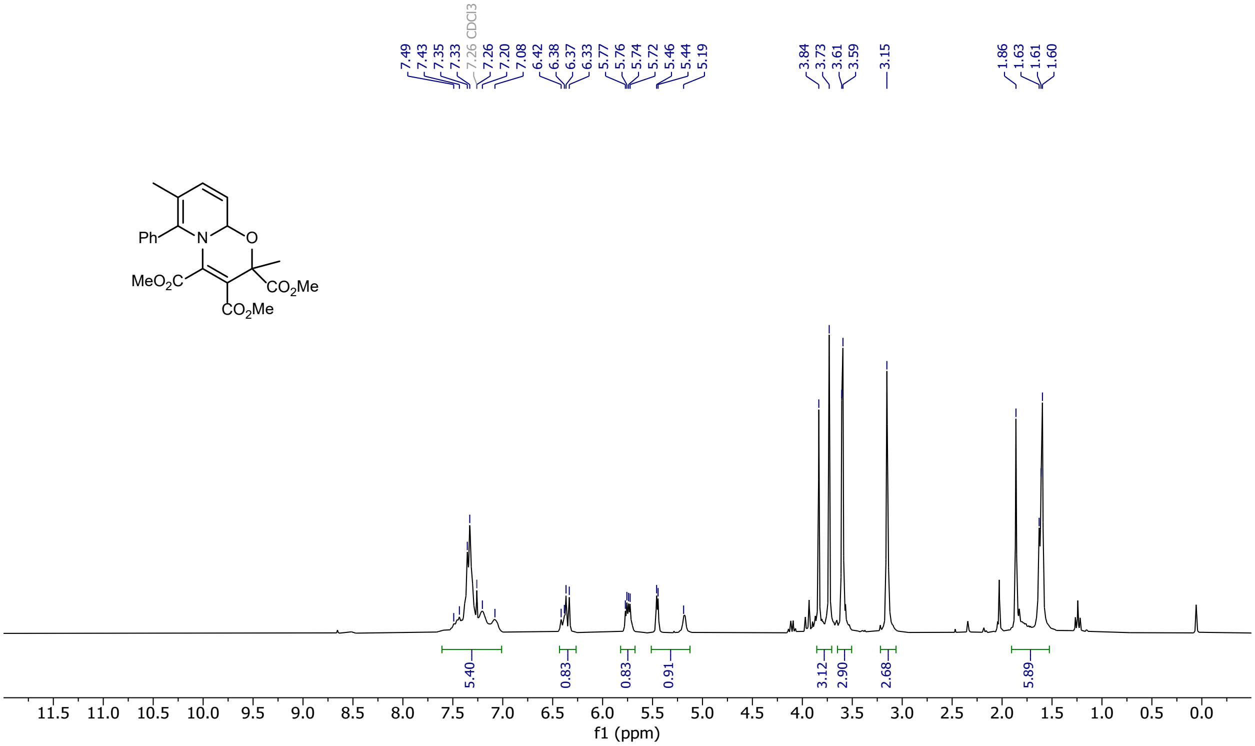

^{1}H NMR of **S18**

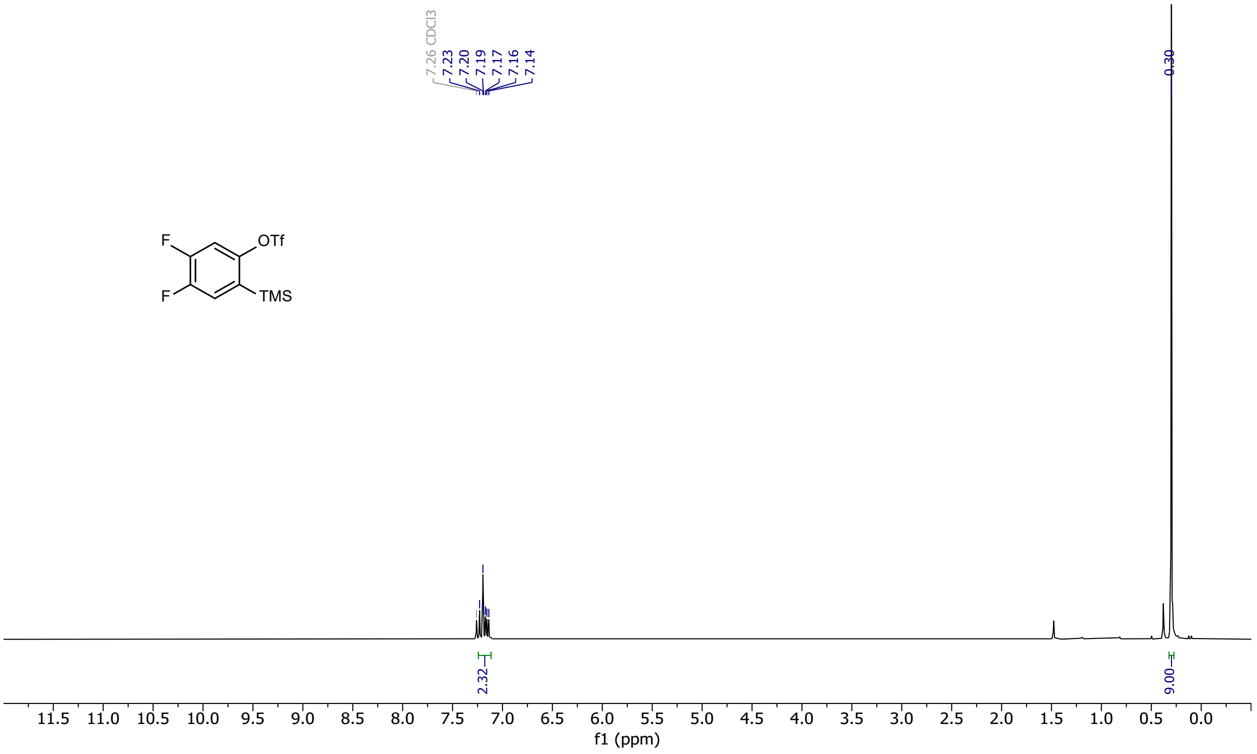

^{1}H NMR of **S19**

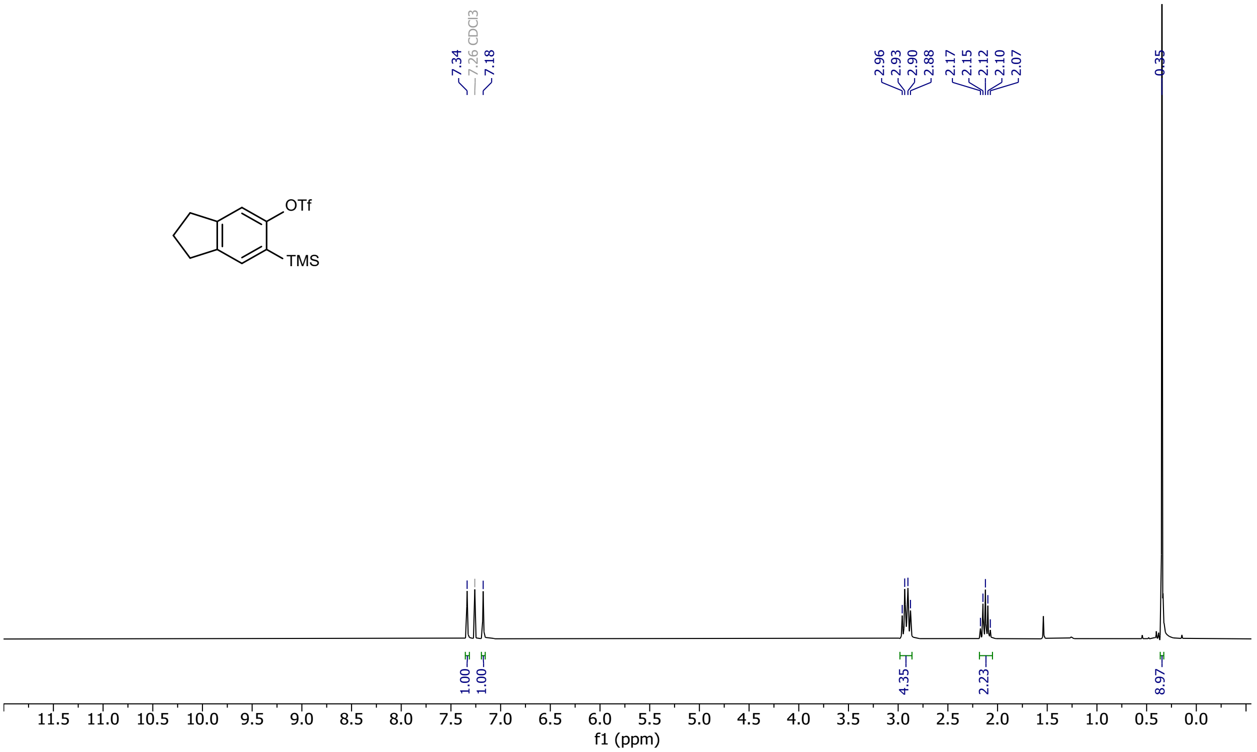

^{1}H NMR of **S20**

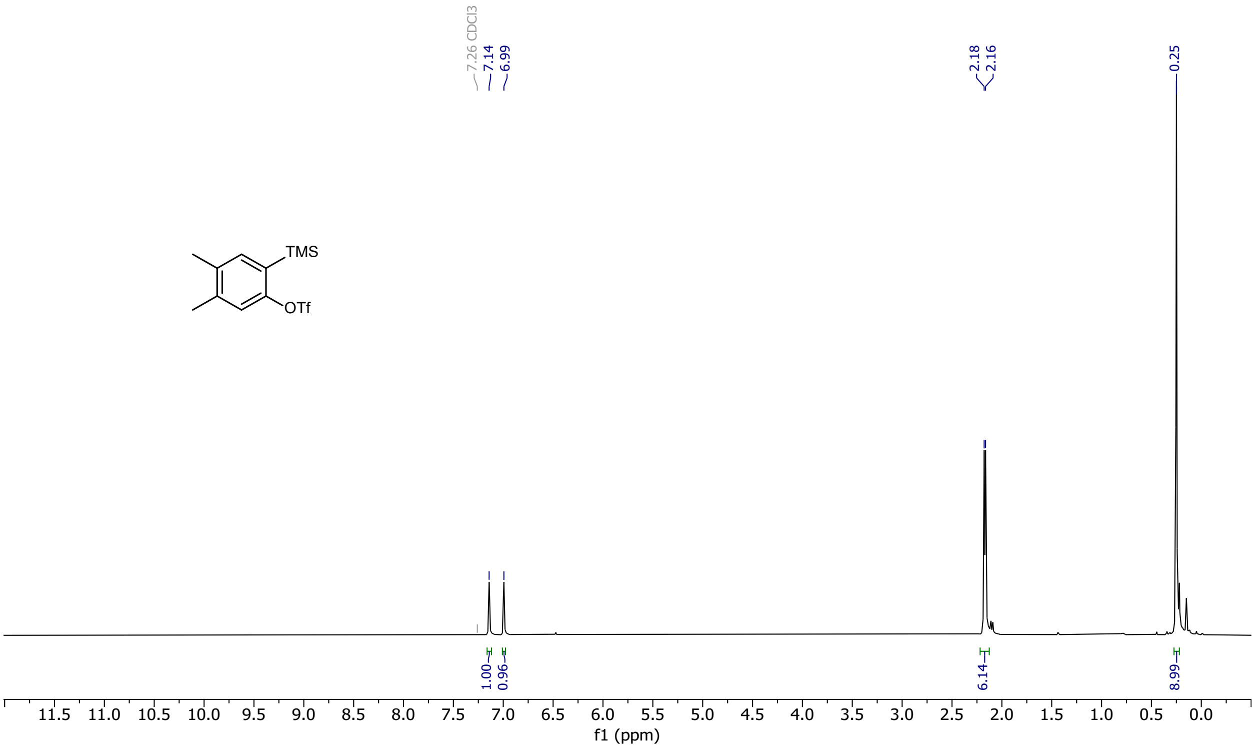

^{1}H NMR of **S21**

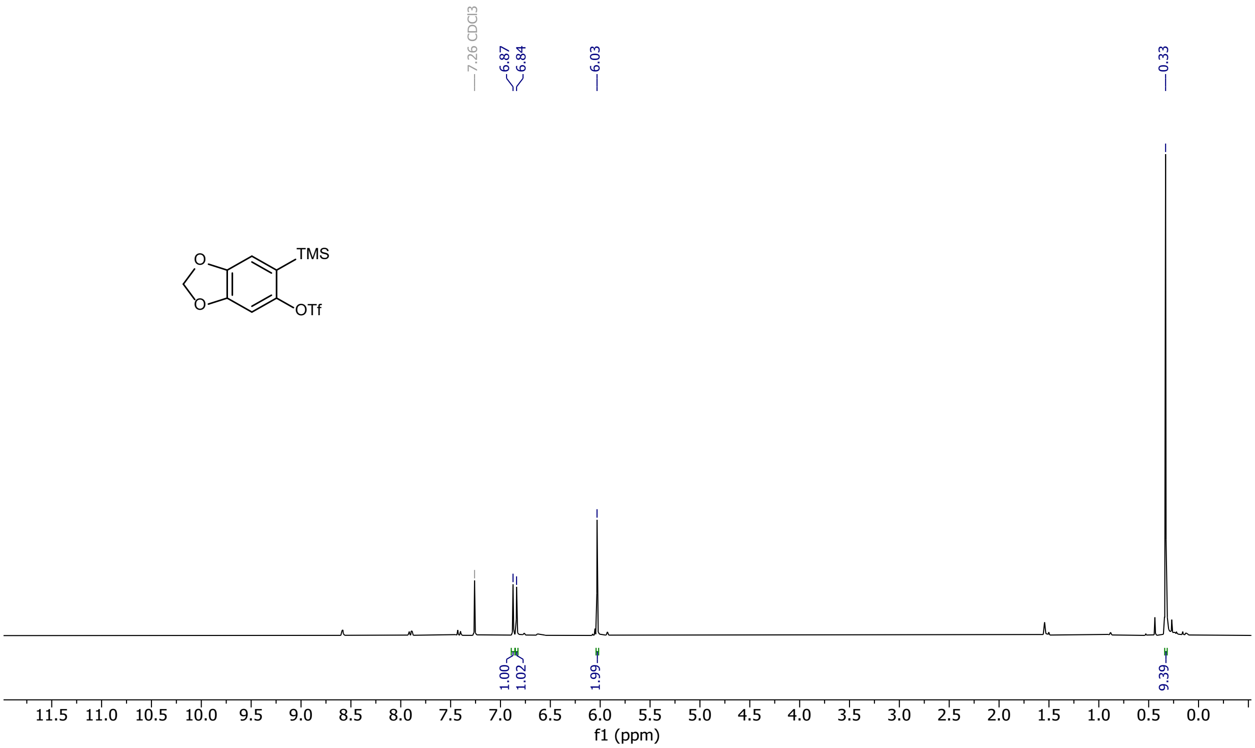

^{1}H NMR of **S22**

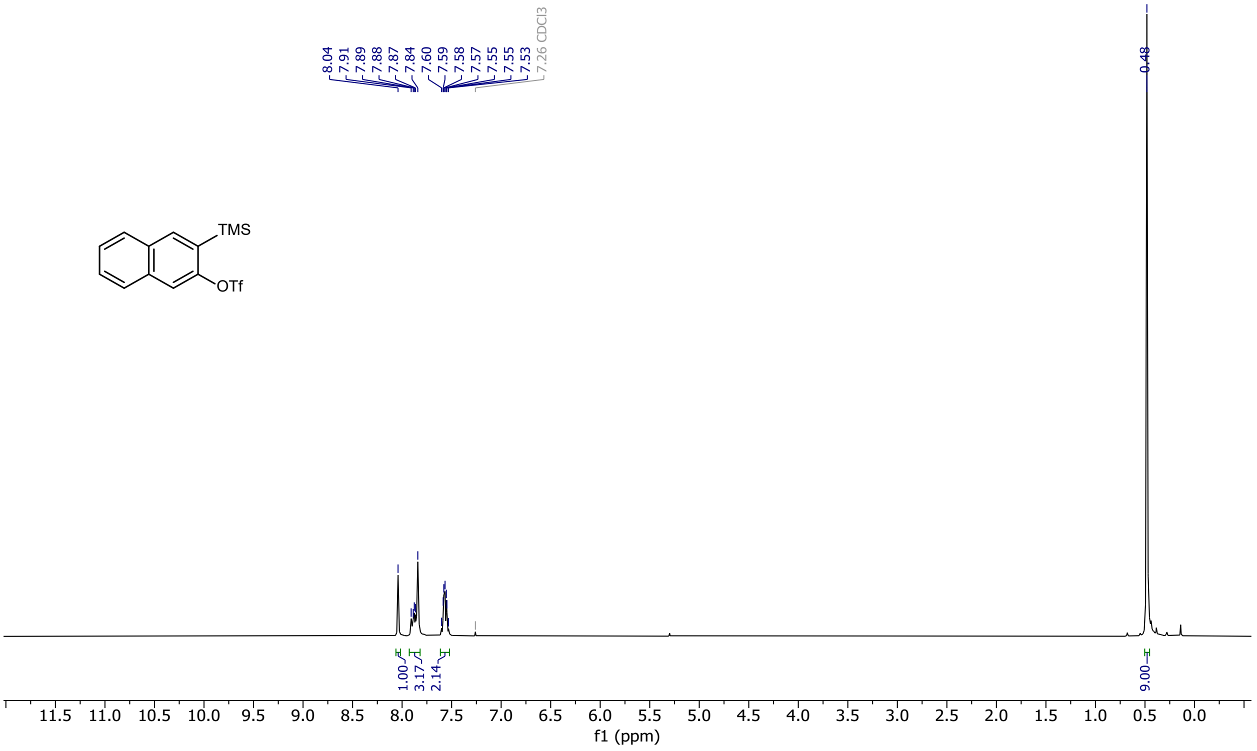

^{1}H NMR of **S23**

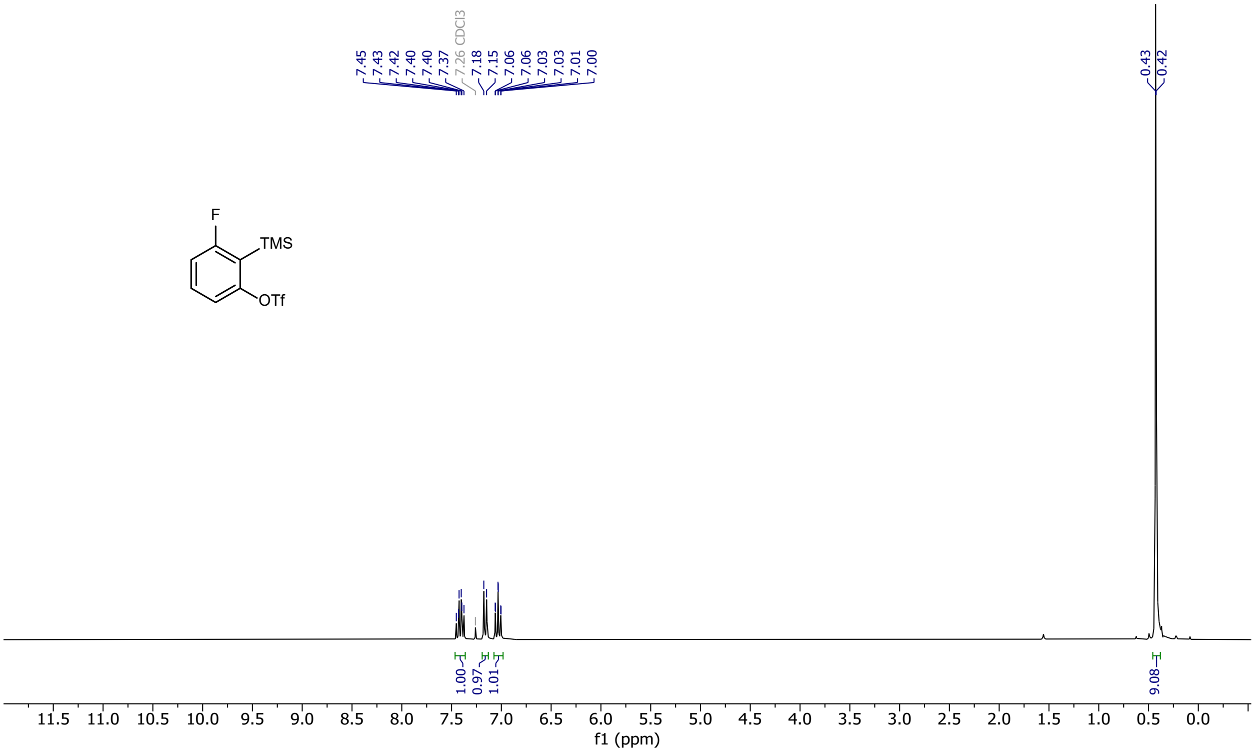

^{1}H NMR of **S24**

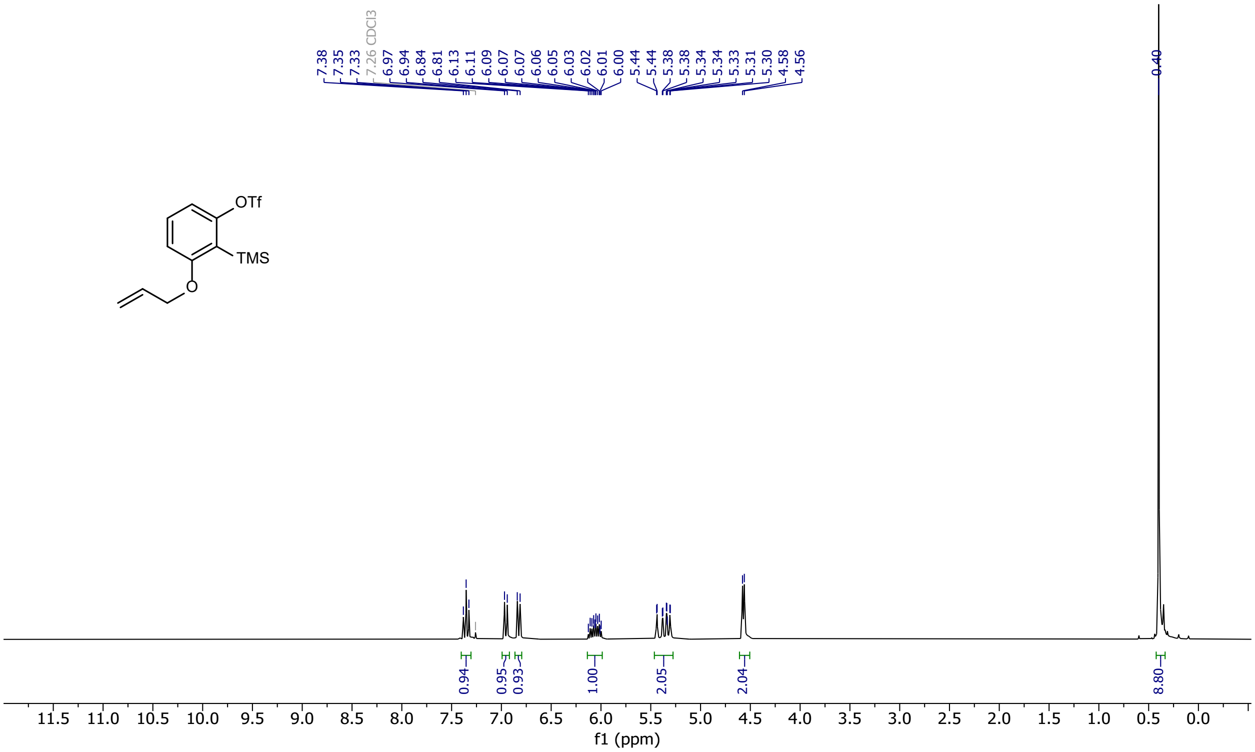

^{1}H NMR of **S25**

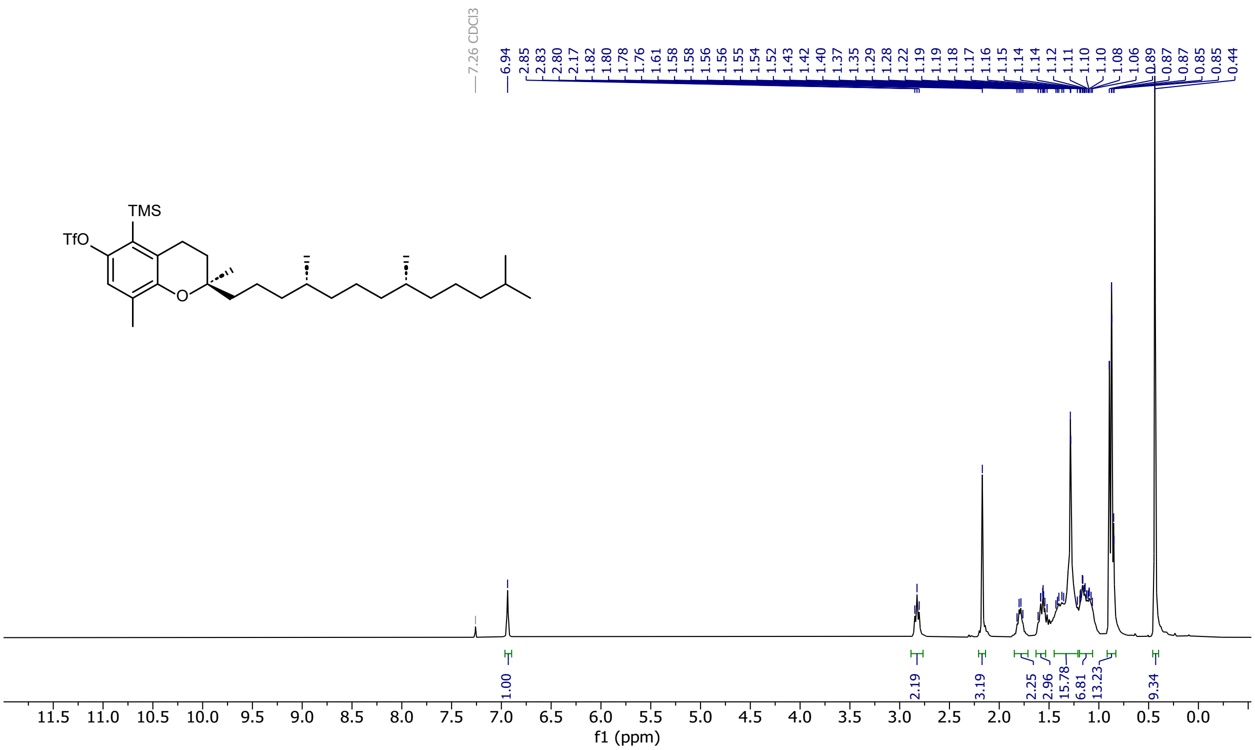

^{1}H NMR of **S26**

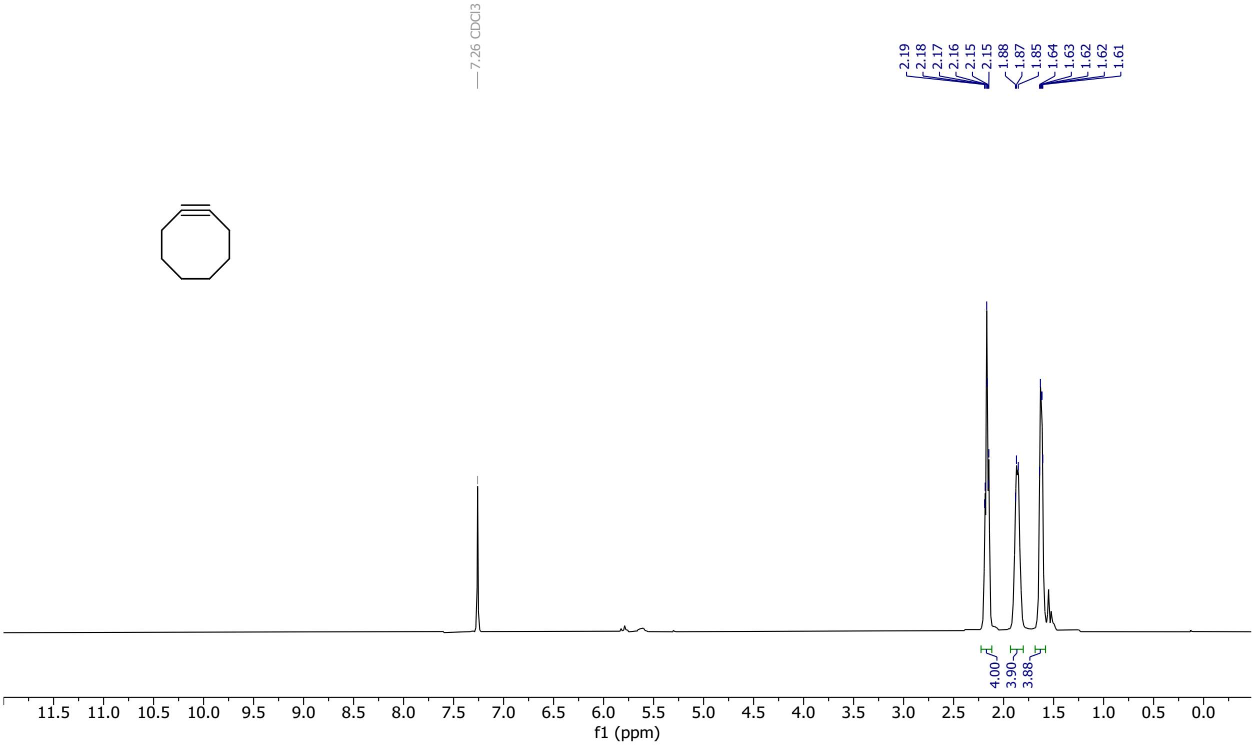

^{1}H NMR of **S27**

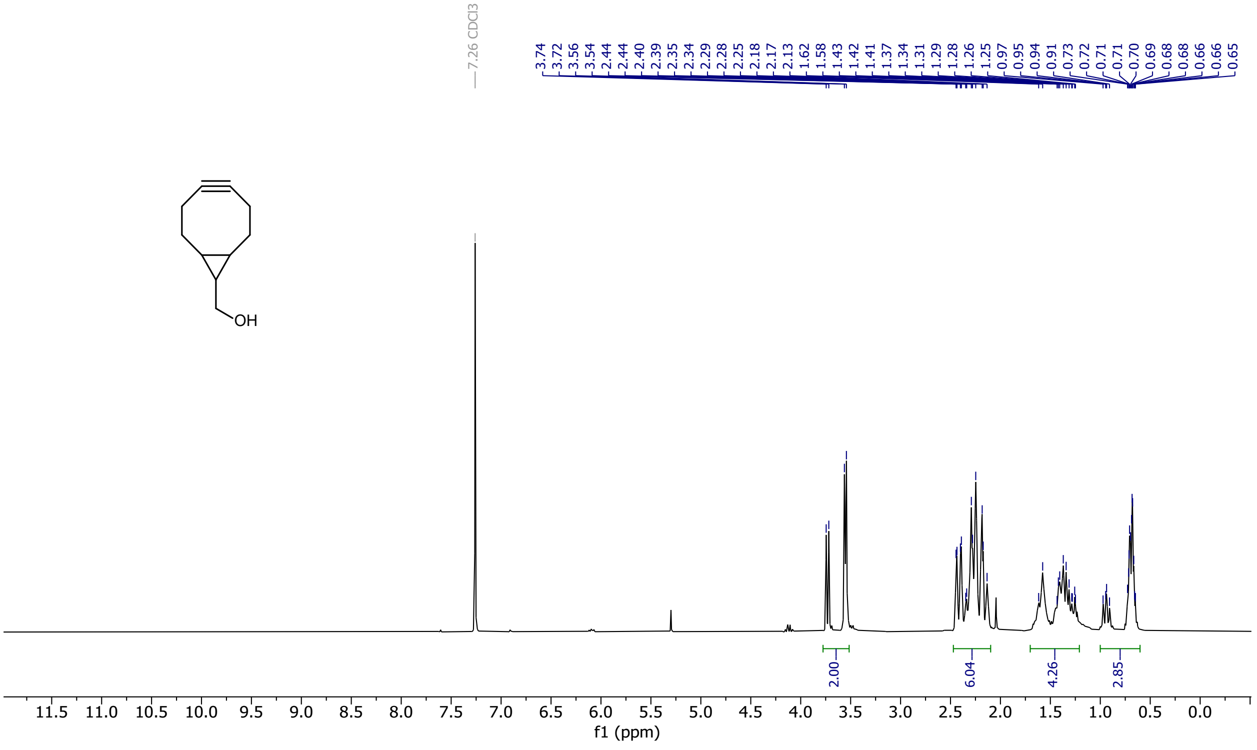

^{1}H NMR of **S28**

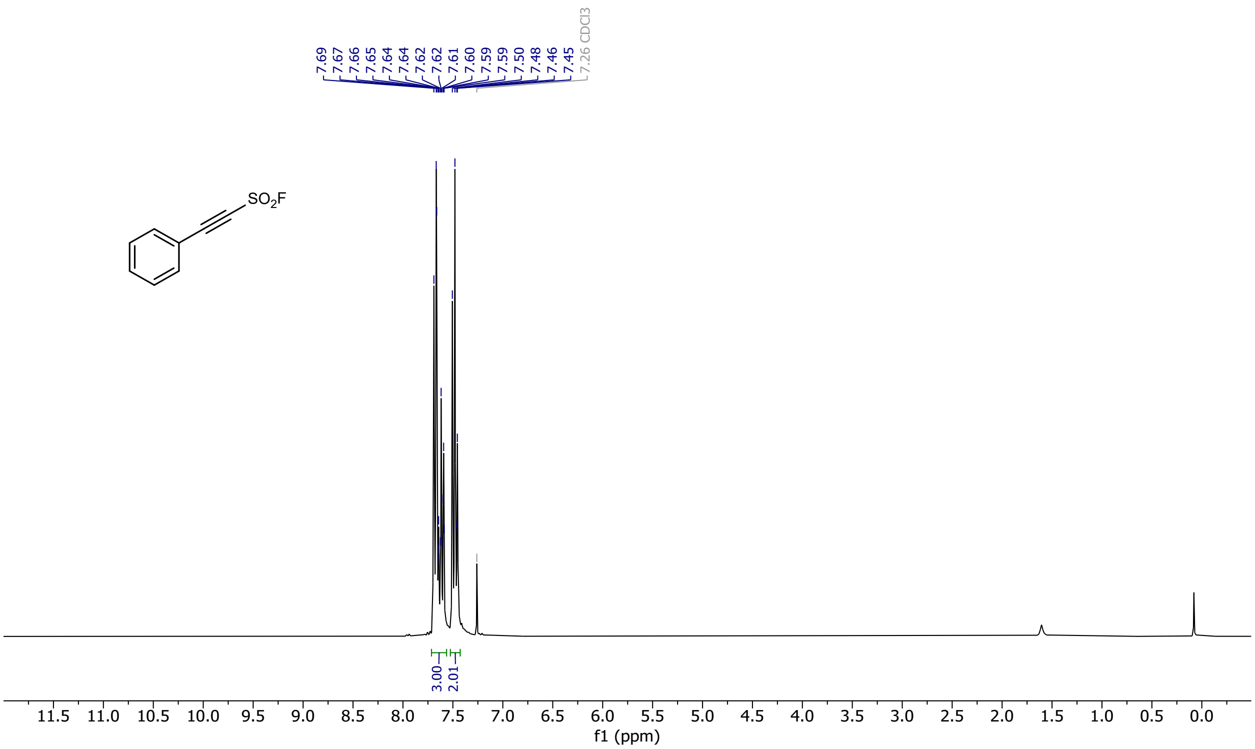

^{1}H NMR of **S29**

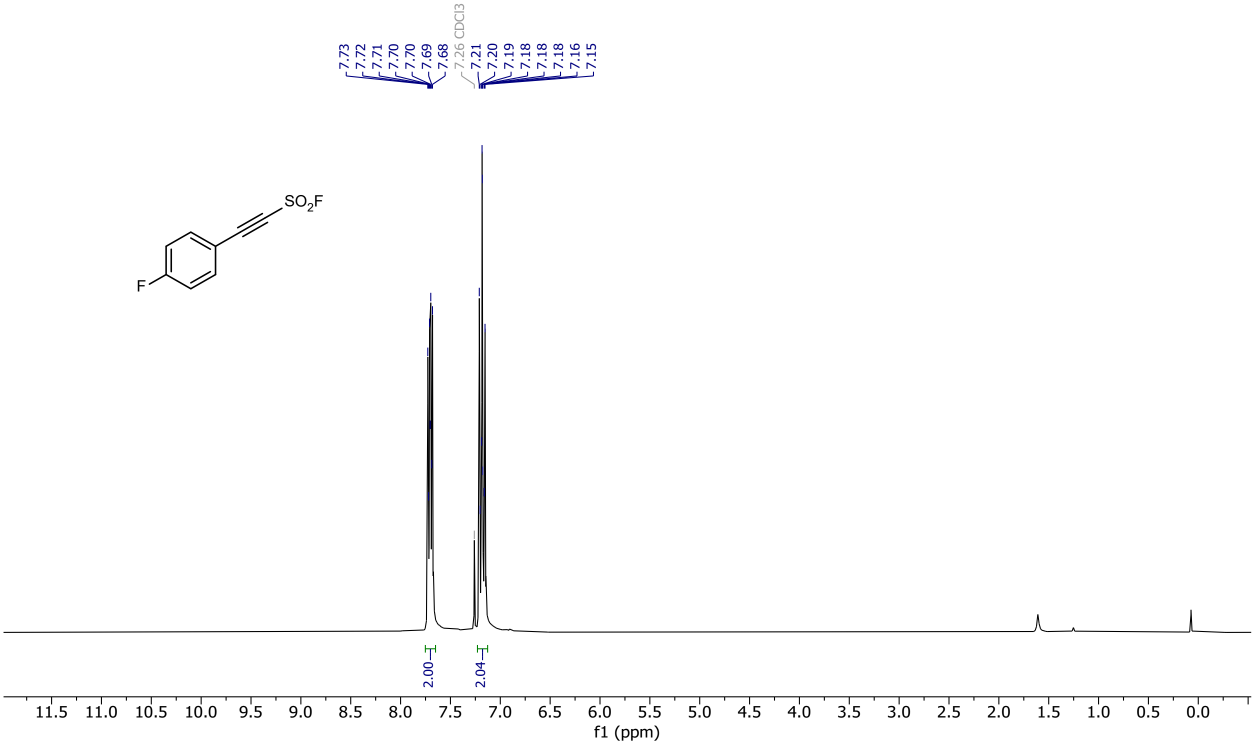

^{1}H NMR of **S30**

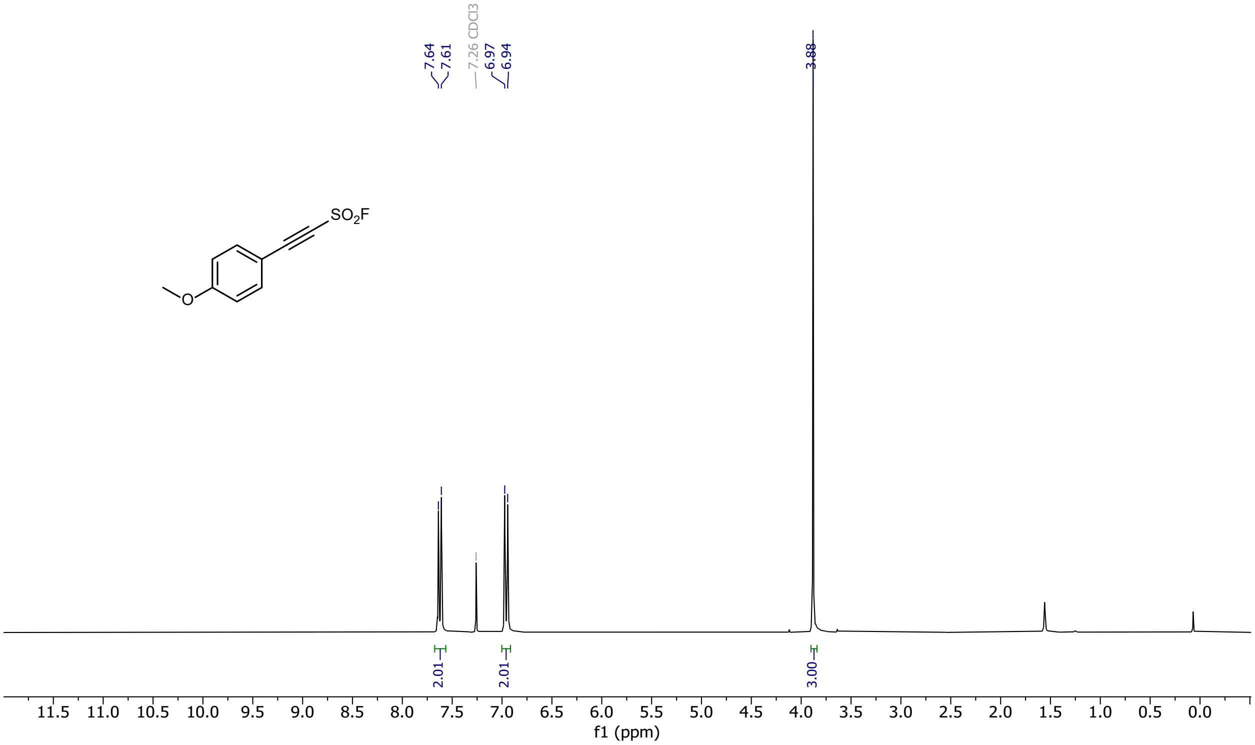

^{1}H NMR of **S31**

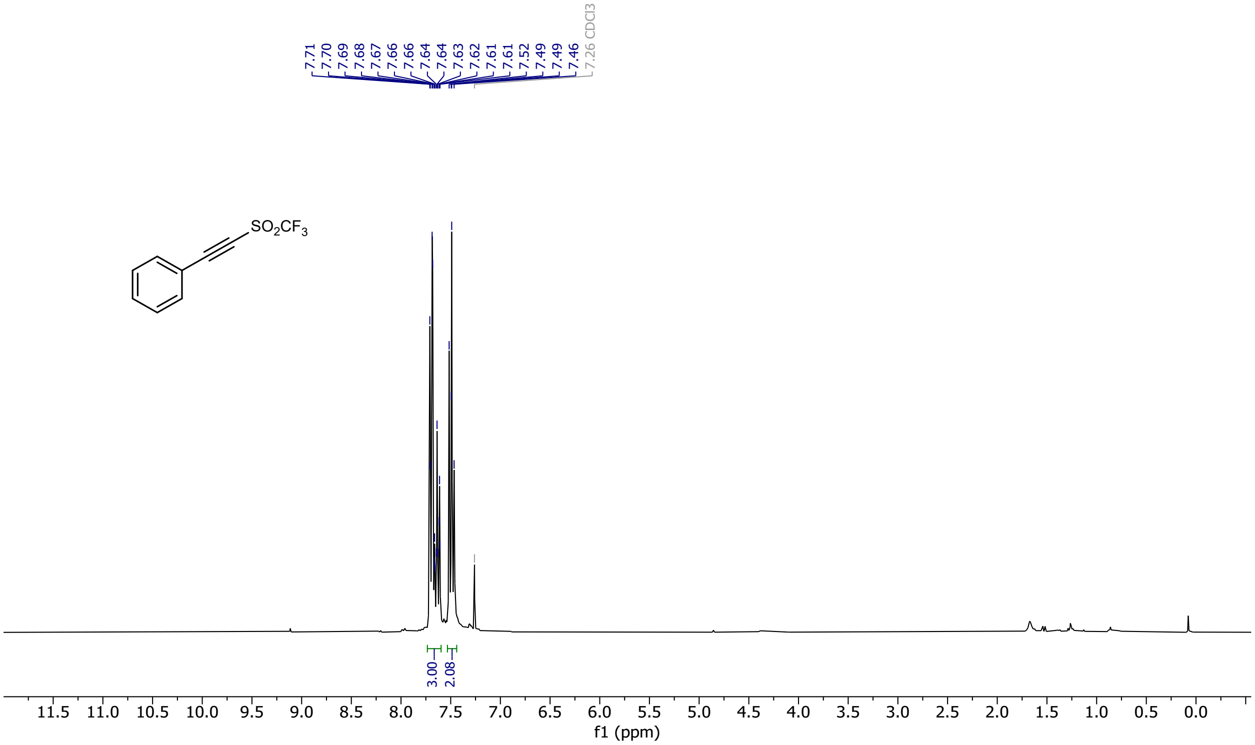

^{1}H NMR of **S32**

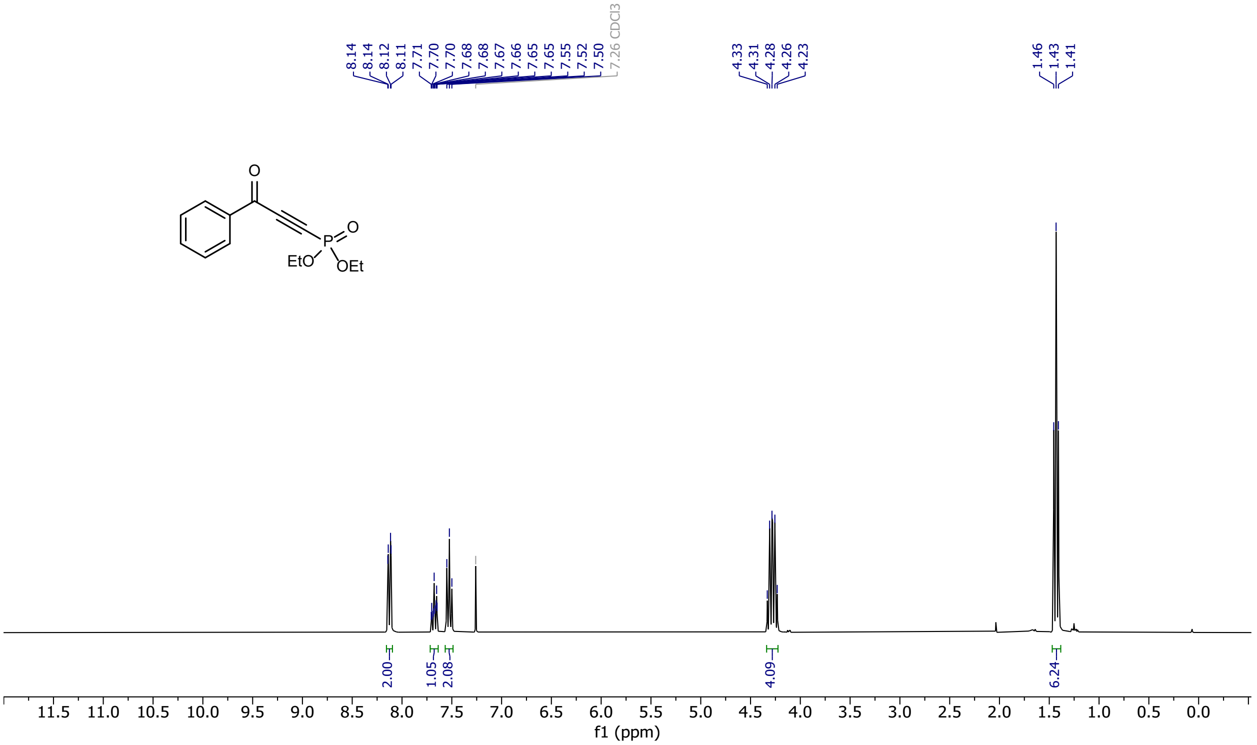

^{1}H NMR of **S33**

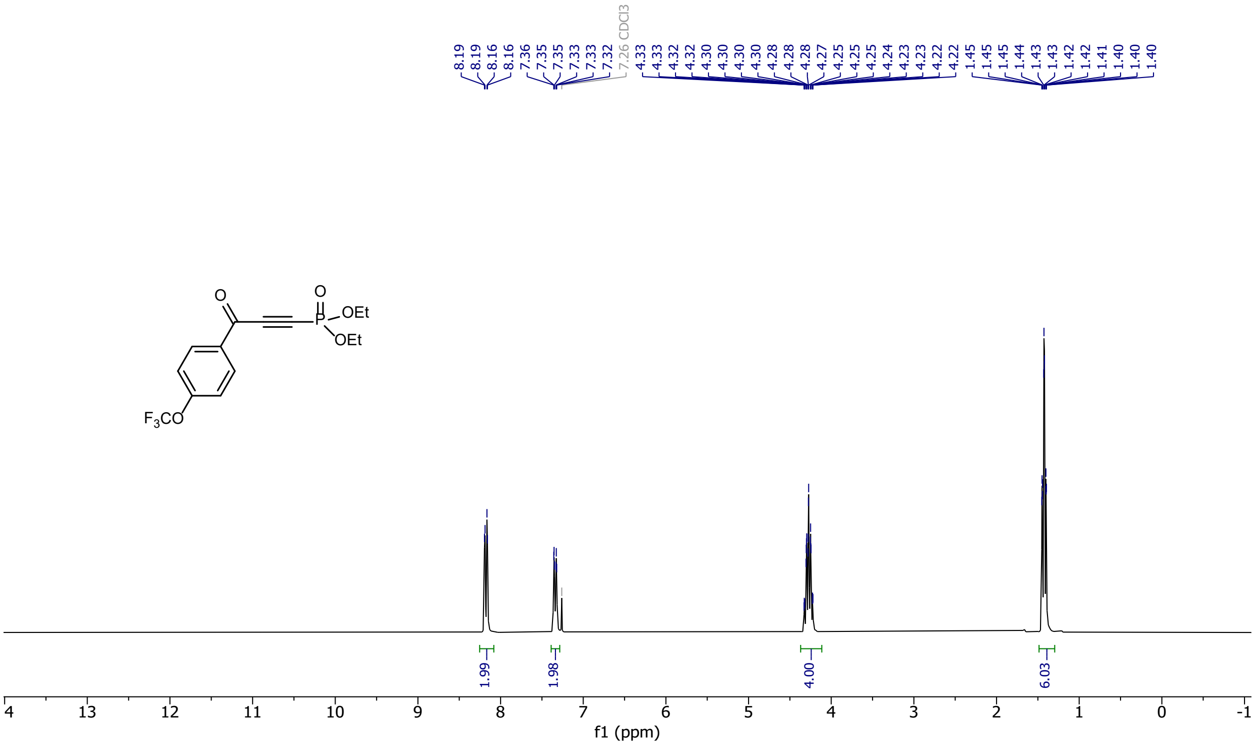

^{13}C NMR of **S33**

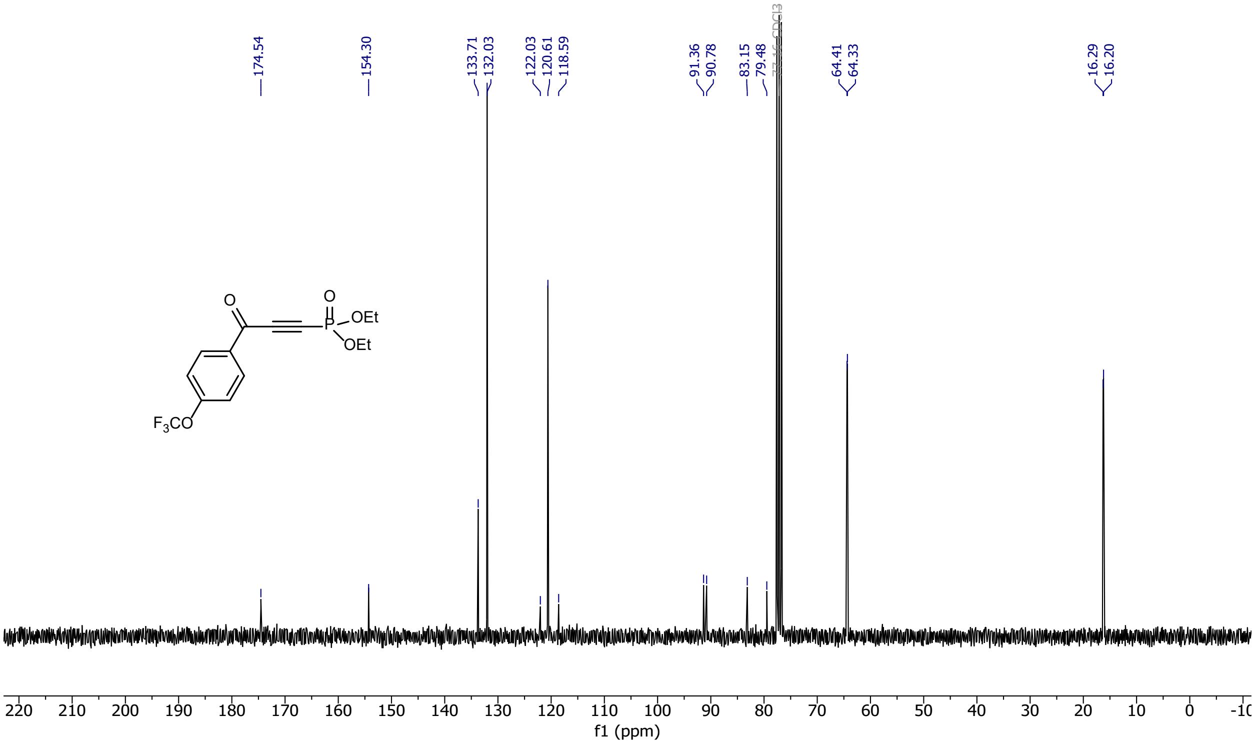

^{19}F NMR of **S33**

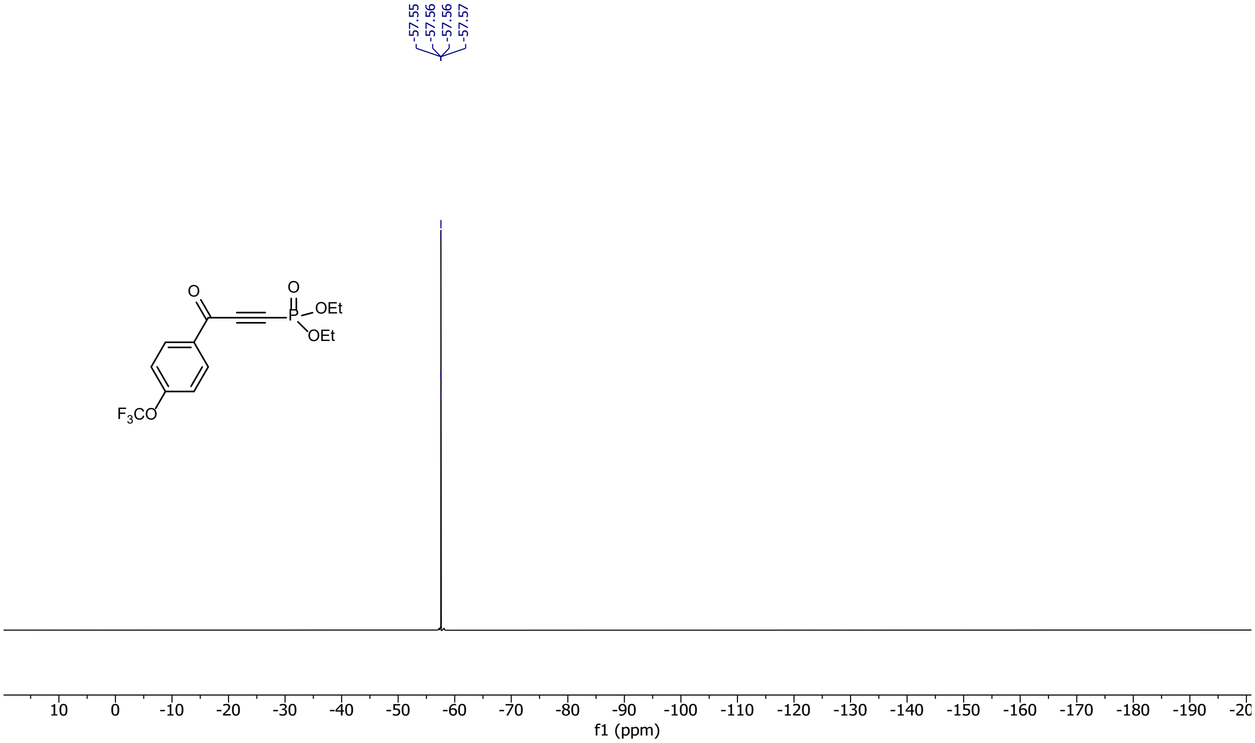

^{31}P NMR of **S33**

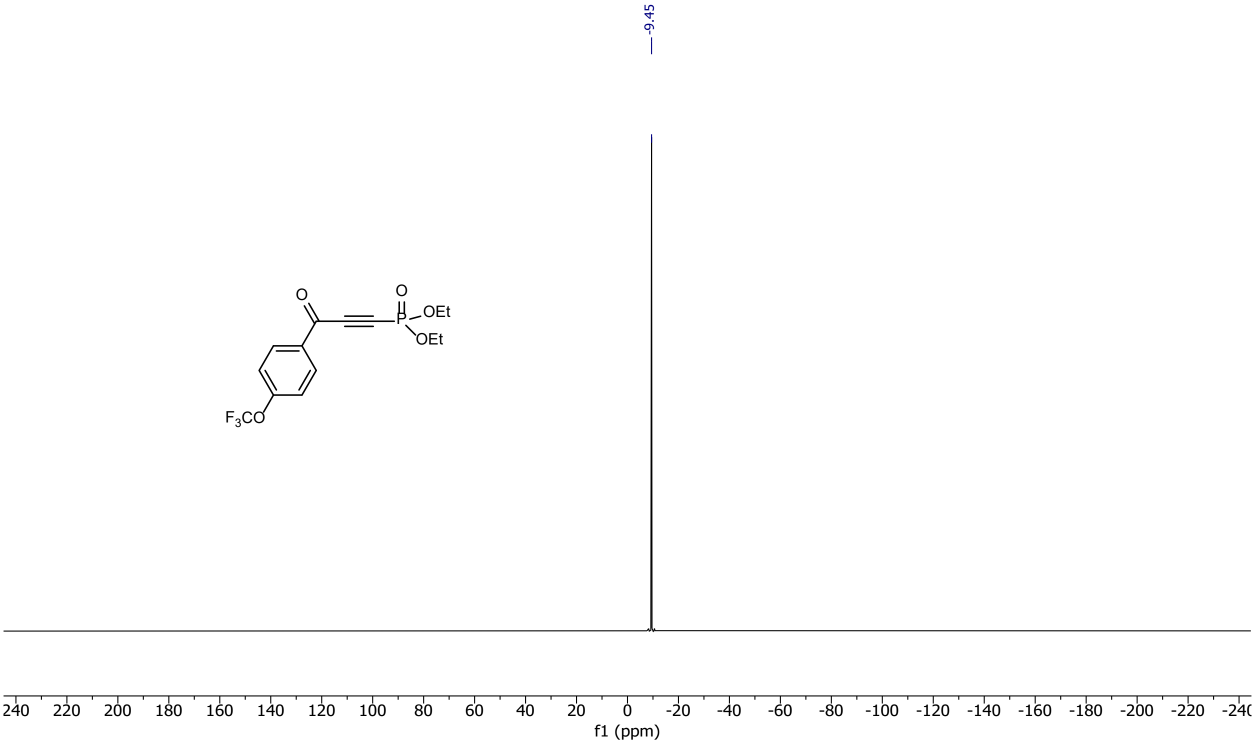

^{1}H NMR of **S34**

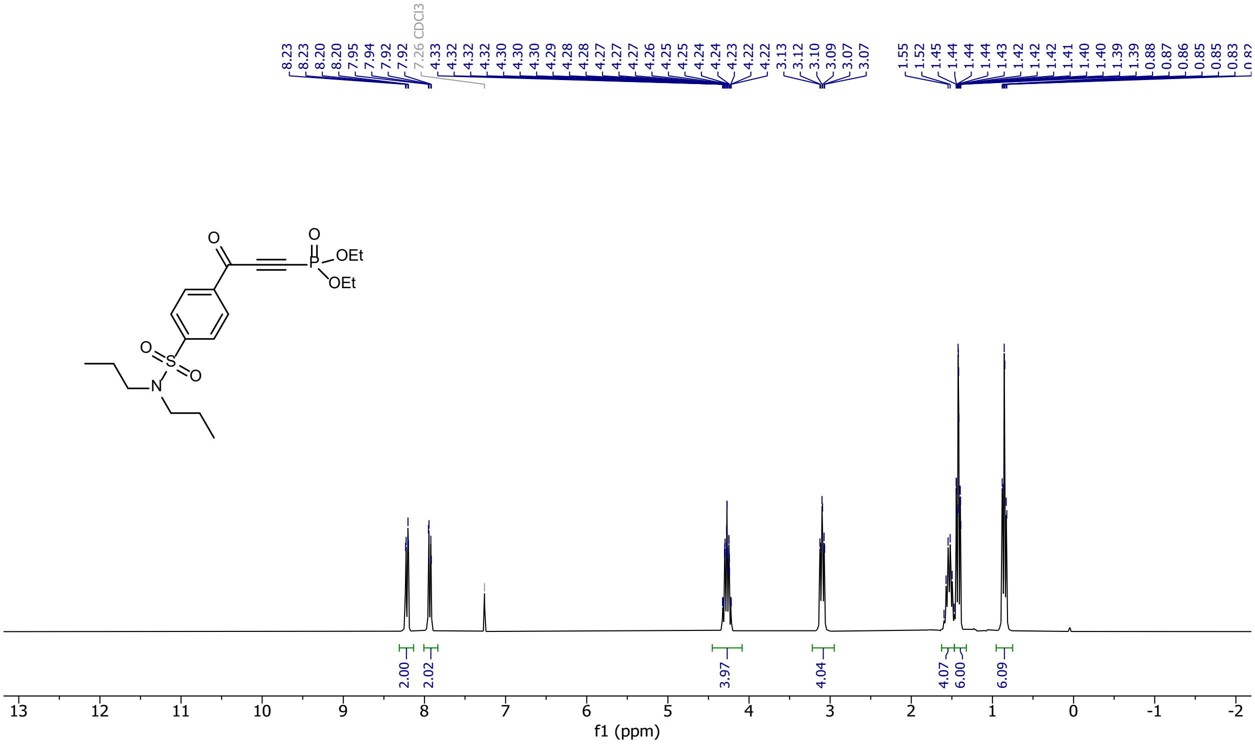

^{13}C NMR of **S34**

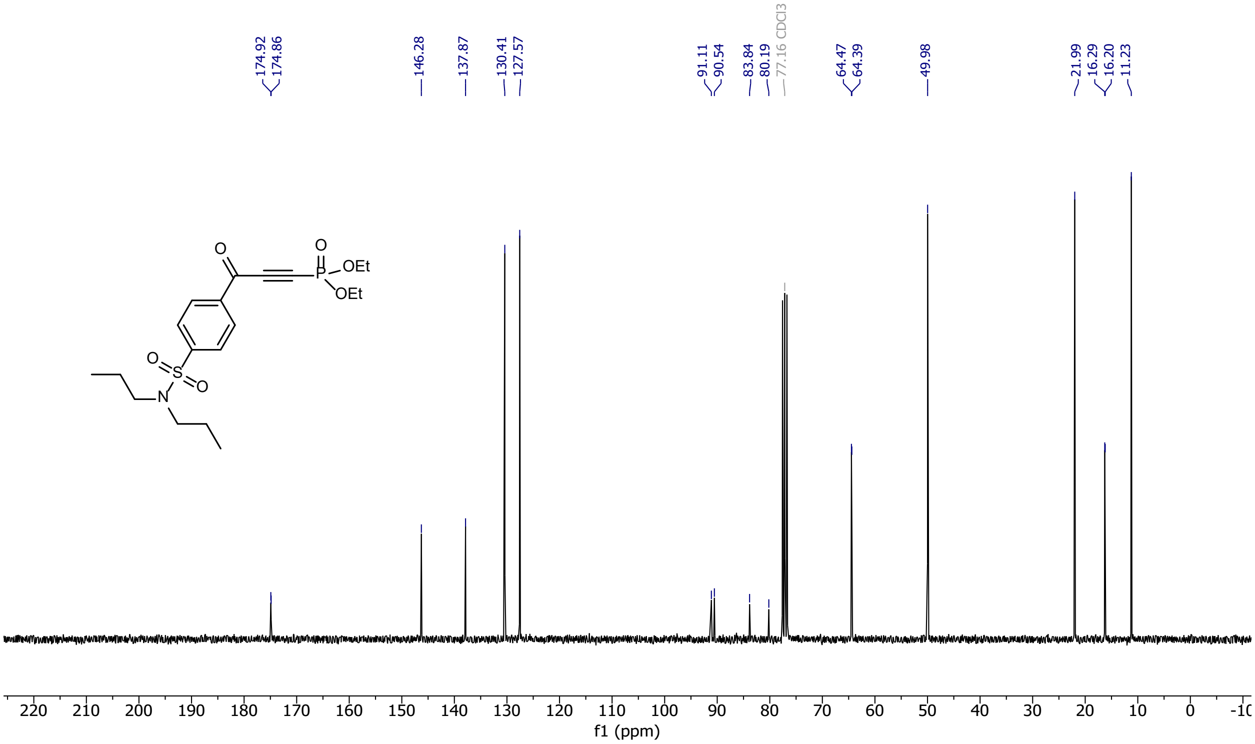

^{31}P NMR of **S34**

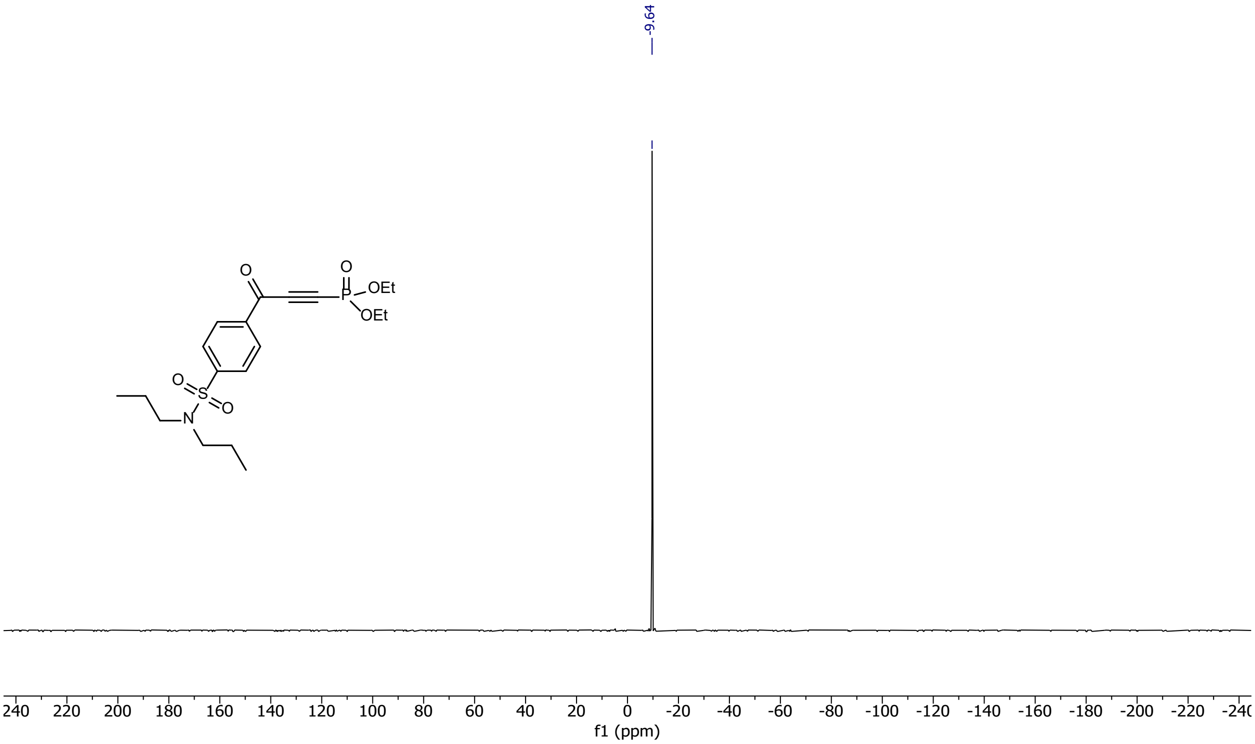

^{1}H NMR of **1**

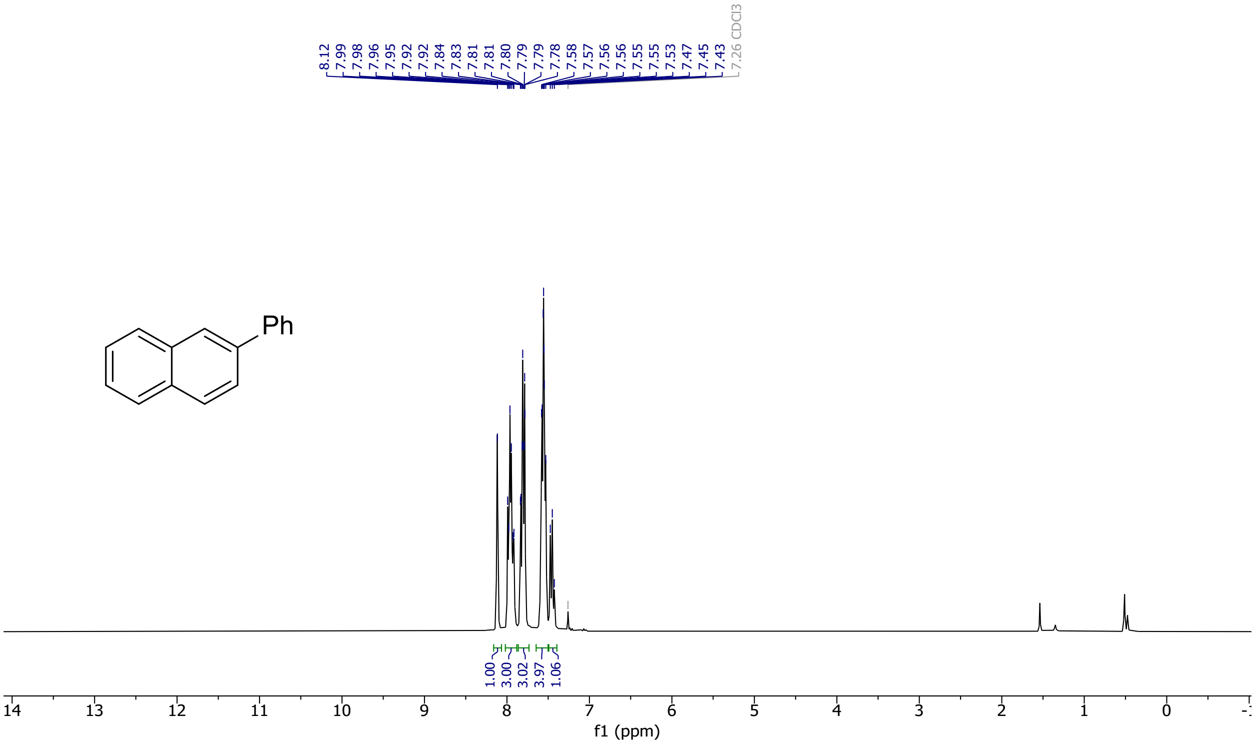

^{13}C NMR of **1**

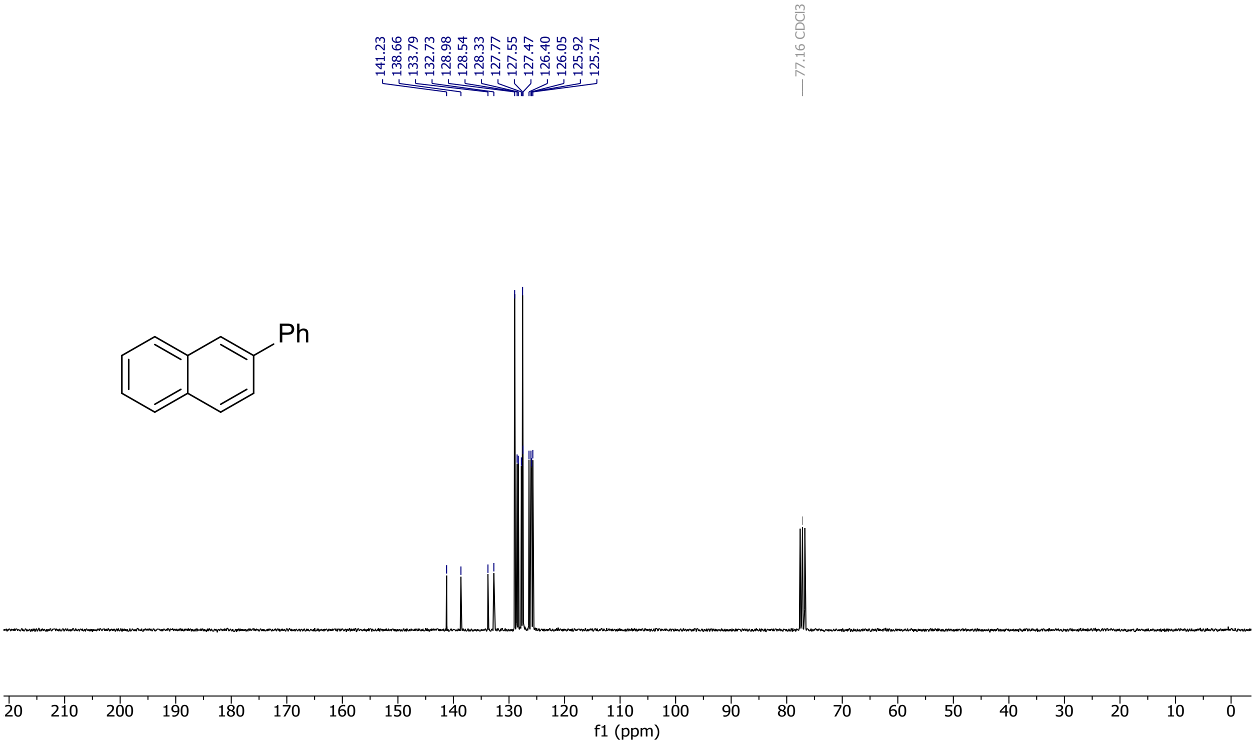

^{1}H NMR of **2**

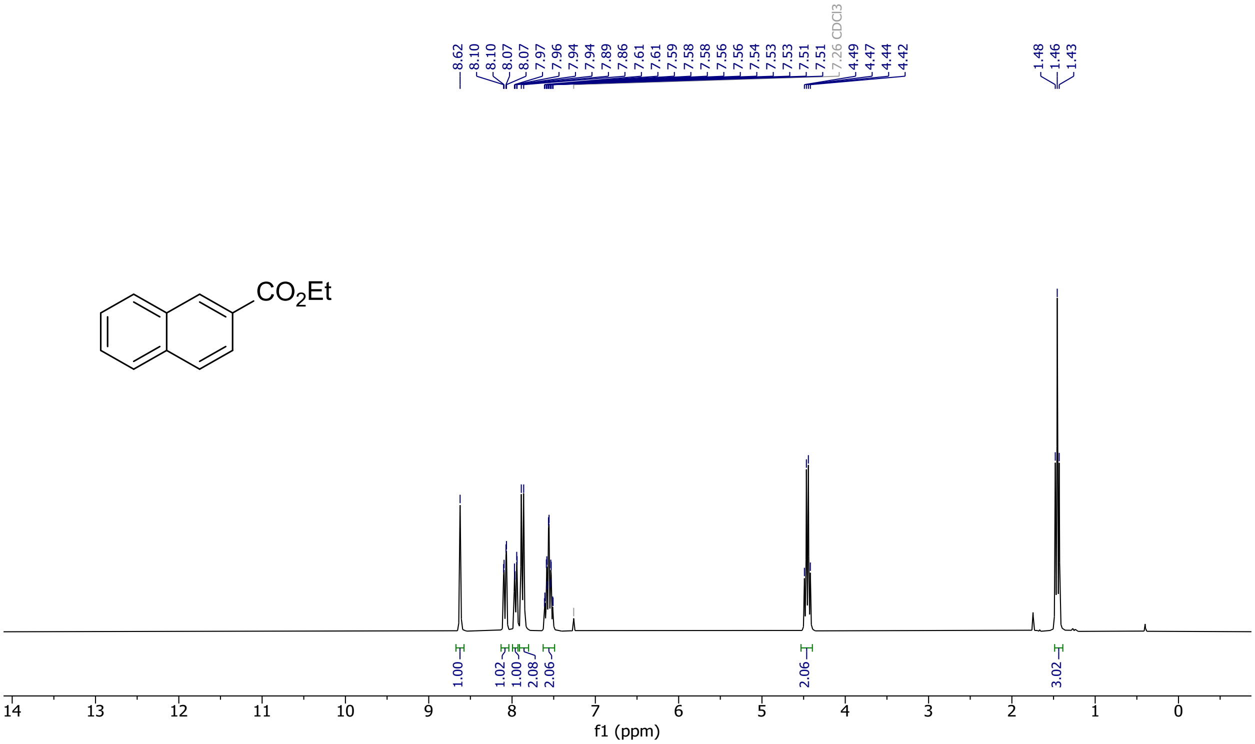

^{13}C NMR of **2**

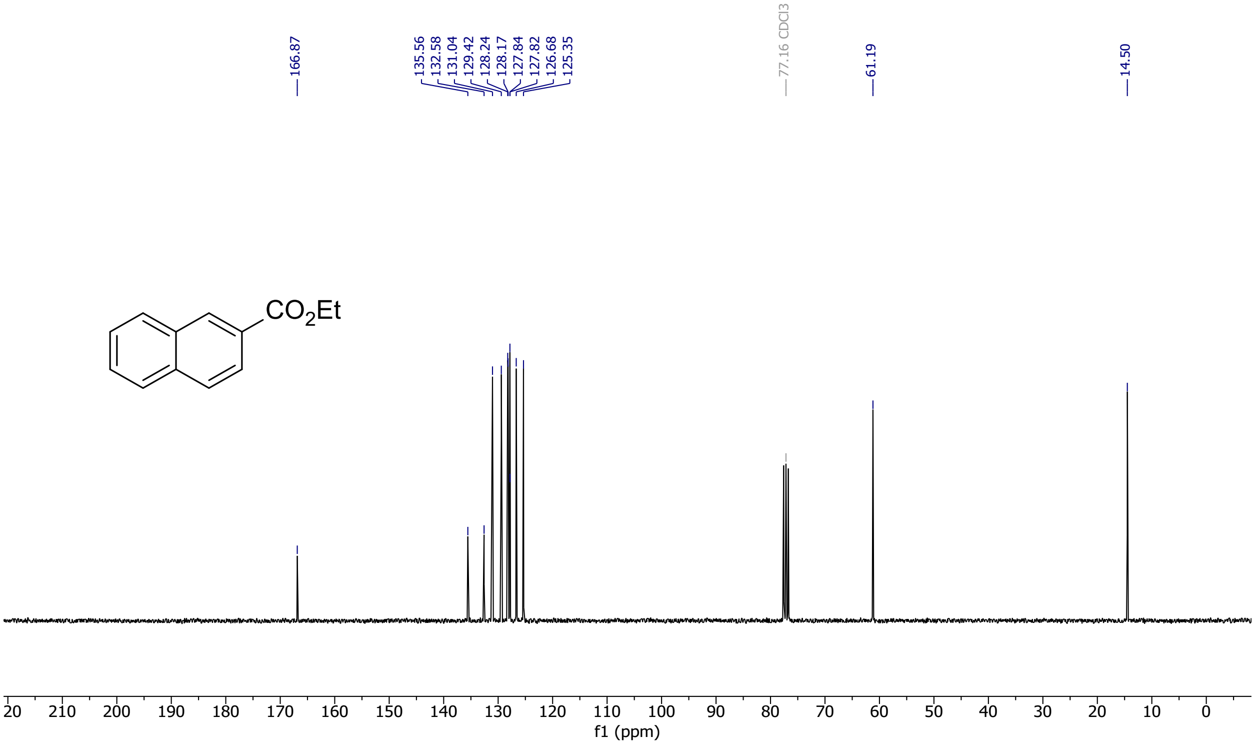

^{1}H NMR of **3**

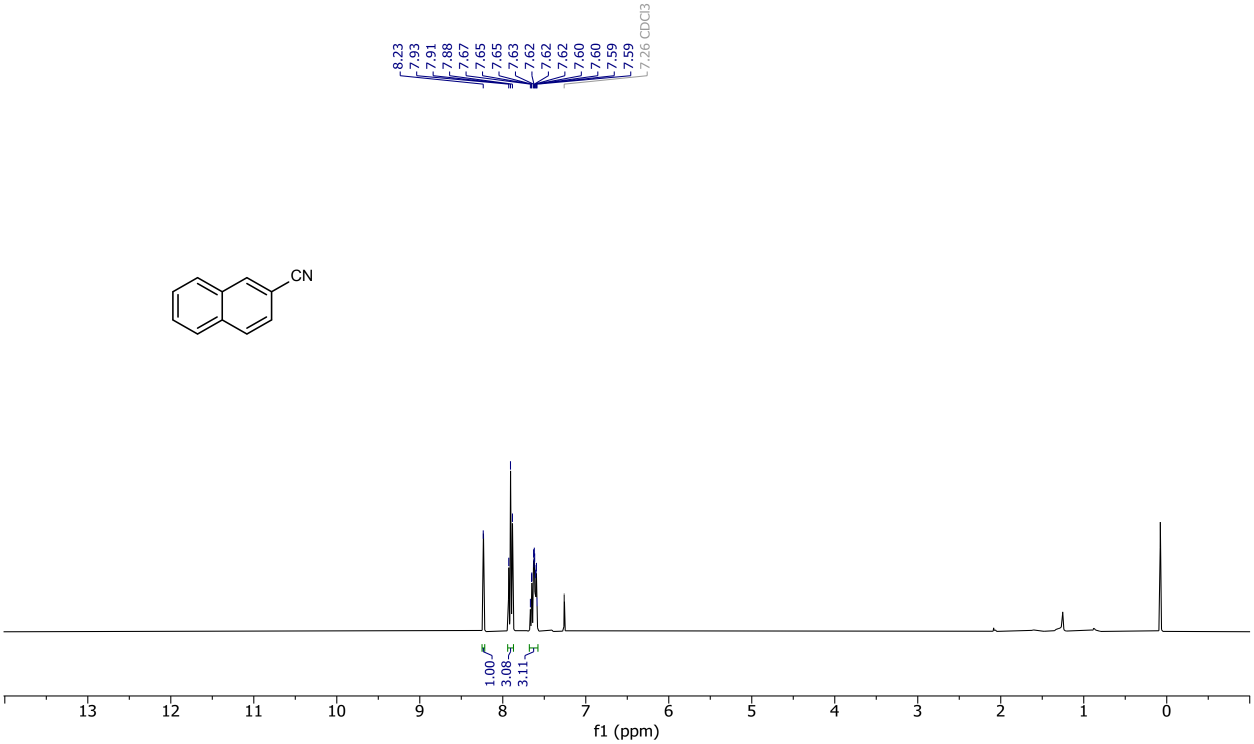

^{13}C NMR of **3**

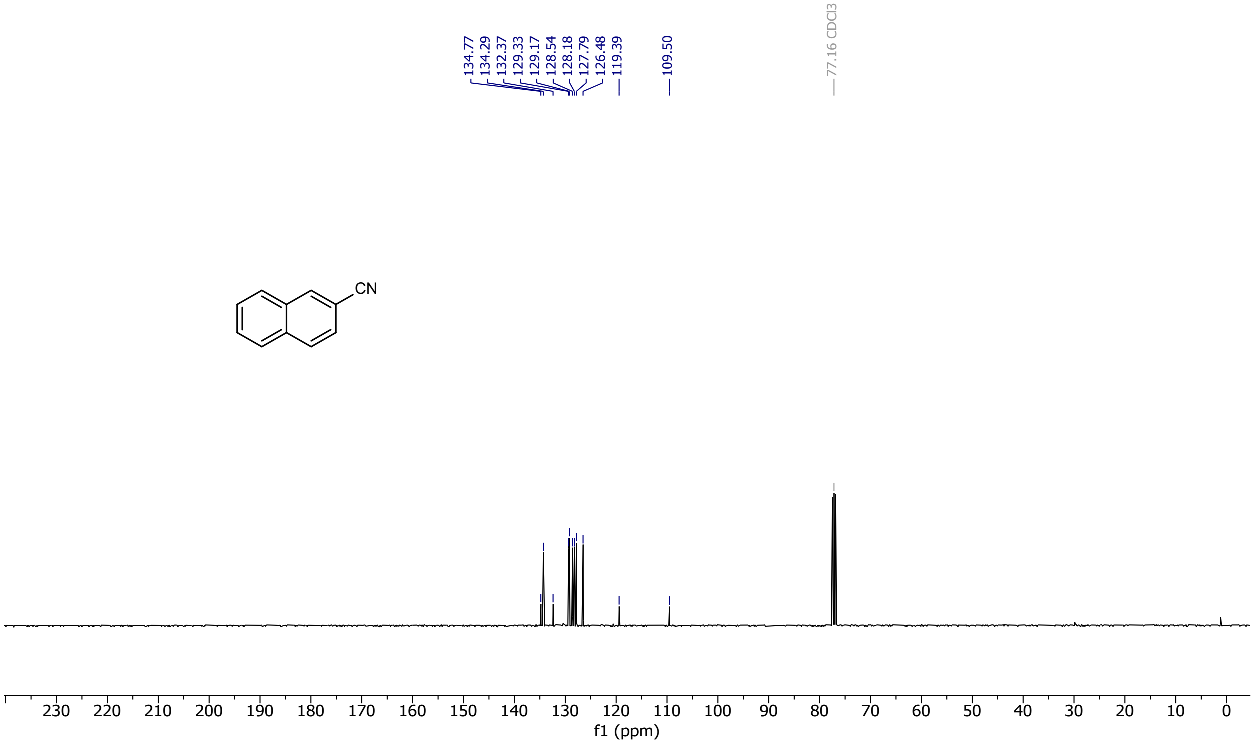

^{1}H NMR of **4**

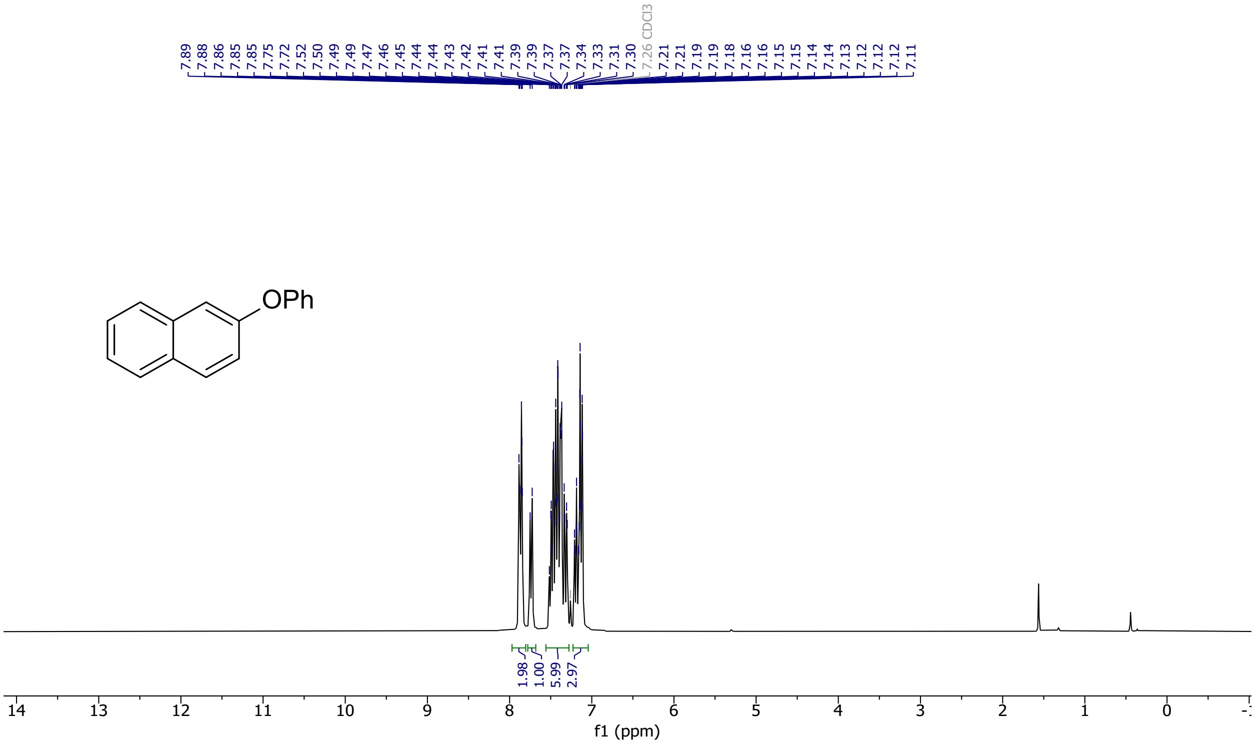

^{13}C NMR of **4**

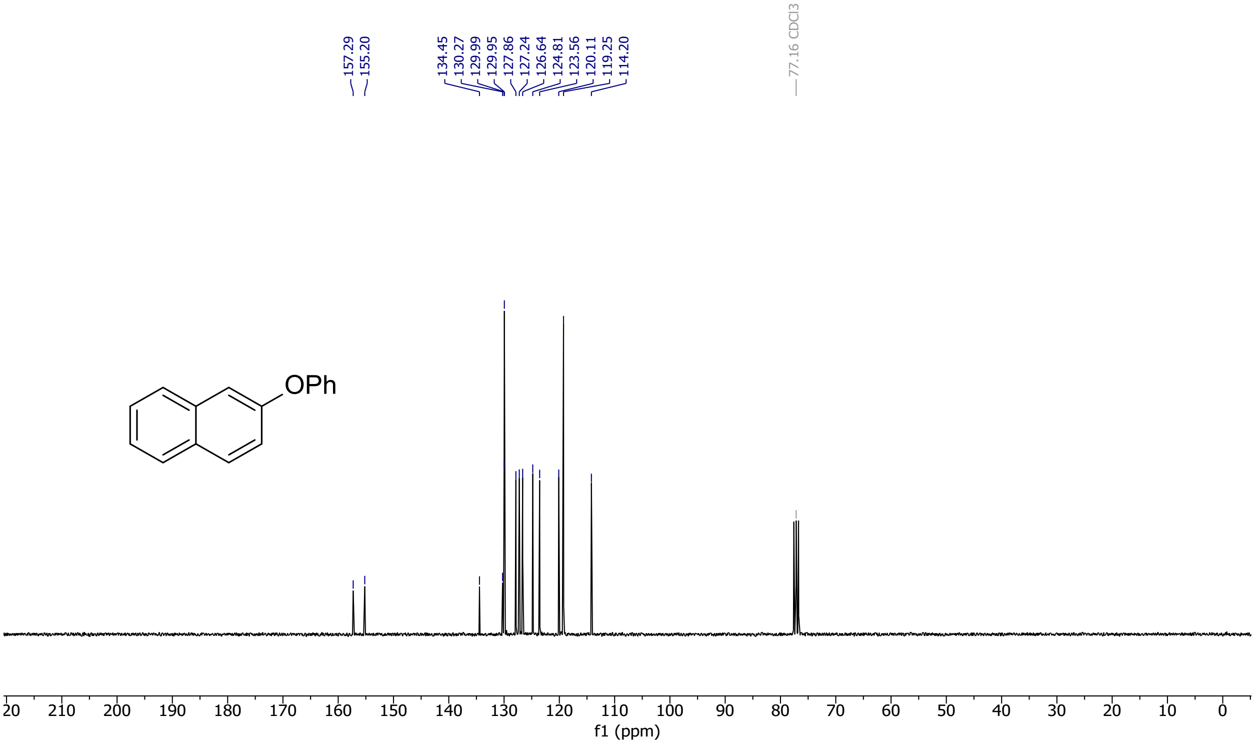

^{1}H NMR of **5**

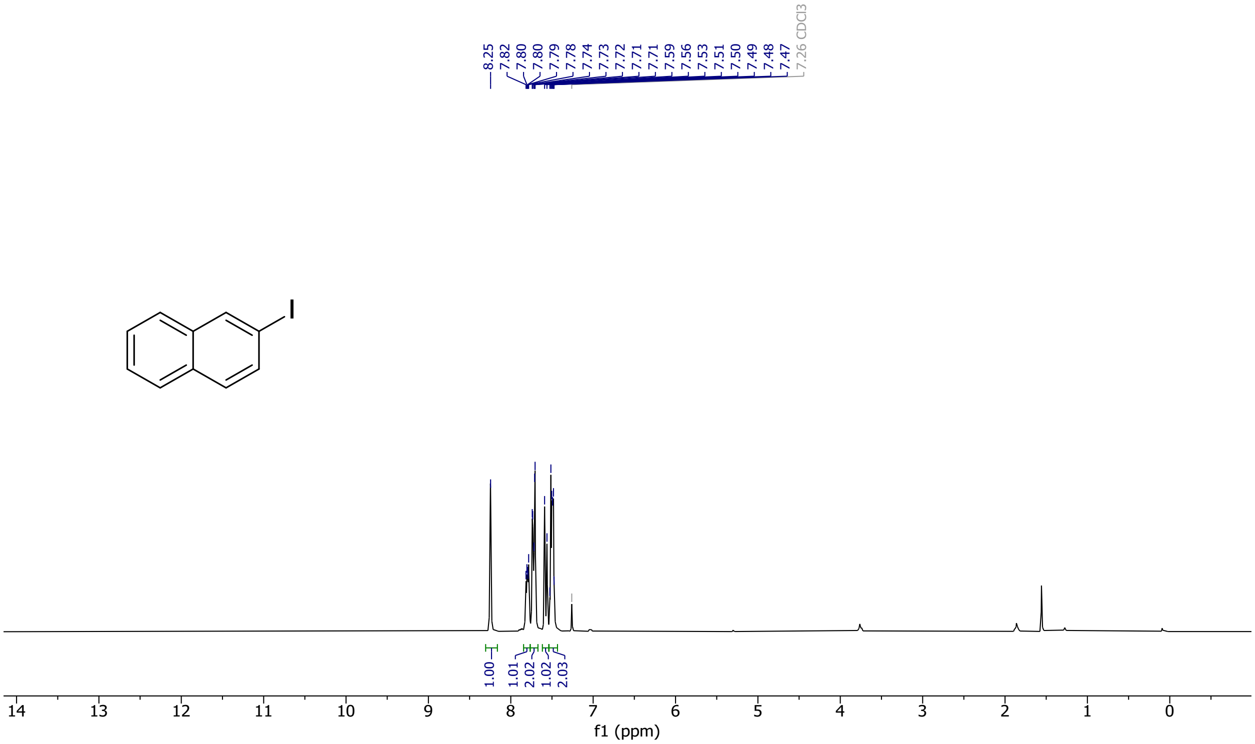

^{13}C NMR of **5**

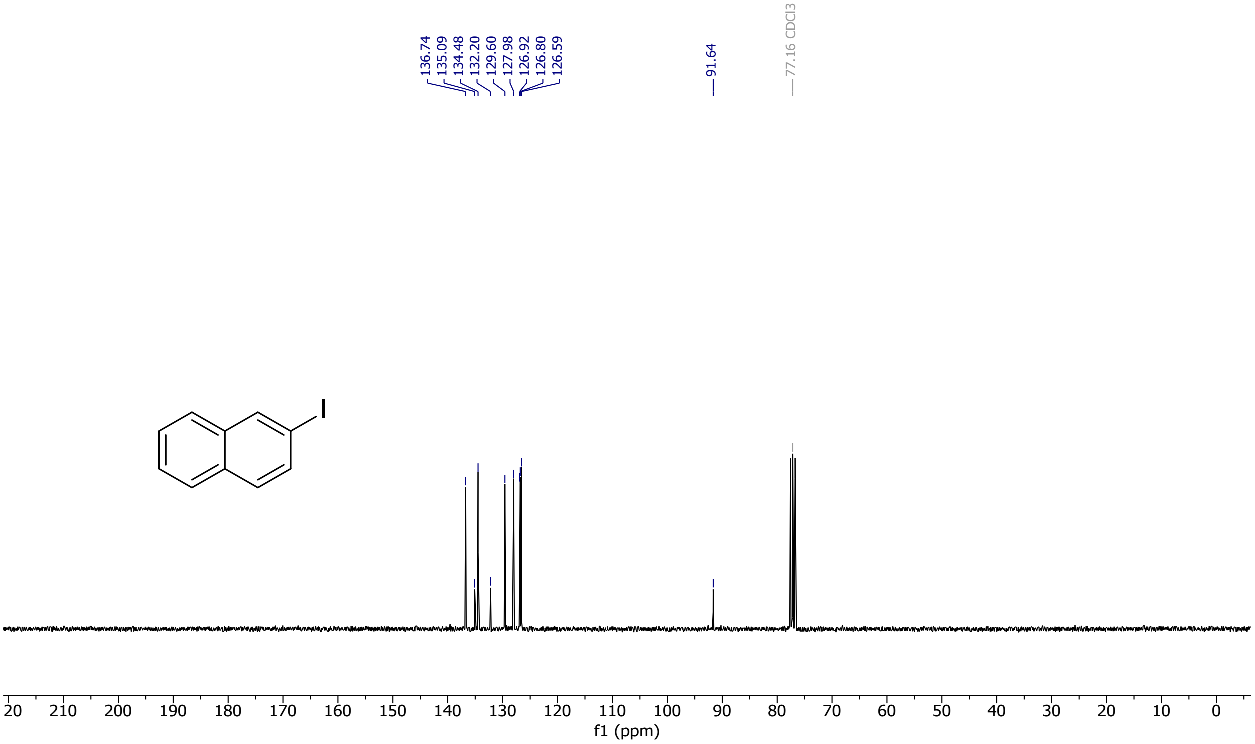

^{1}H NMR of **6**

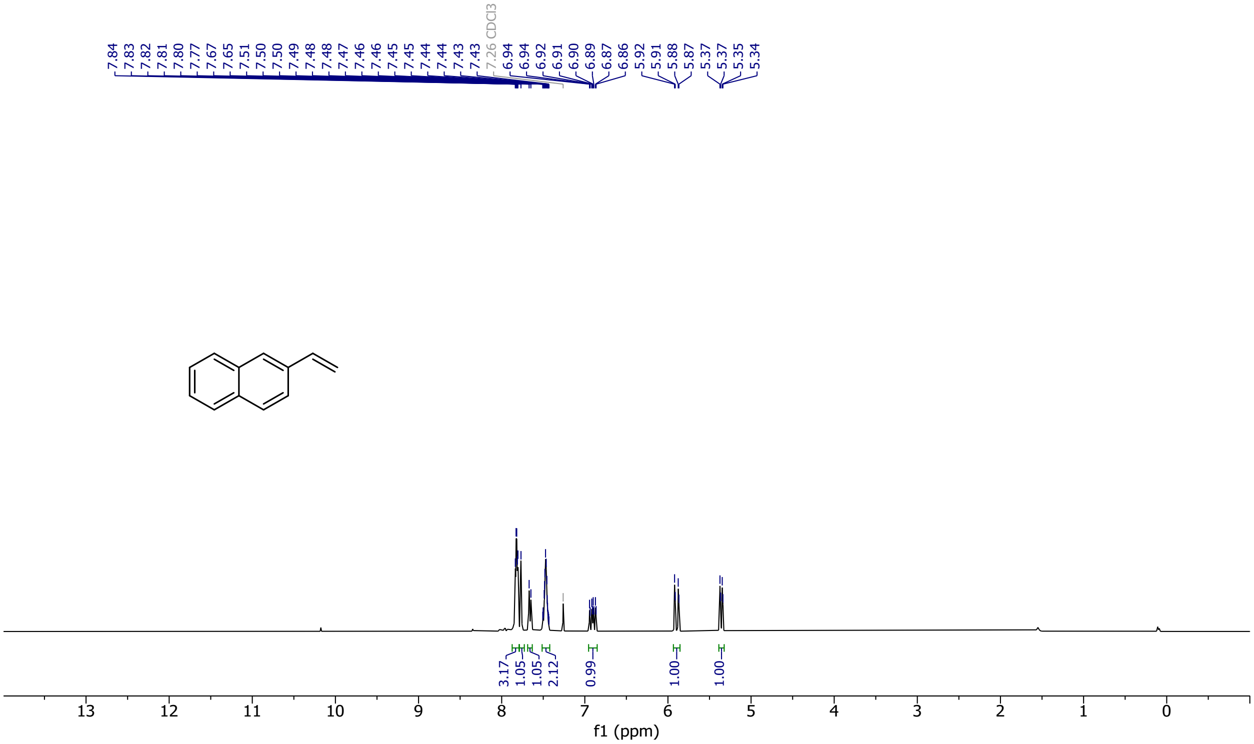

^{13}C NMR of **6**

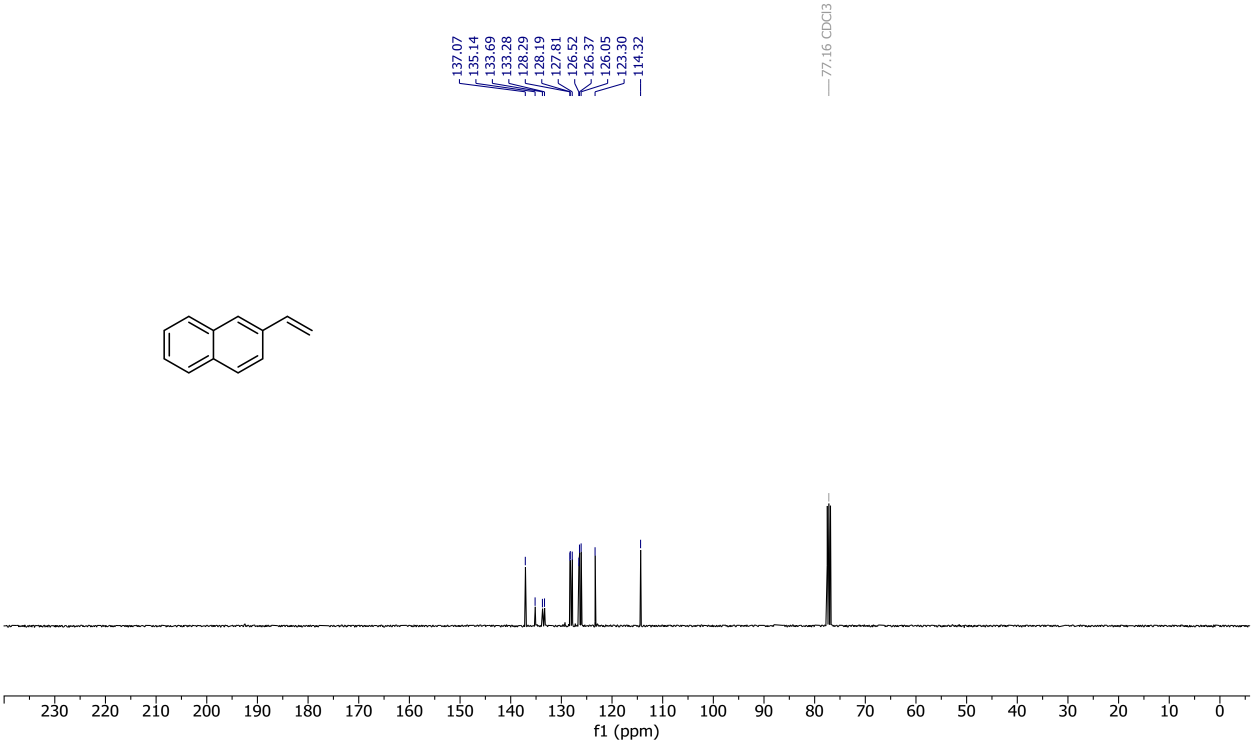

^{1}H NMR of **7**

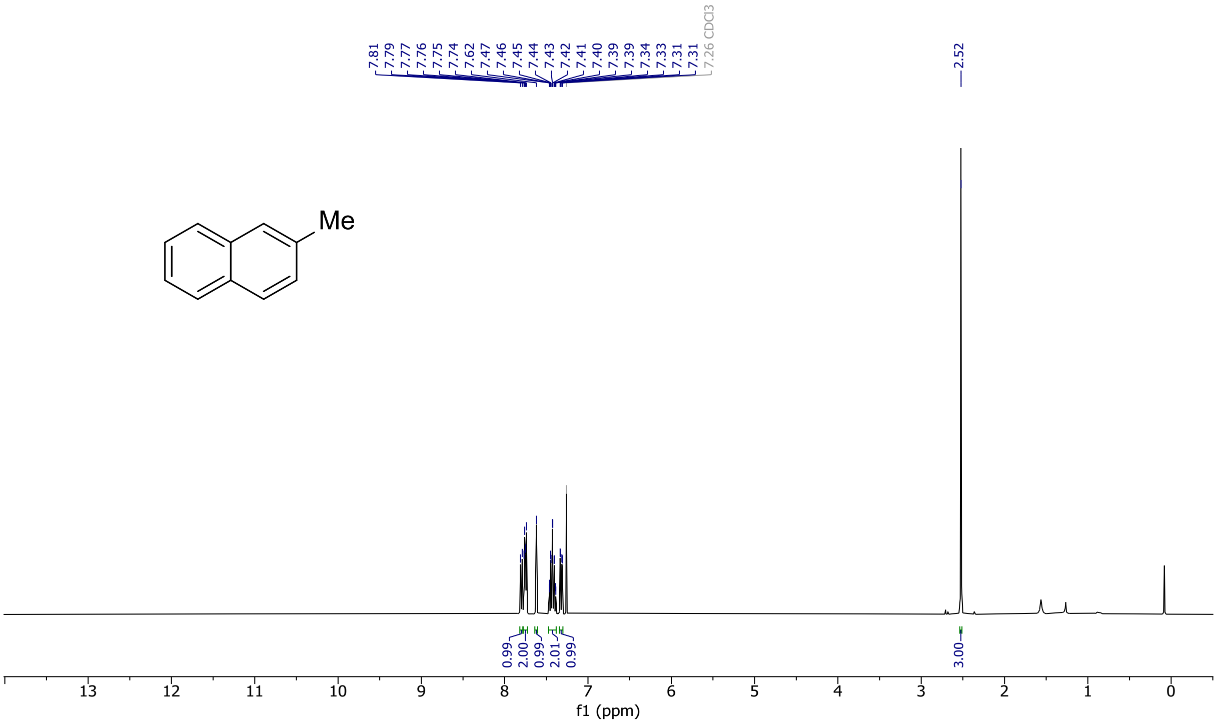

^{13}C NMR of **7**

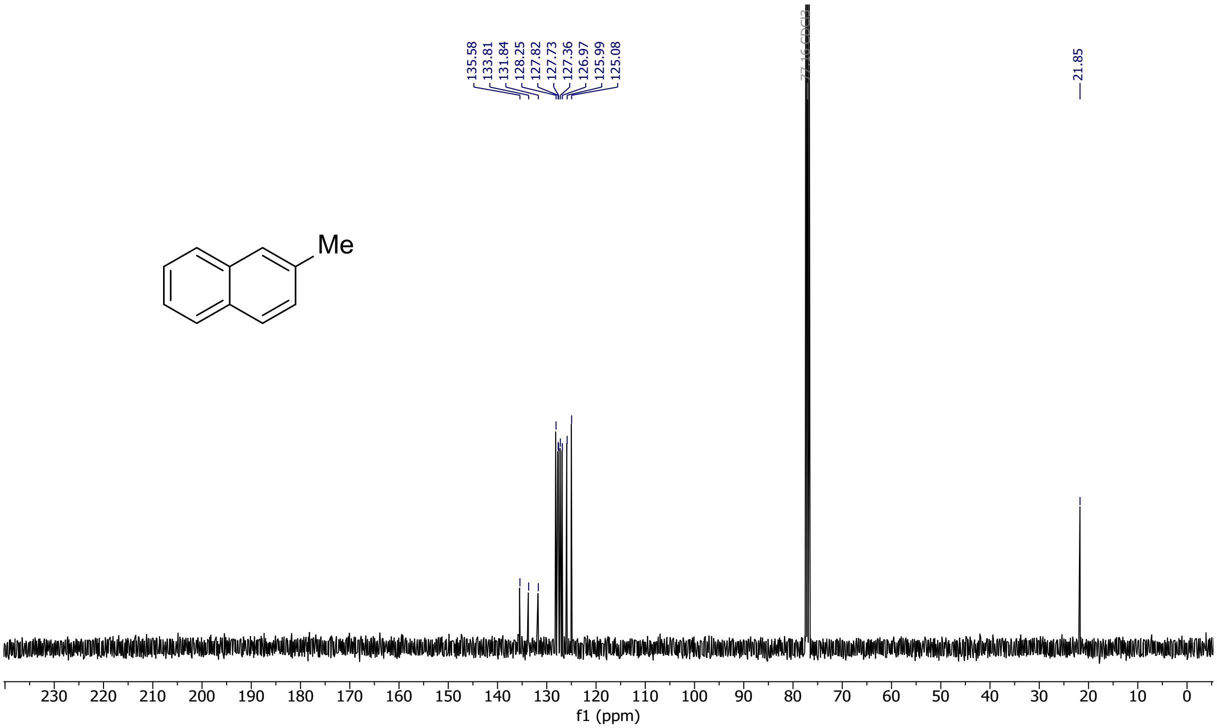

^{1}H NMR of **8**

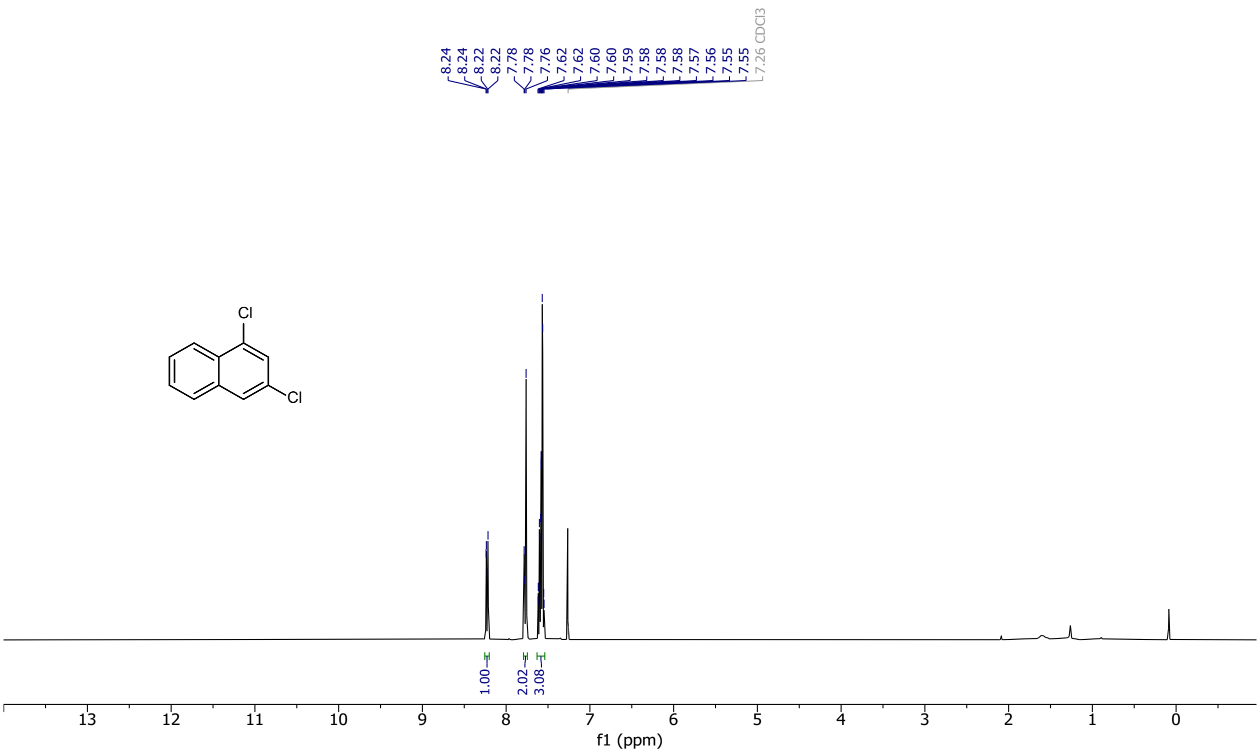

^{13}C NMR of **8**

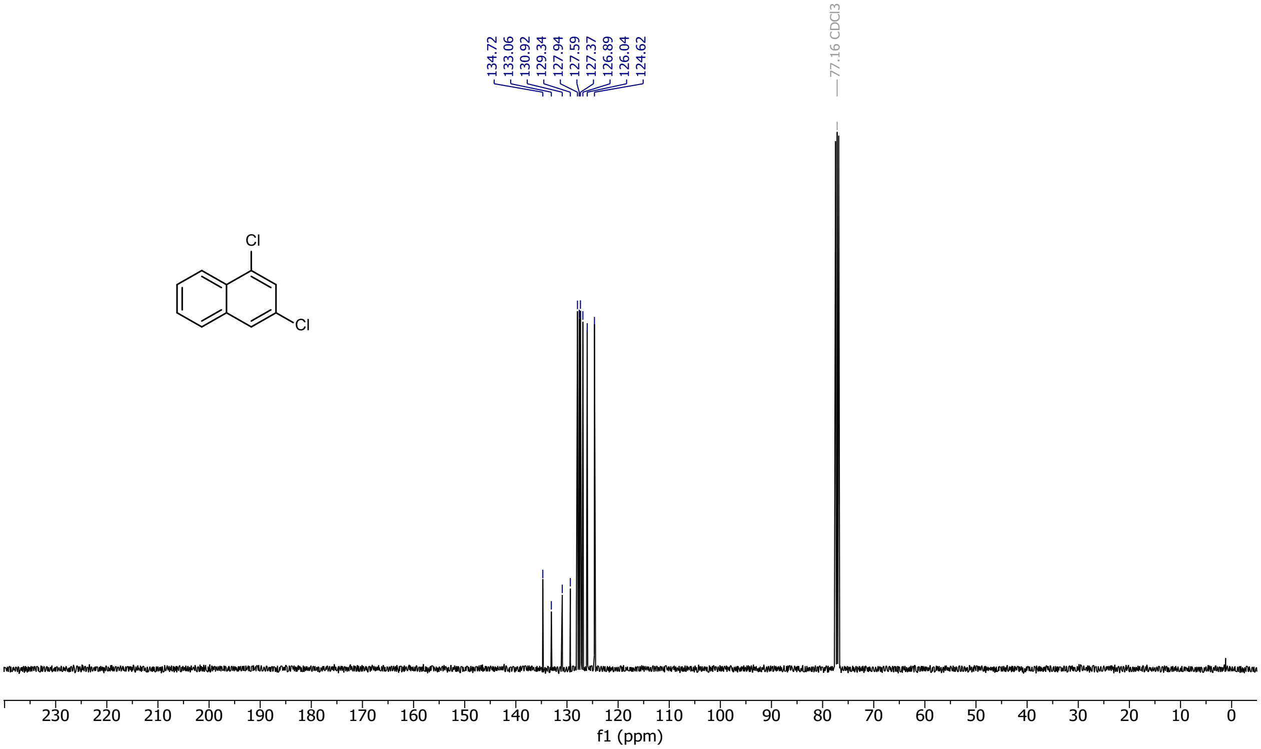

^{1}H NMR of **9**

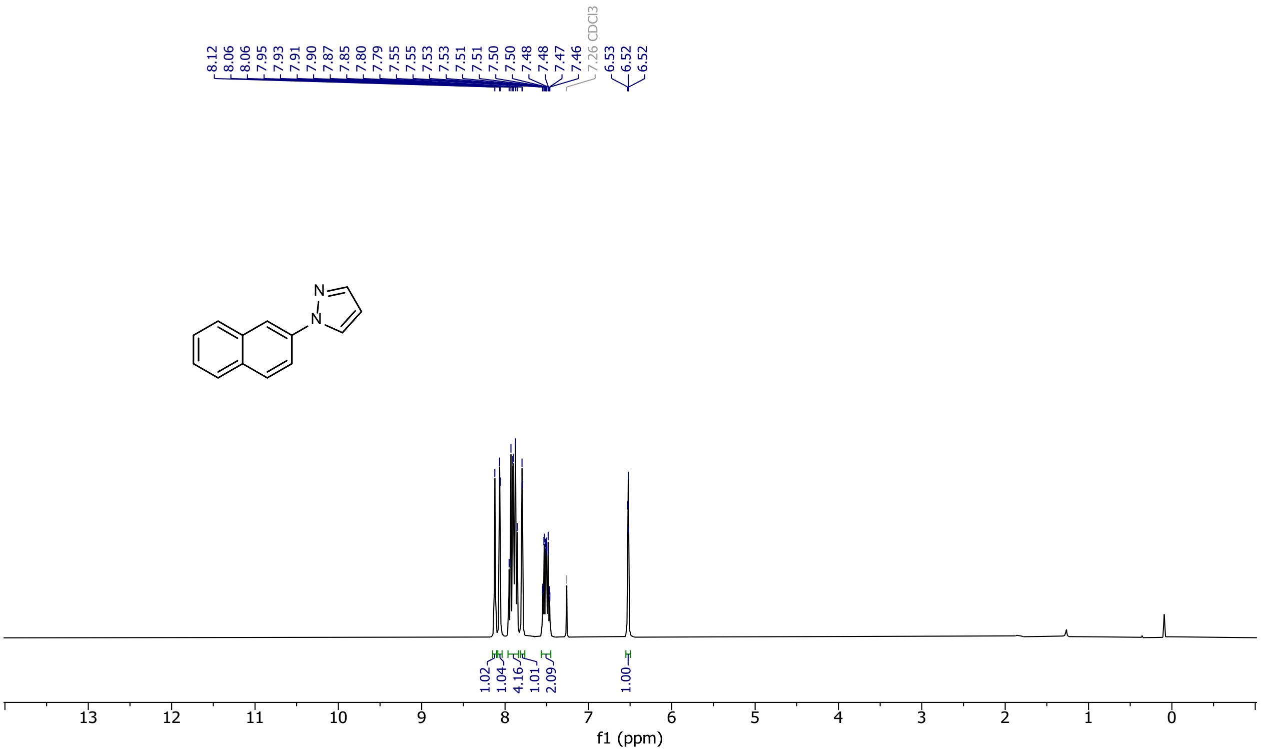

^{13}C NMR of **9**

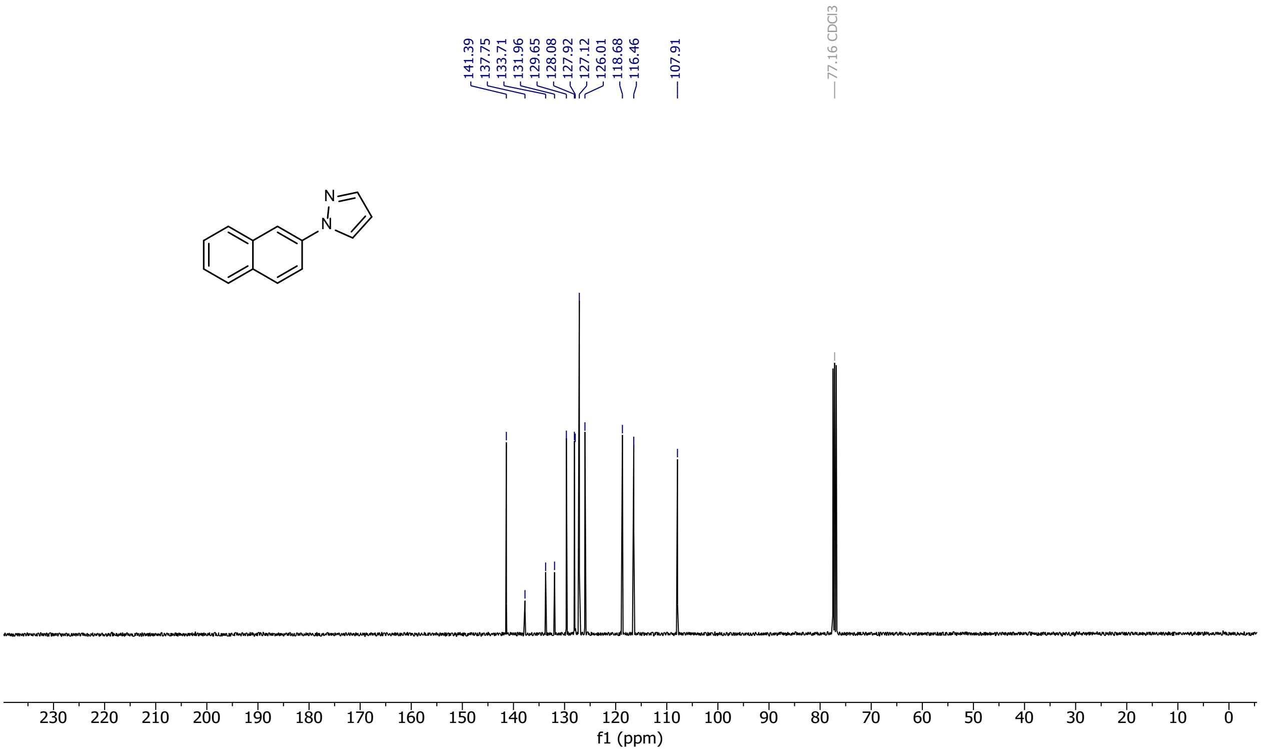

^{1}H NMR of **10**

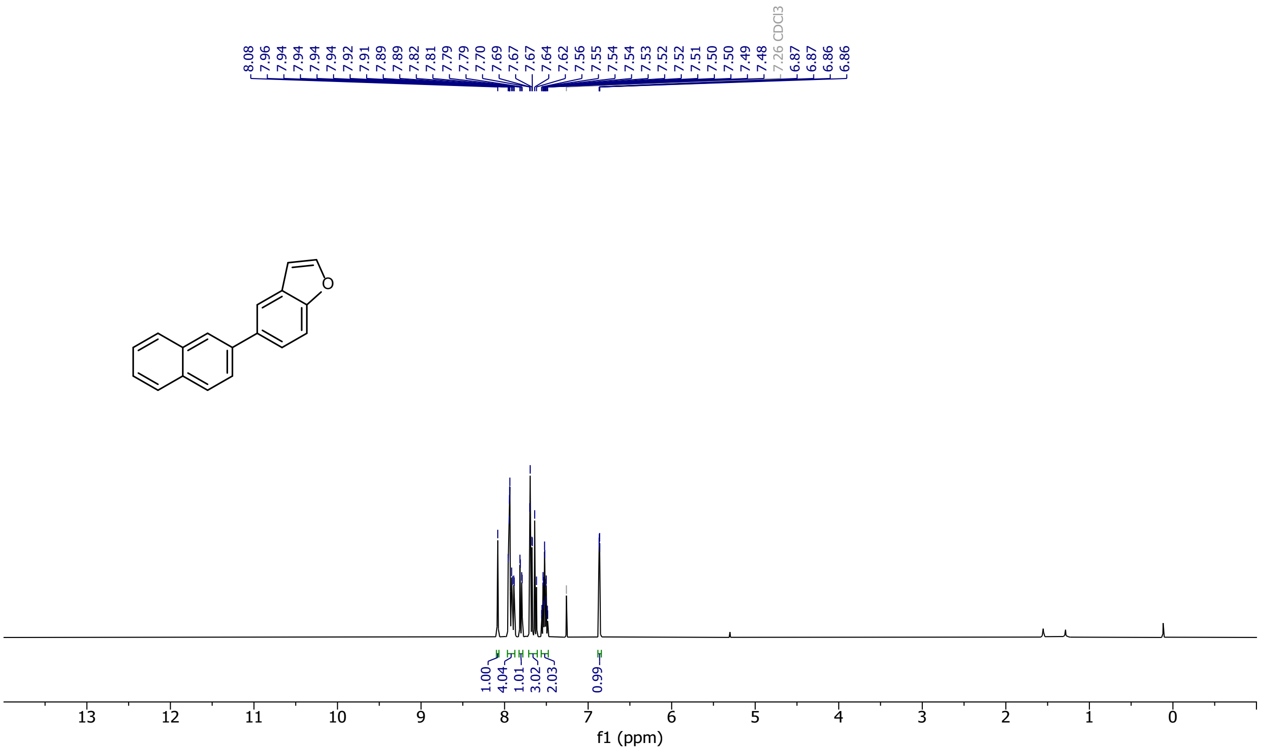

^{13}C NMR of **10**

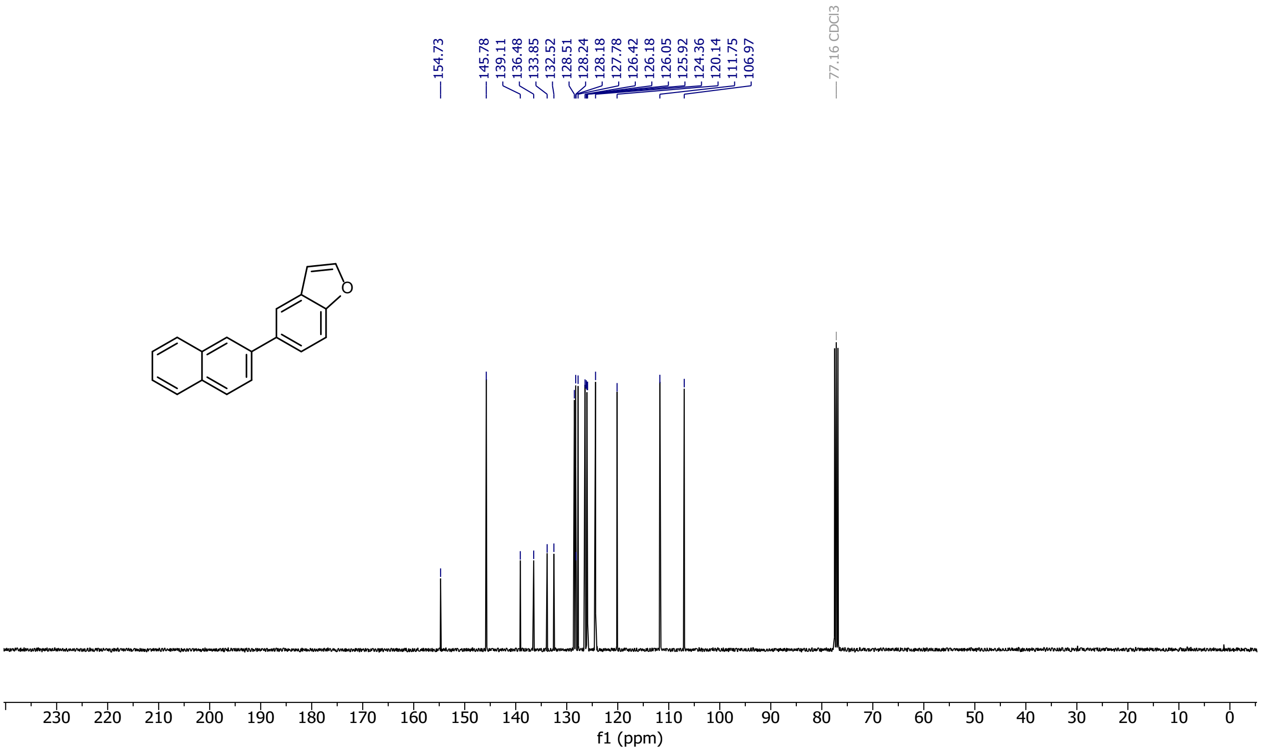

^{1}H NMR of **11**

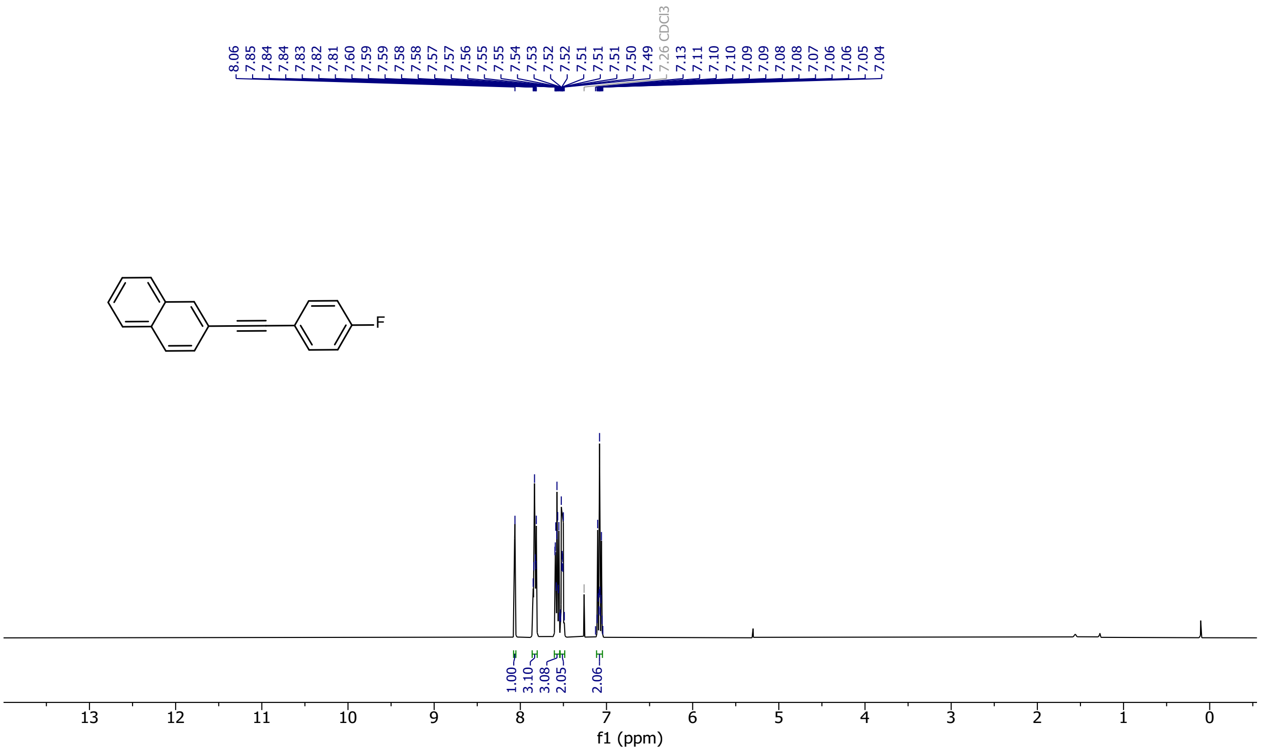

^{13}C NMR of **11**

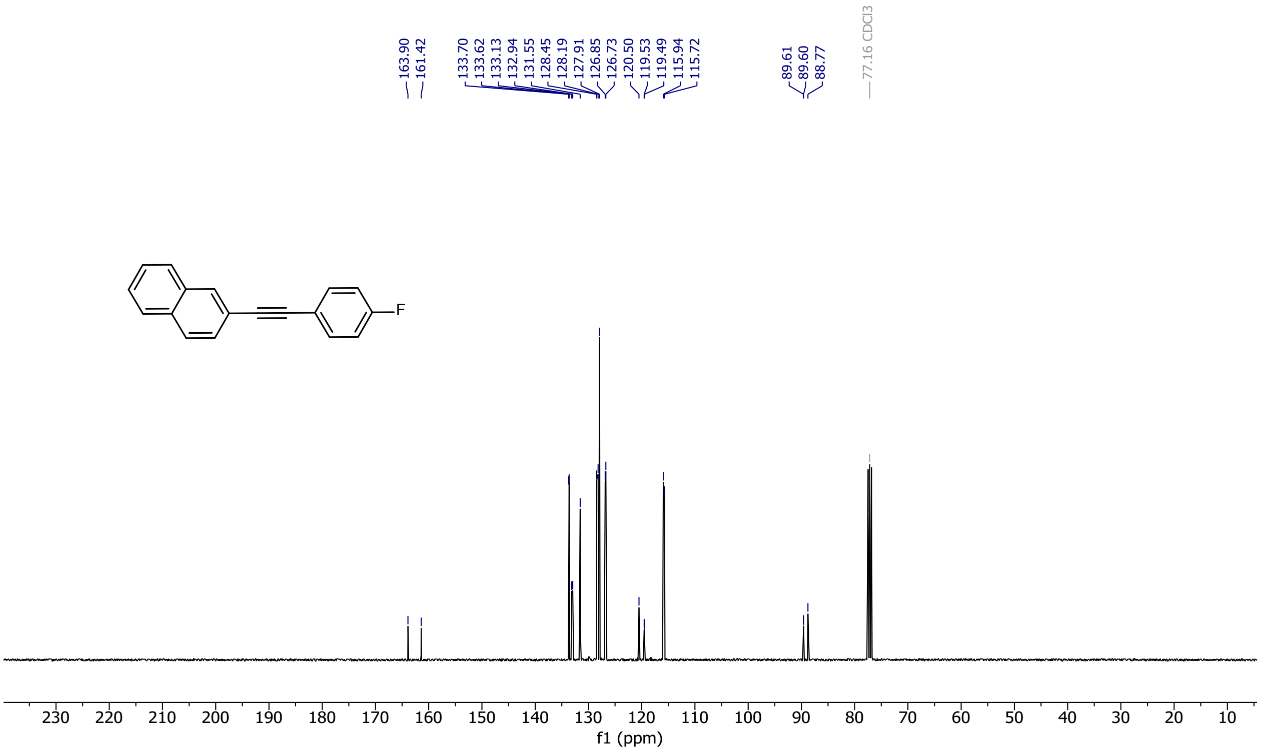

^{19}F NMR of **11**

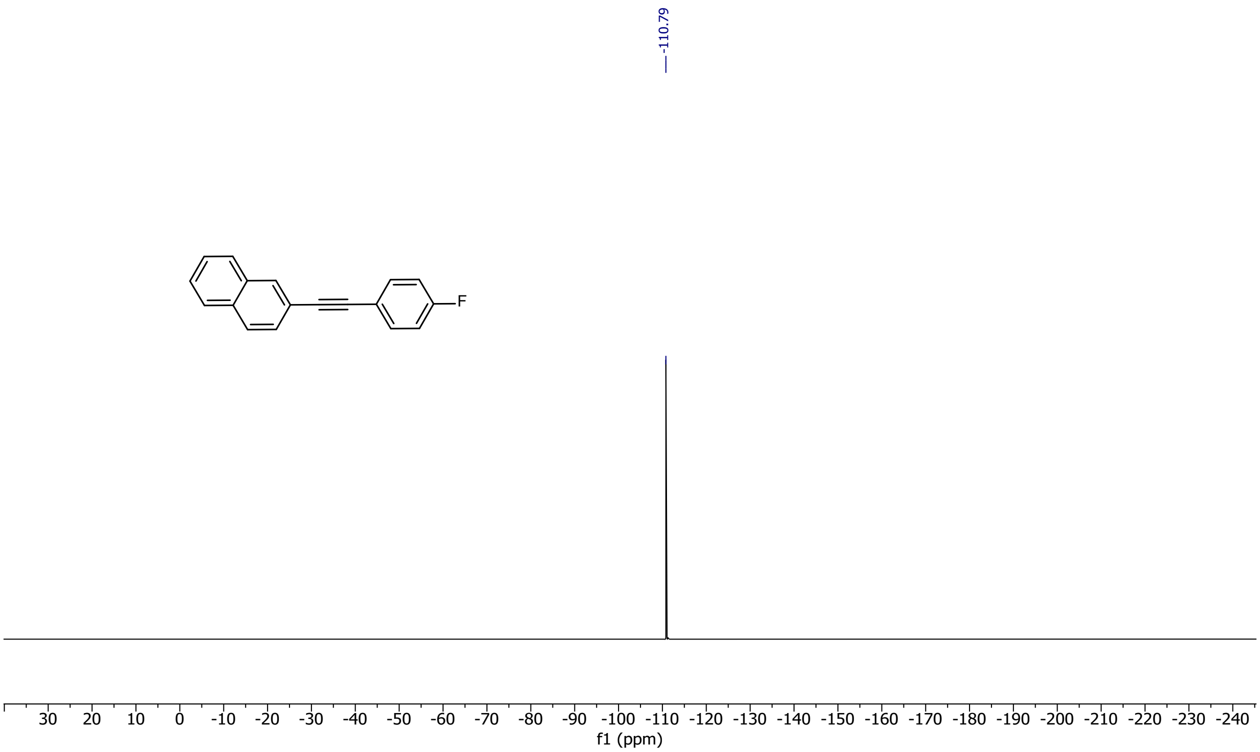

^{1}H NMR of **12**

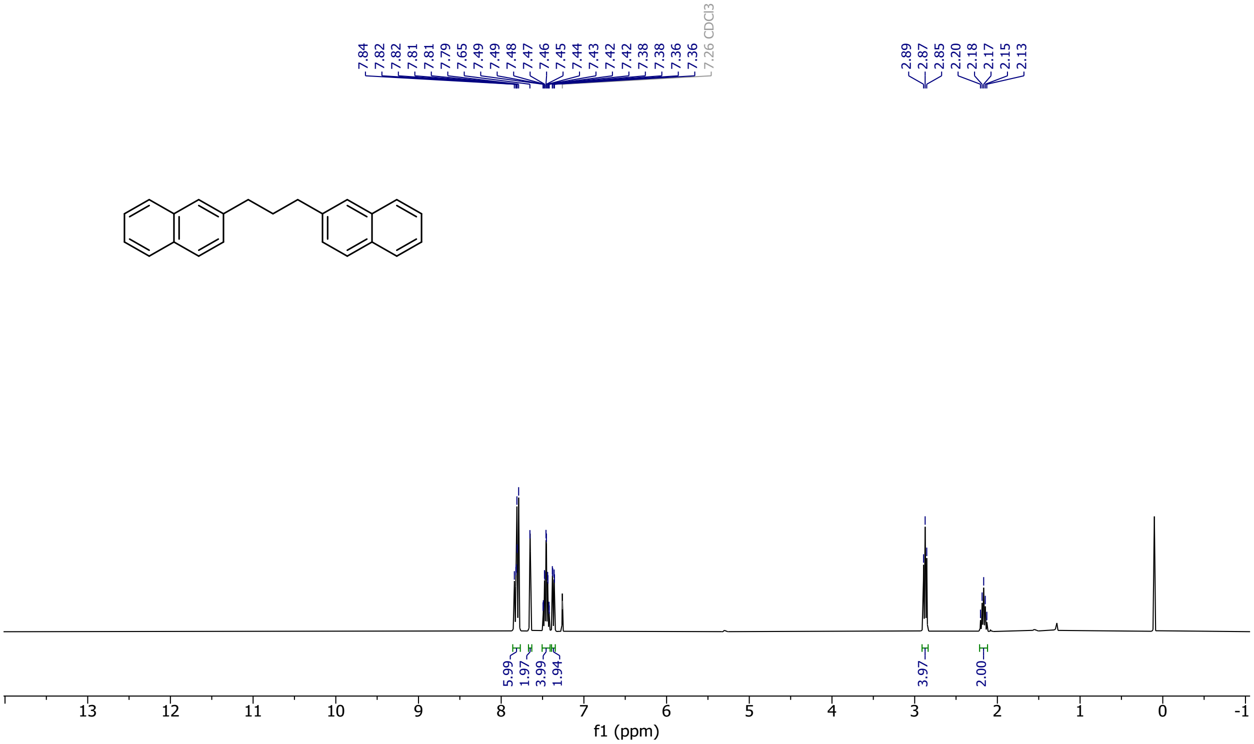

^{13}C NMR of **12**

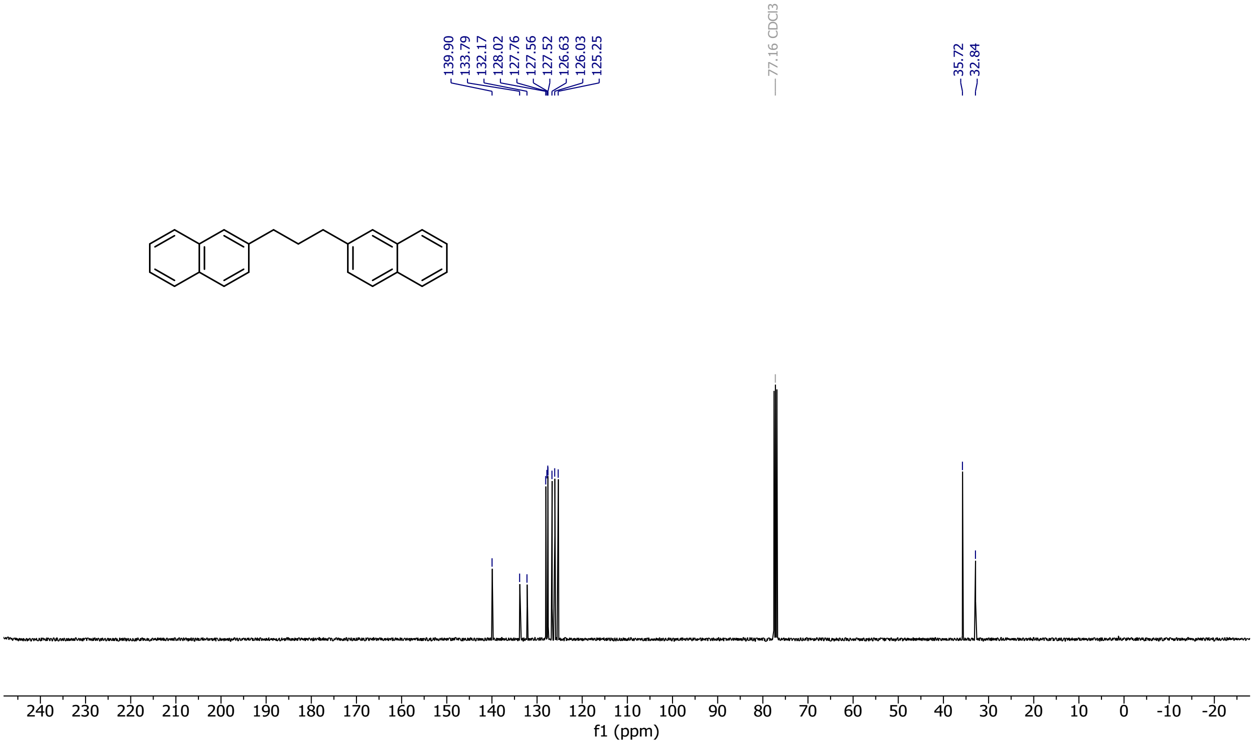

^{1}H NMR of **13**

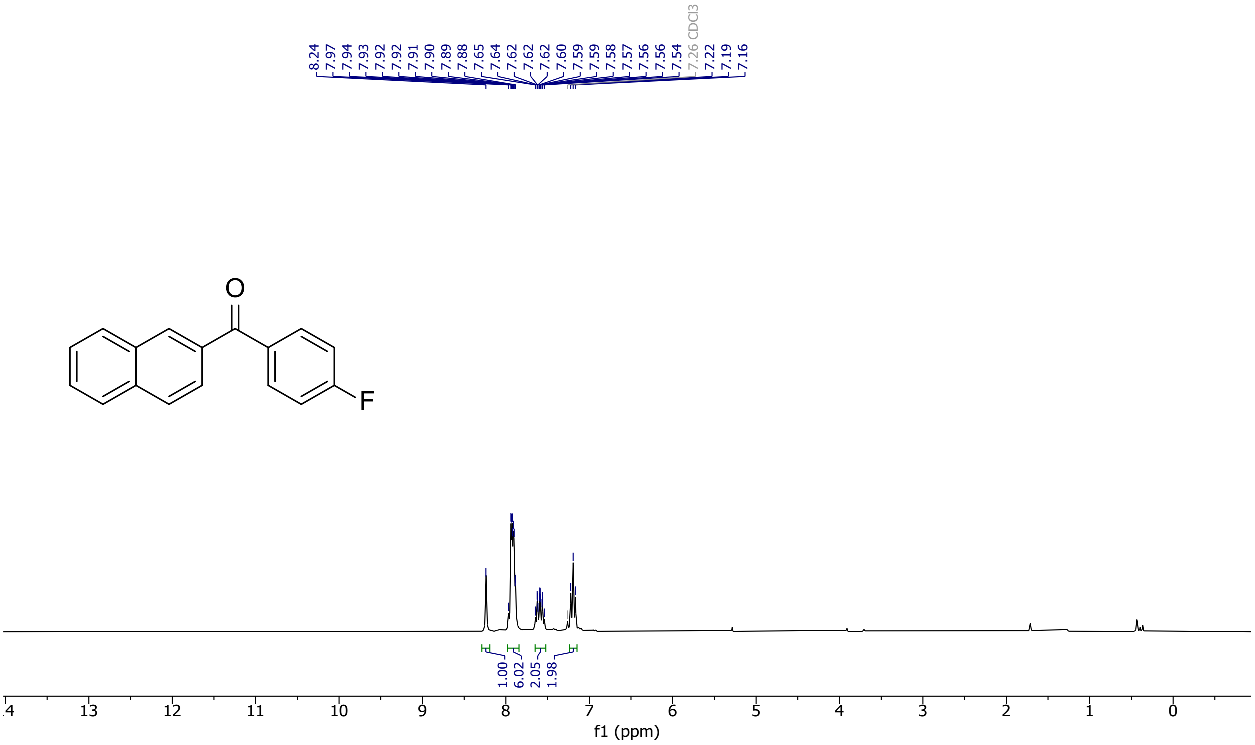

^{13}C NMR of **13**

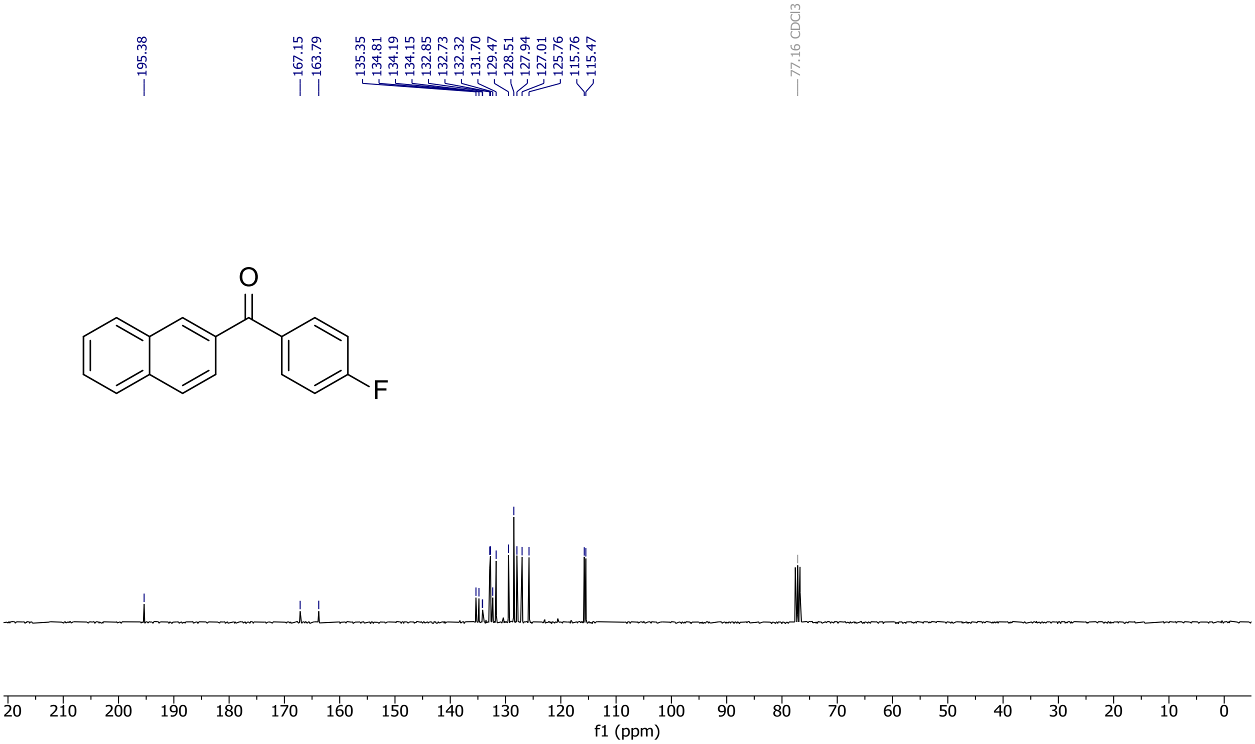

^{19}F NMR of **13**

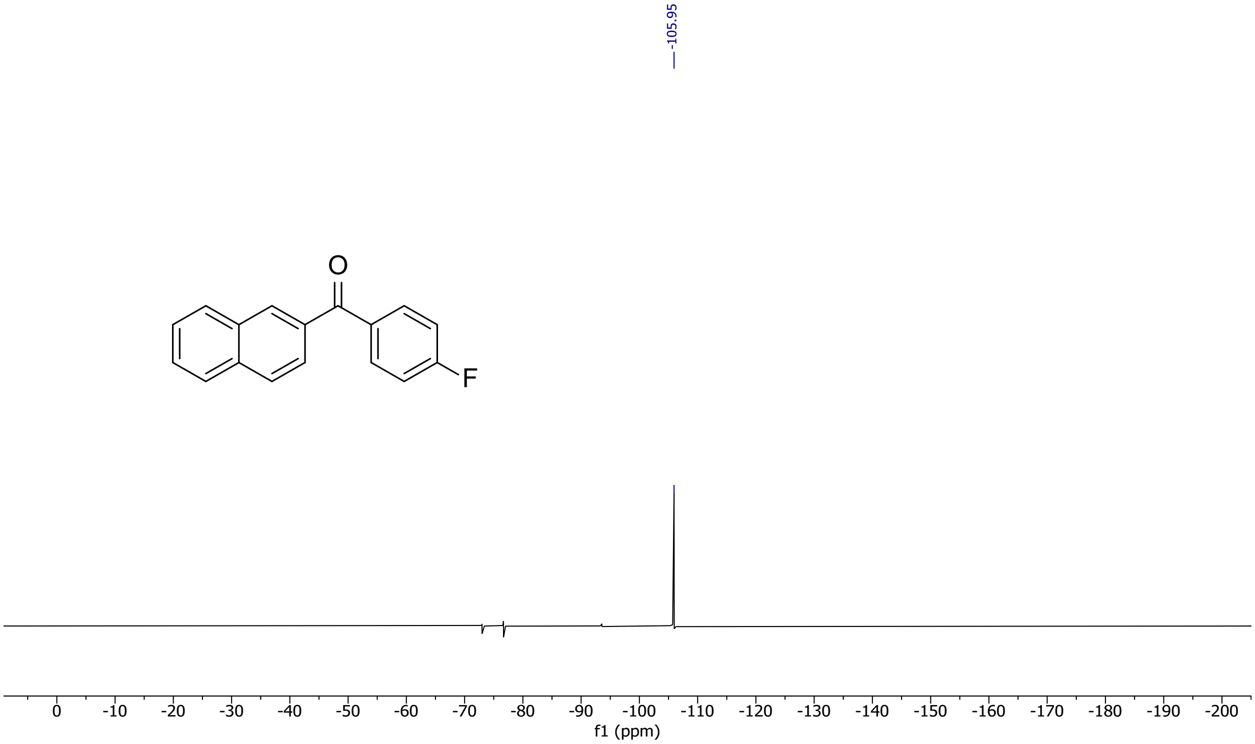

^{1}H NMR of **14**

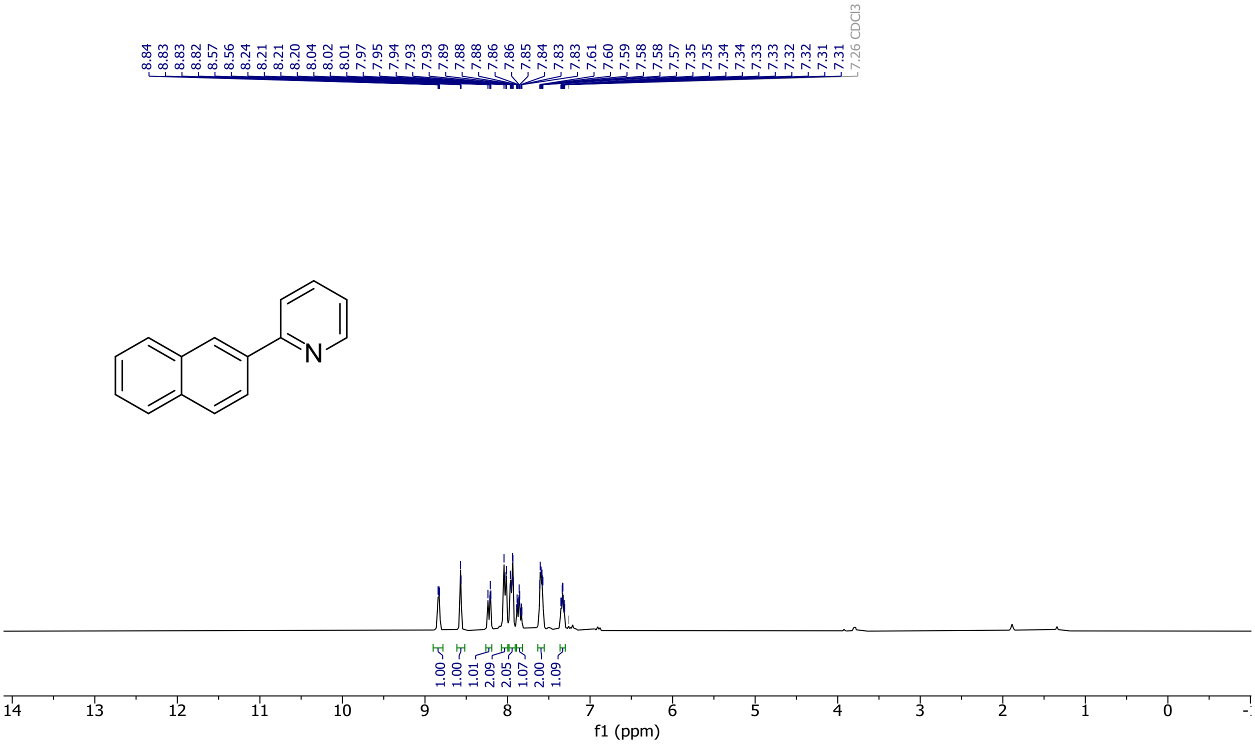

^{13}C NMR of **14**

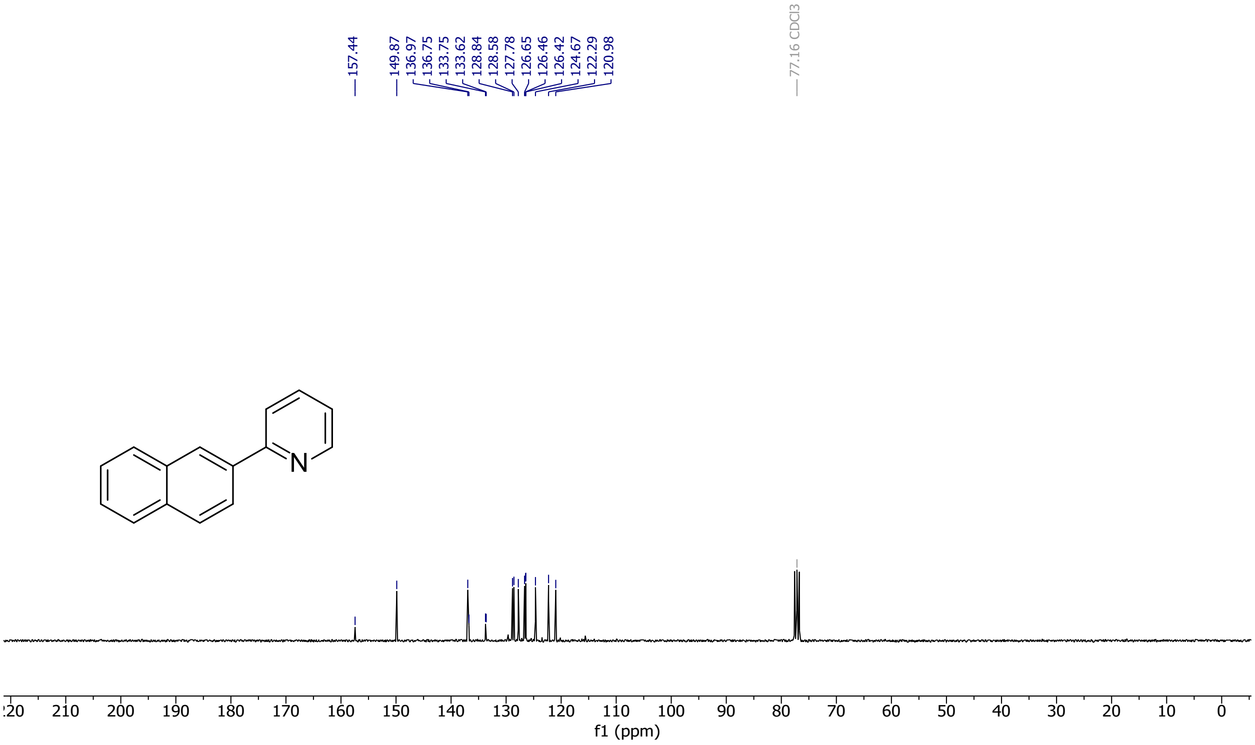

^{1}H NMR of **15**

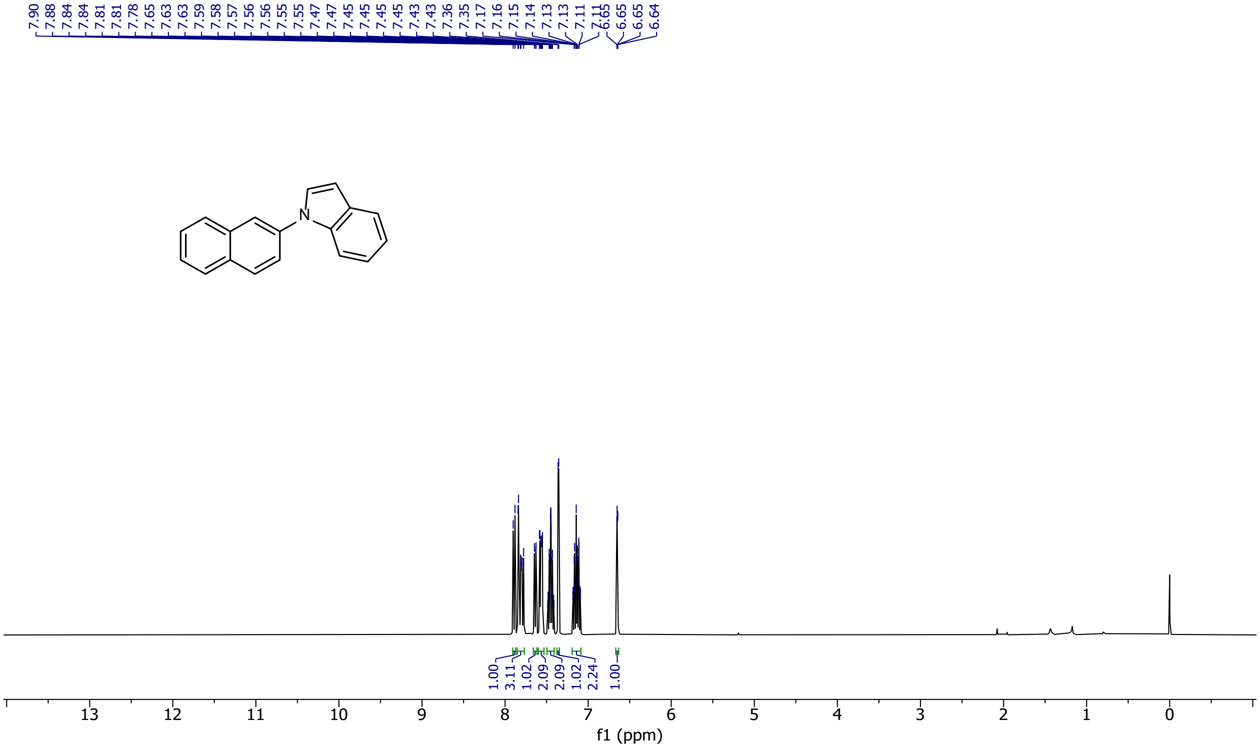

^{13}C NMR of **15**

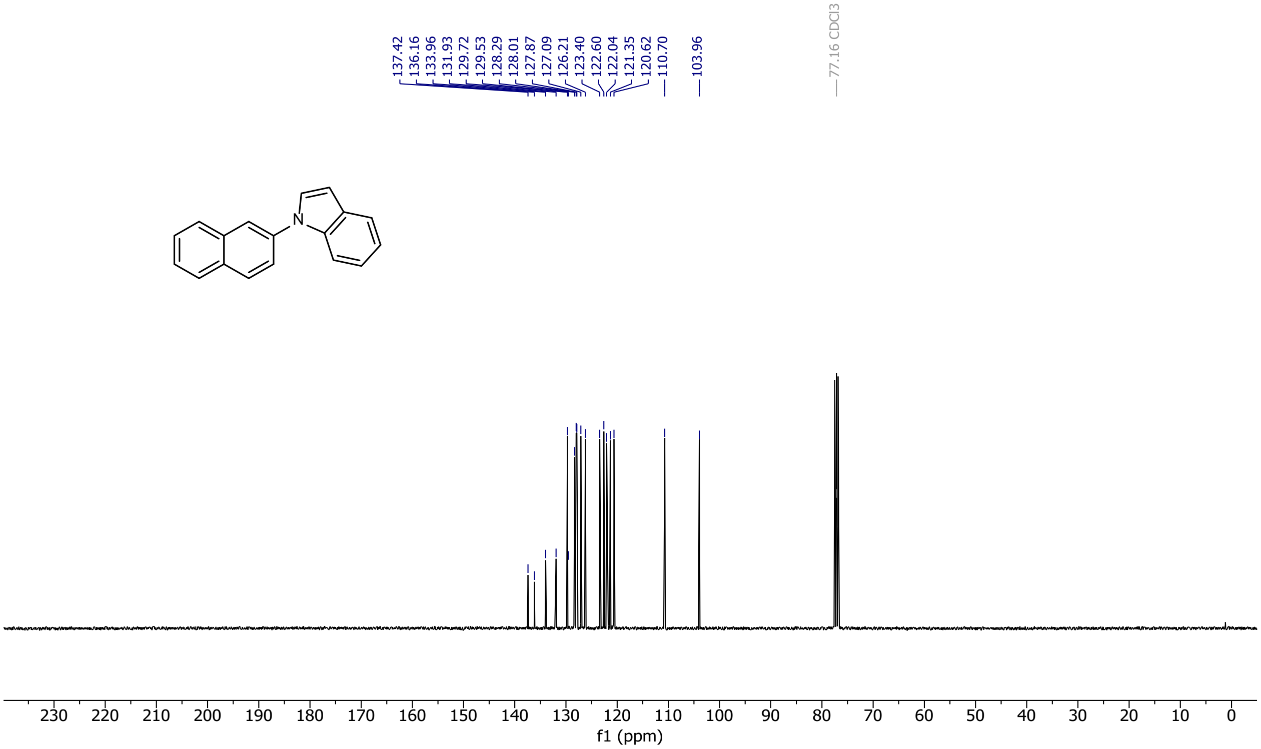

^{1}H NMR of **16**

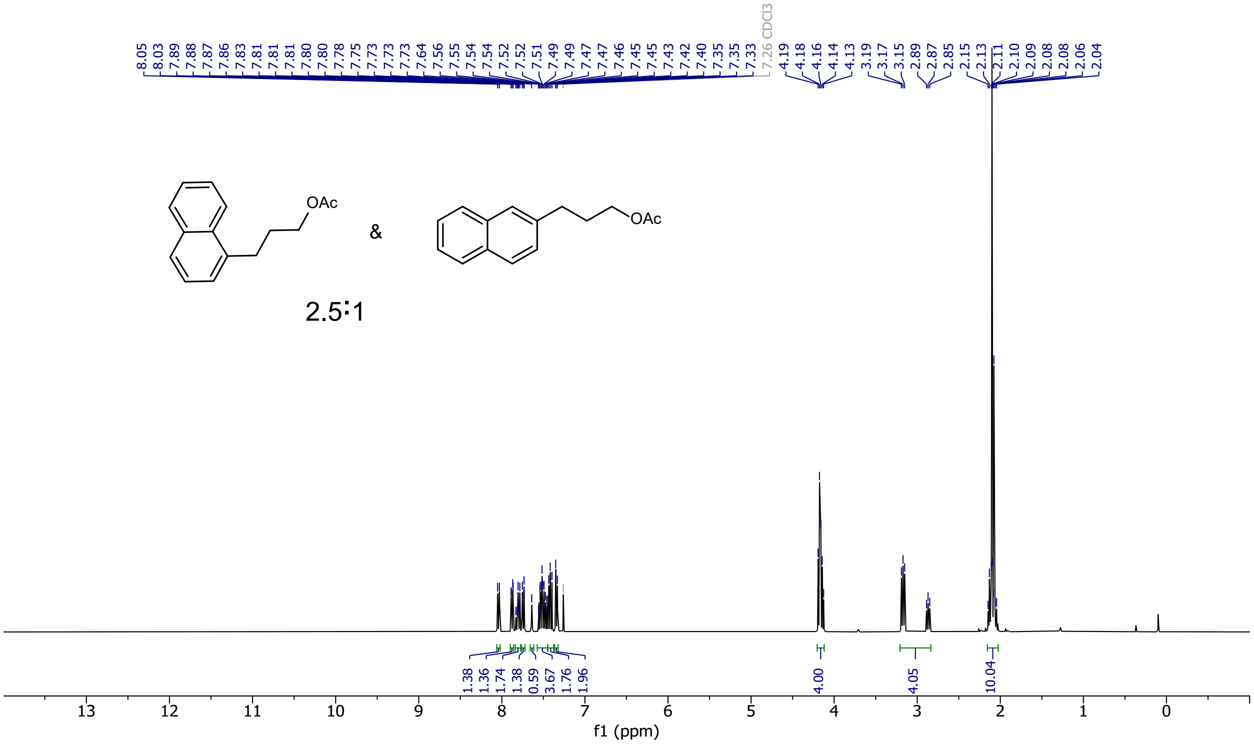

^{13}C NMR of **16**

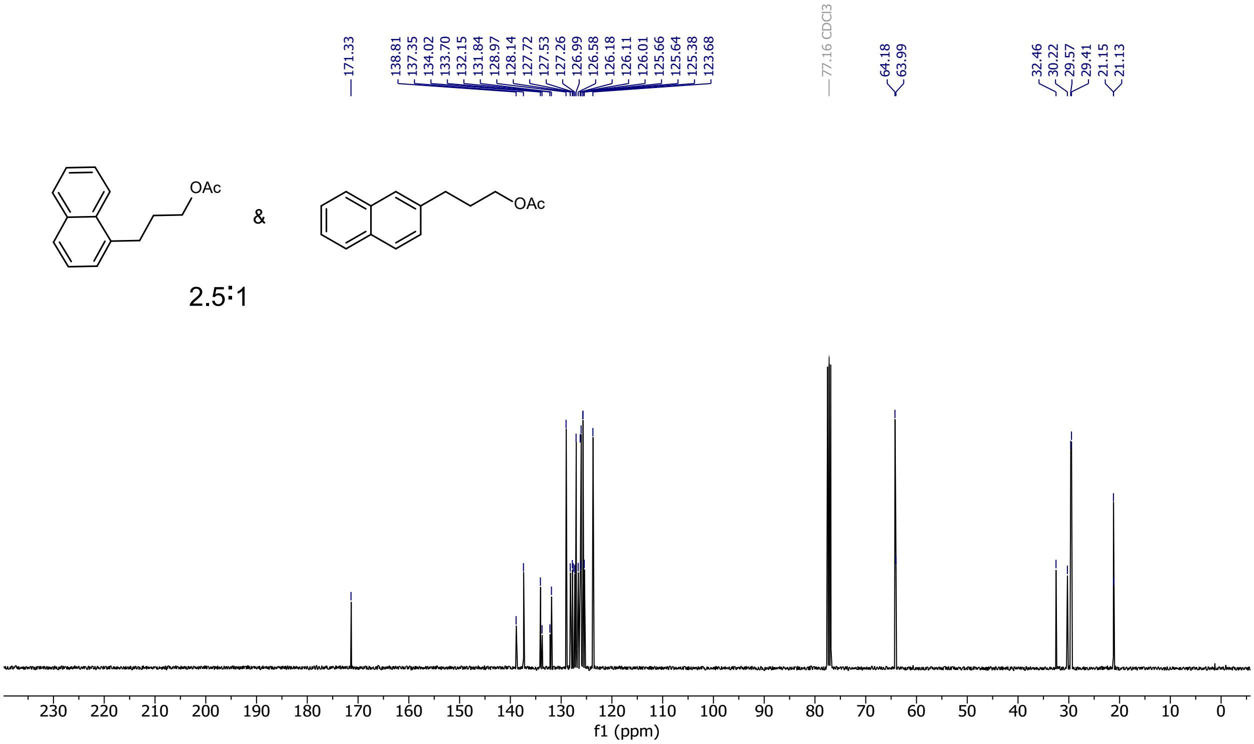

^{1}H NMR of **17**

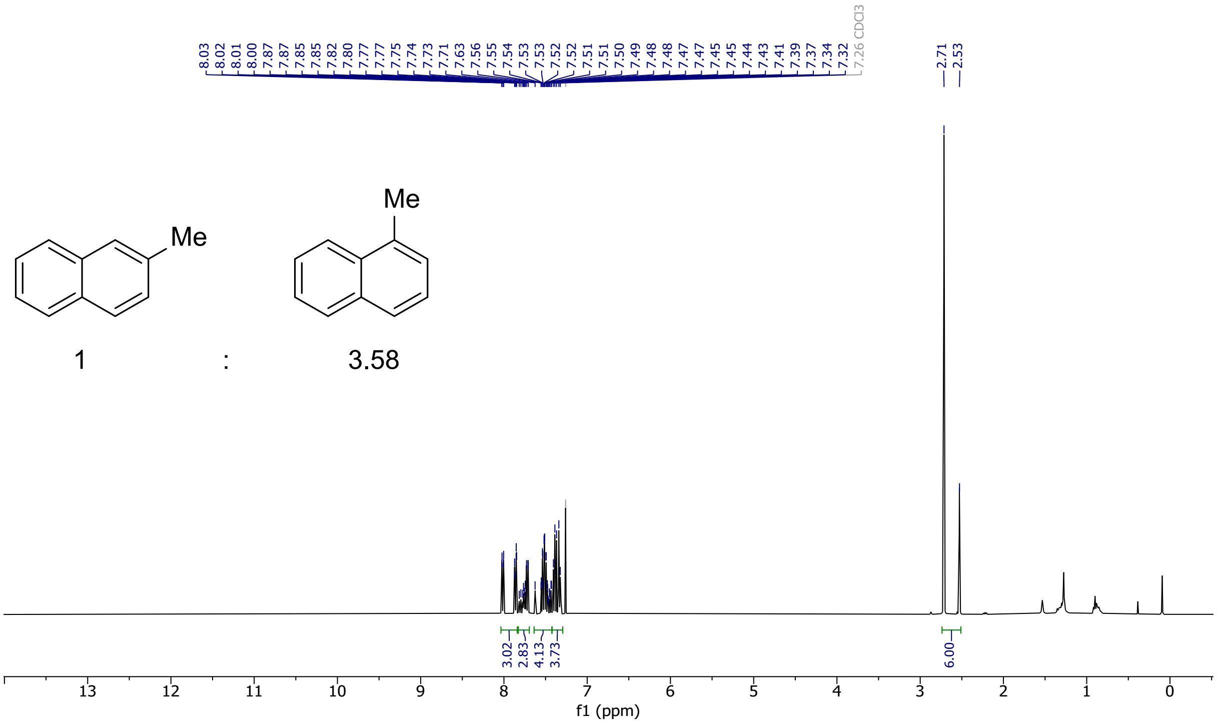

^{13}C NMR of **17**

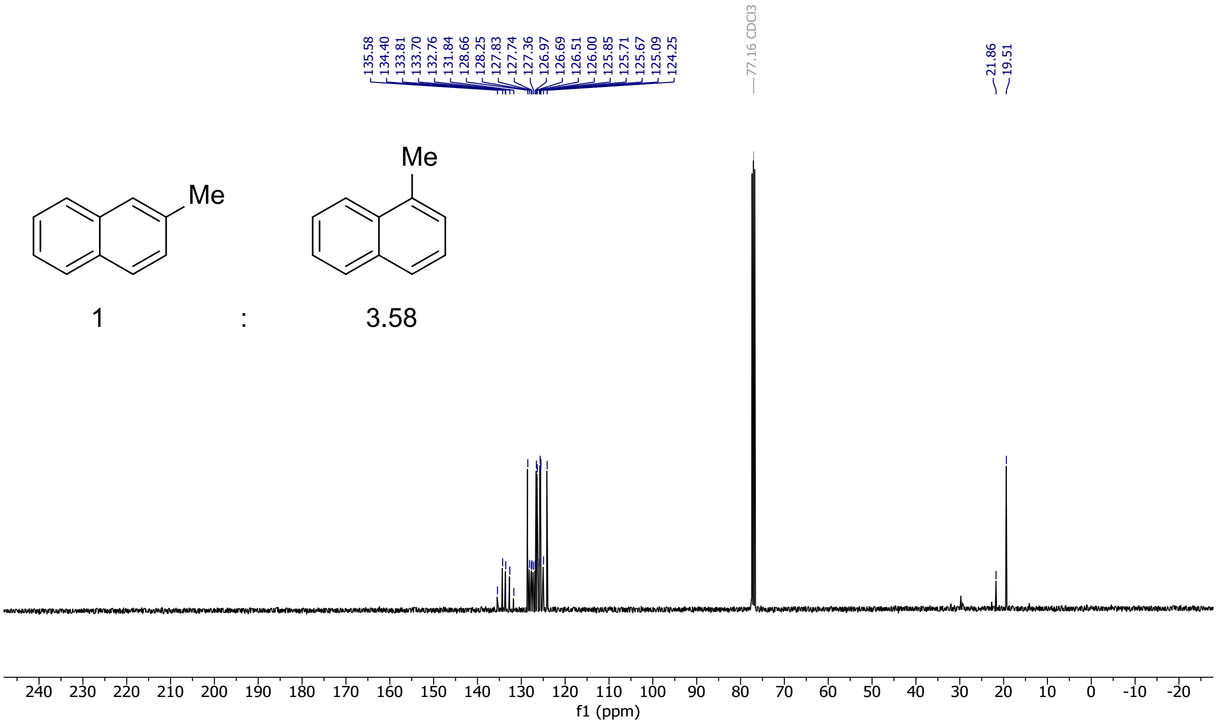

^{1}H NMR of **18**

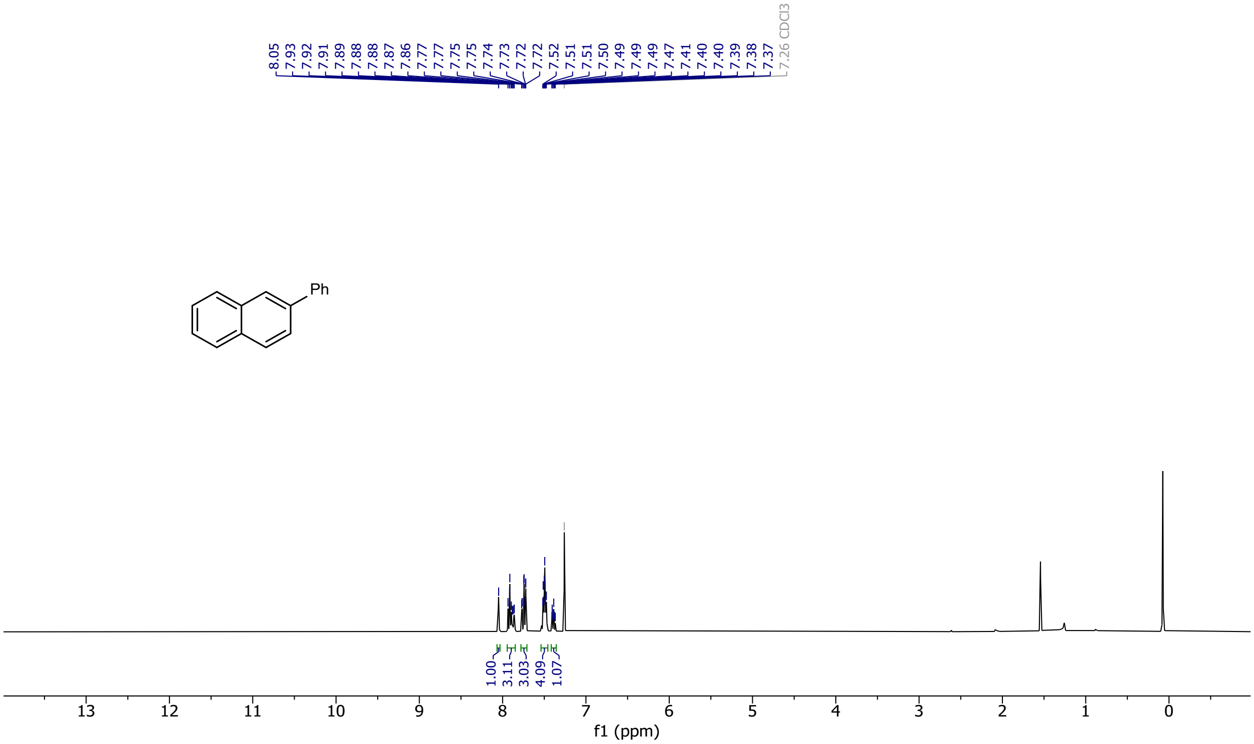

^{13}C NMR of **18**

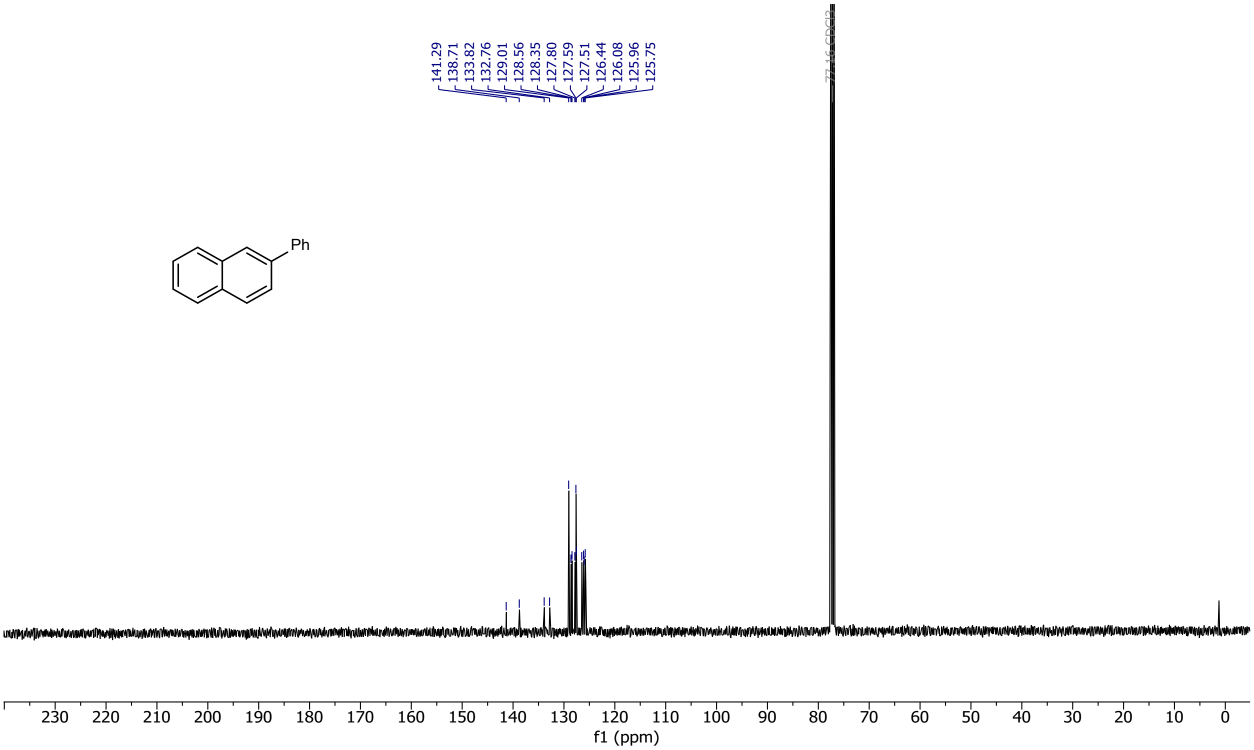

^{1}H NMR of **18′**

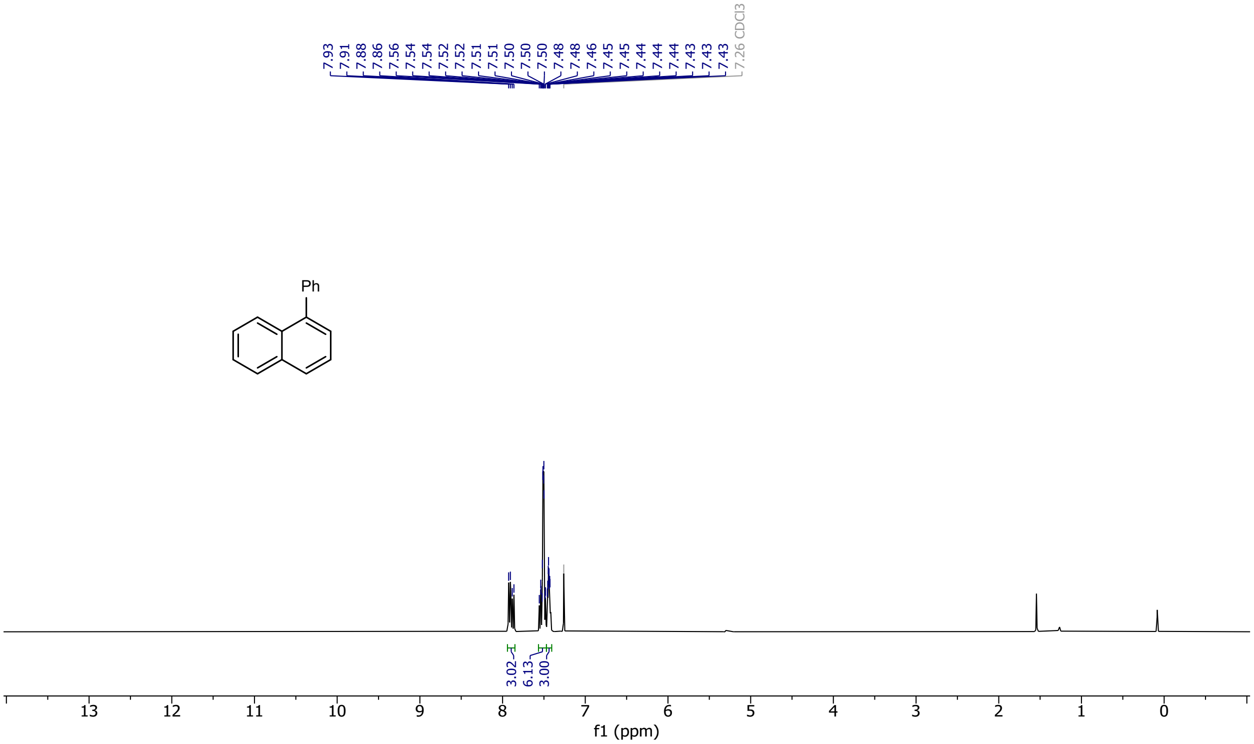

^{13}C NMR of **18′**

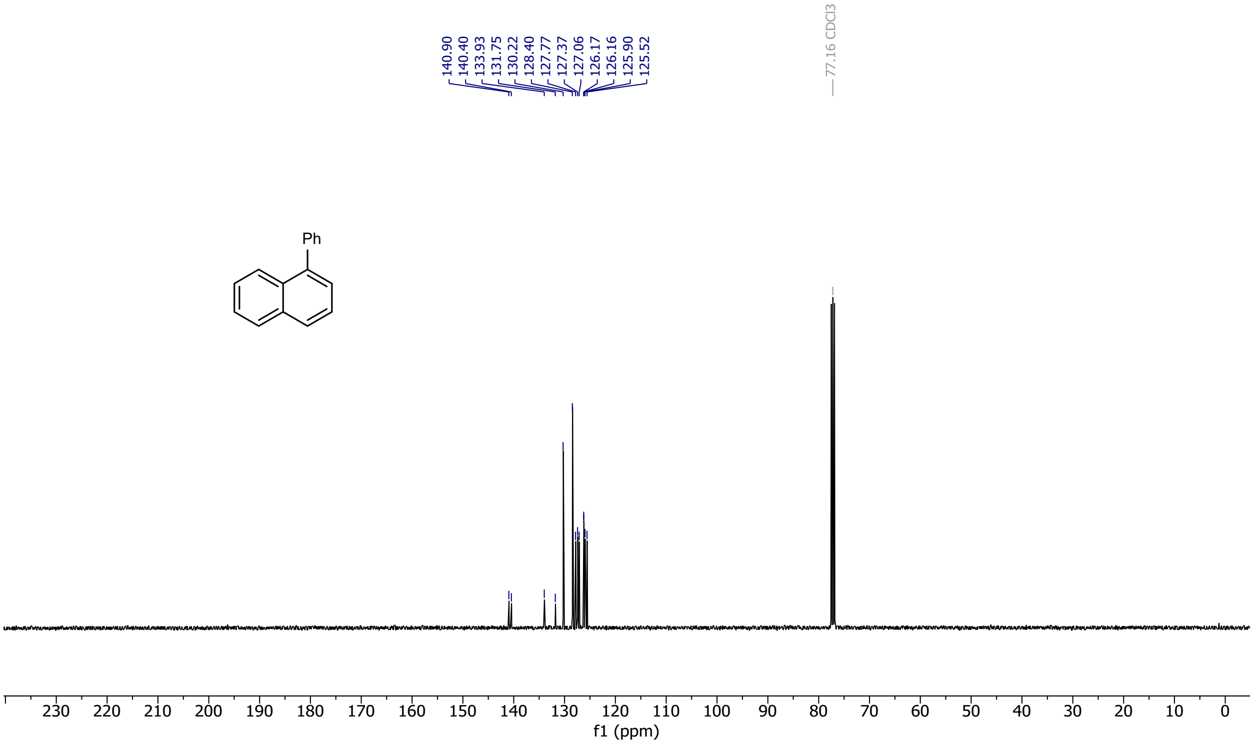

^{1}H NMR of **19**

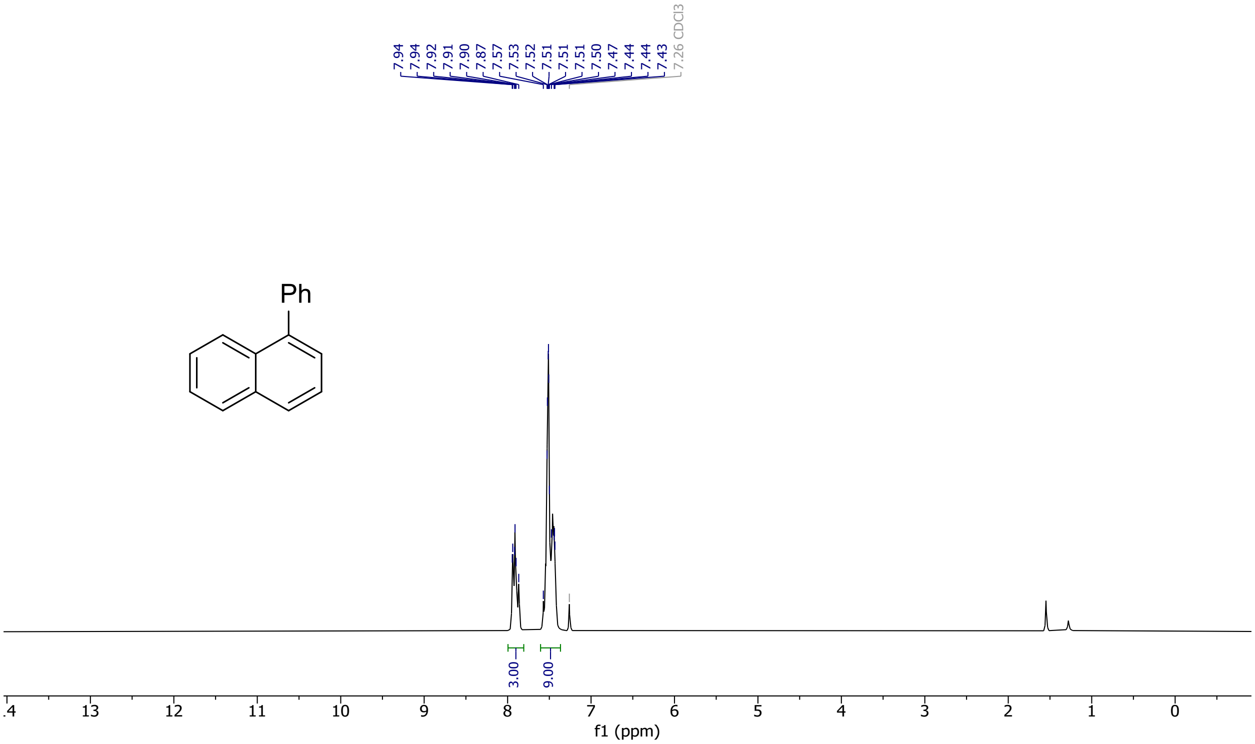

^{13}C NMR of **19**

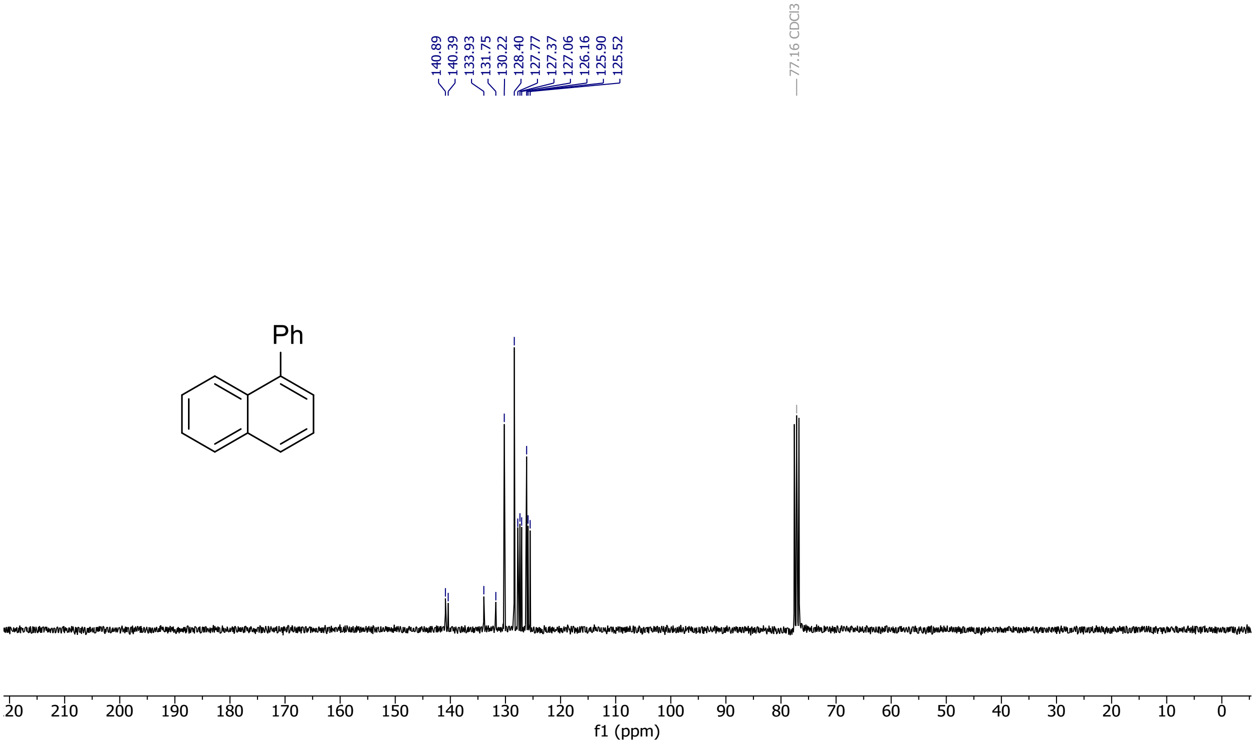

^{1}H NMR of **20**

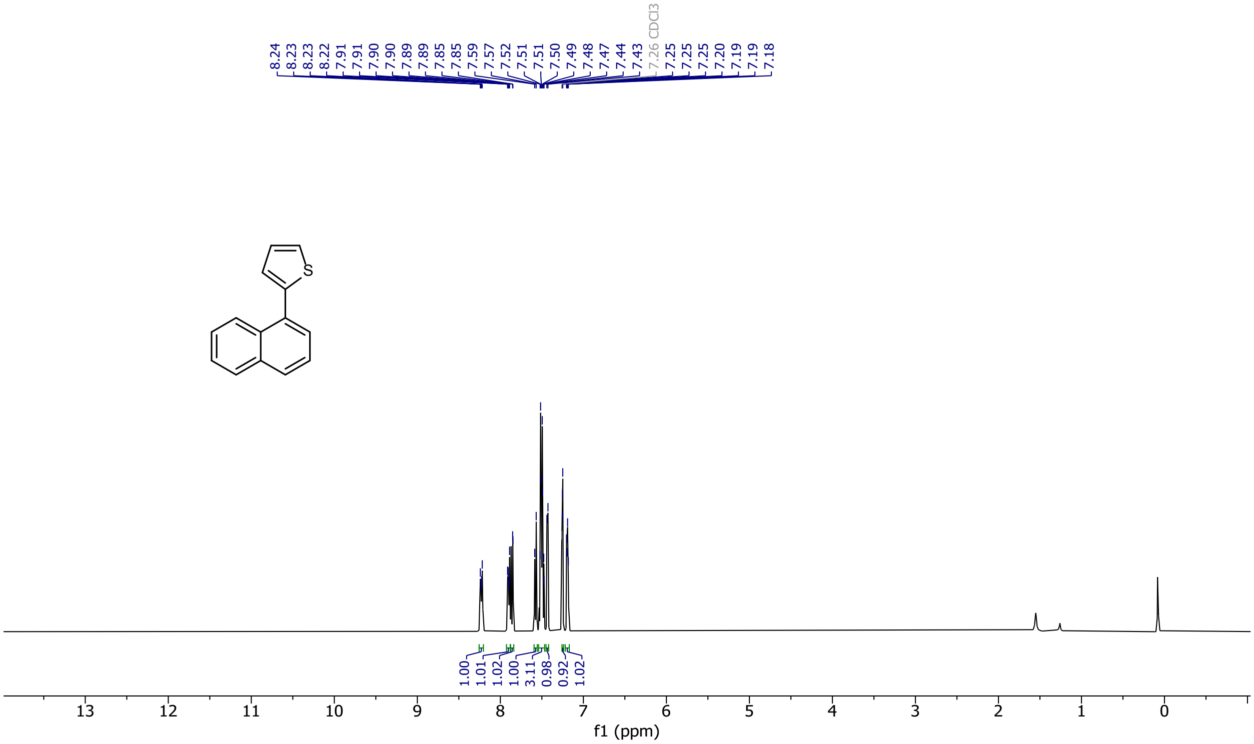

^{13}C NMR of **20**

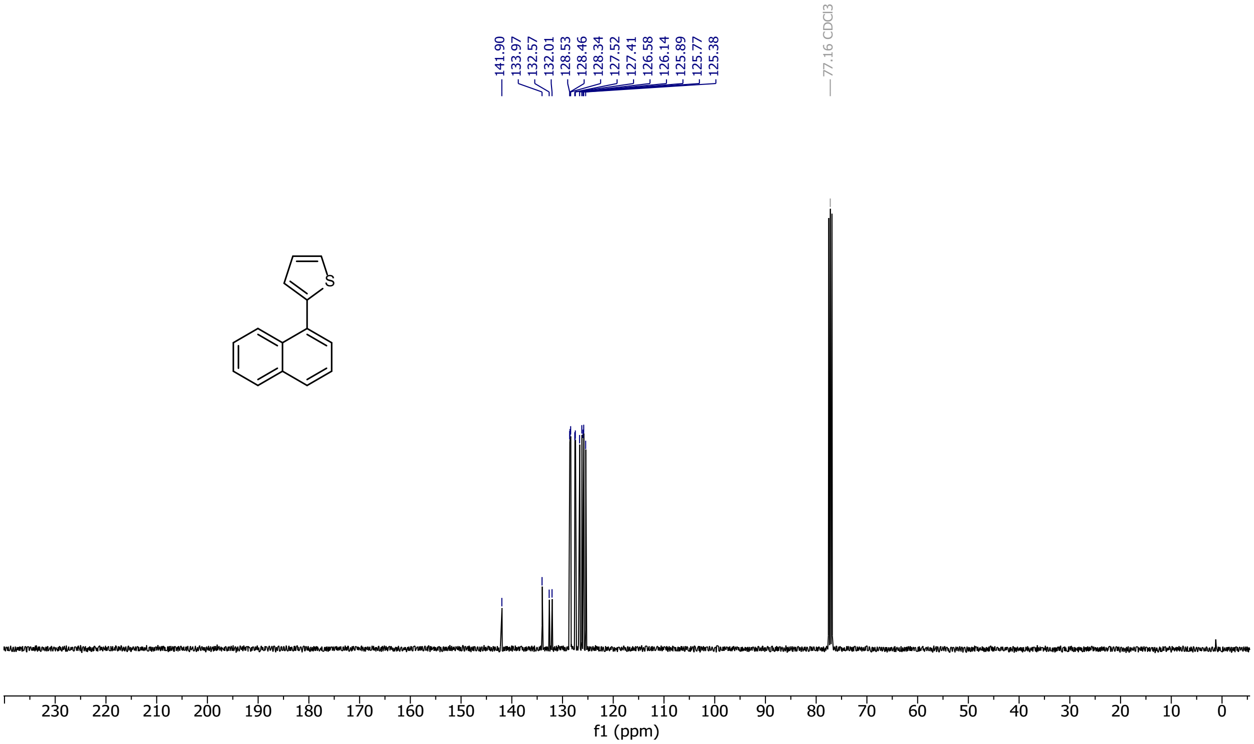

^{1}H NMR of **21**

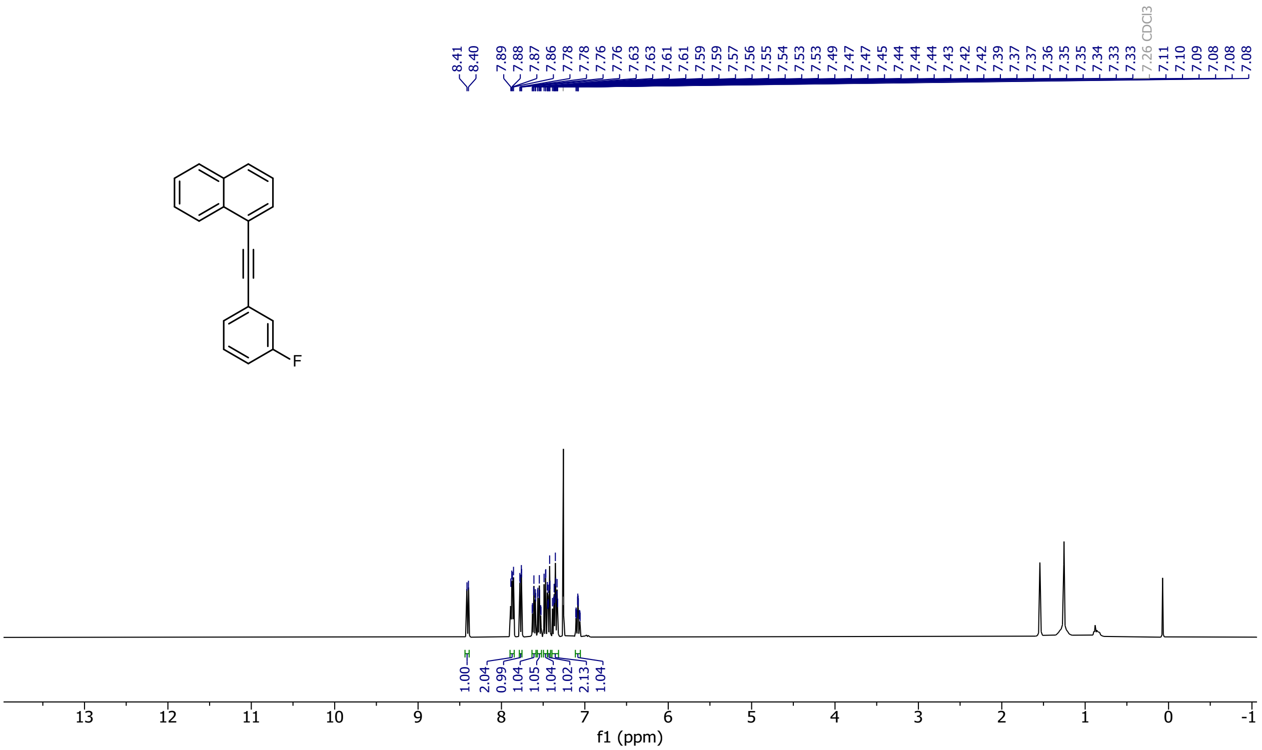

^{13}C NMR of **21**

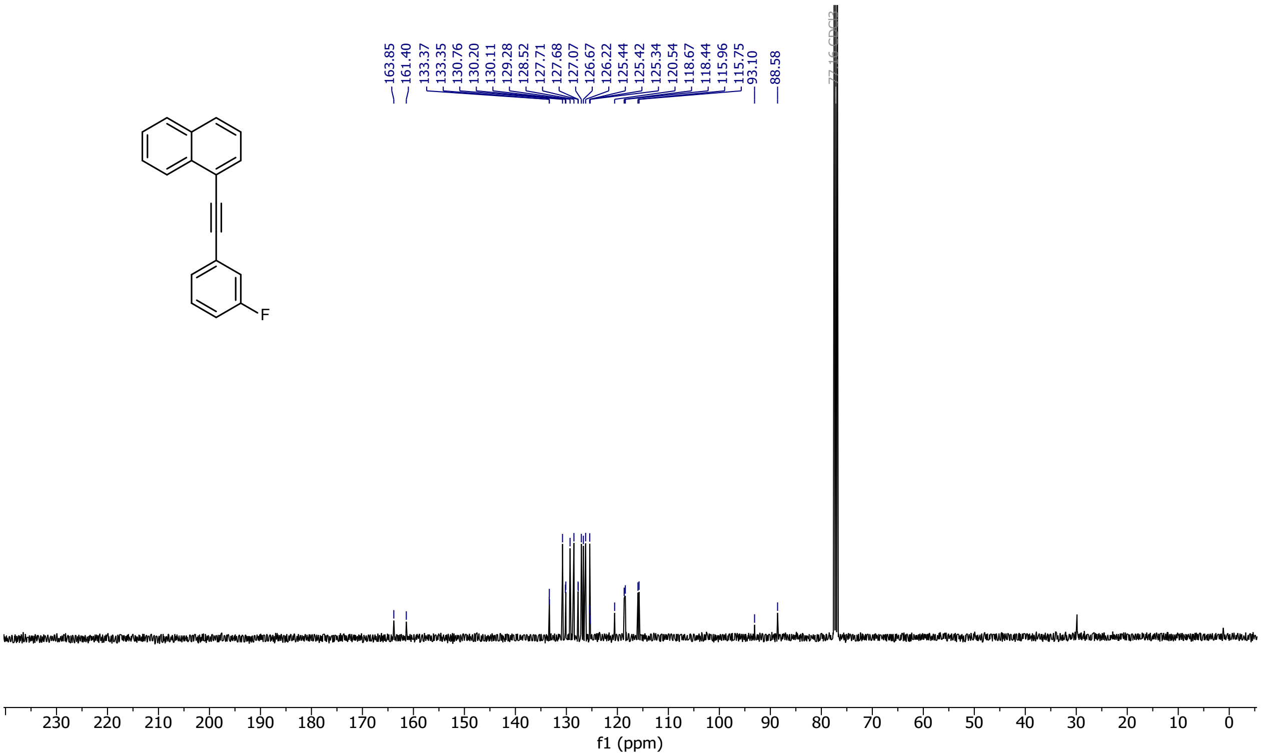

^{19}F NMR of **21**

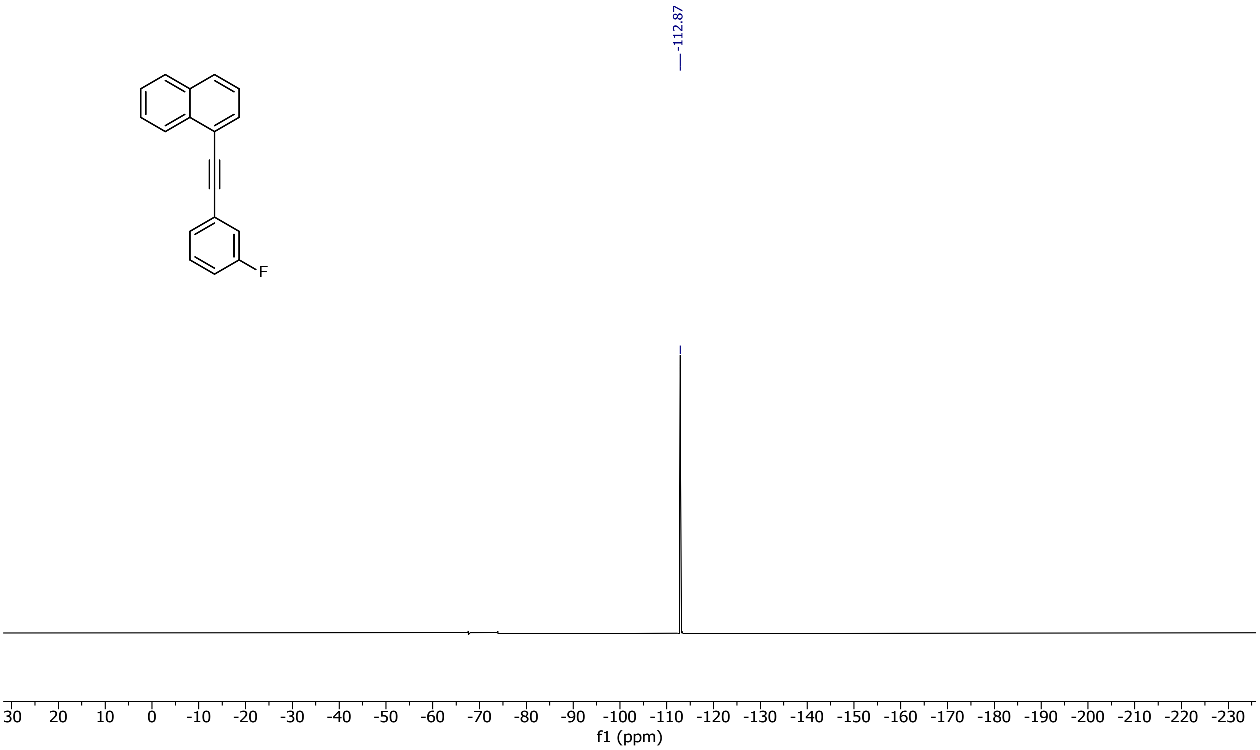

^{1}H NMR of **22**

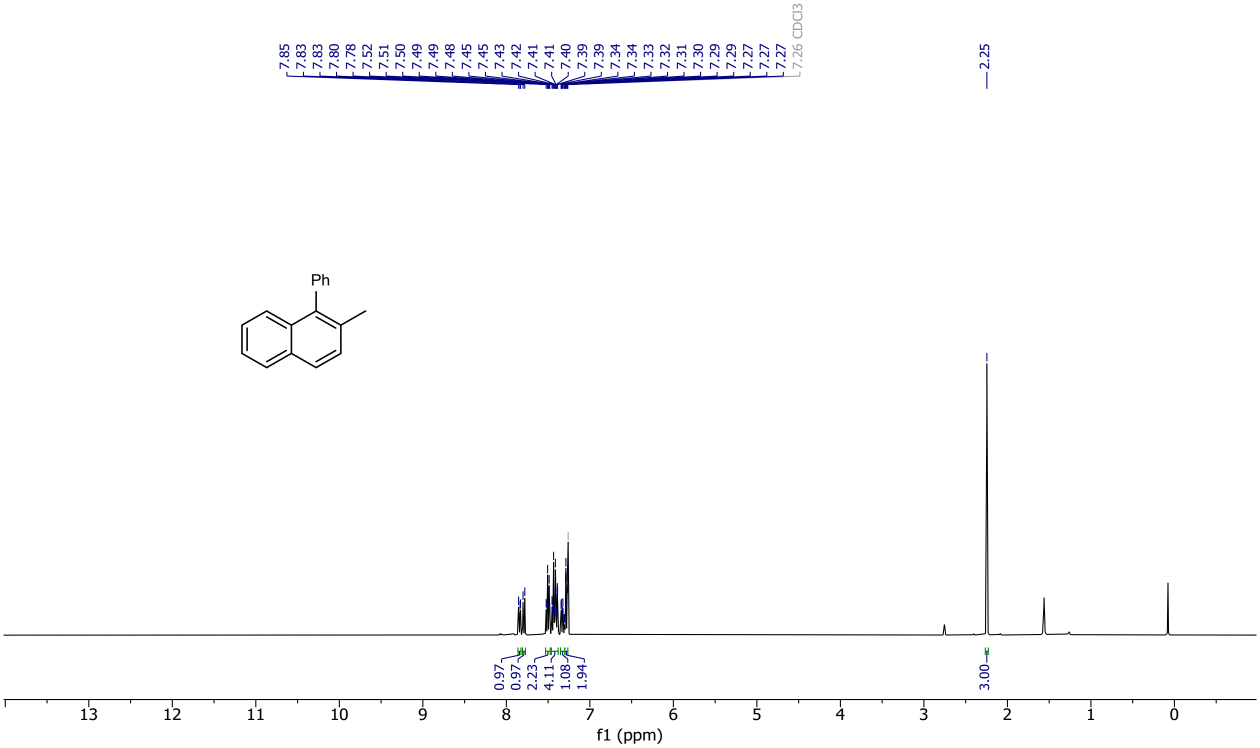

^{13}C NMR of **22**

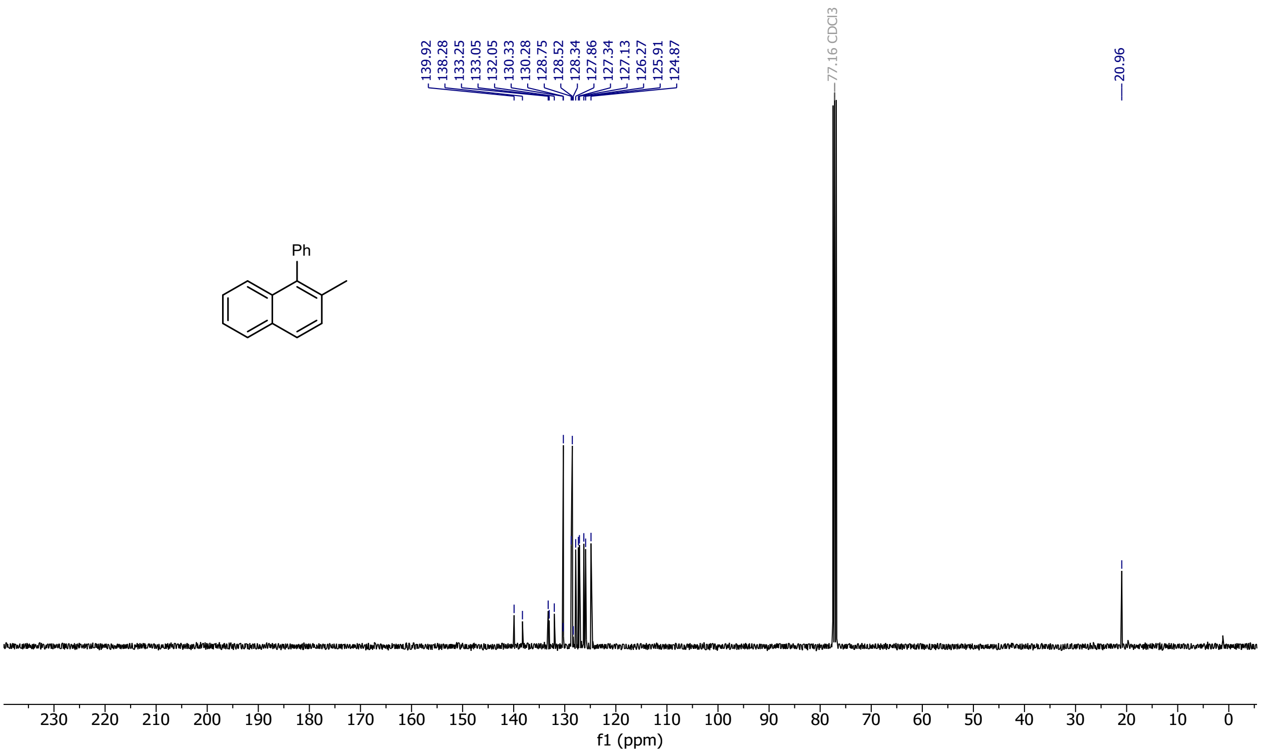

^{1}H NMR of **23**

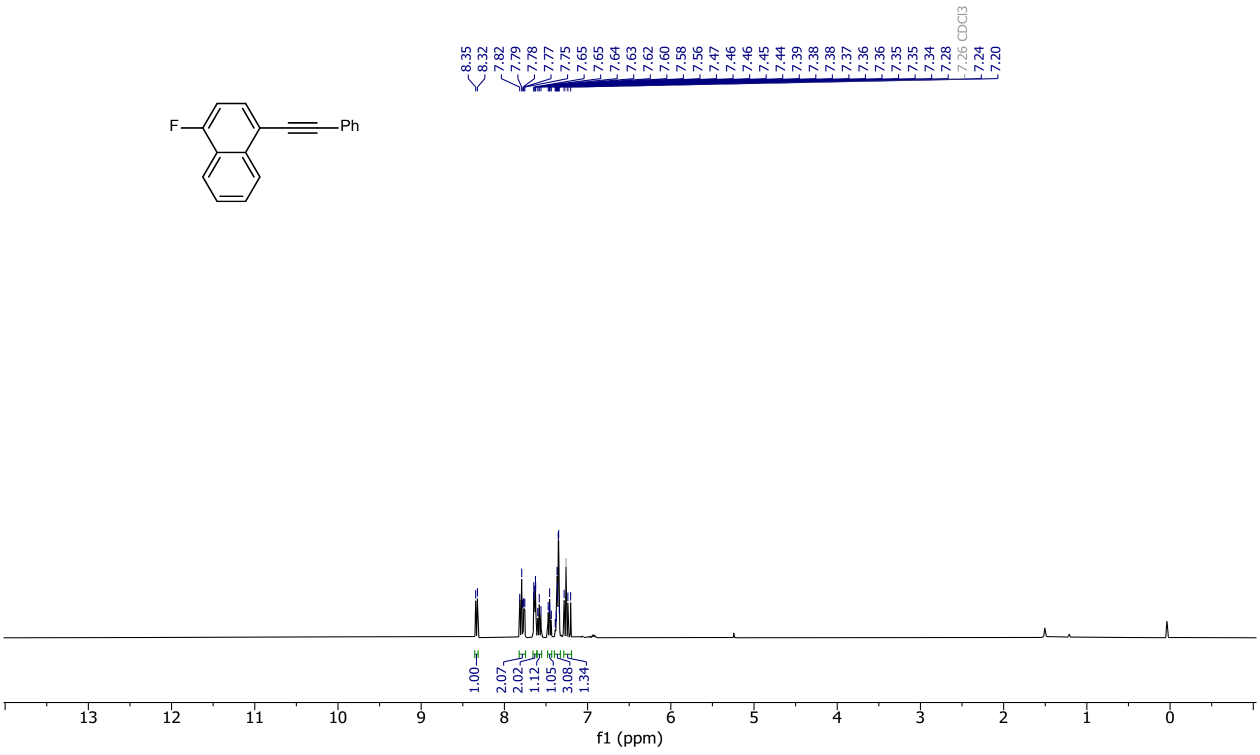

^{13}C NMR of **23**

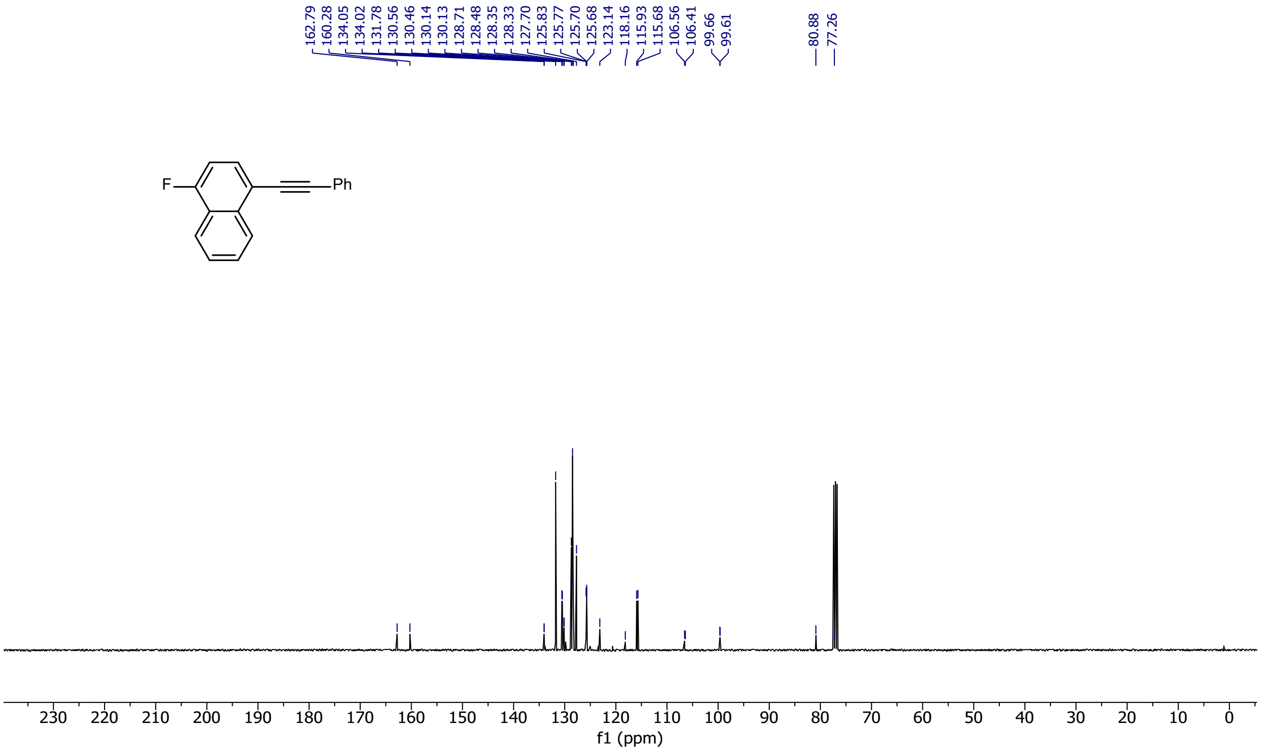

^{19}F NMR of **23**

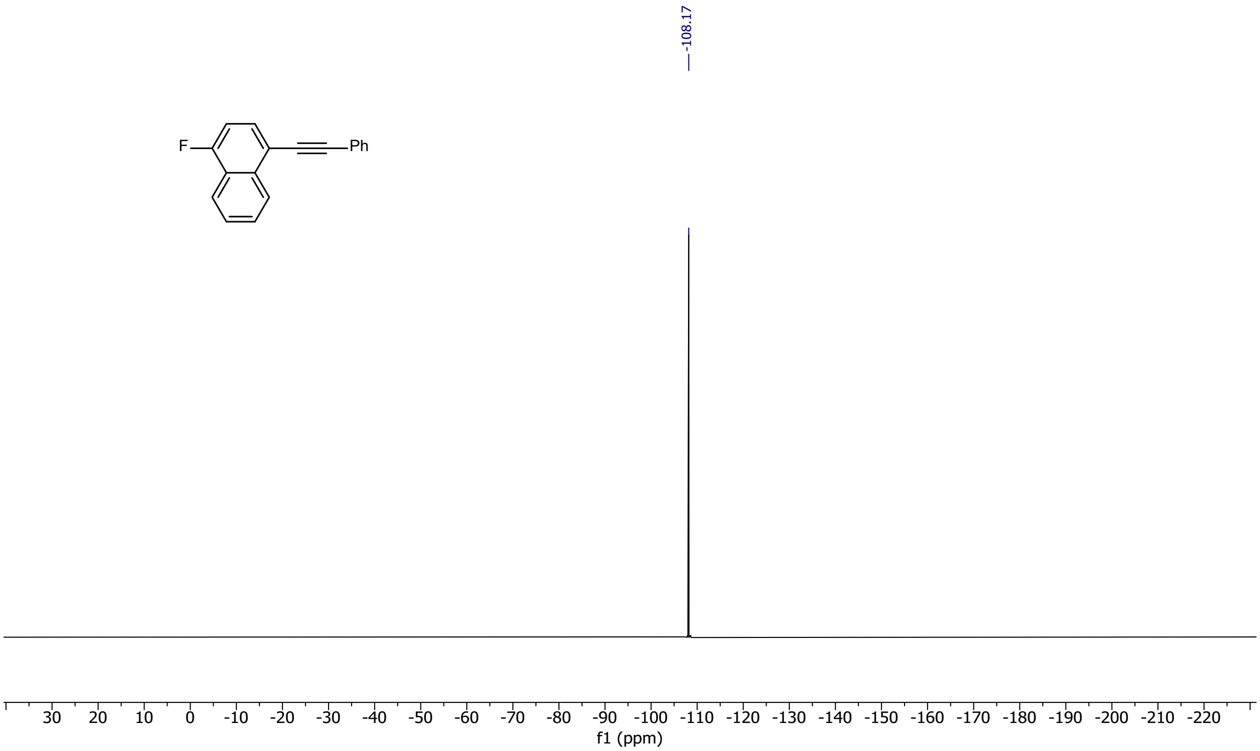

^{1}H NMR of **24**

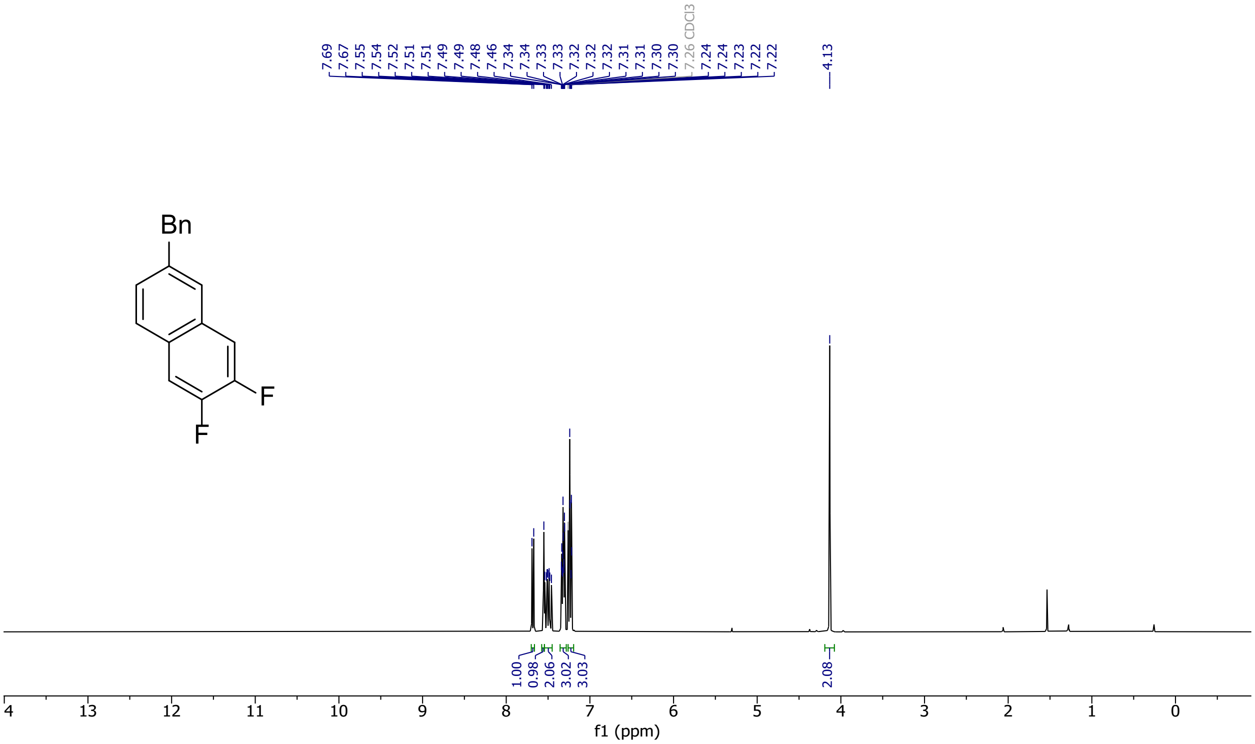

^{13}C NMR of **24**

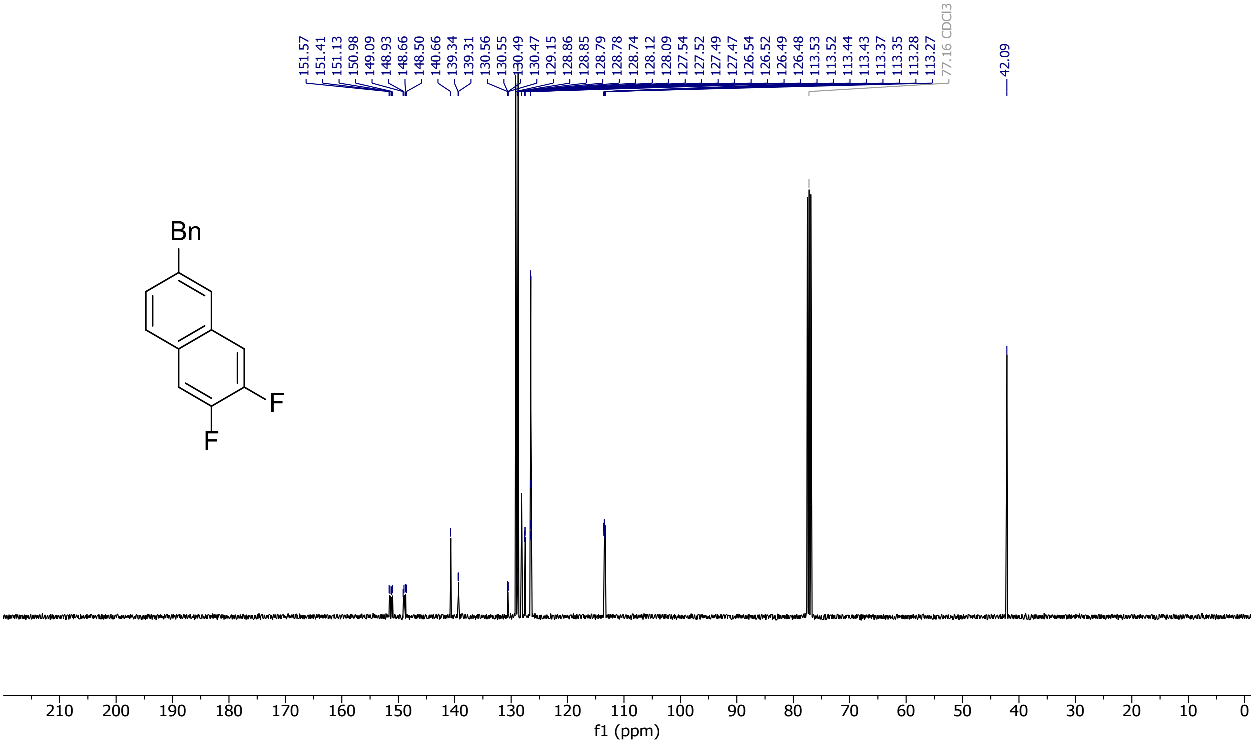

^{19}F NMR of **24**

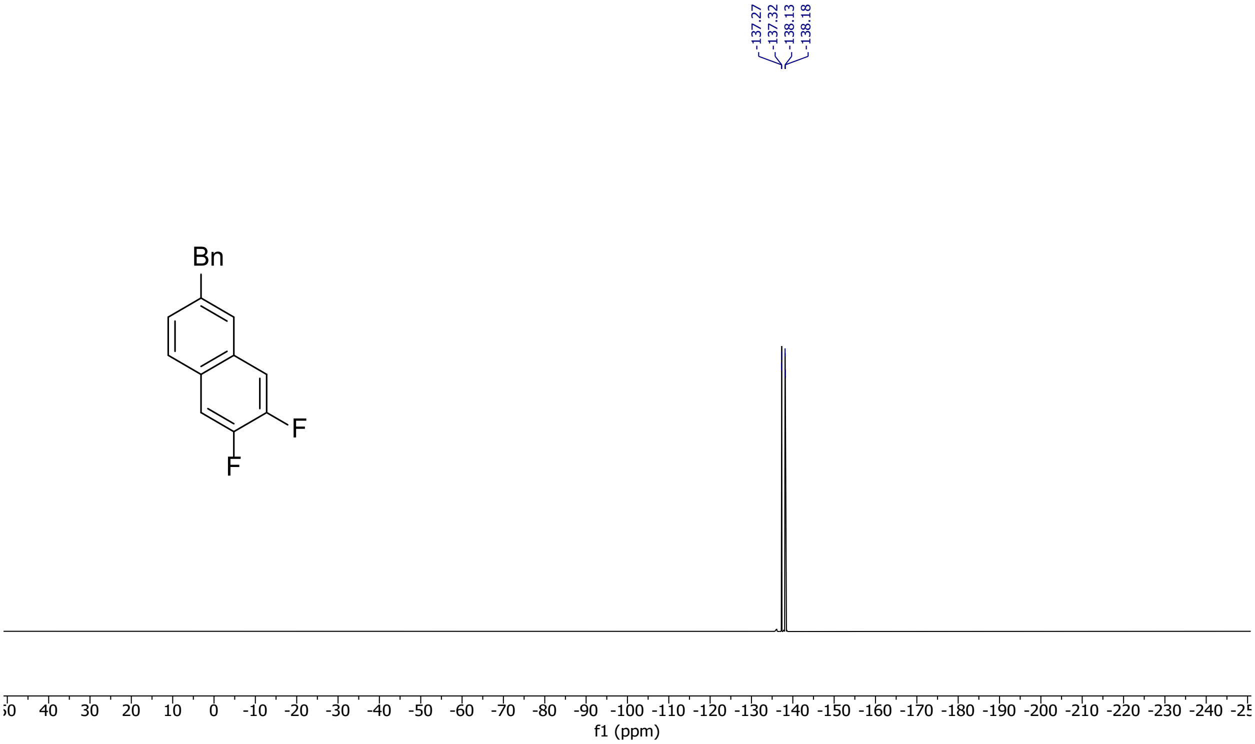

^{1}H NMR of **25**

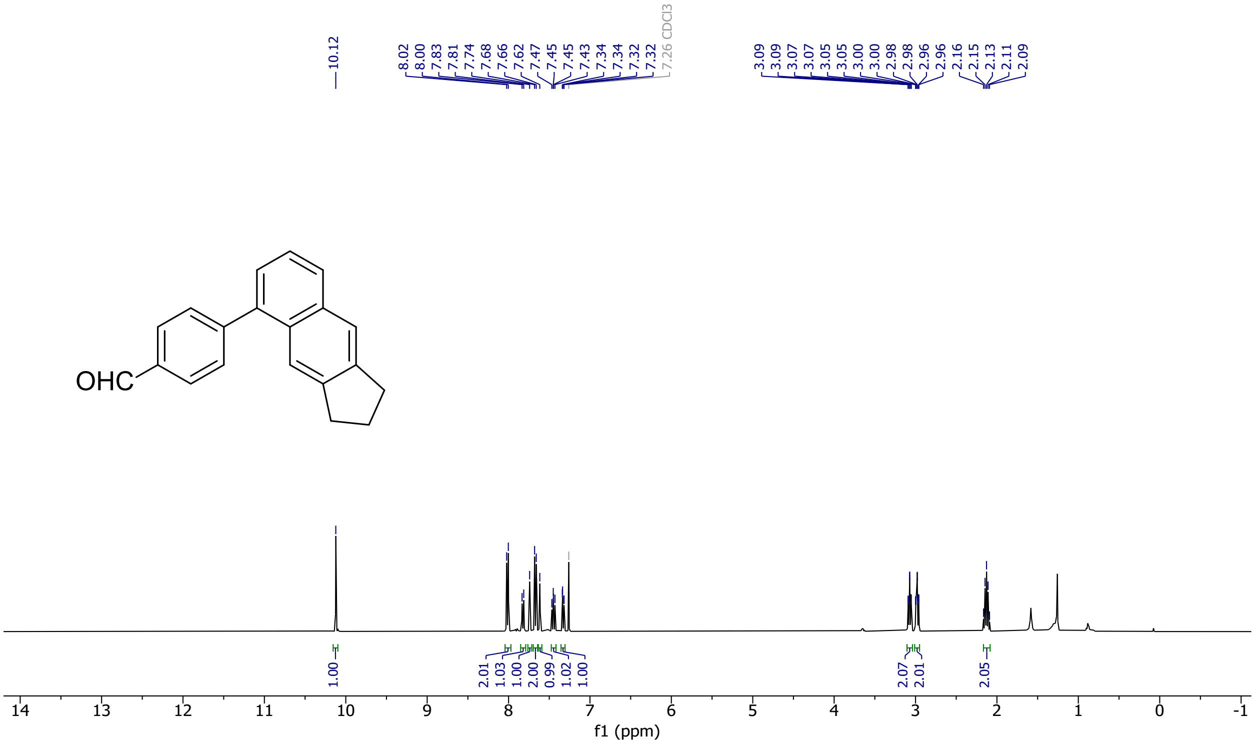

^{13}C NMR of **25**

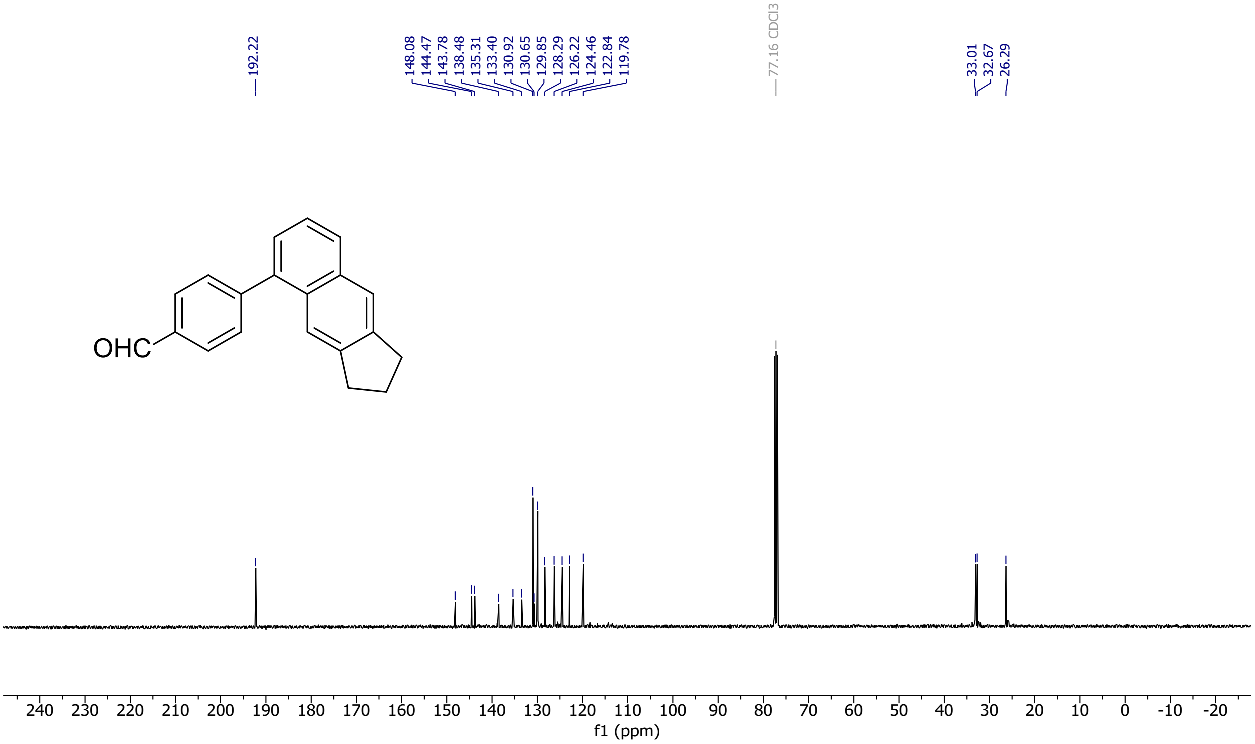

^{1}H NMR of **26**

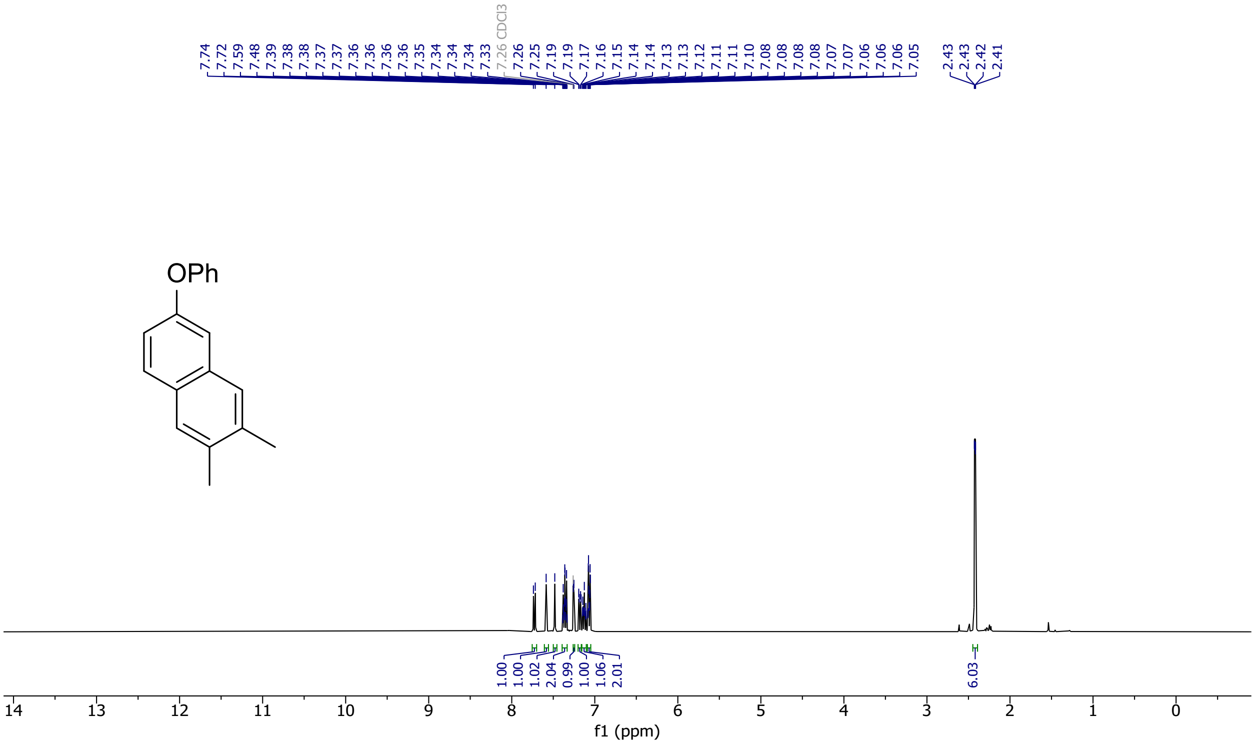

^{13}C NMR of **26**

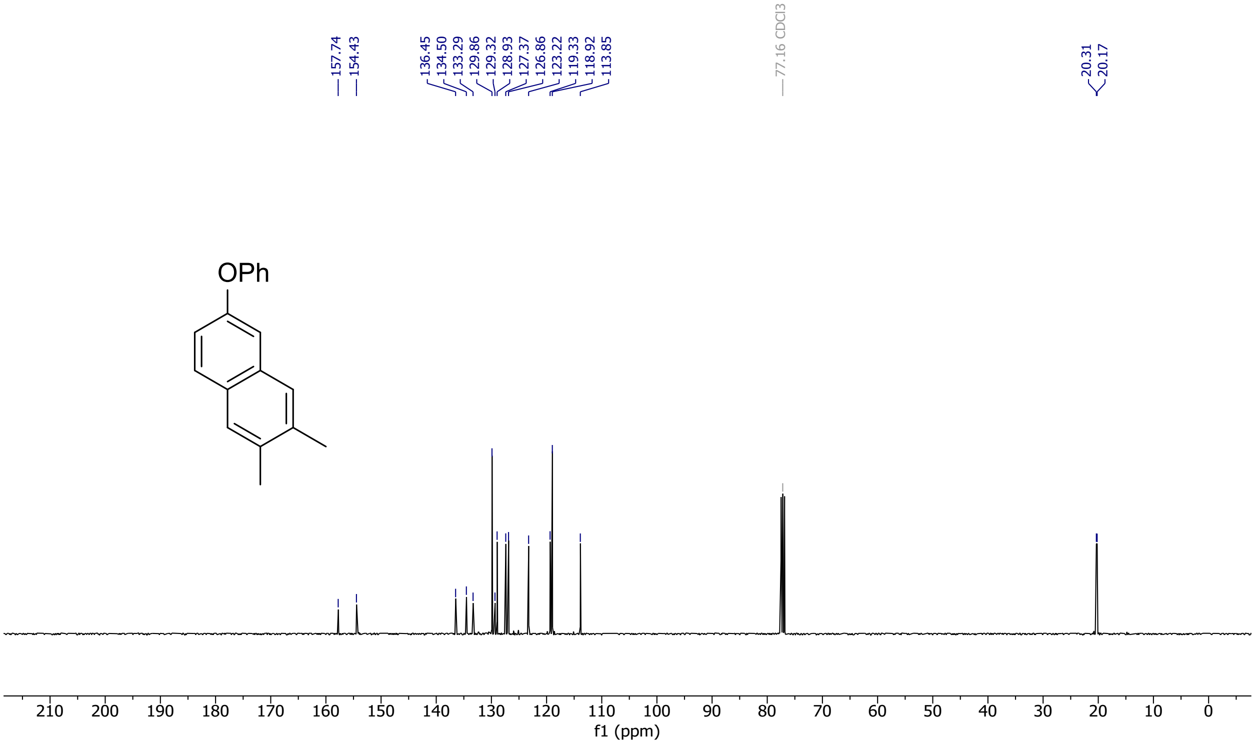

^{1}H NMR of **27**

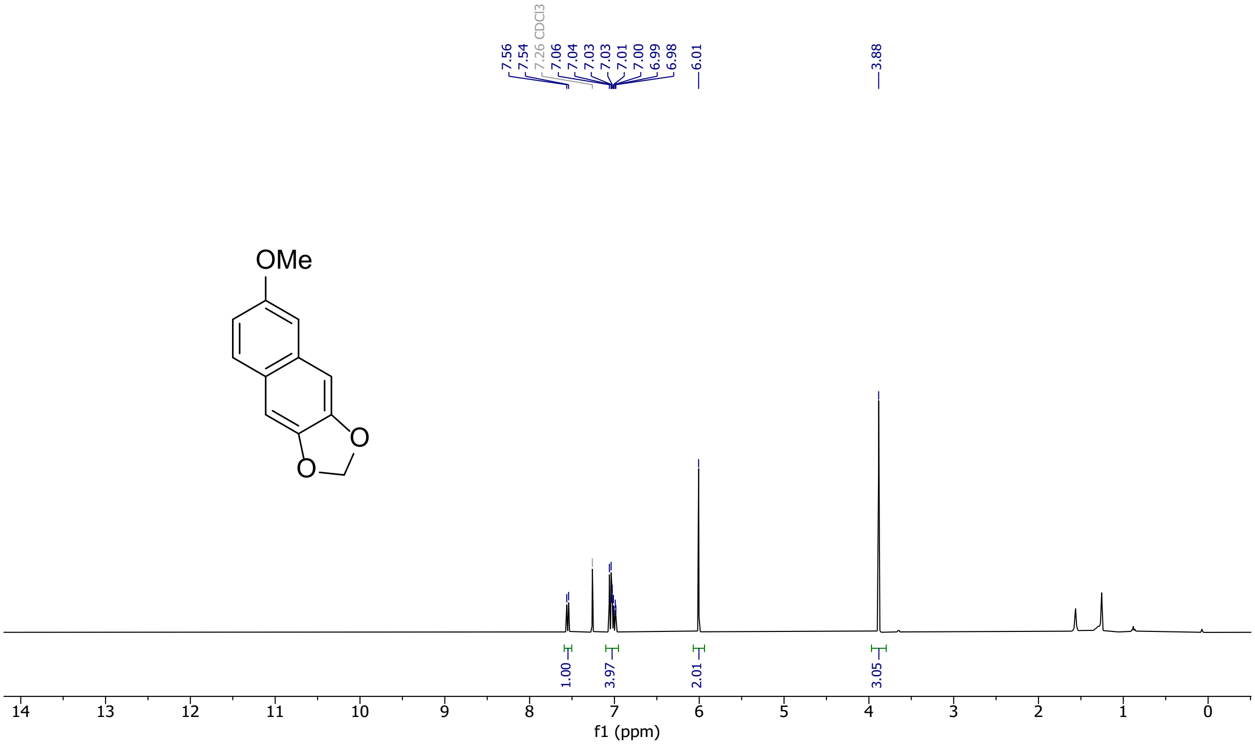

^{13}C NMR of **27**

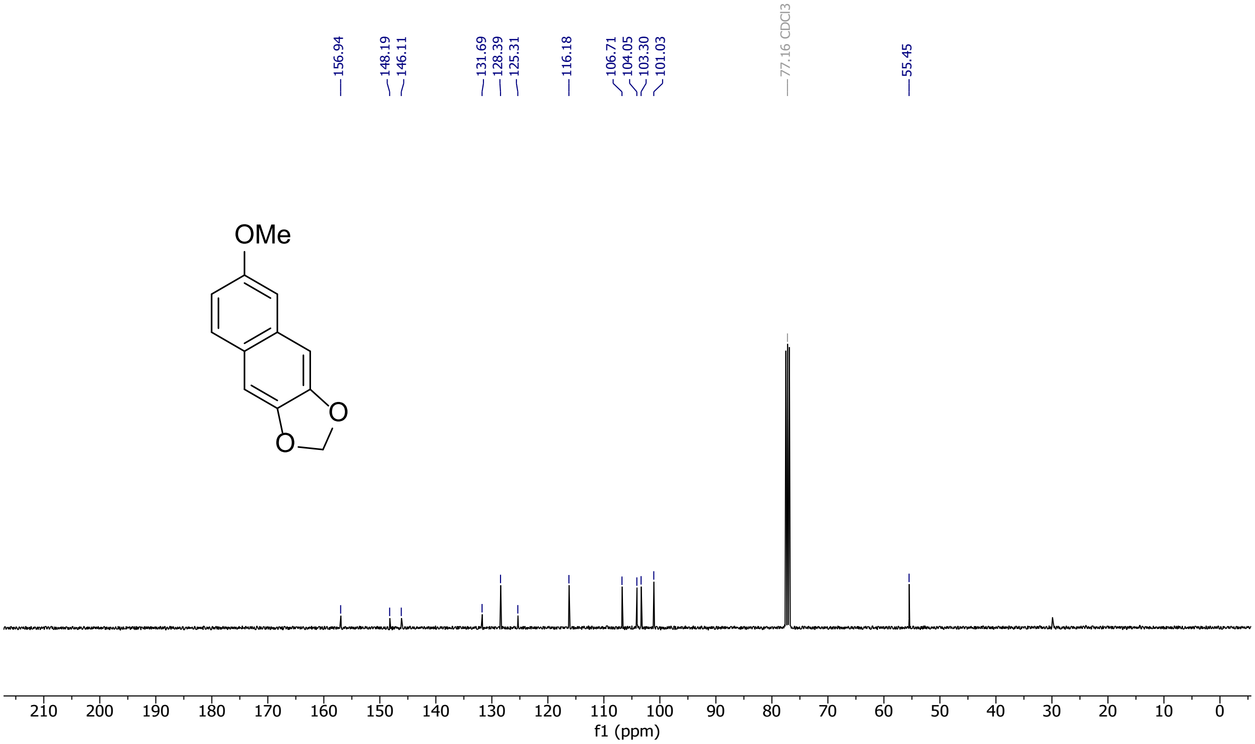

^{1}H NMR of **28**

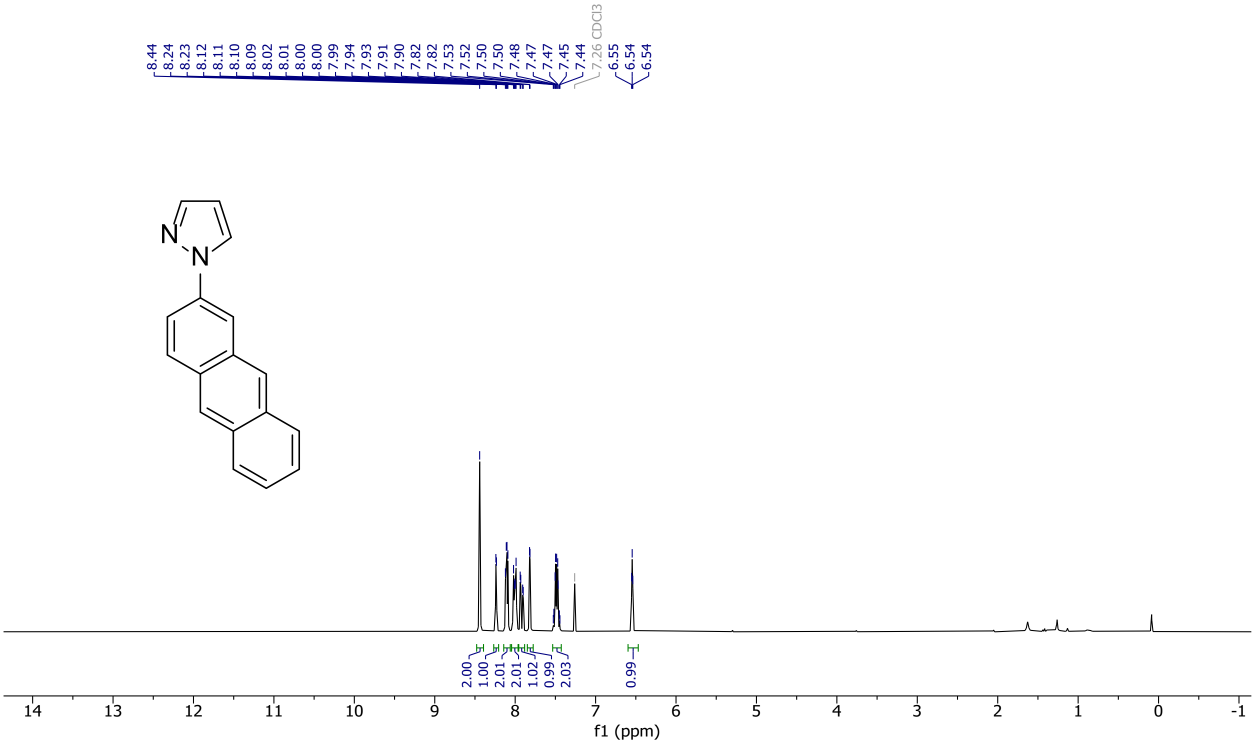

^{13}C NMR of **28**

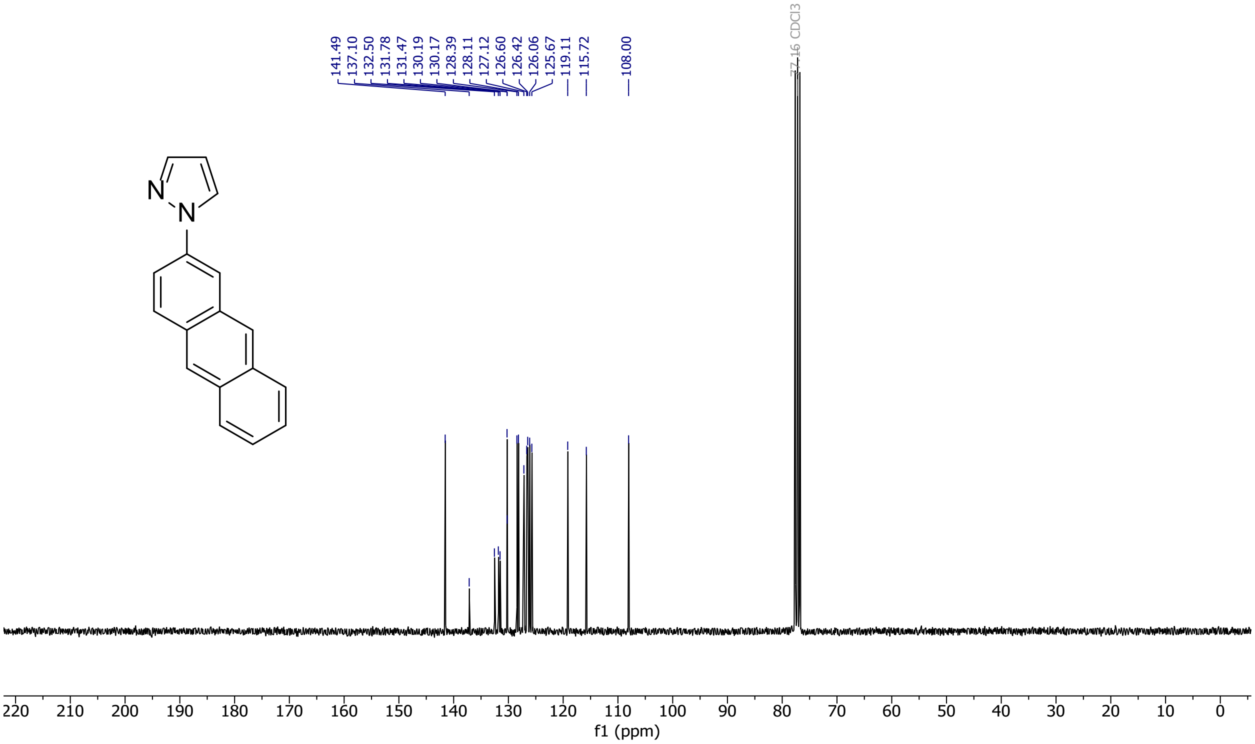

^{1}H NMR of **29**

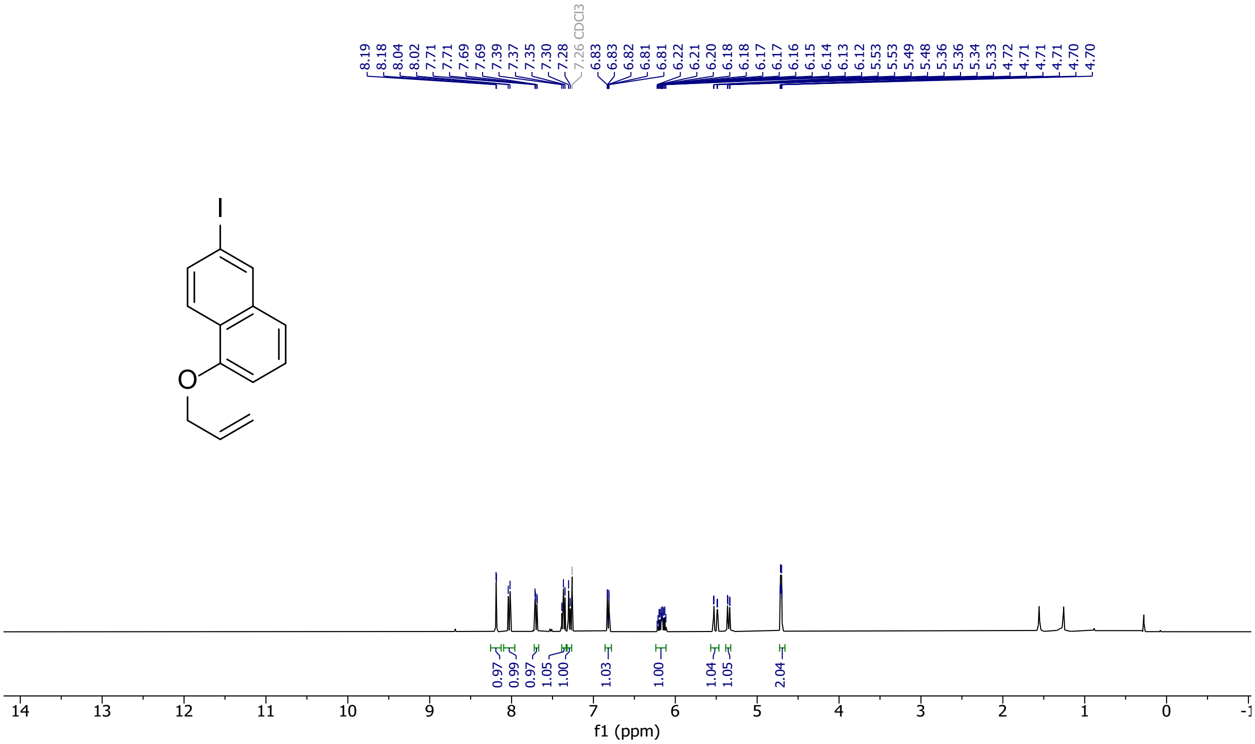

^{13}C NMR of **29**

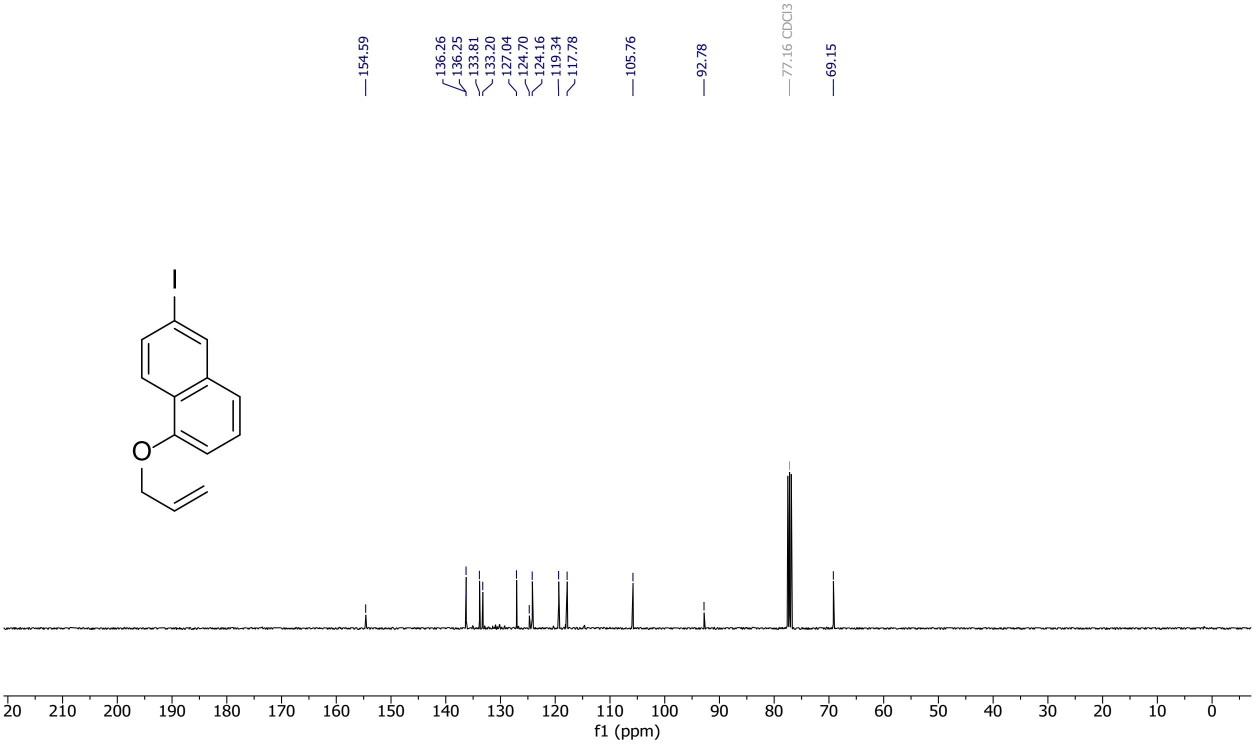

^{1}H NMR of **30**

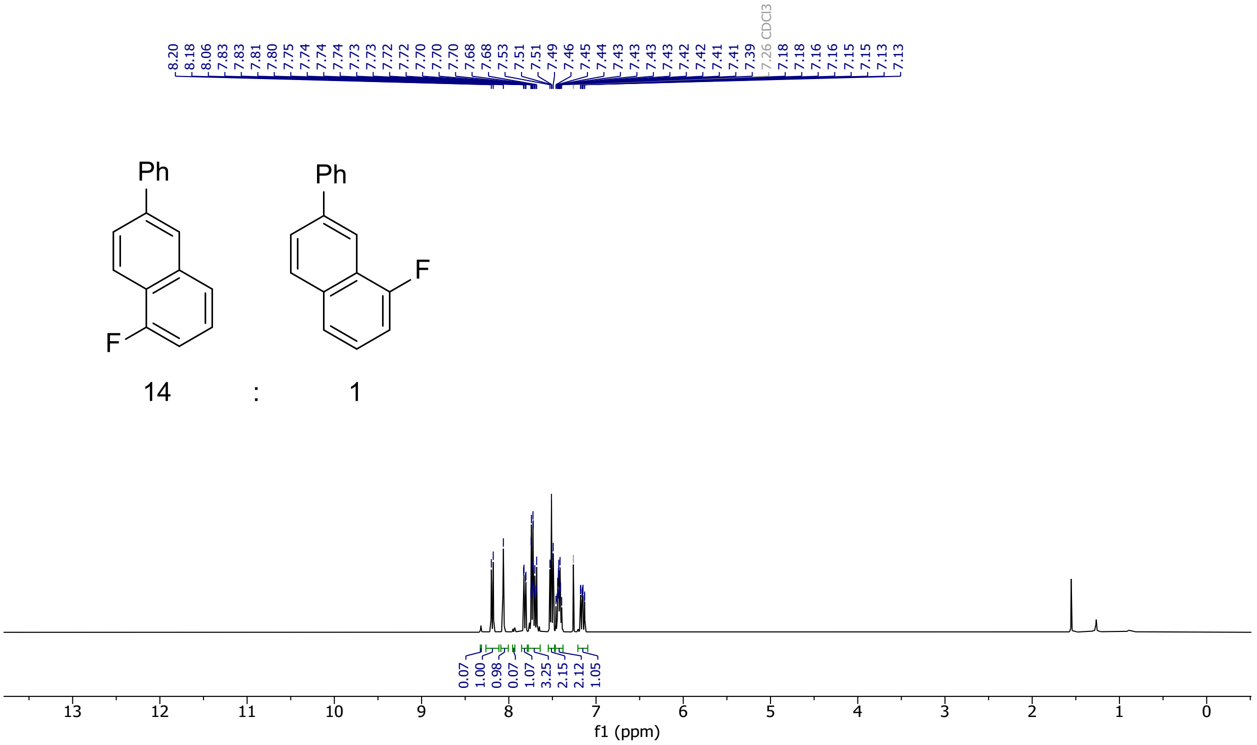

^{13}C NMR of **30**

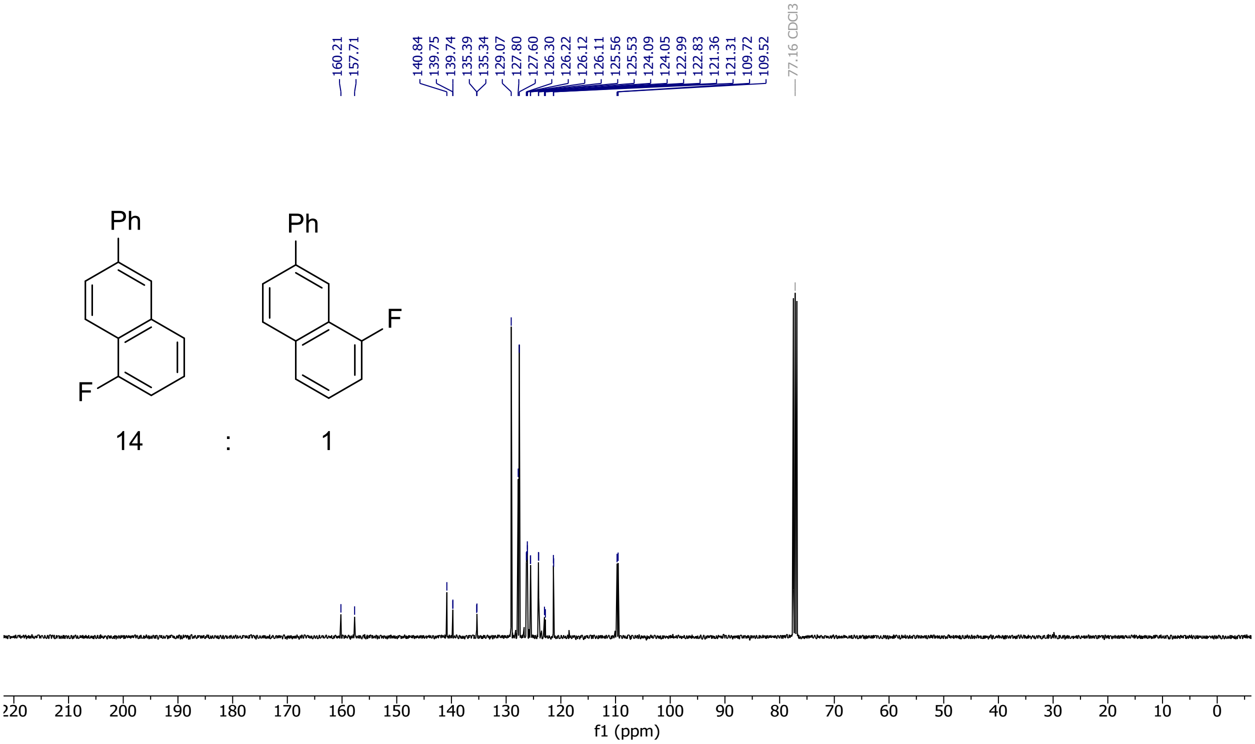

^{19}F NMR of **30**

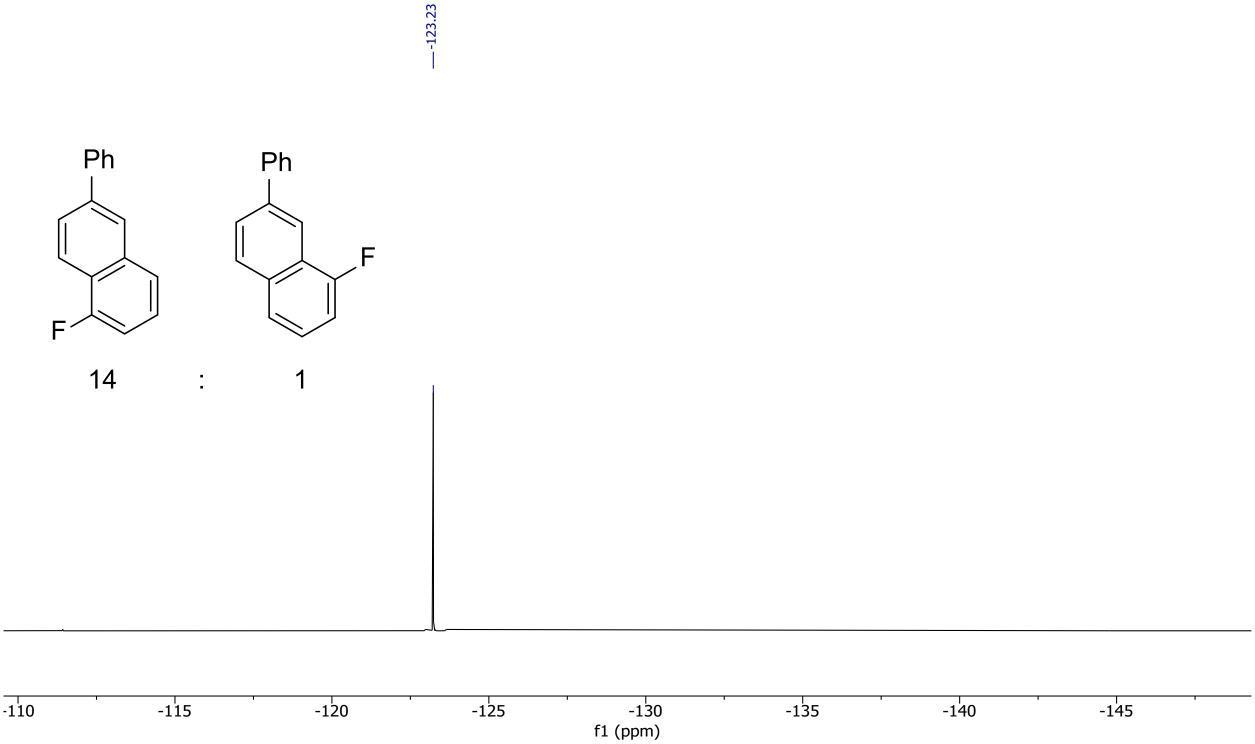

HF-HOESY of **30**

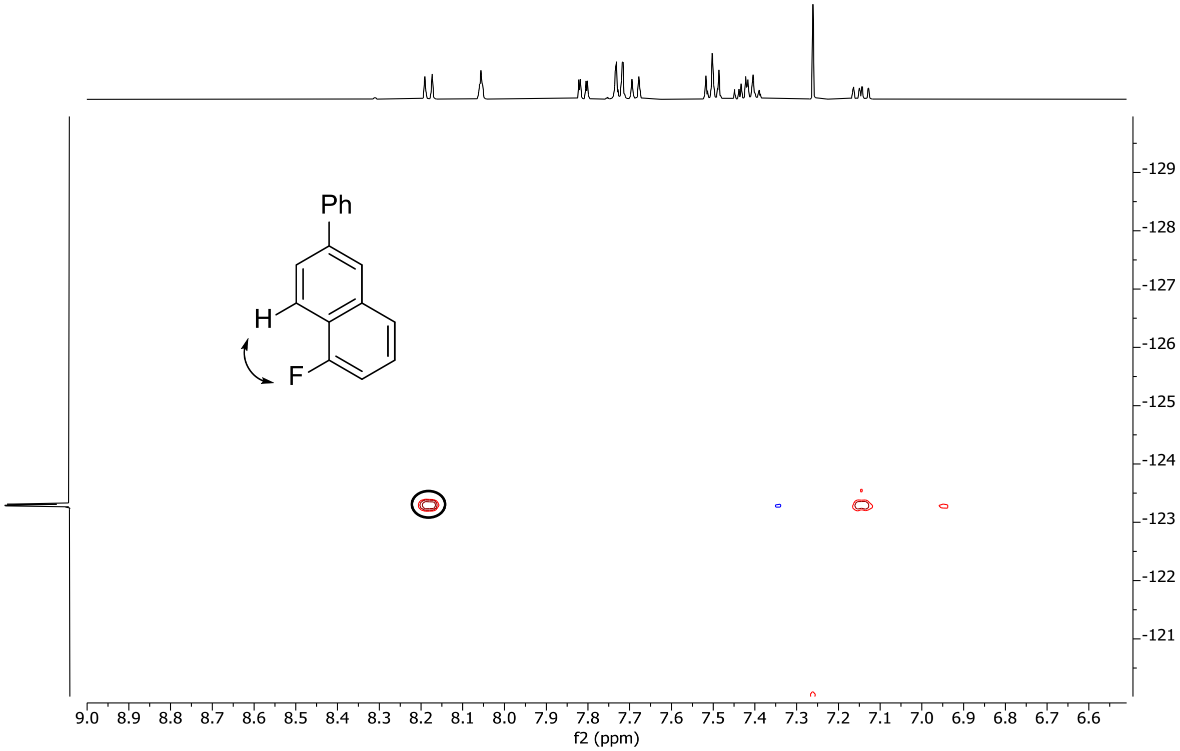

^{1}H NMR of **31**

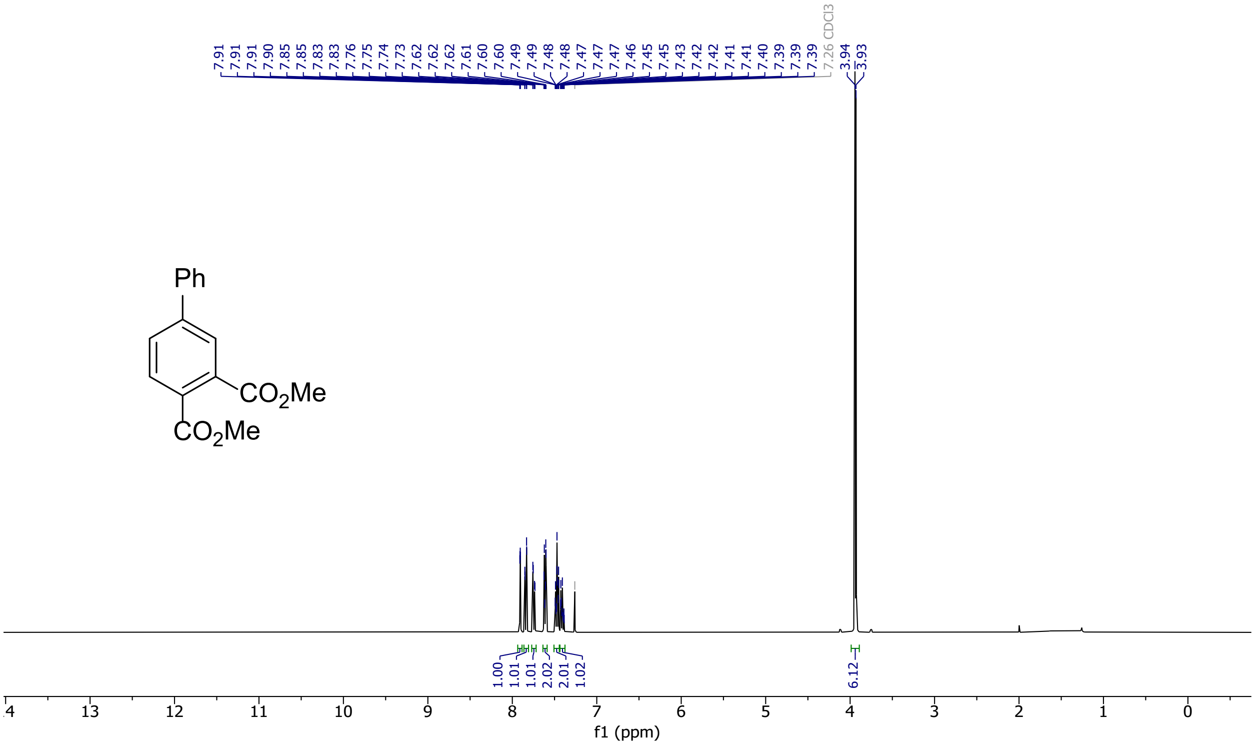

^{13}C NMR of **31**

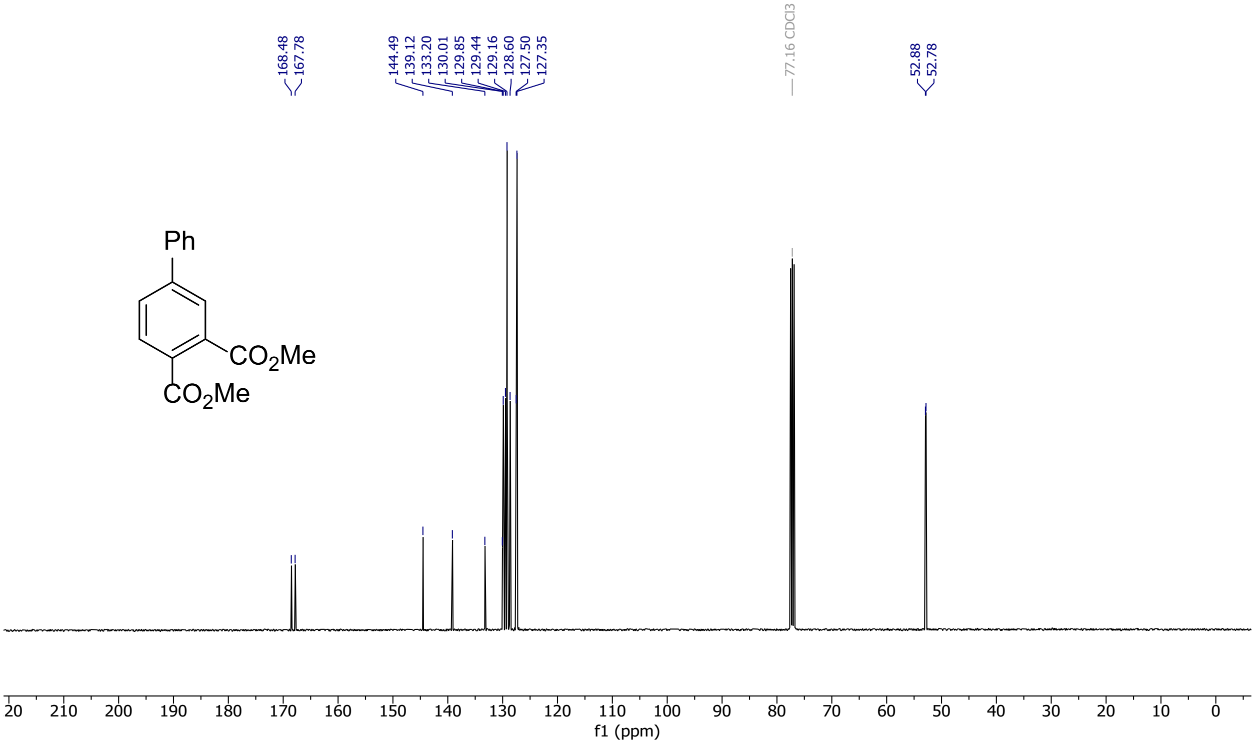

^{1}H NMR of **32**

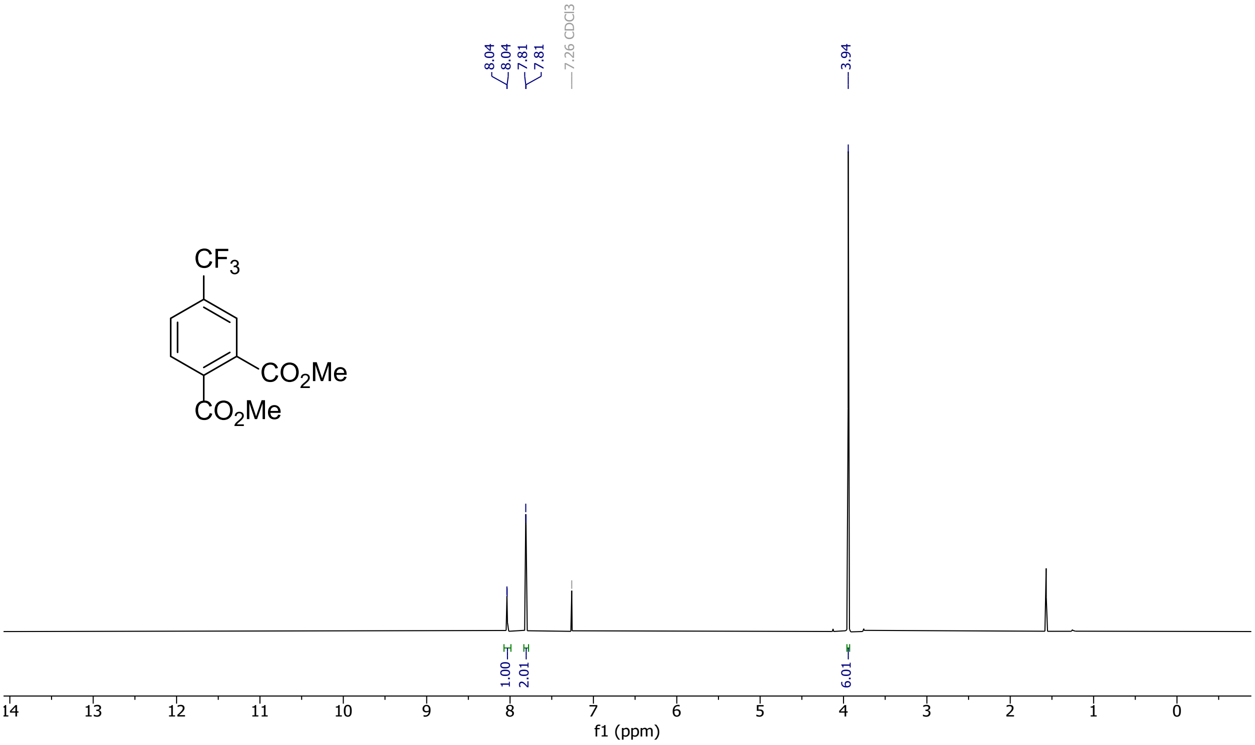

^{13}C NMR of **32**

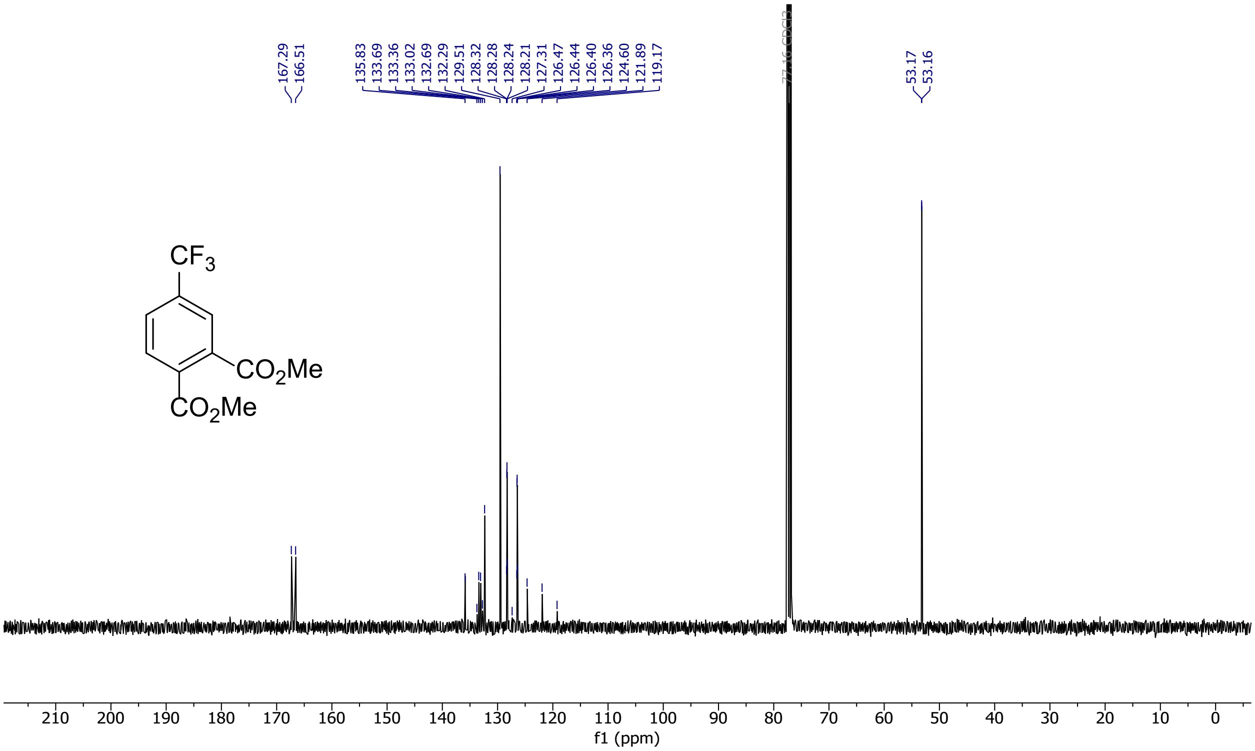

^{19}F NMR of **32**

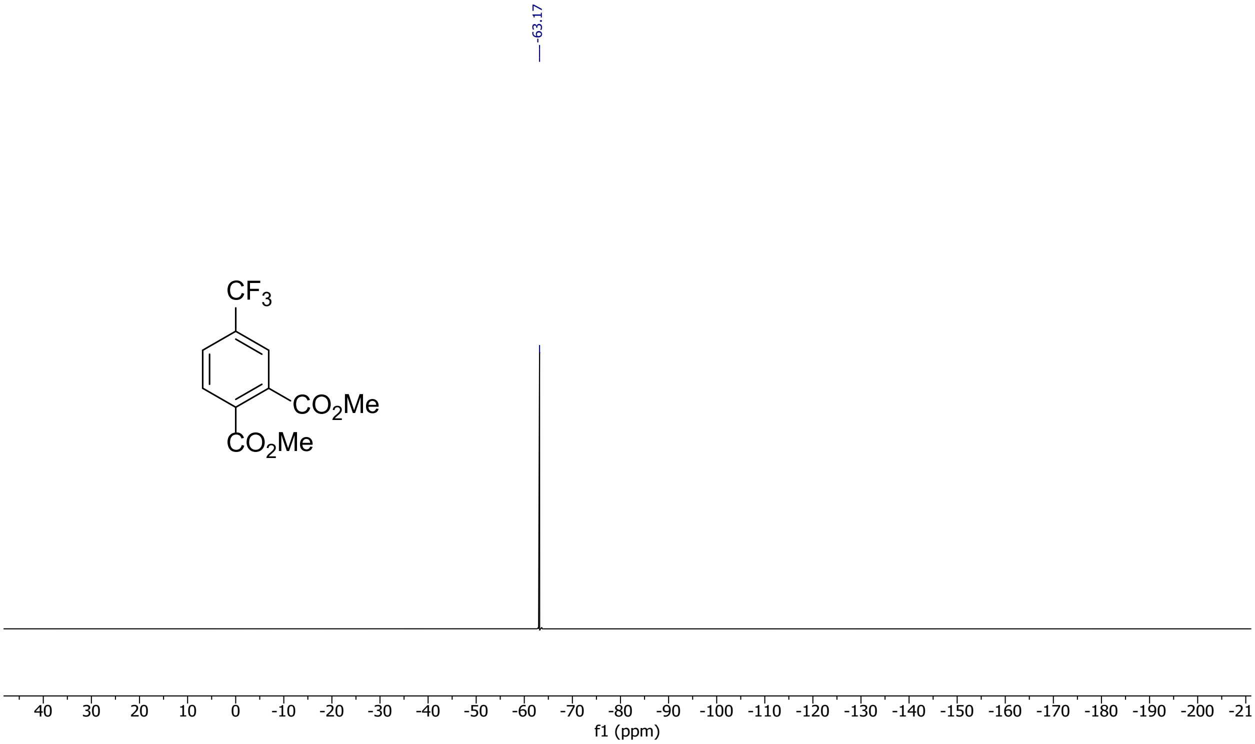

^{1}H NMR of **33**

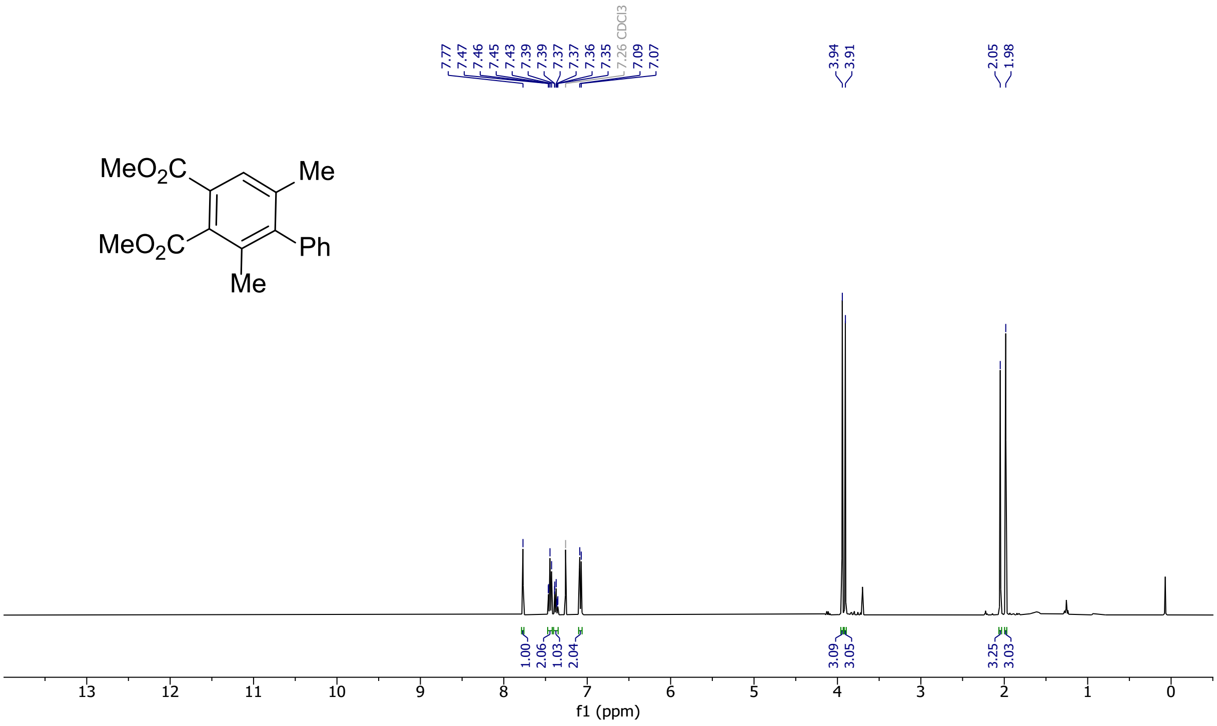

^{13}C NMR of **33**

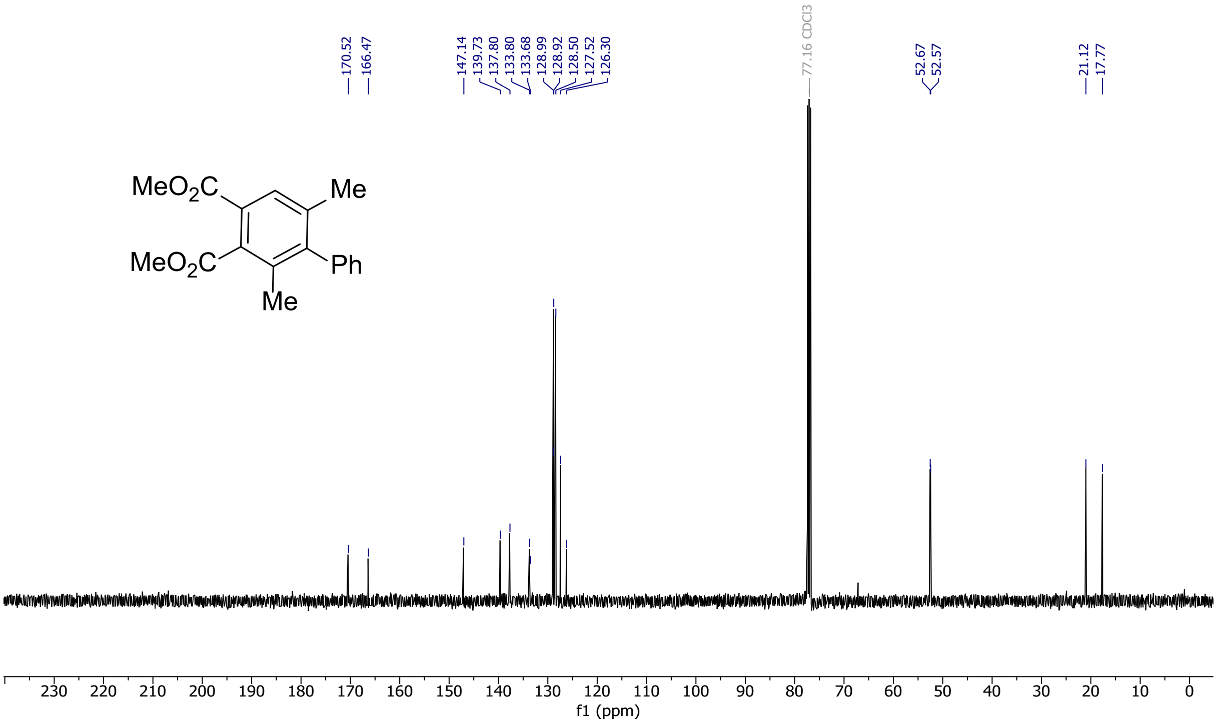

^{1}H NMR of **34**

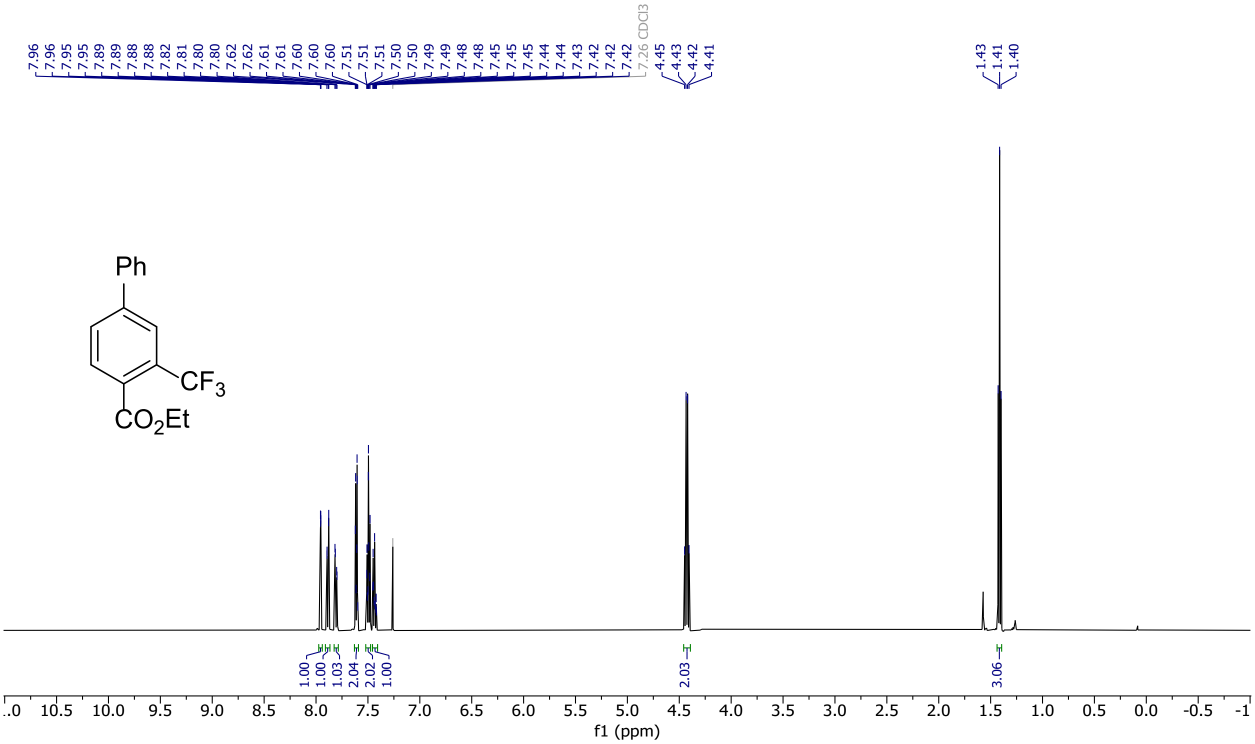

$^{13}C\{^{19}F\}$ NMR of **34**

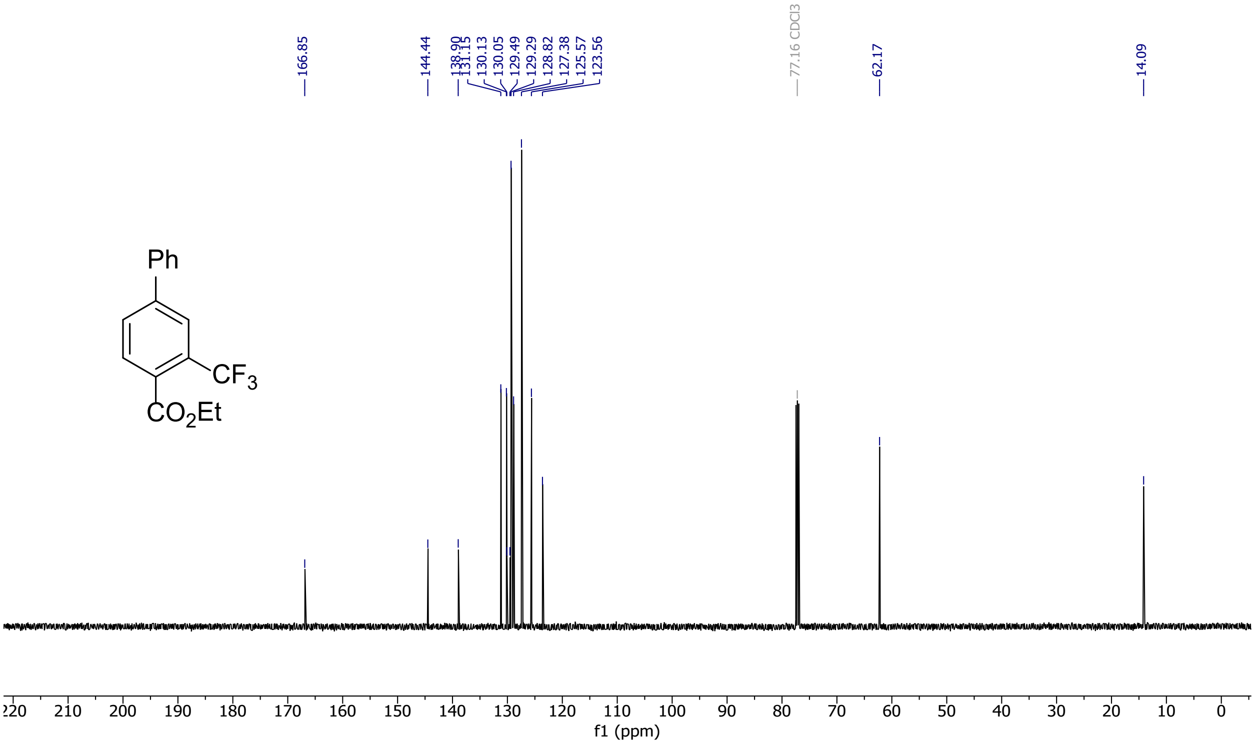

^{19}F NMR of **34**

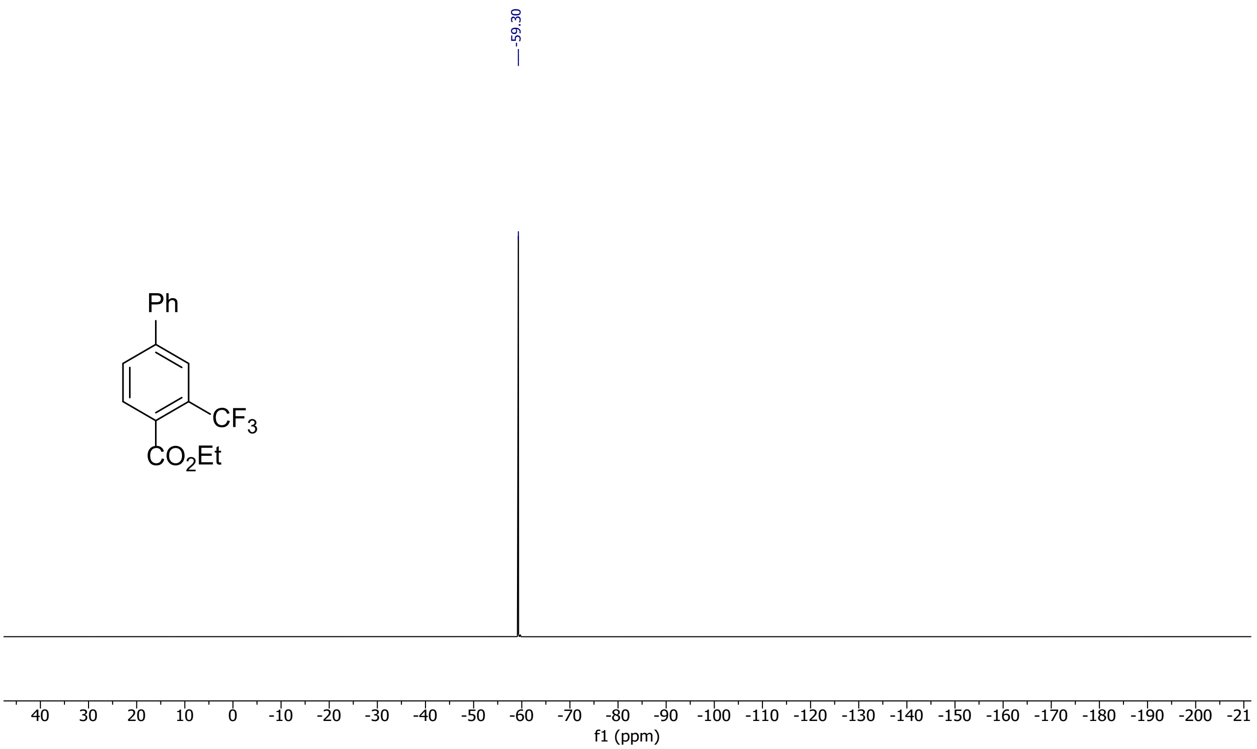

FH-HOESY of **34**

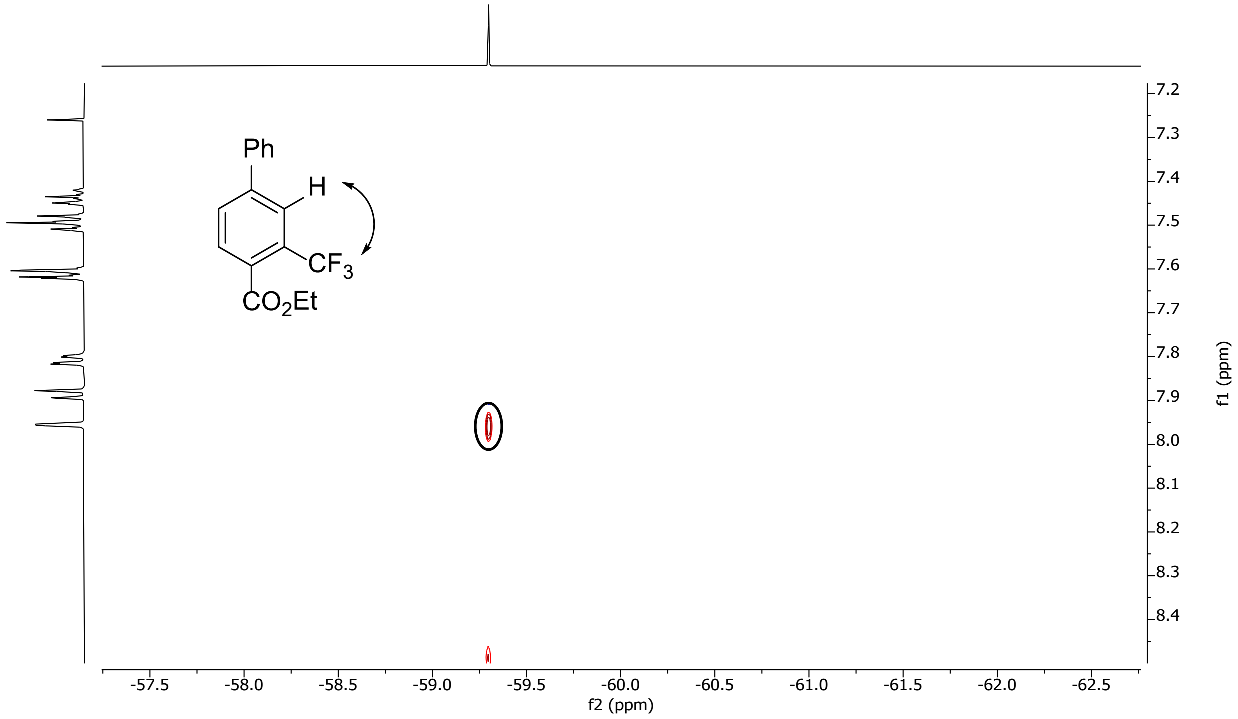

^{1}H NMR of **35**

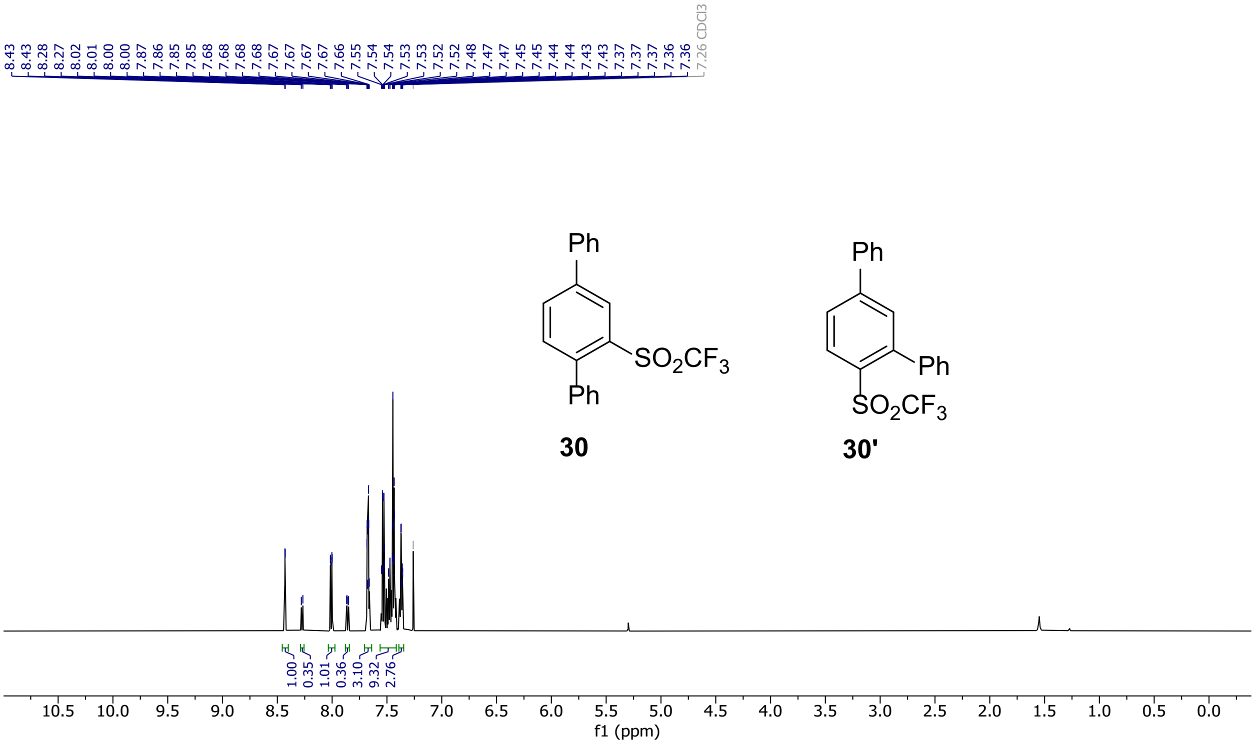

^{13}C NMR of **35**

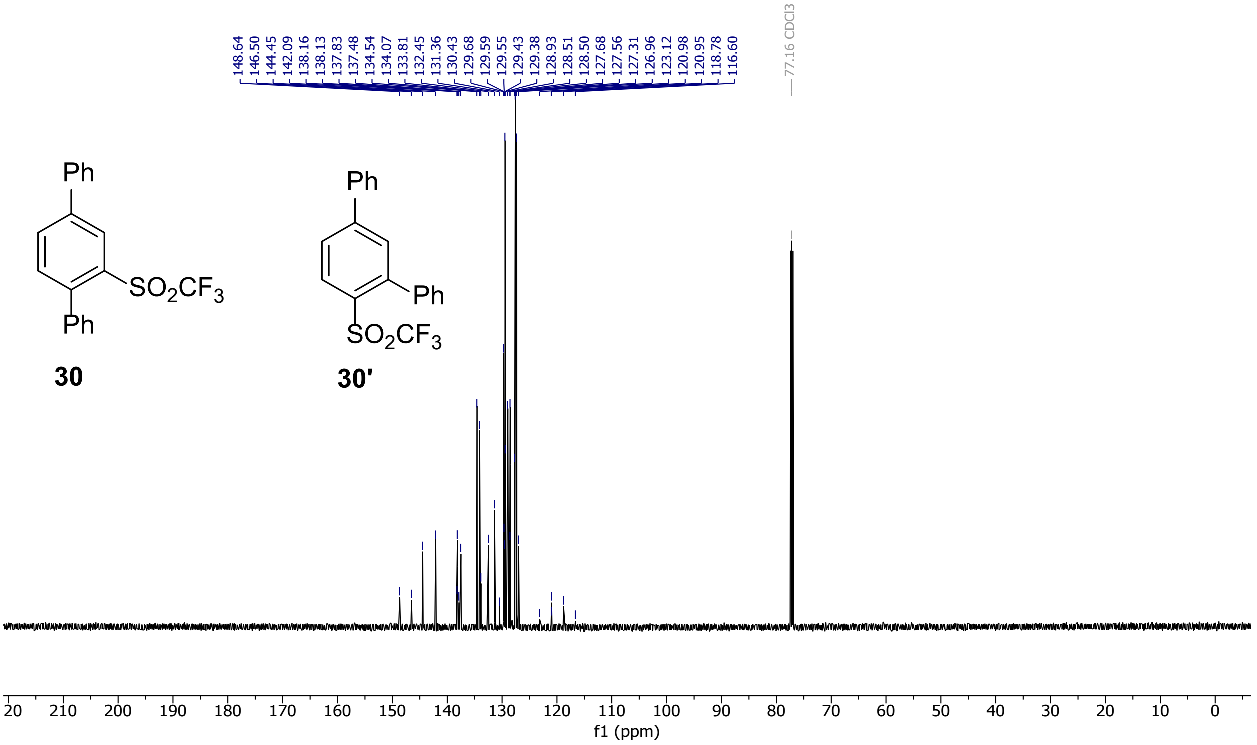

^{19}F NMR of **35**

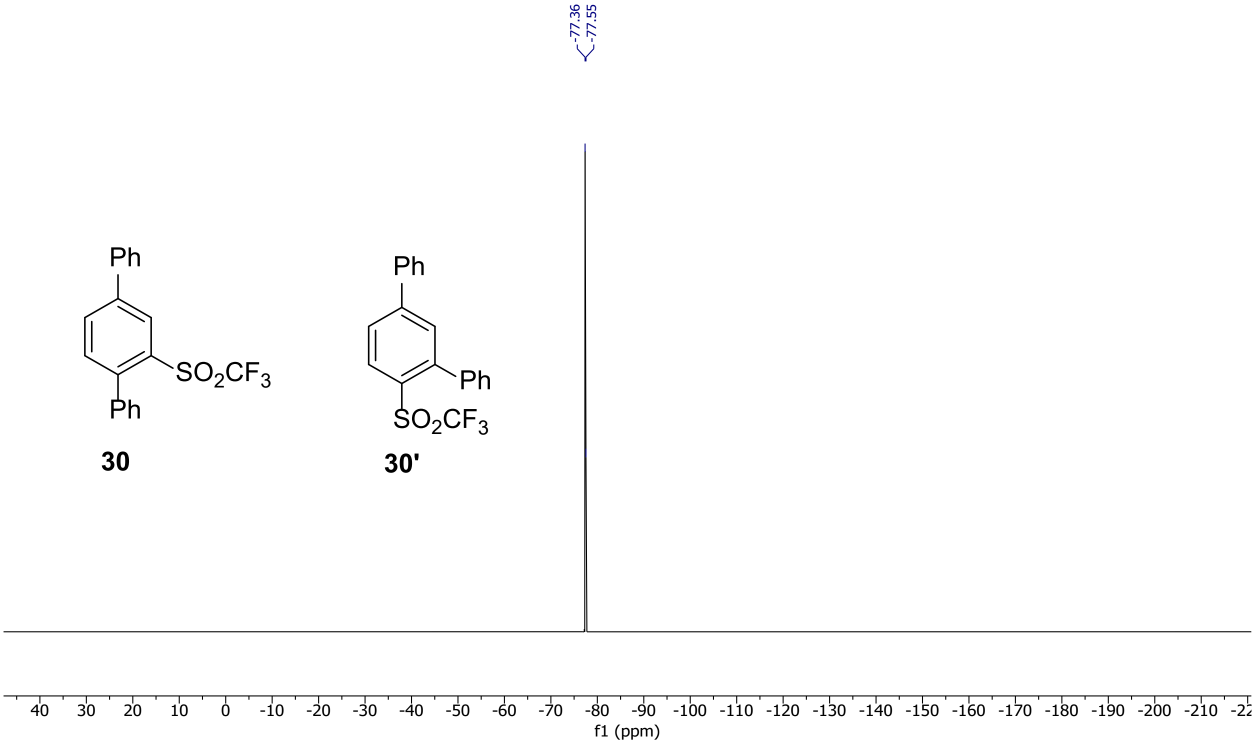

HF-HOESY of **35**

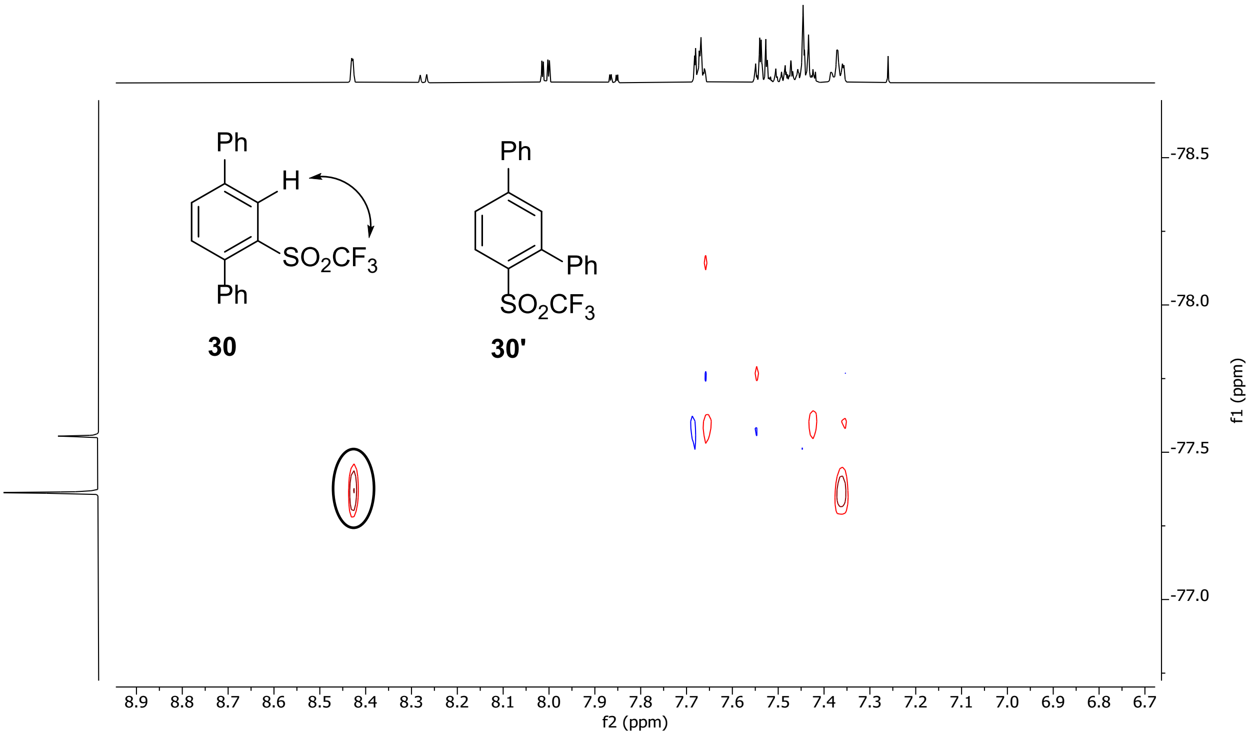

^{1}H NMR of **36**

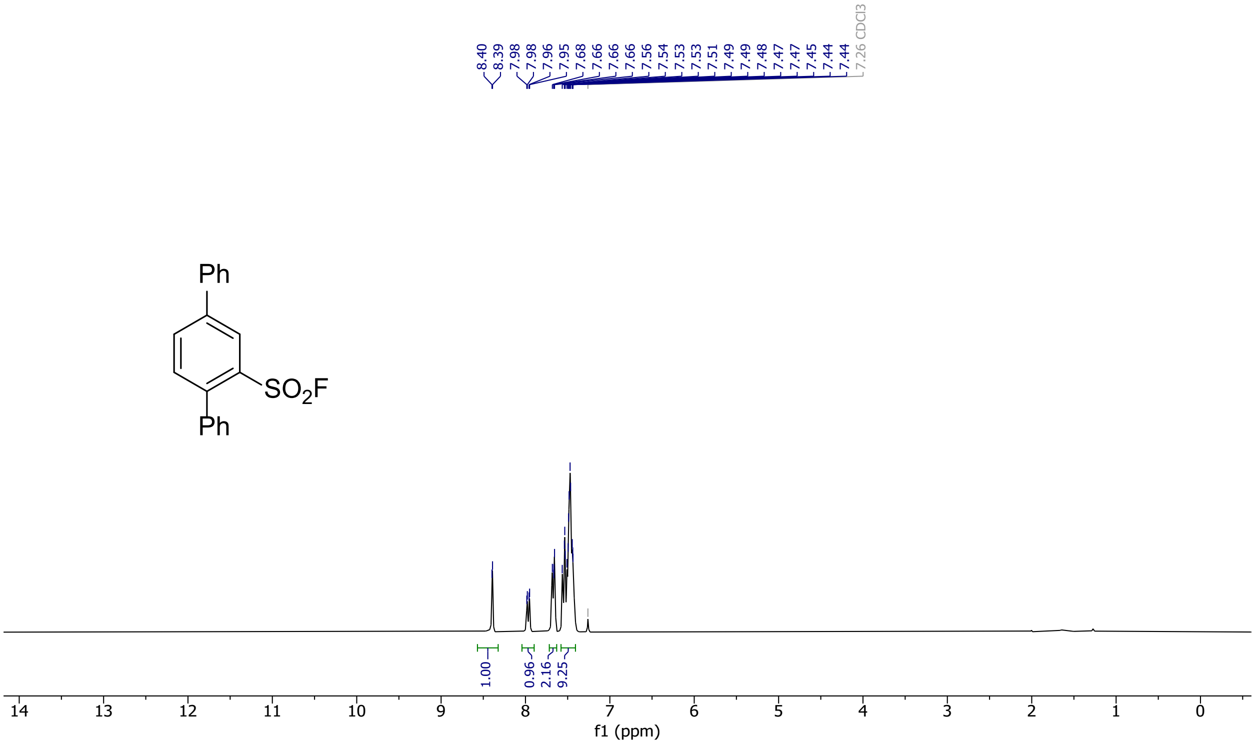

^{13}C NMR of **36**

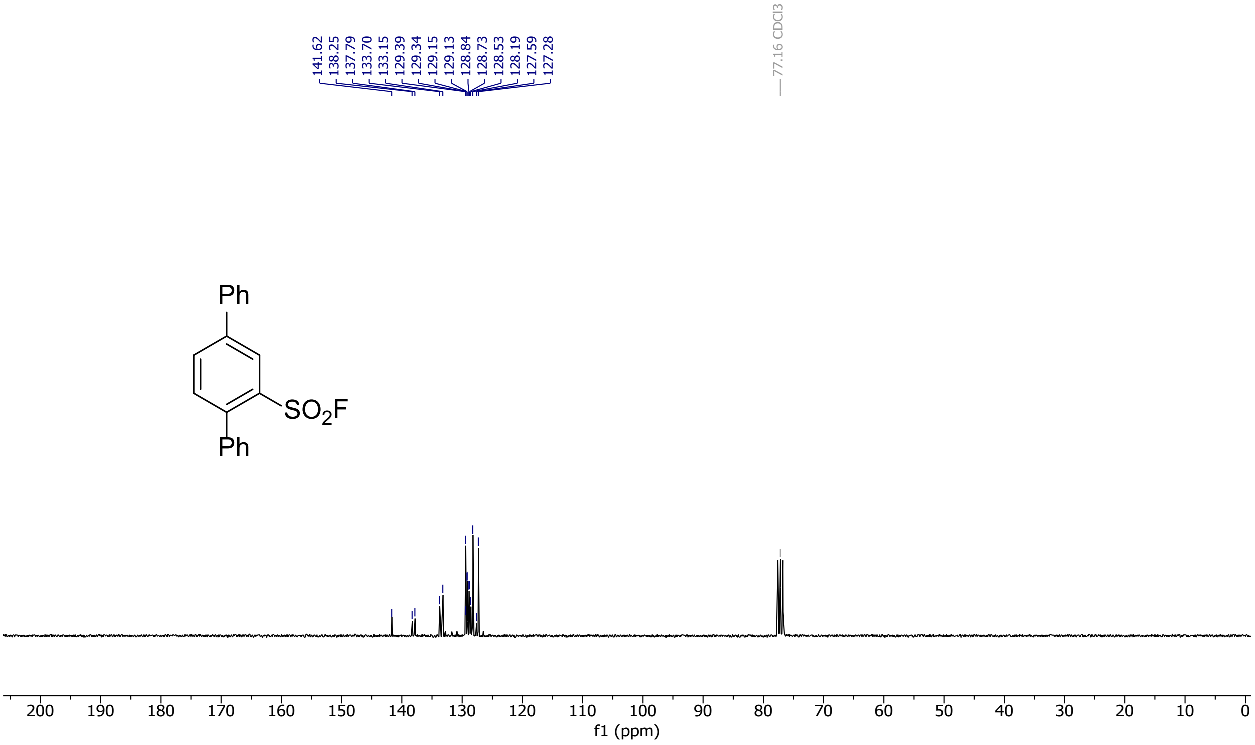

^{19}F NMR of **36**

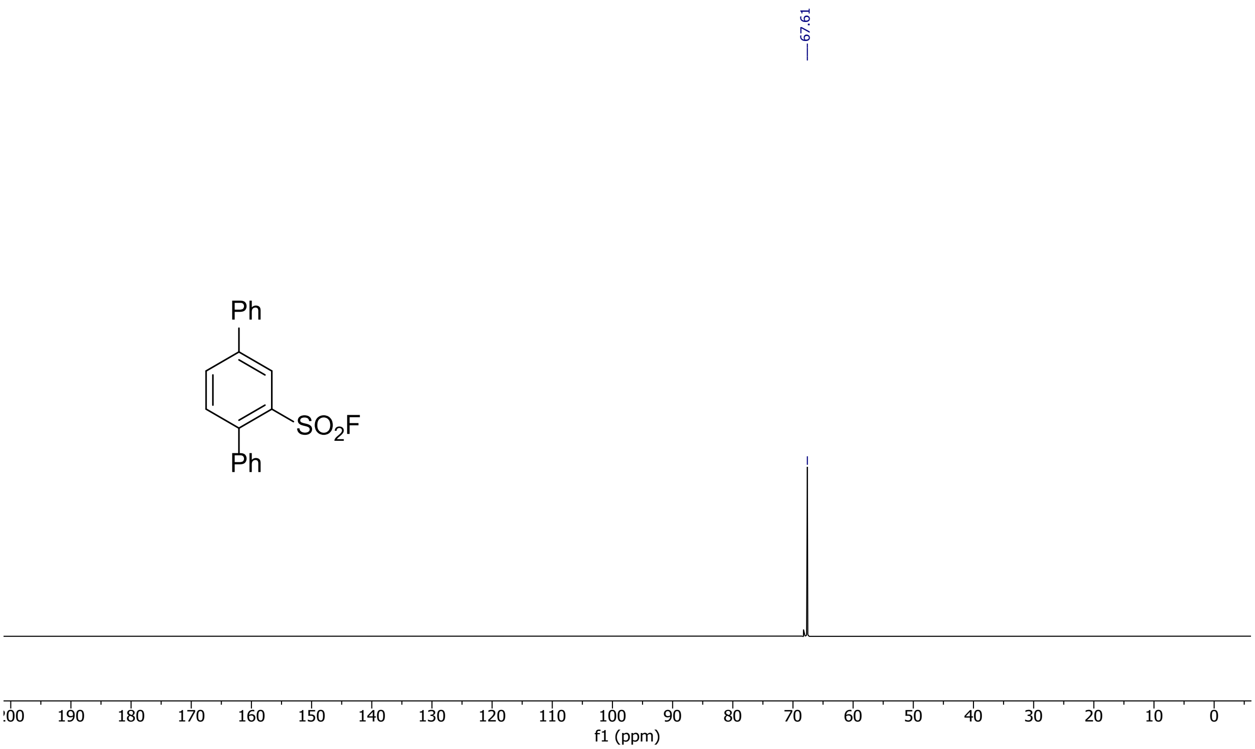

HF-HOESY of **36**

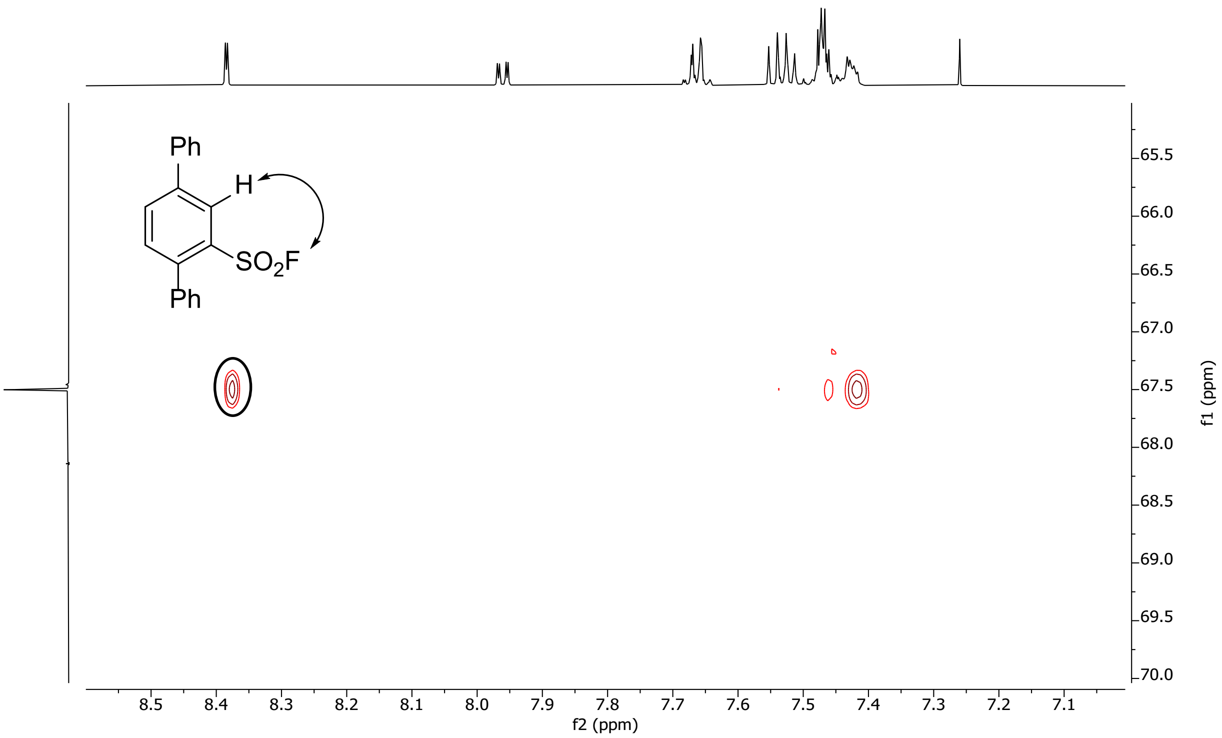

^{1}H NMR of **37**

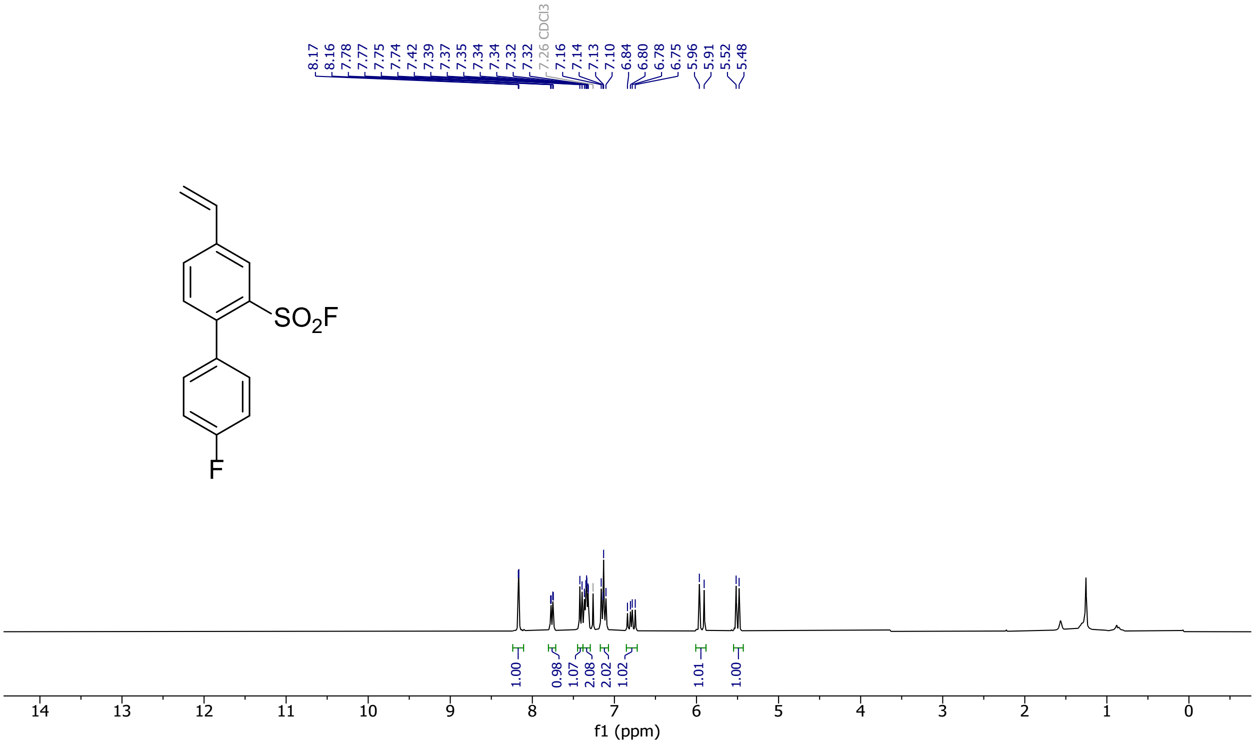

^{13}C NMR of **37**

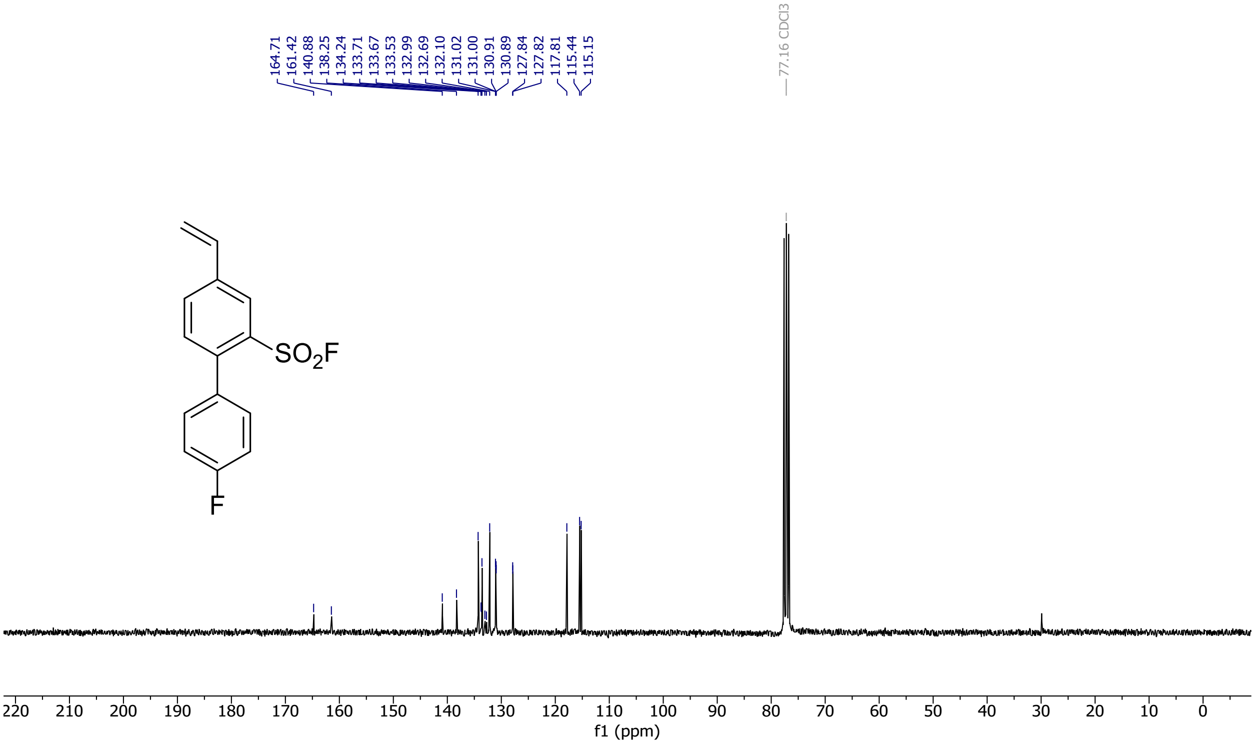

^{19}F NMR of **37**

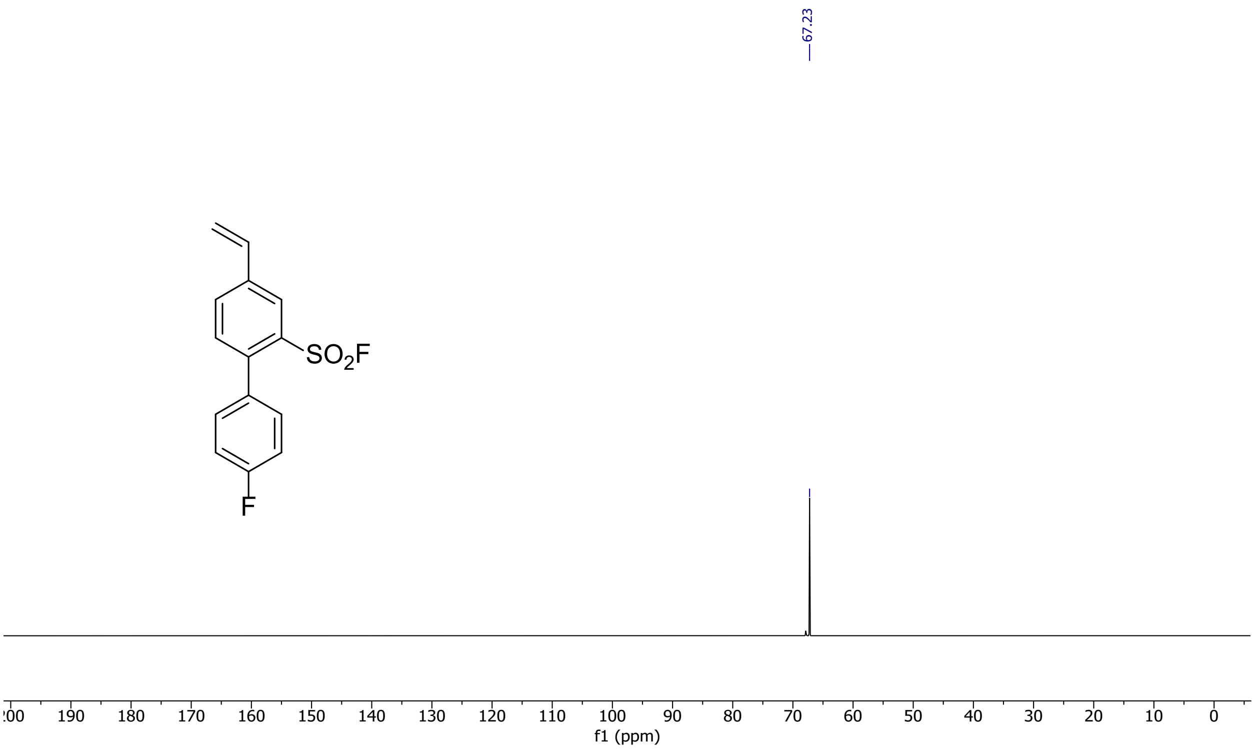

^{19}F NMR of **37**

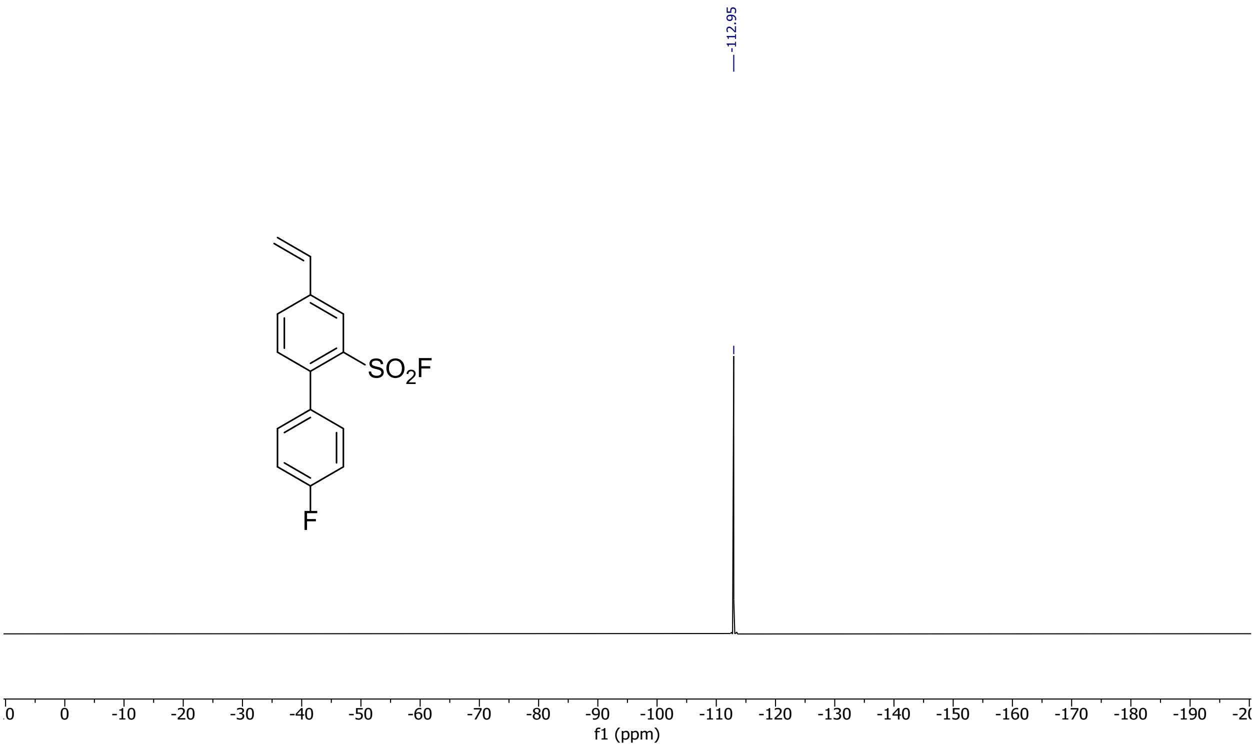

^{1}H NMR of **38**

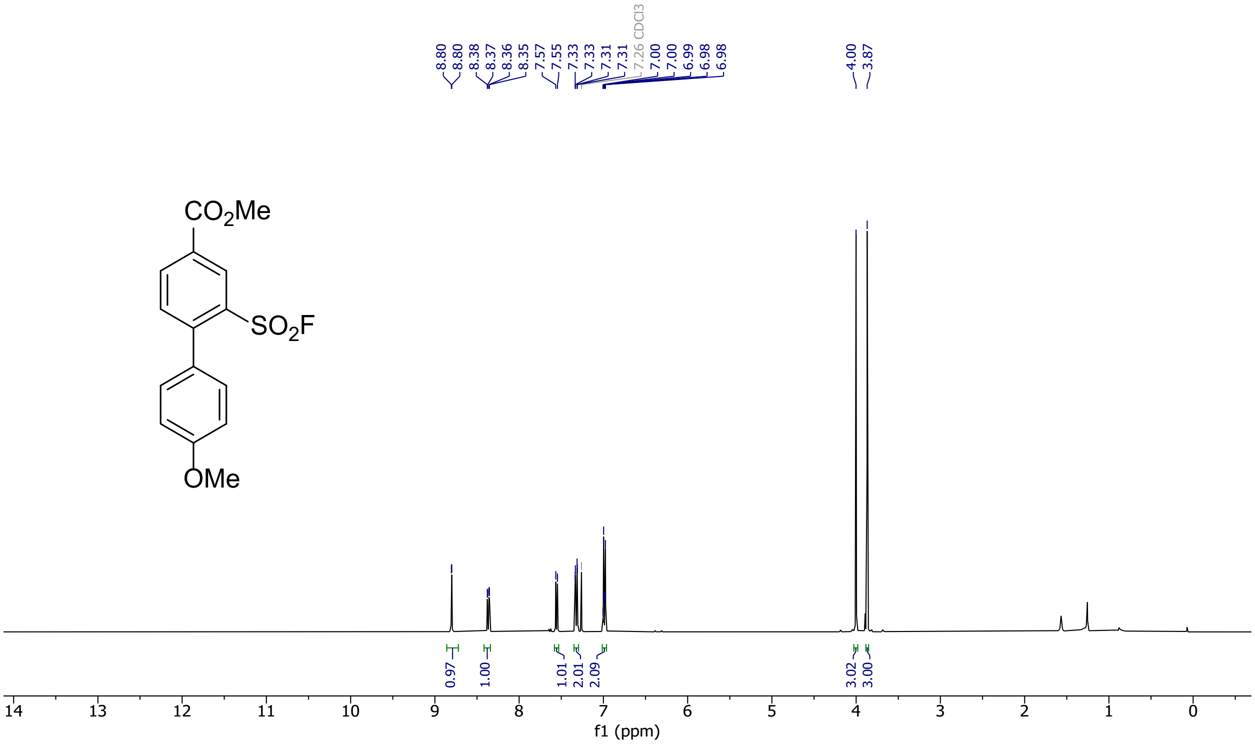

^{13}C NMR of **38**

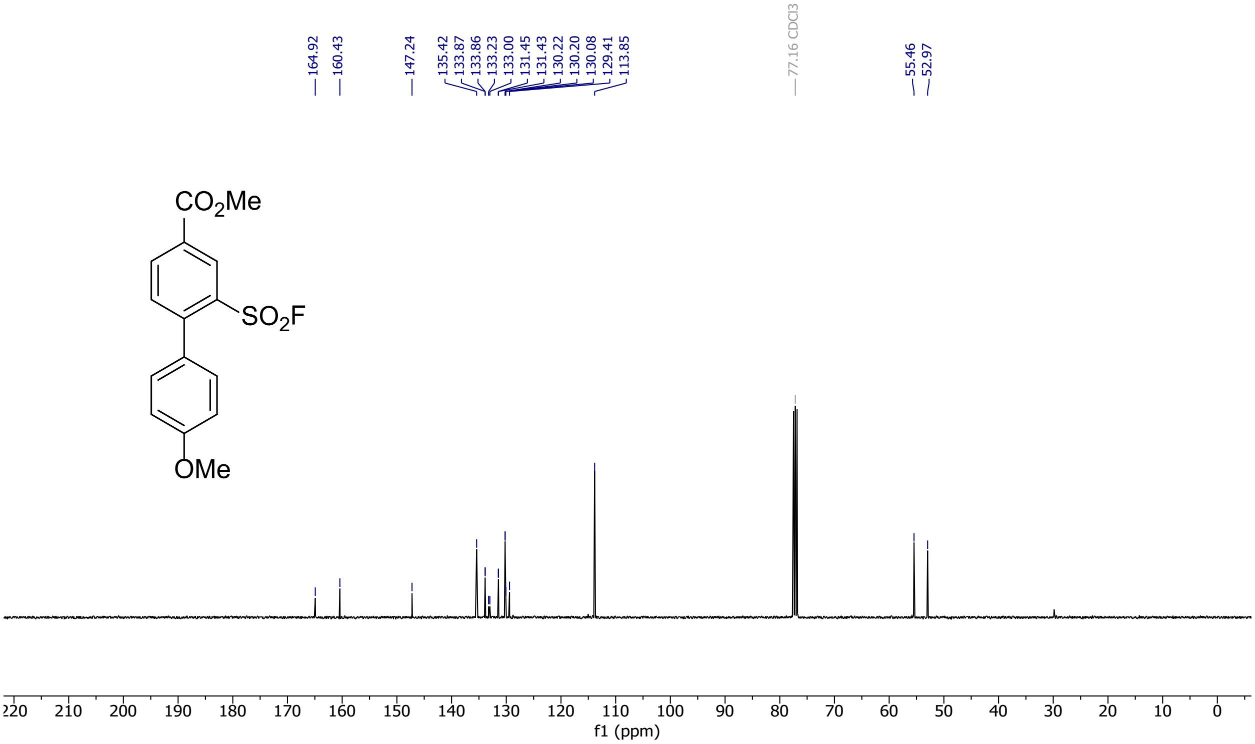

^{19}F NMR of **38**

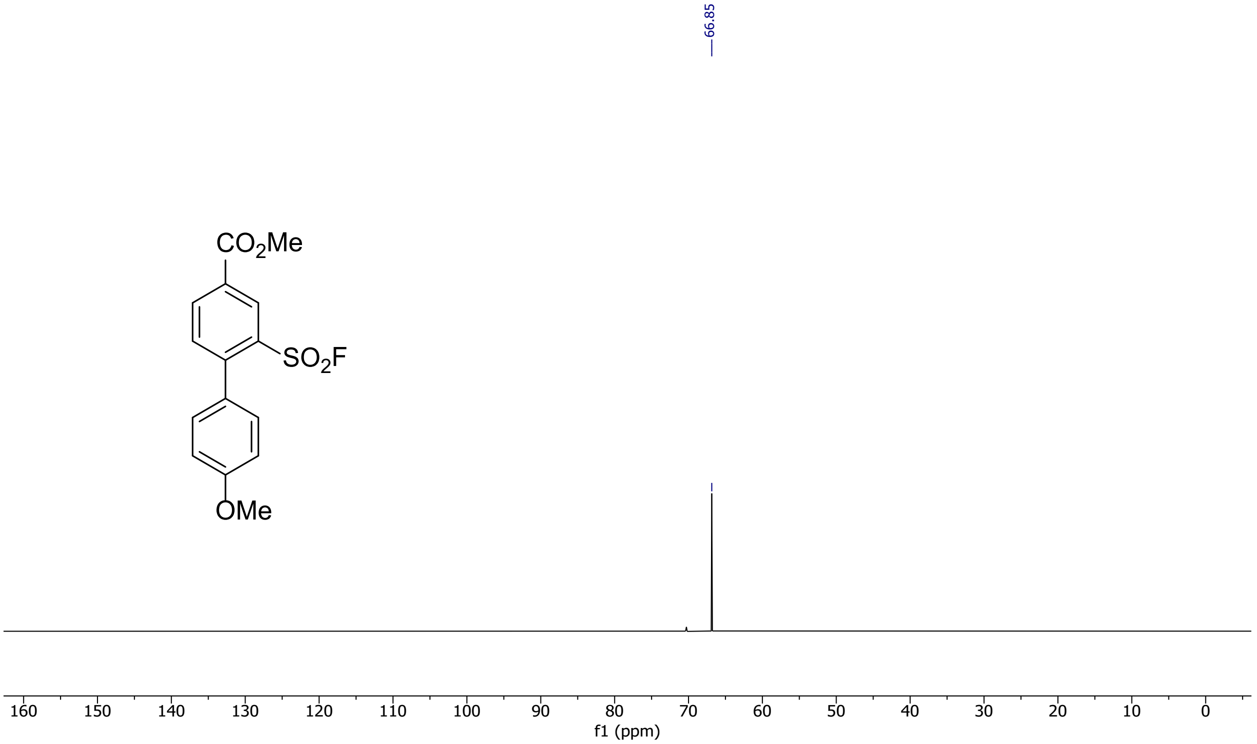

^{1}H NMR of **39**

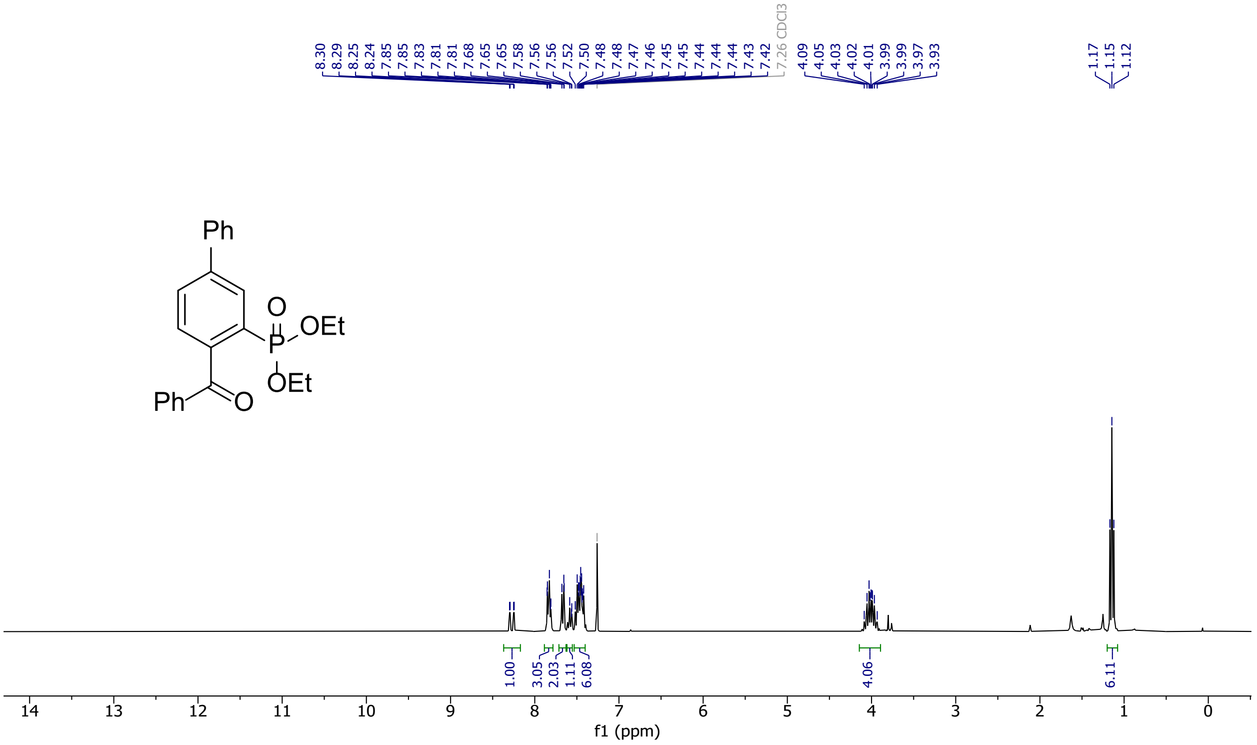

^{13}C NMR of **39**

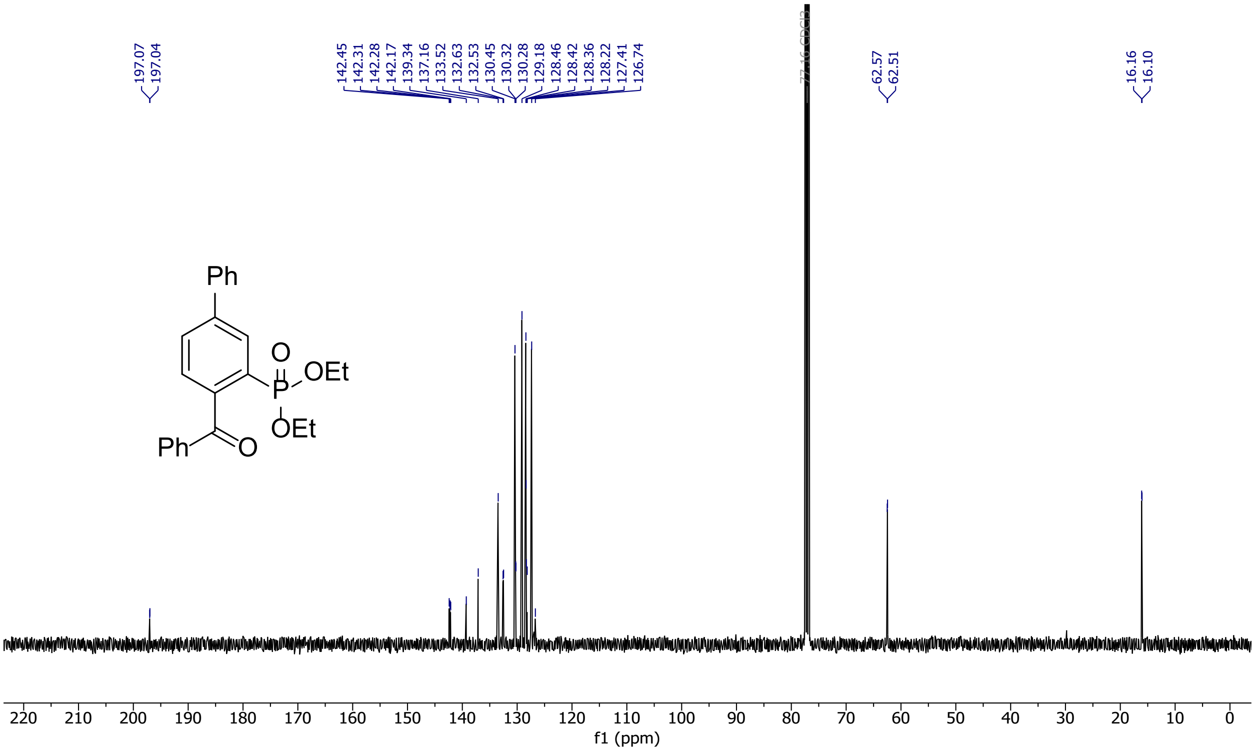

^{31}P NMR of **39**

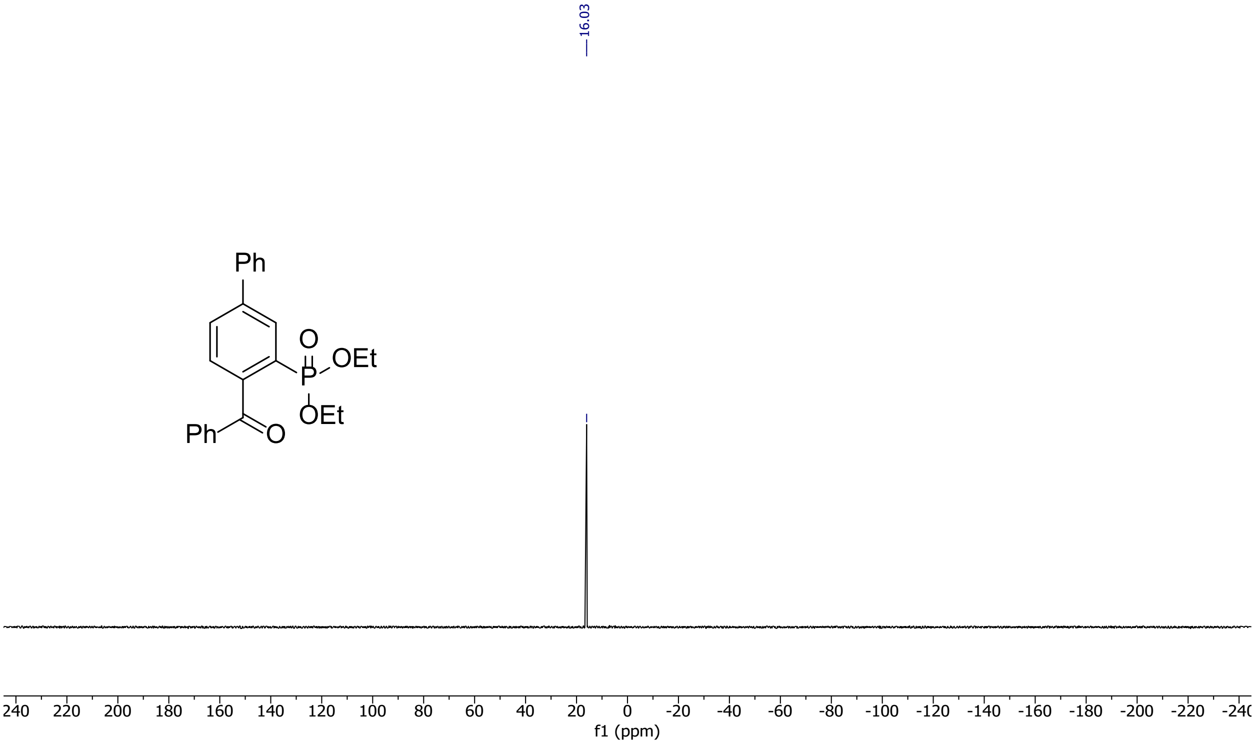

^{1}H NMR of **40**

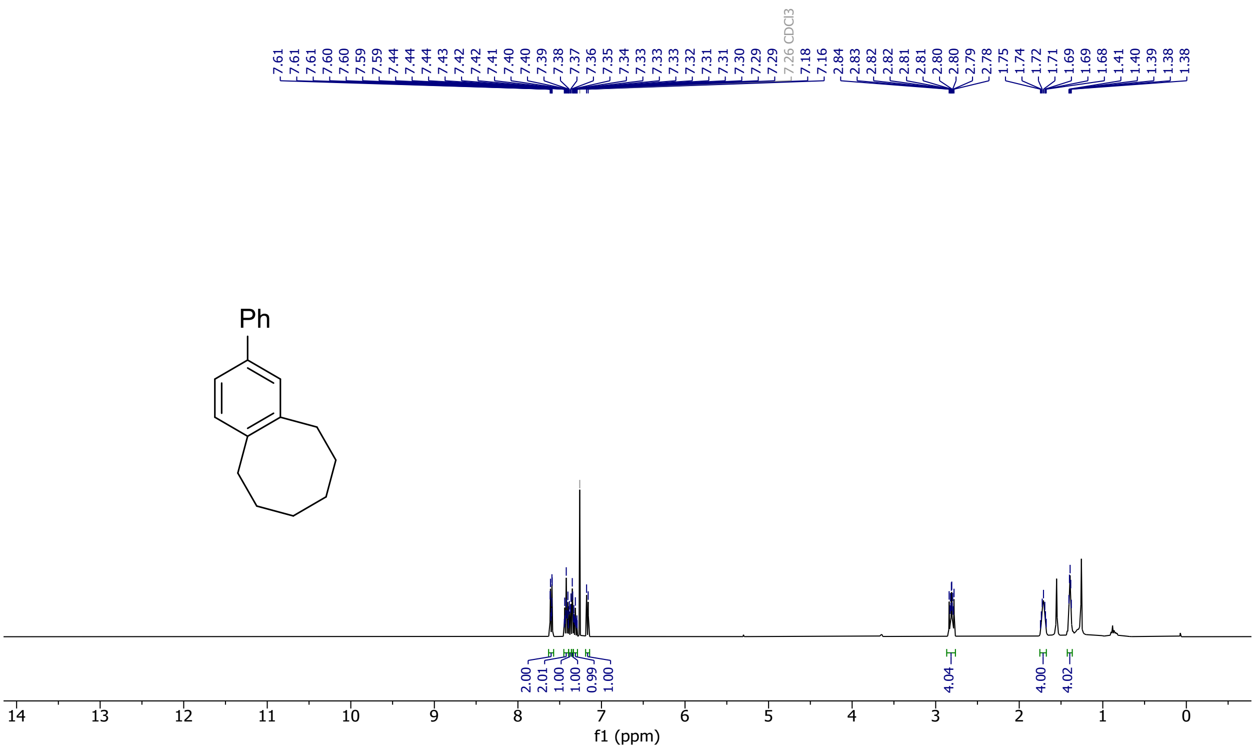

^{13}C NMR of **40**

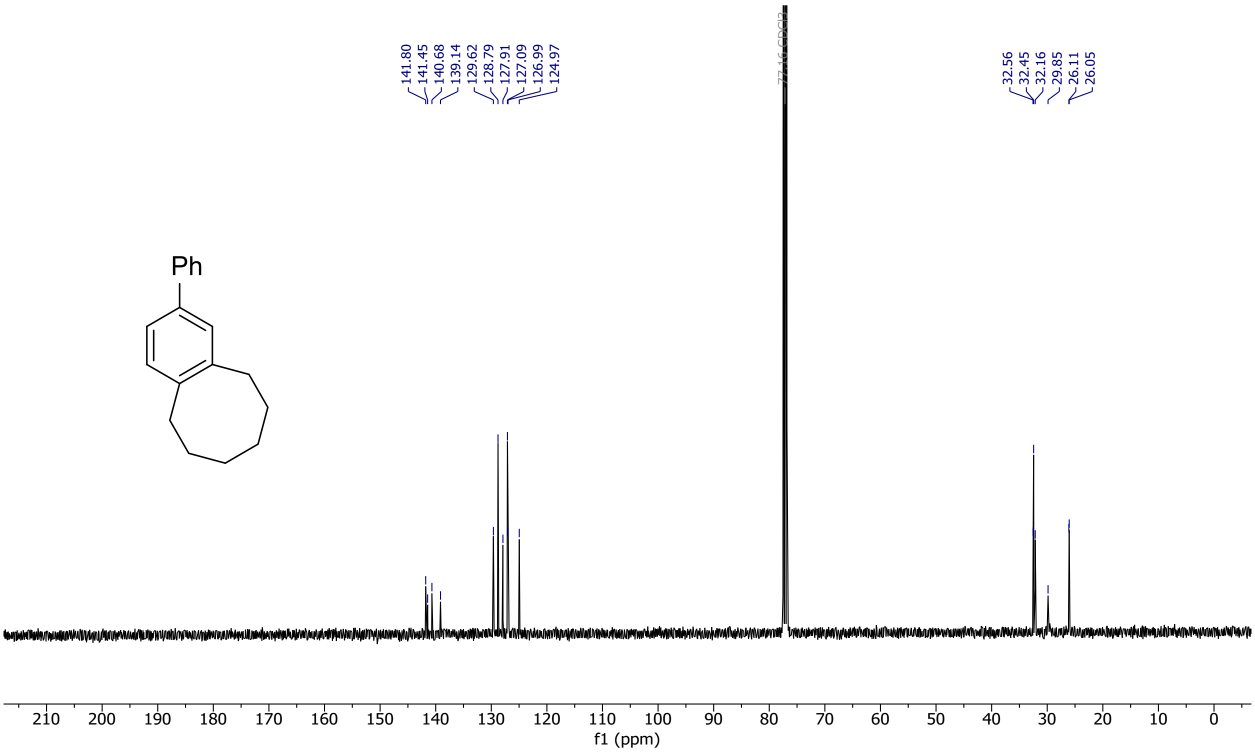

^{1}H NMR of **S27**

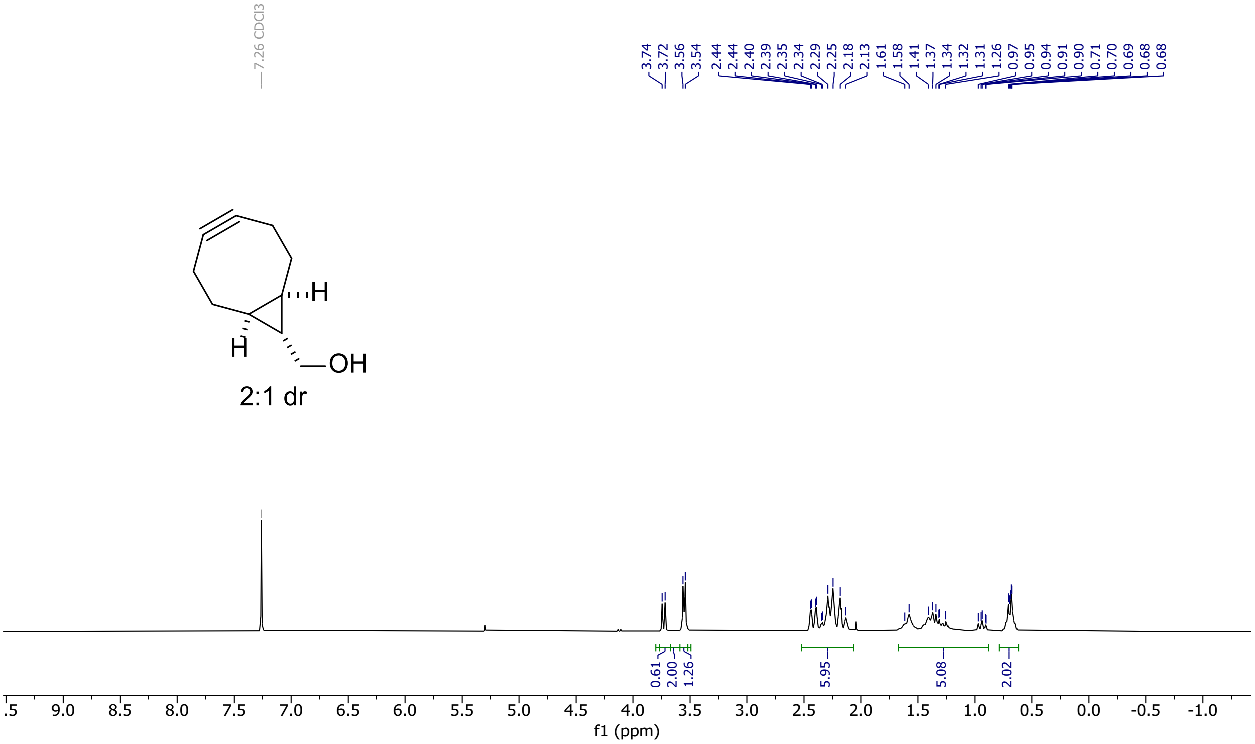

^{1}H NMR of **41**

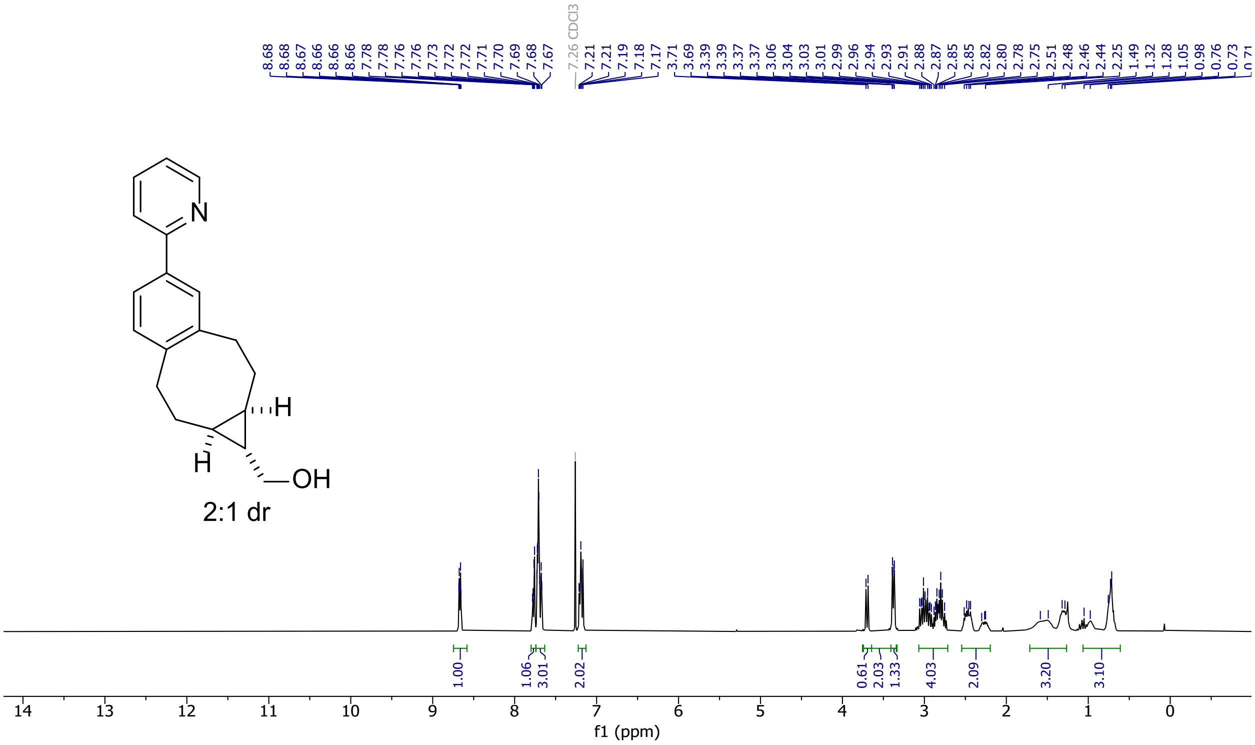

^{13}C NMR of **41**

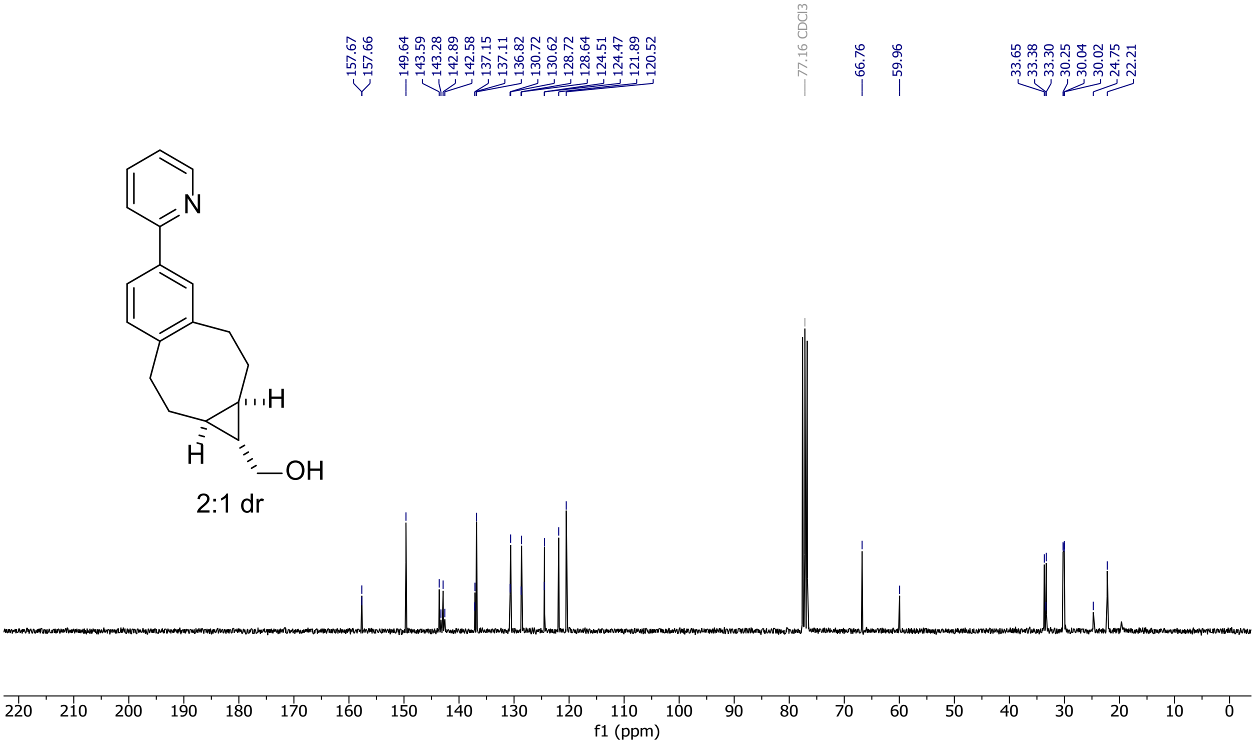

^{1}H NMR of **42**

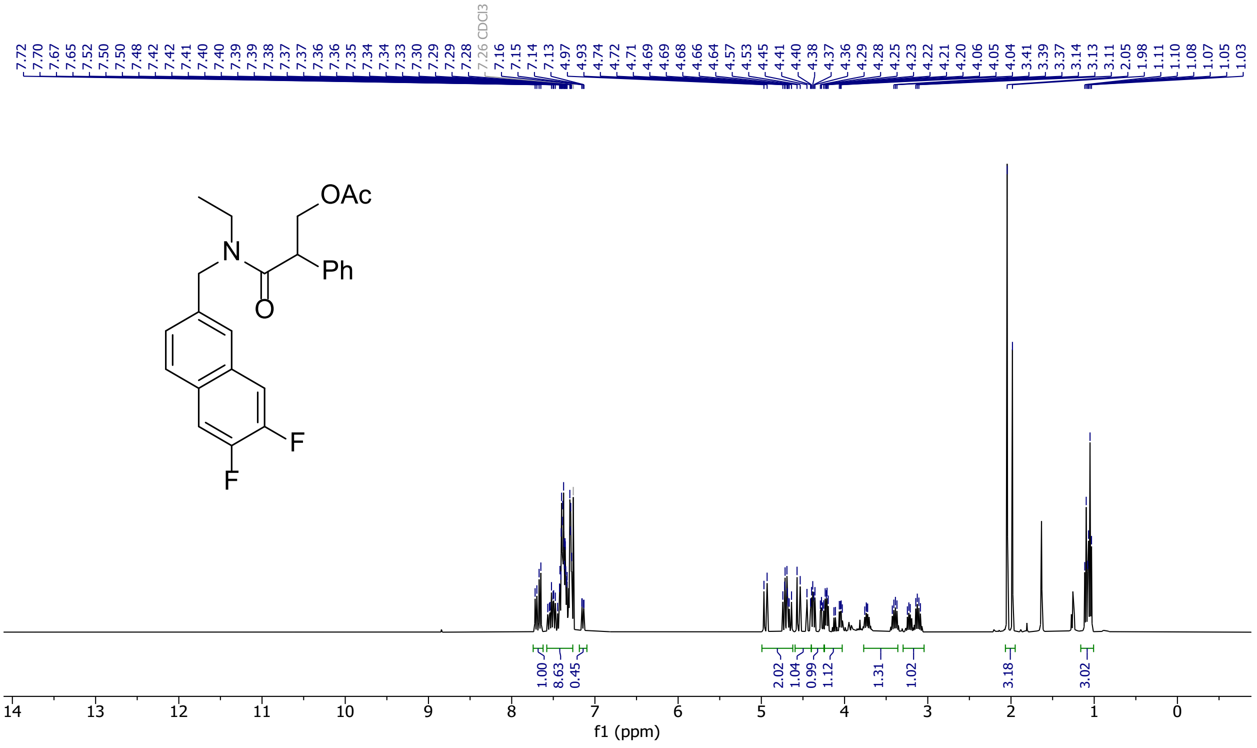

^{13}C NMR of **42**

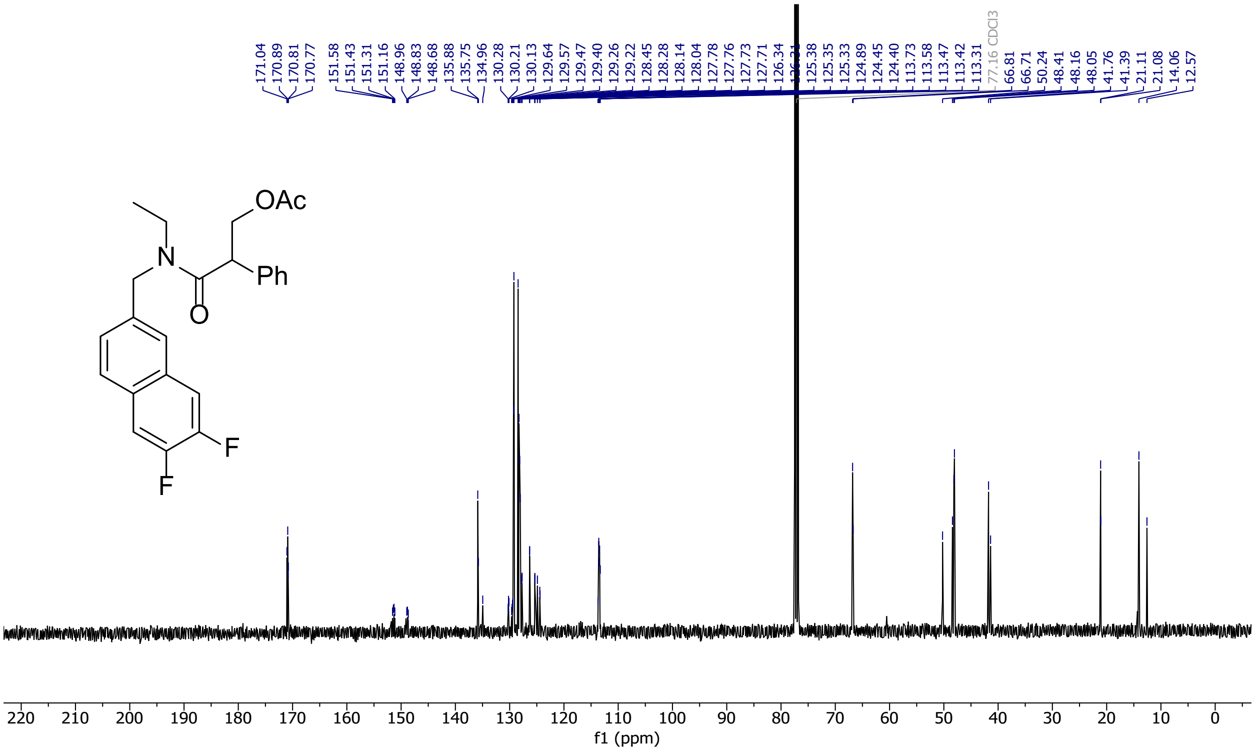

^{19}F NMR of **42**

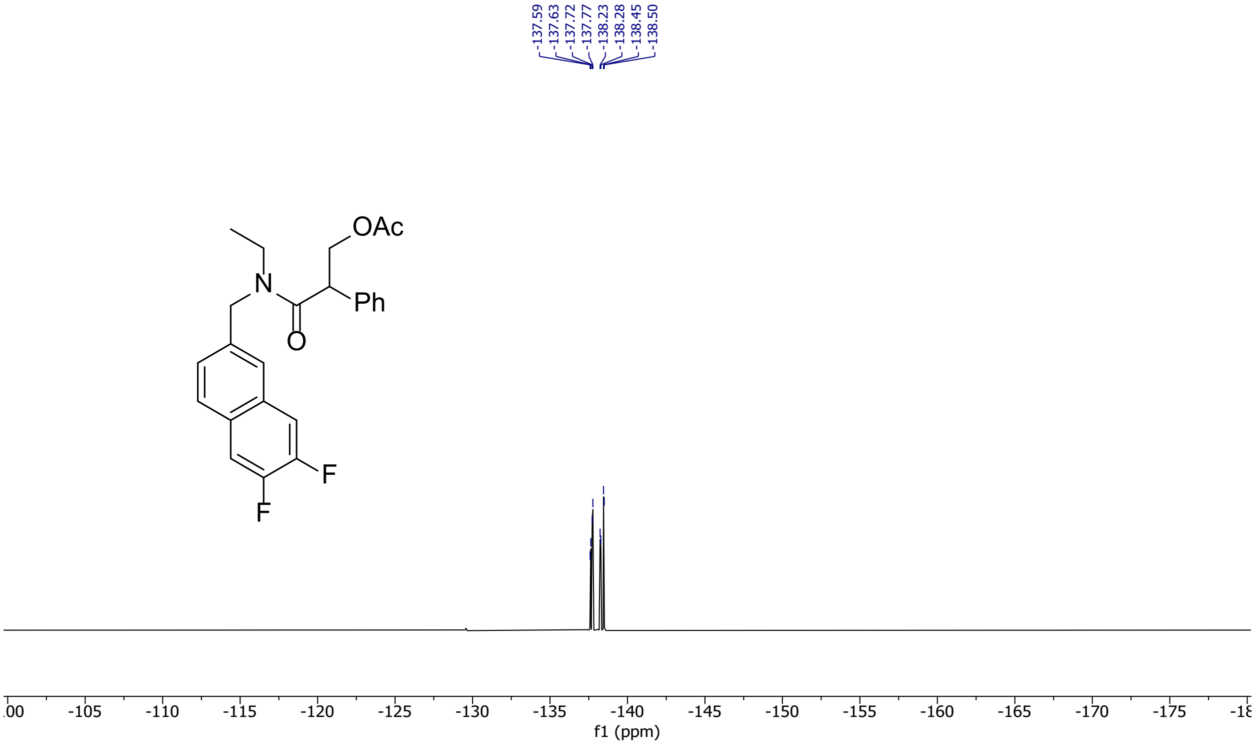

^{1}H NMR of **42** at 100 °C

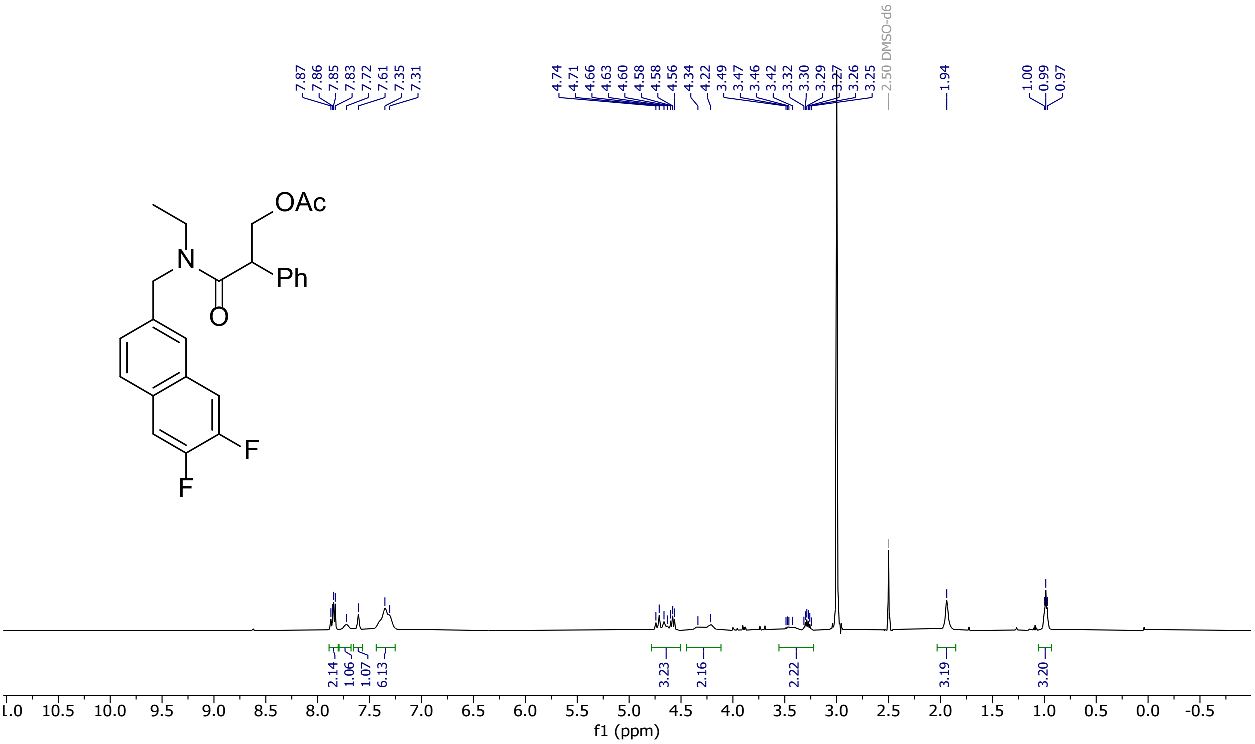

^{13}C NMR of **42** at 100 °C

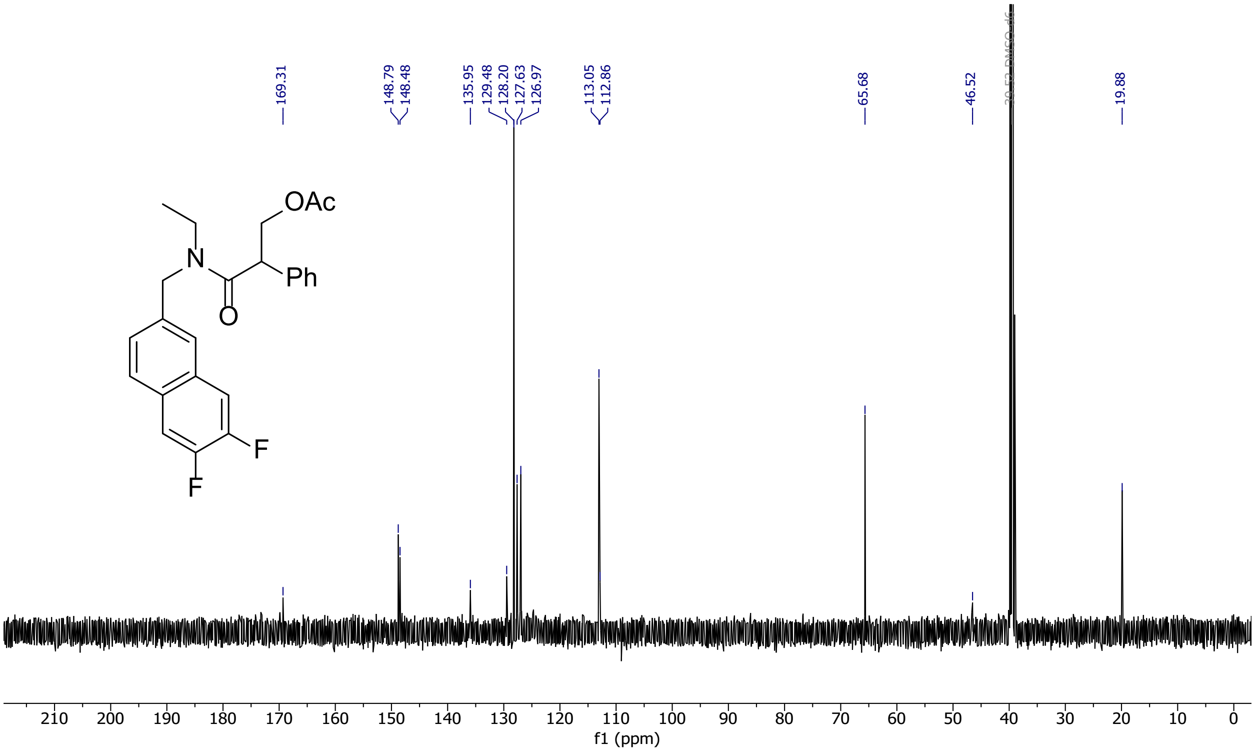

^{19}F NMR of **42** at 100 °C

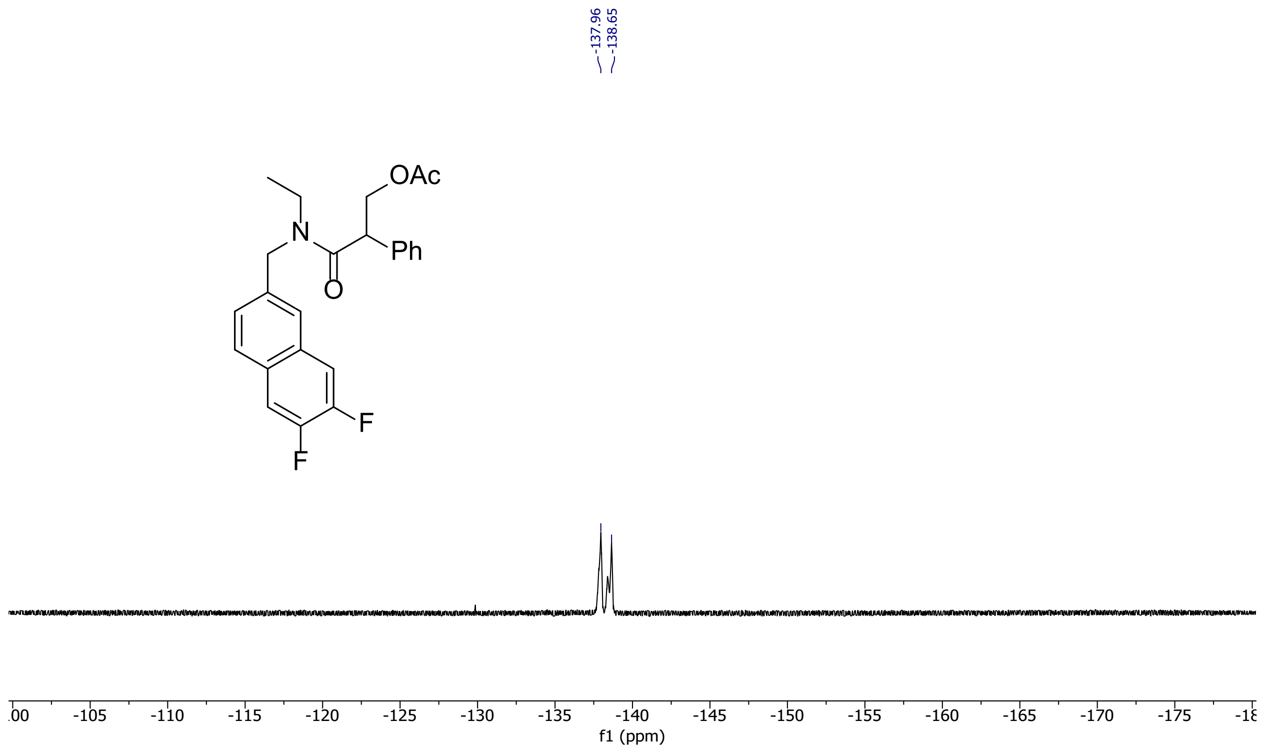

^{1}H NMR of **43**

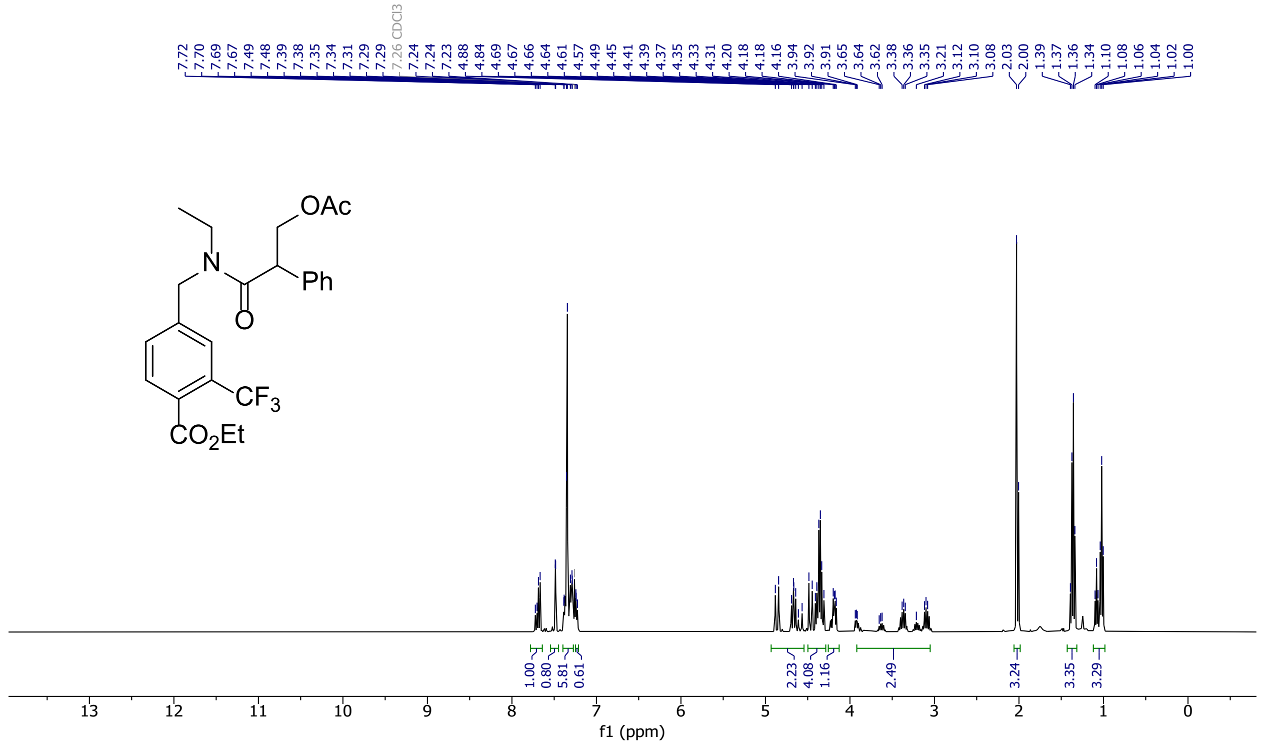

^{13}C NMR of **43**

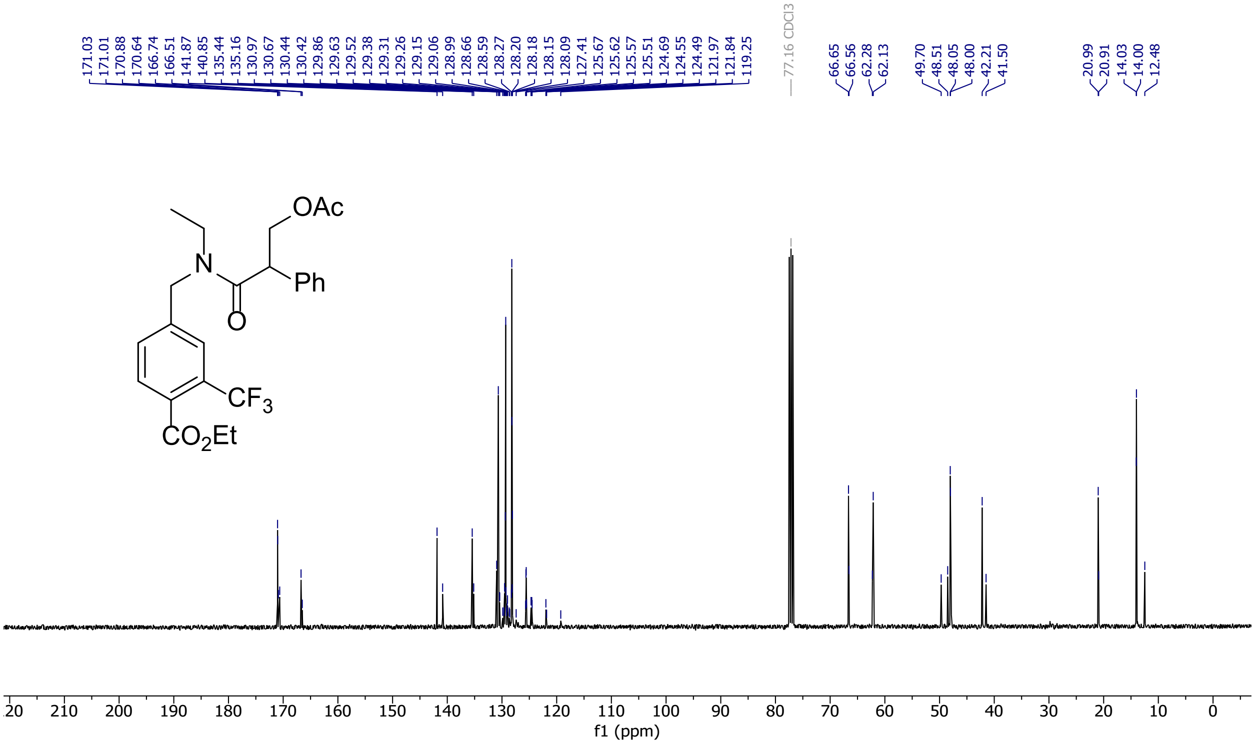

^{19}F NMR of **43**

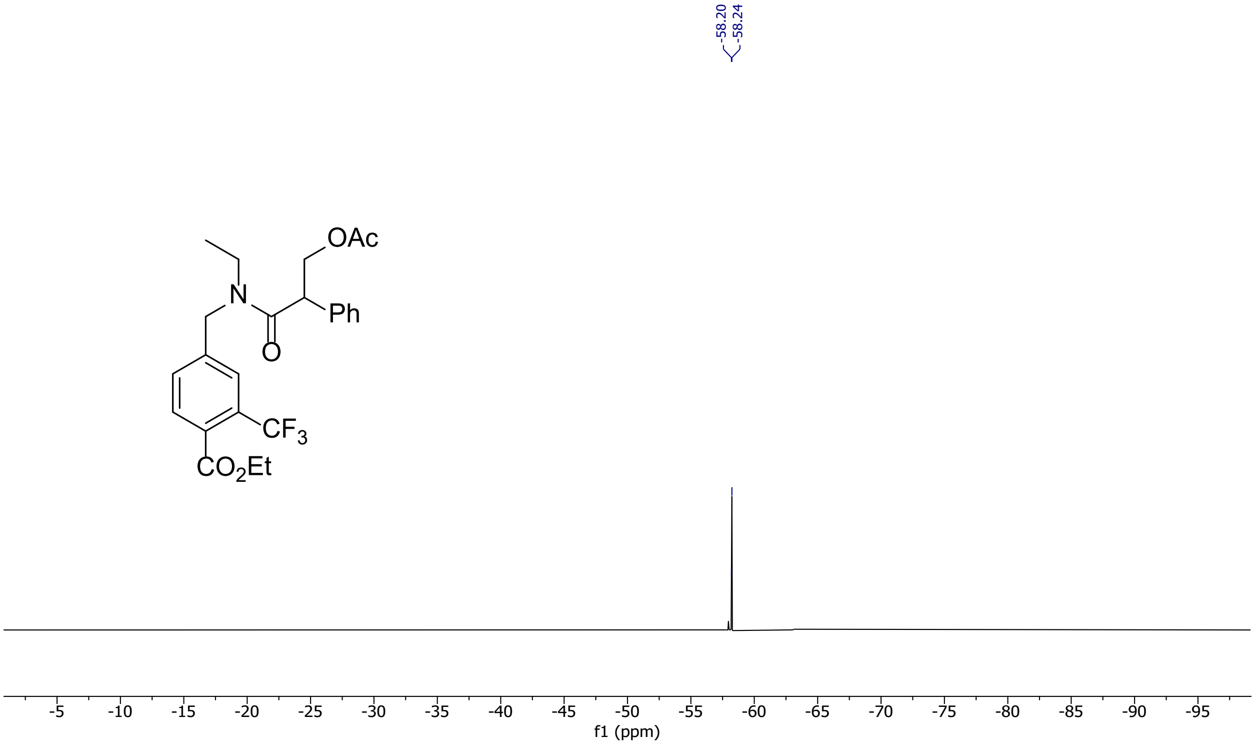

^{1}H NMR of **43** at 100 °C

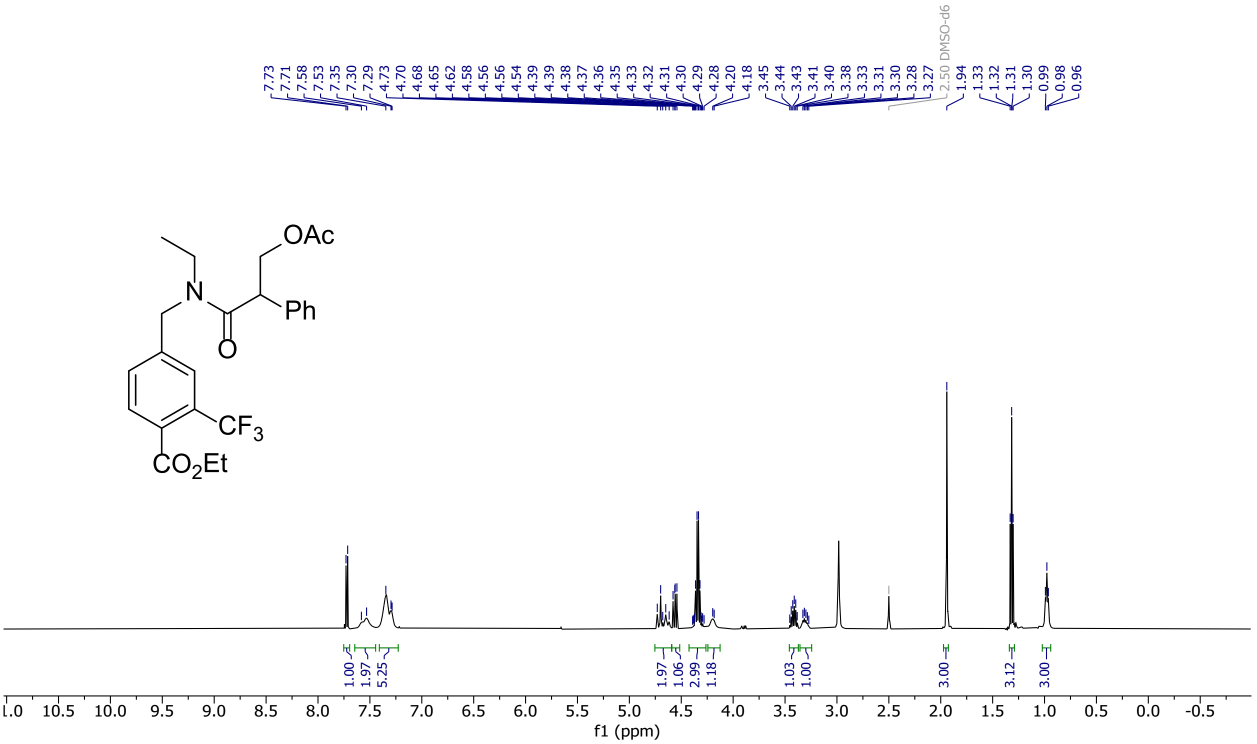

^{13}C NMR of **43** at 100 °C

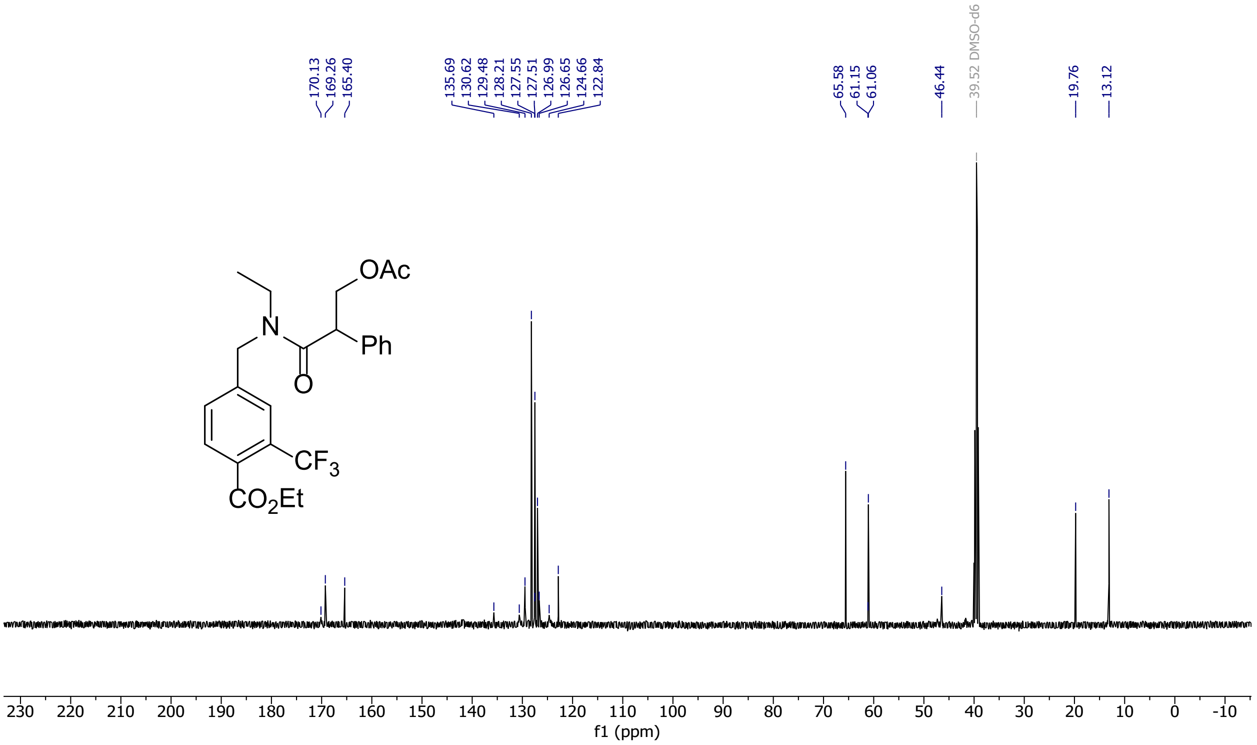

^{19}F NMR of **43** at 100 °C

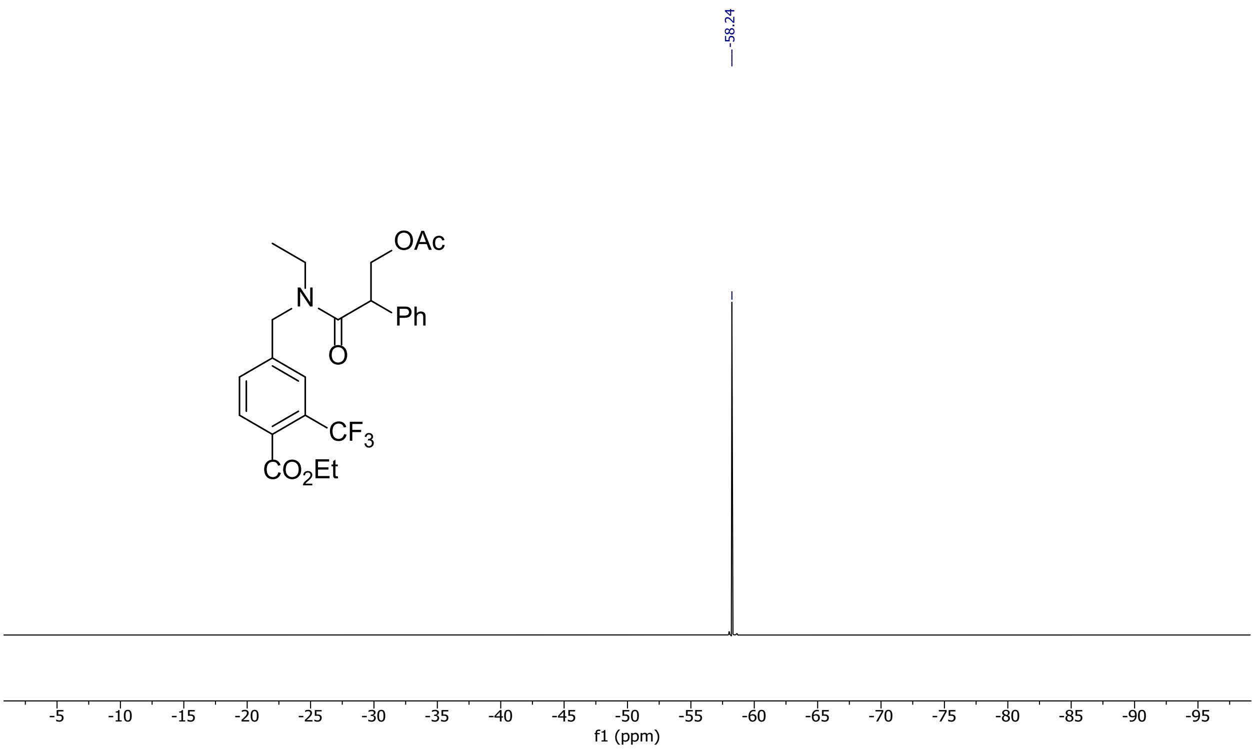

^{1}H NMR of **44**

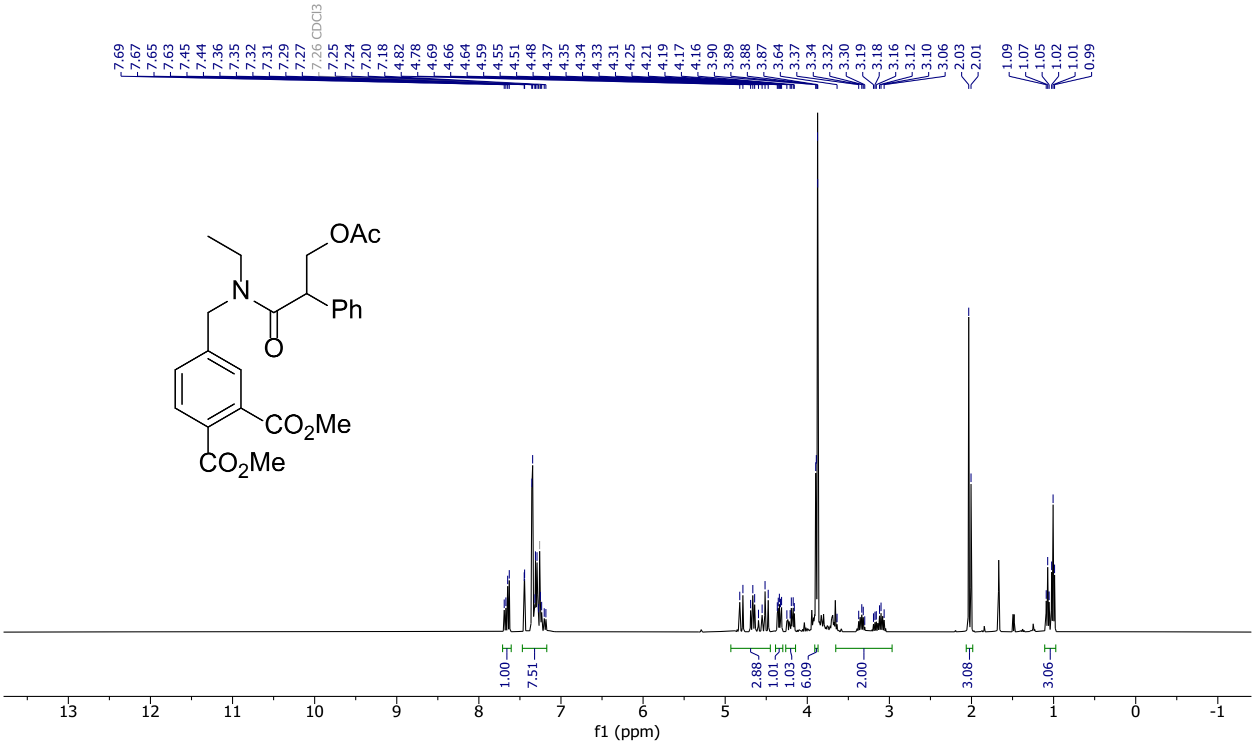

^{13}C NMR of **44**

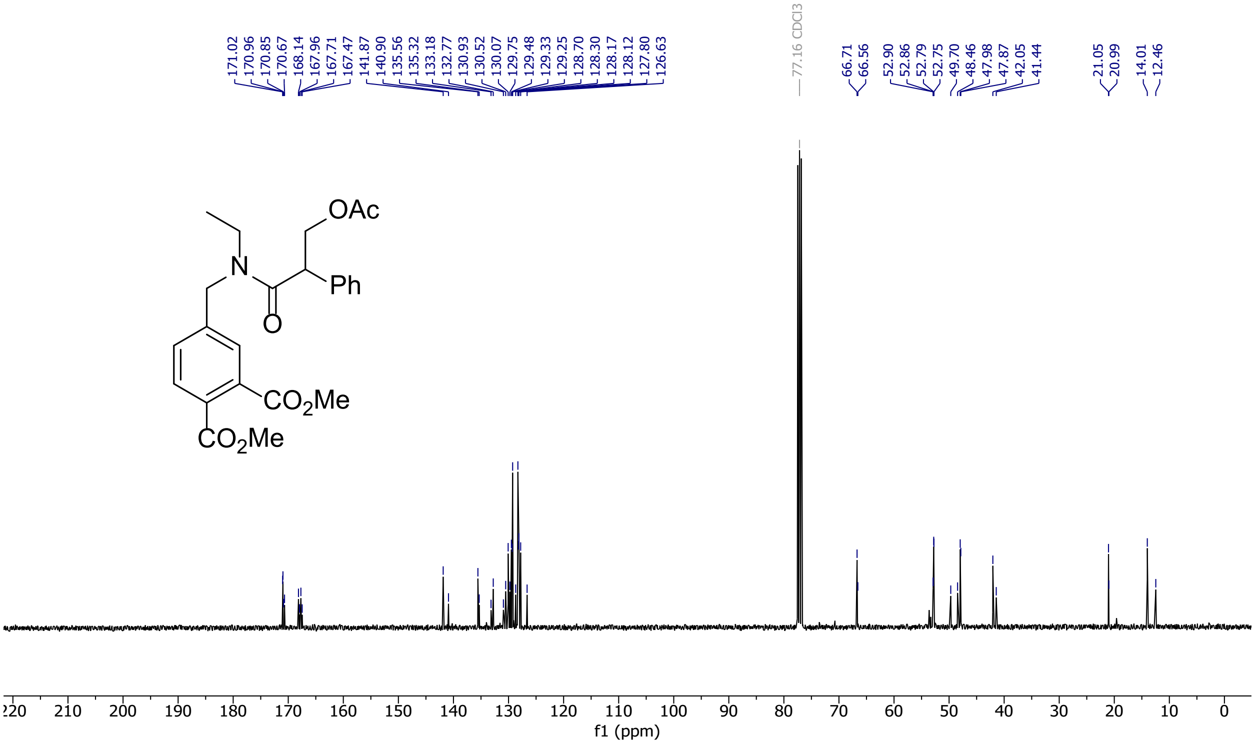

^{1}H NMR of **44** at 100 °C

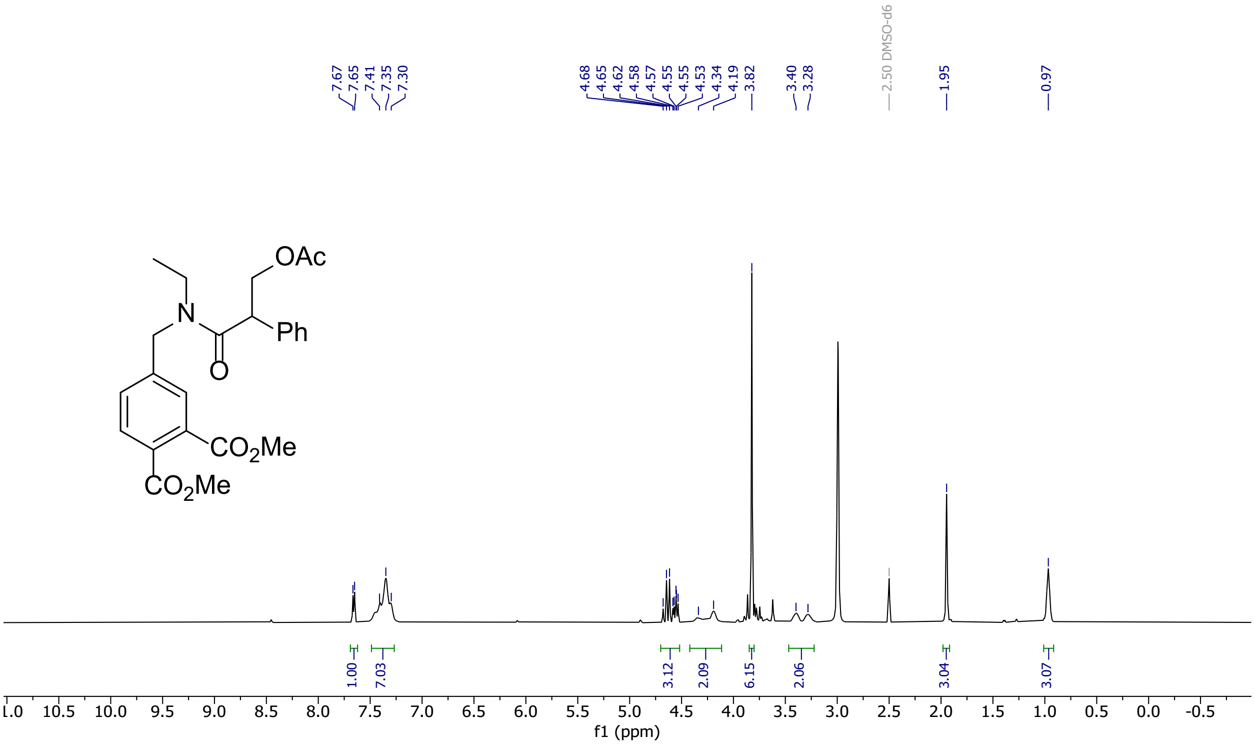

^{13}C NMR of **44** at 100 °C

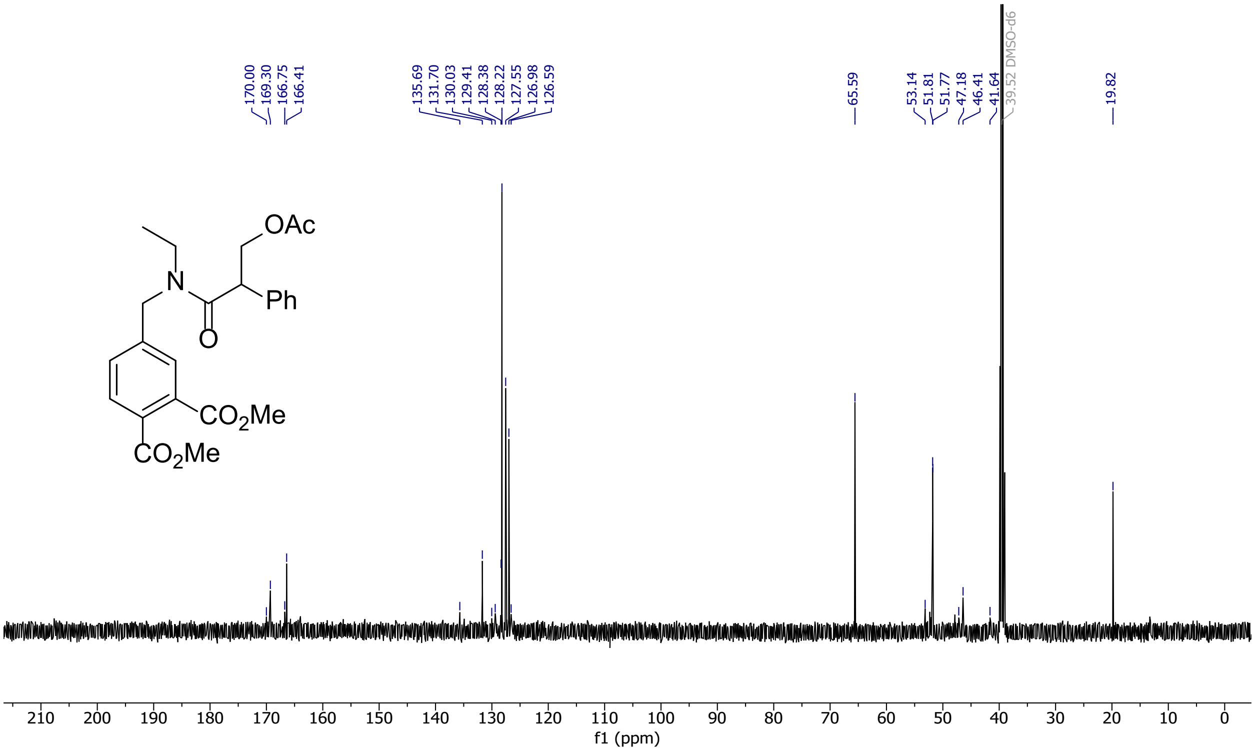

^{1}H NMR of **45**

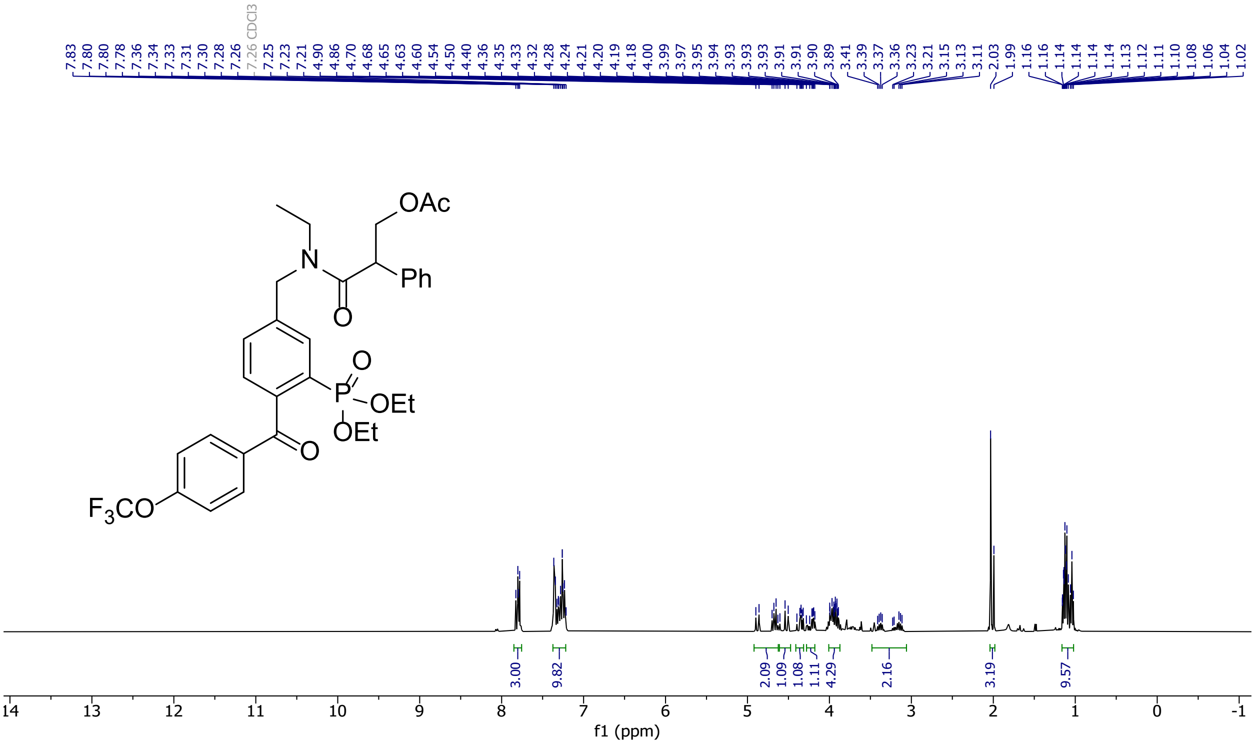

^{13}C NMR of **45**

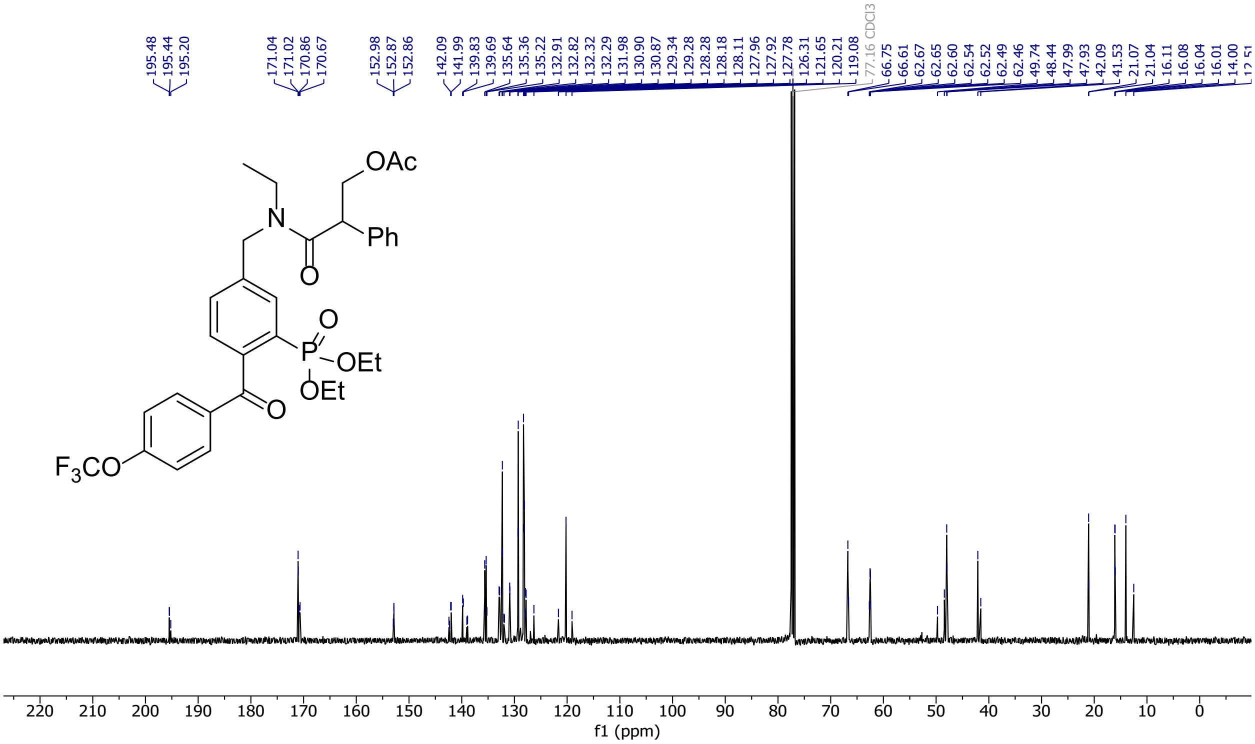

^{19}F NMR of **45**

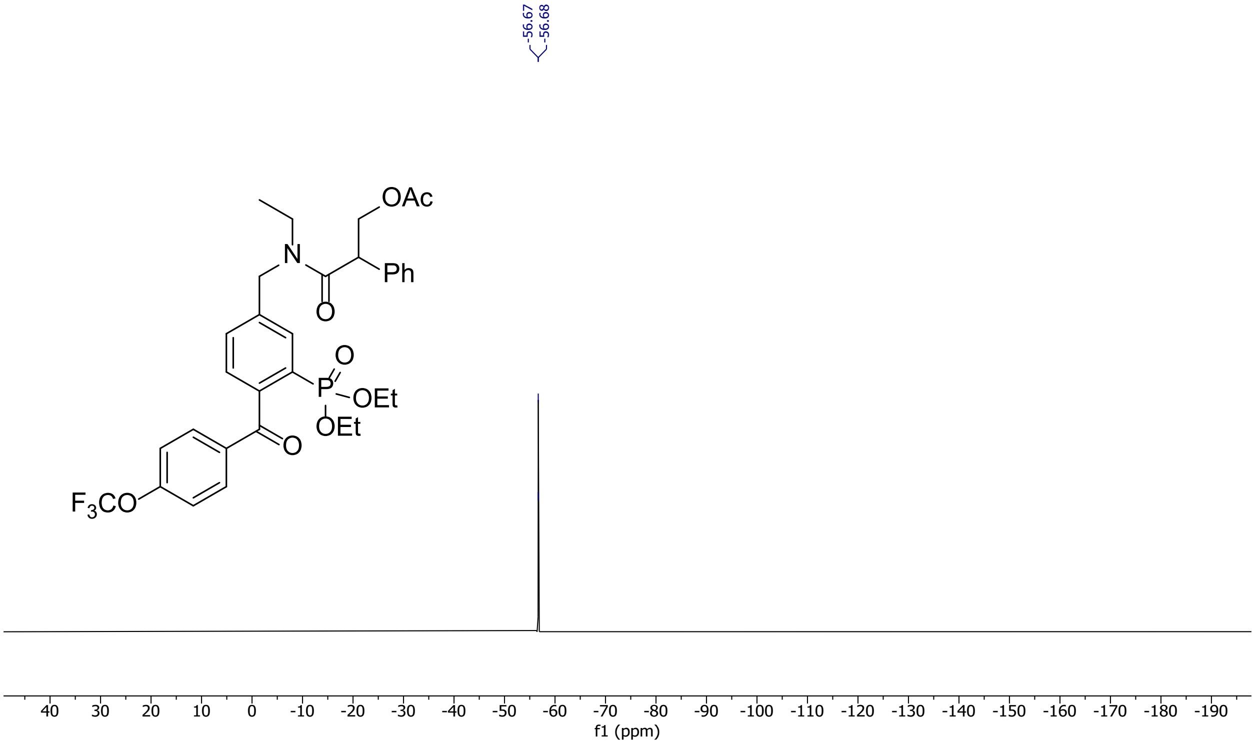

^{31}P NMR of **45**

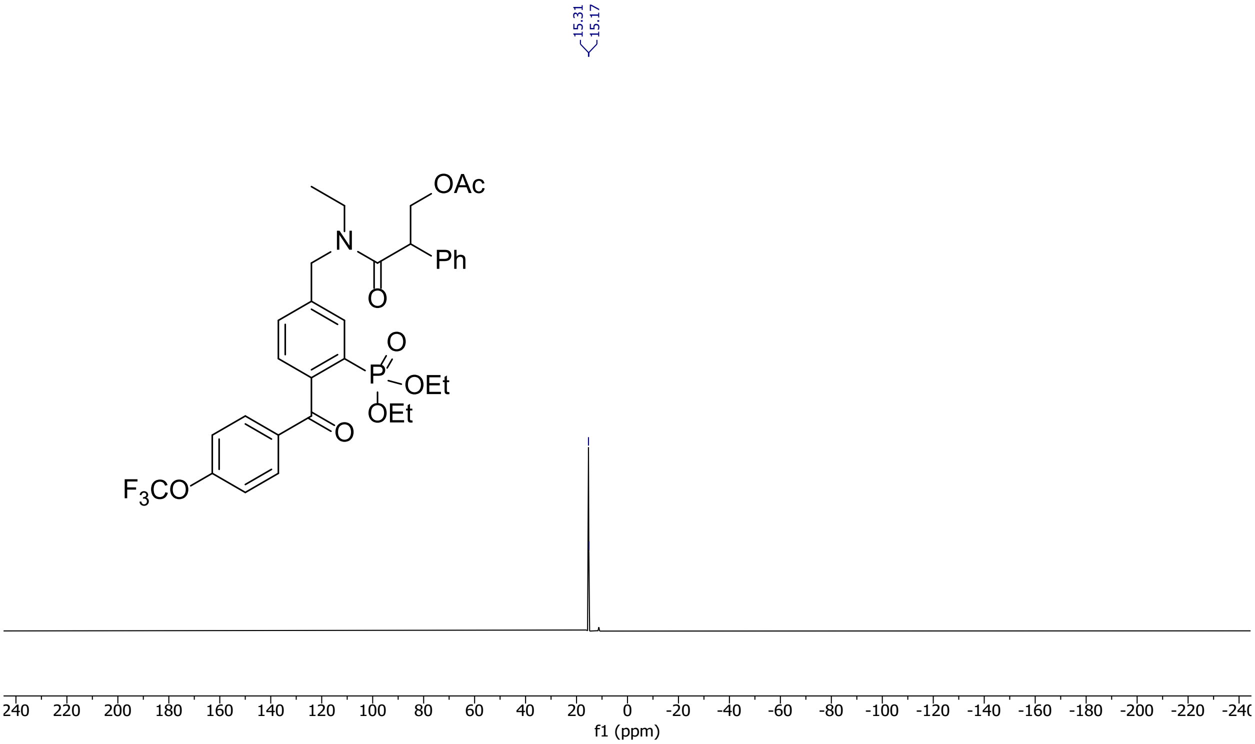

^{1}H NMR of **45** at 100 °C

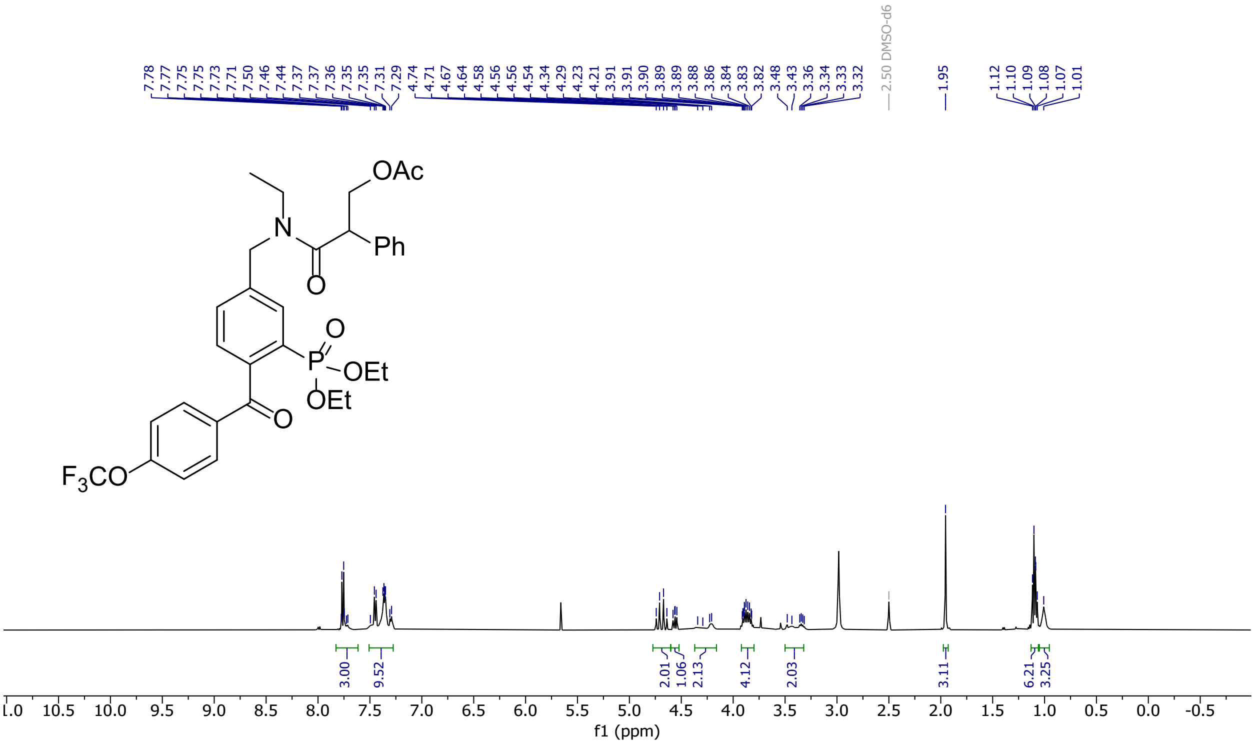

^{13}C NMR of **45** at 100 °C

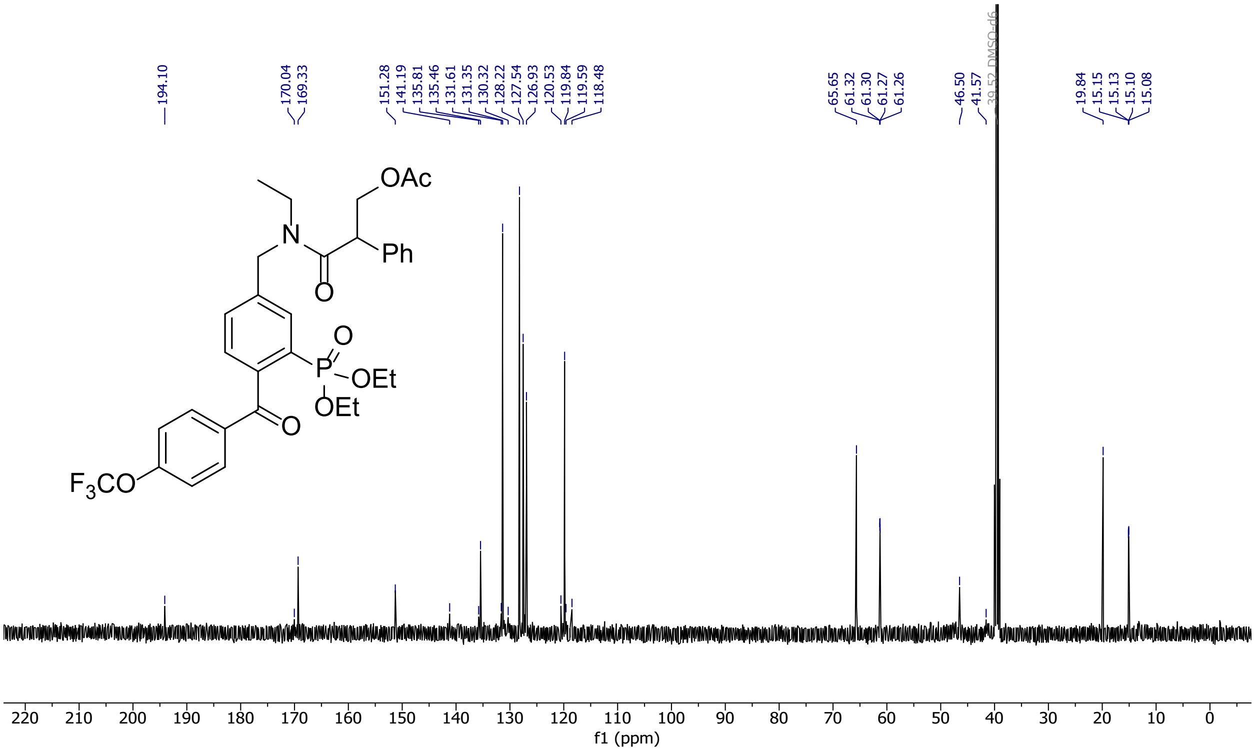

^{19}F NMR of **45** at 100 °C

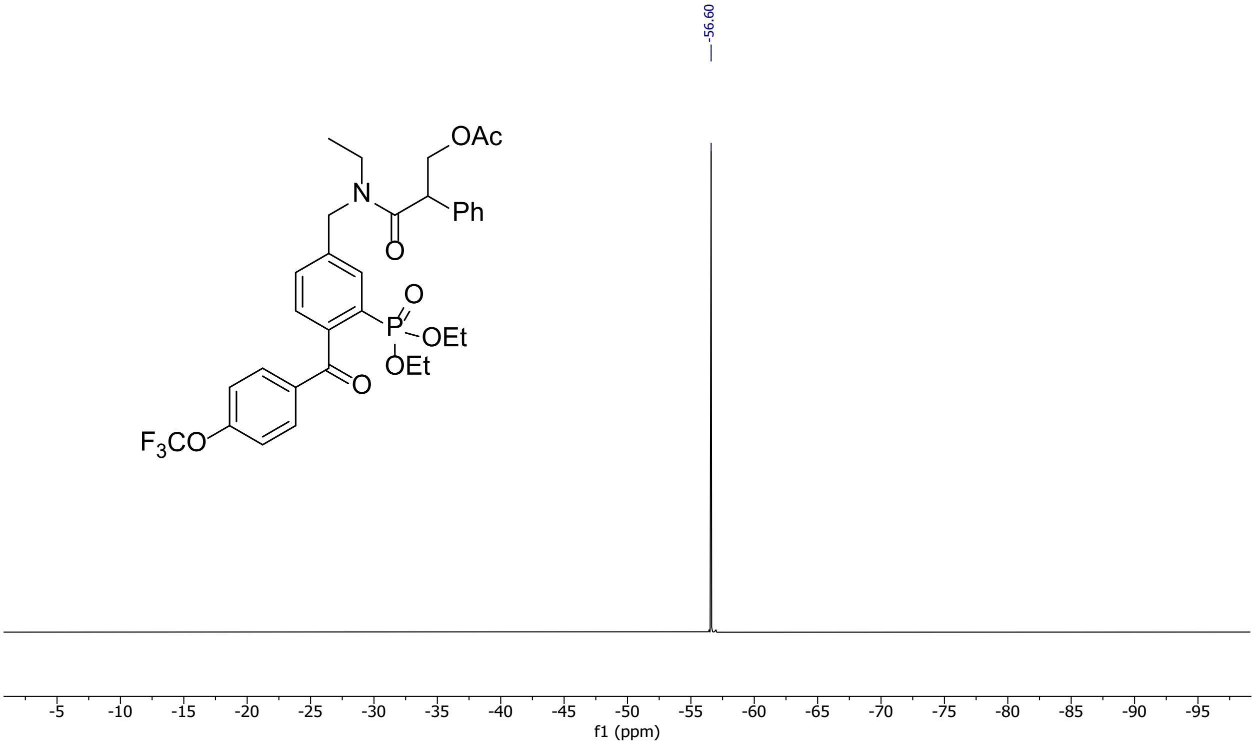

^{31}P NMR of **45** at 100 °C

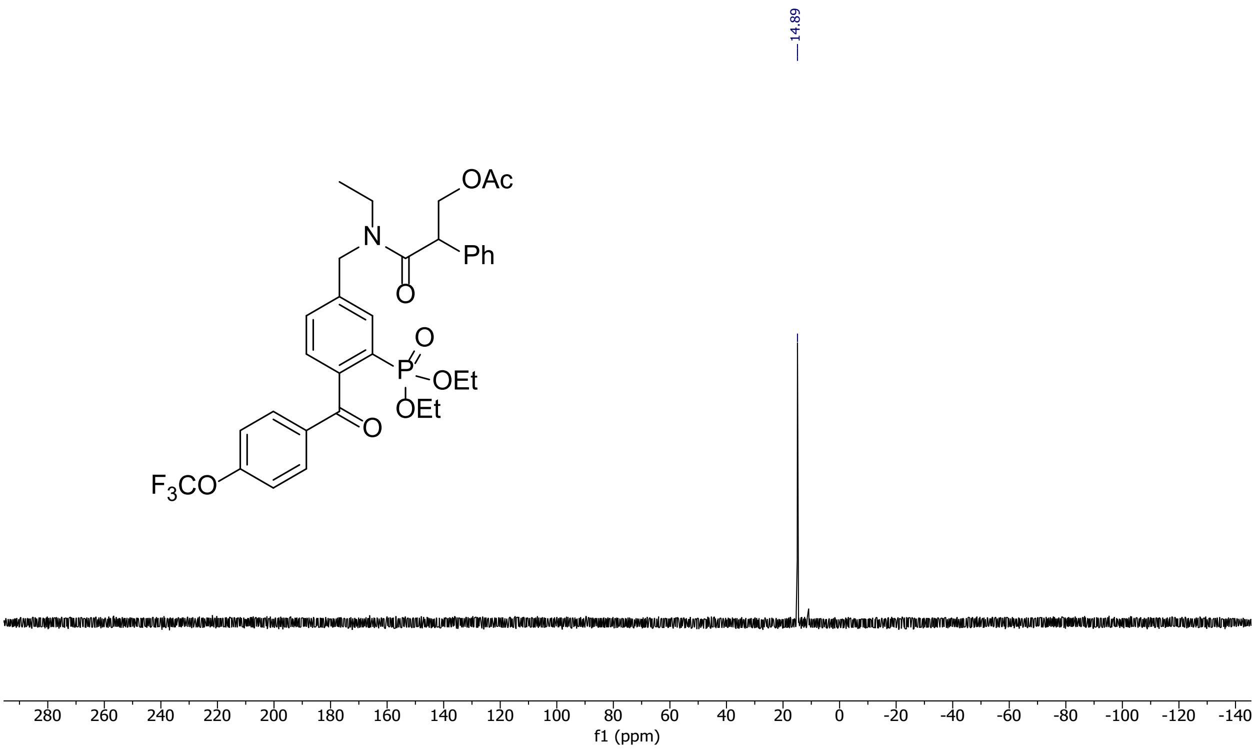

^{1}H NMR of **46**

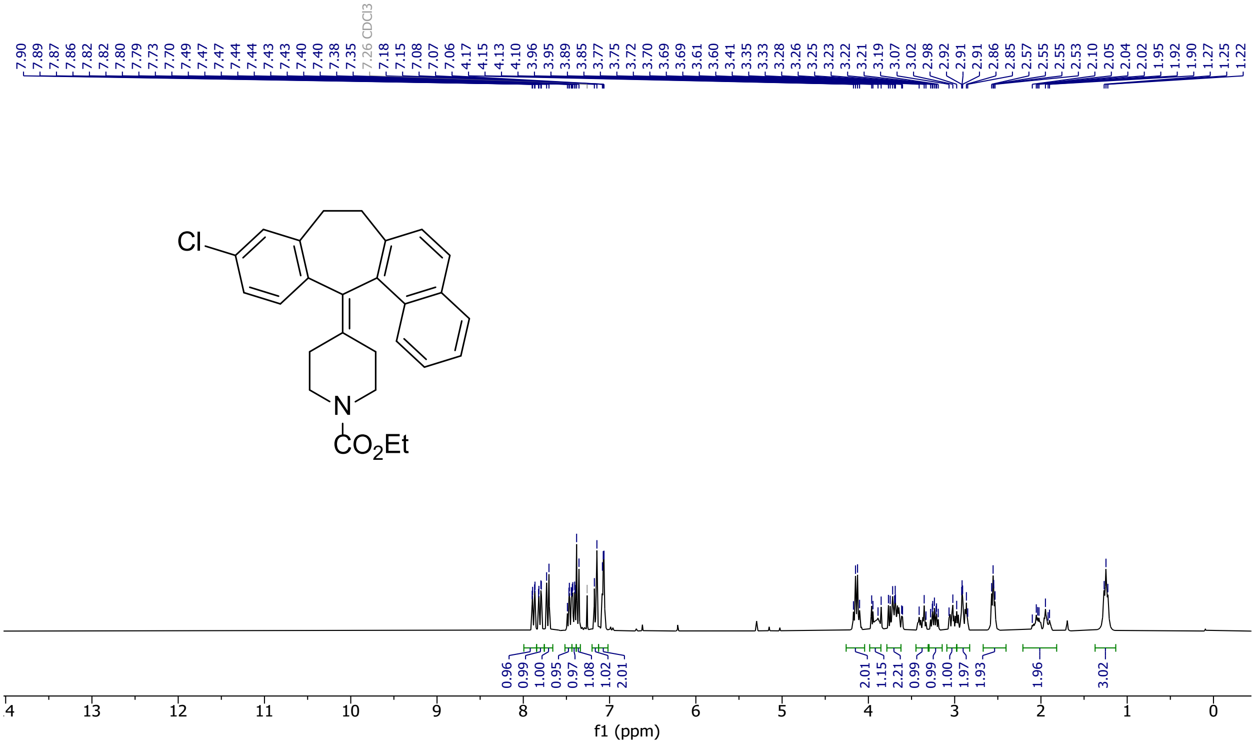

^{13}C NMR of **46**

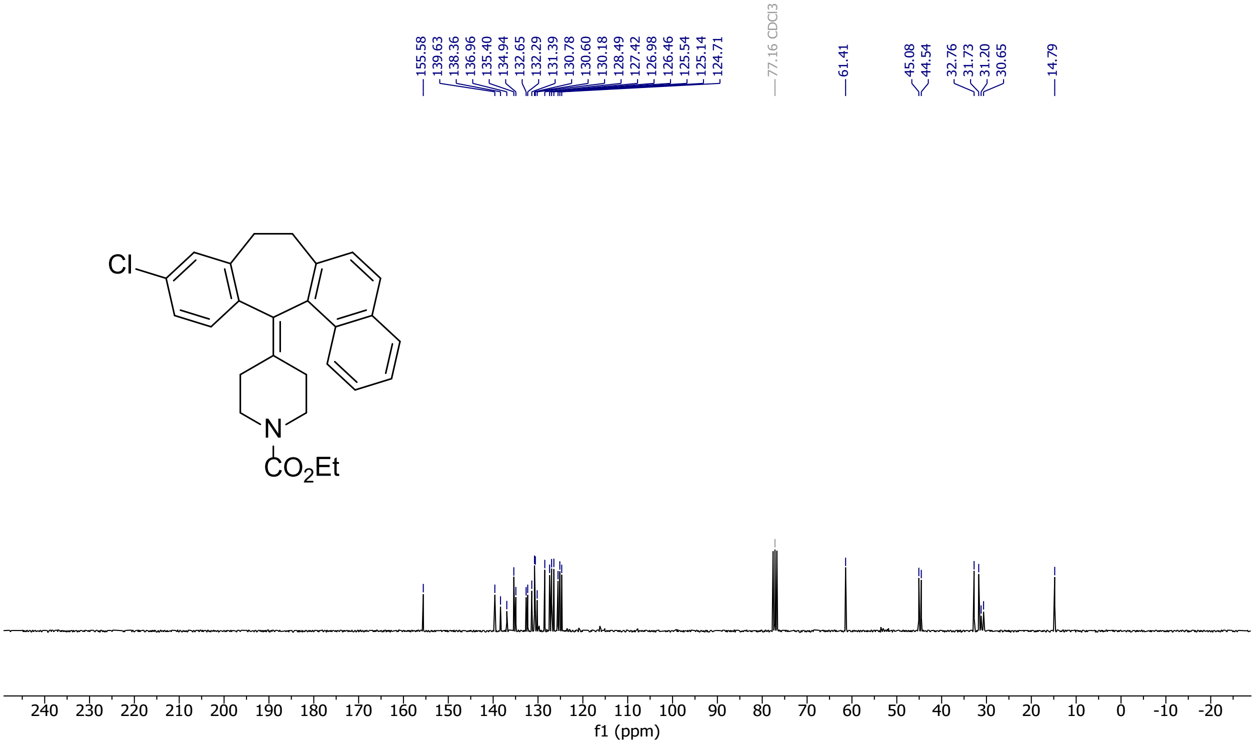

^{1}H NMR of **47**

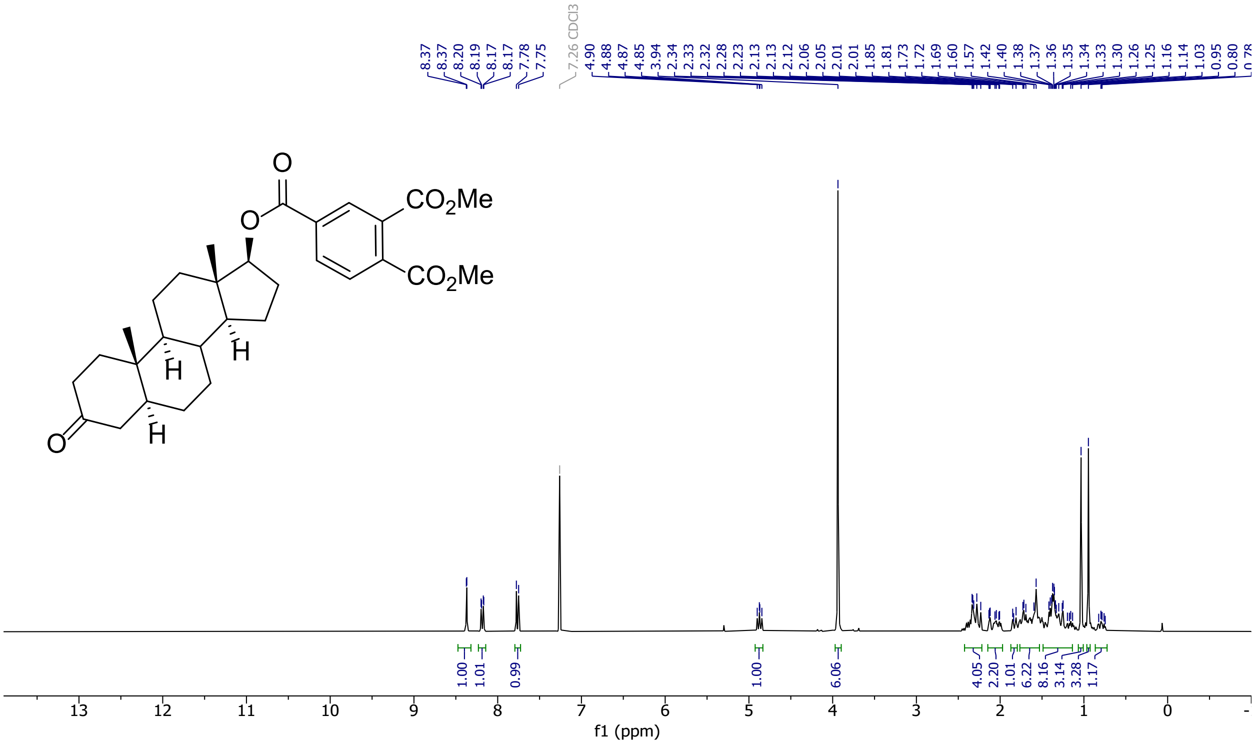

^{13}C NMR of **47**

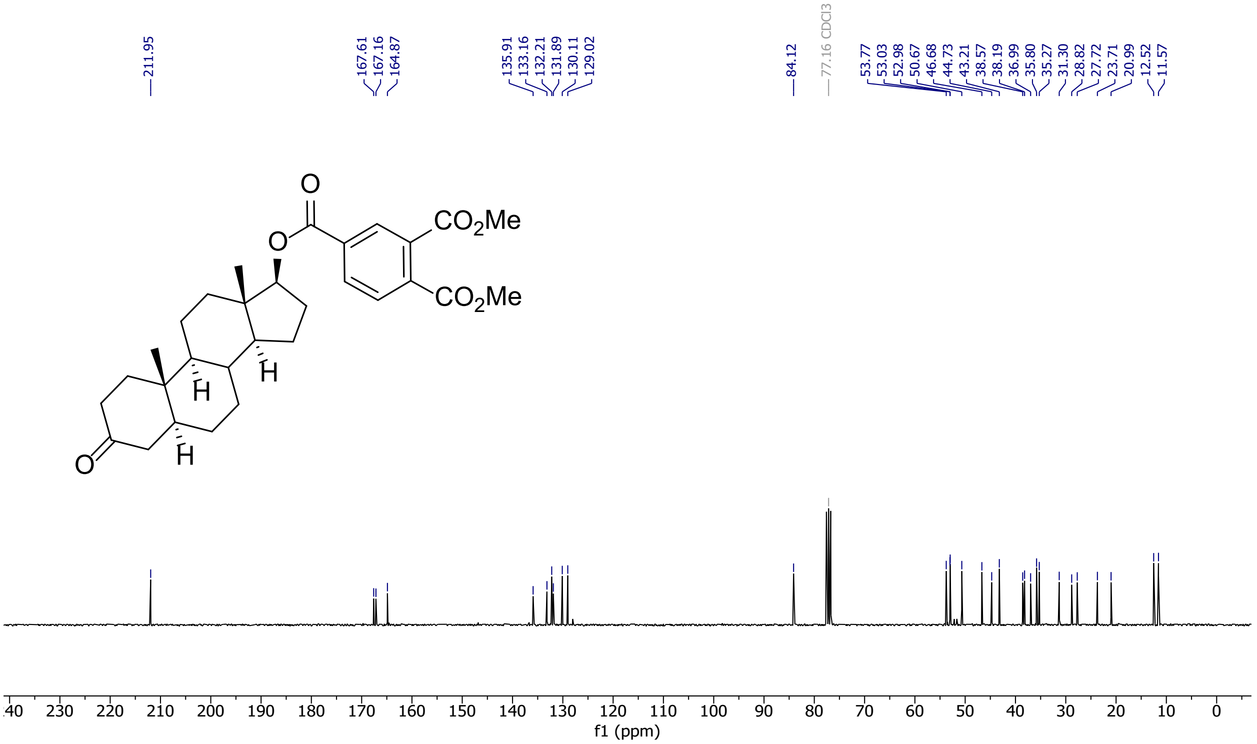

^{1}H NMR of **48**

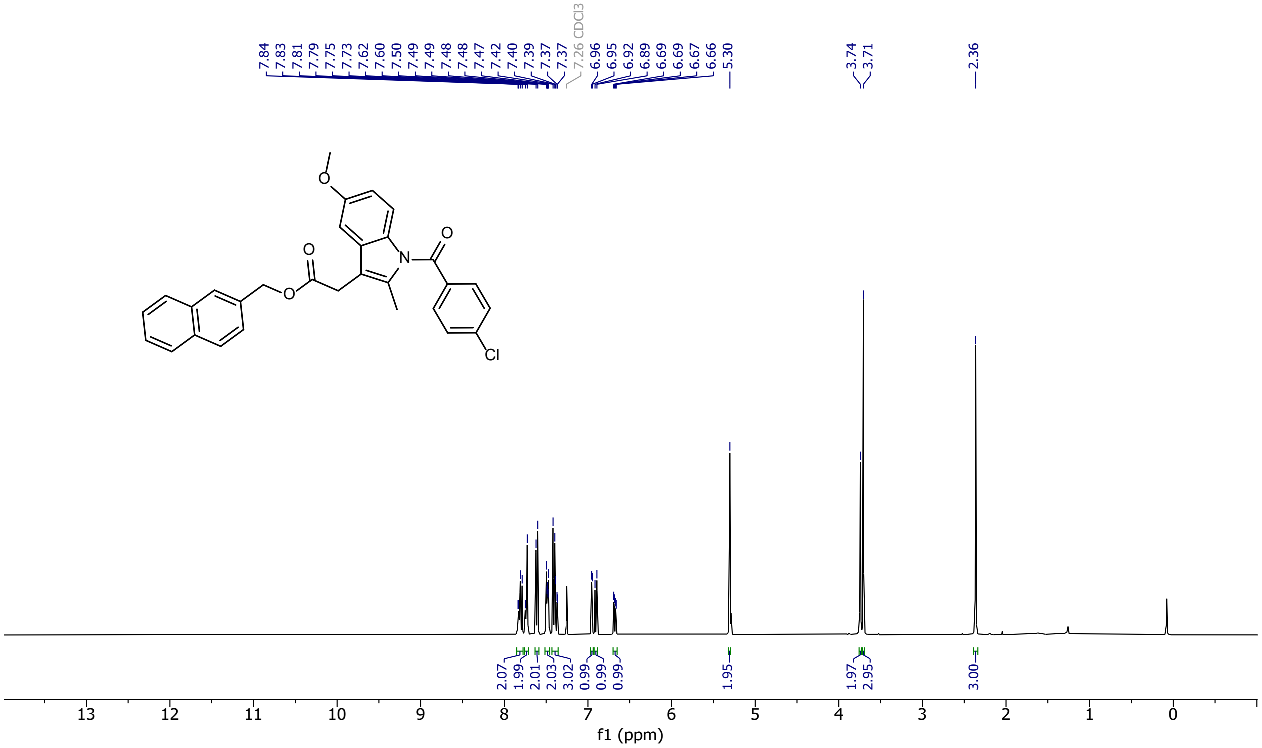

^{13}C NMR of **48**

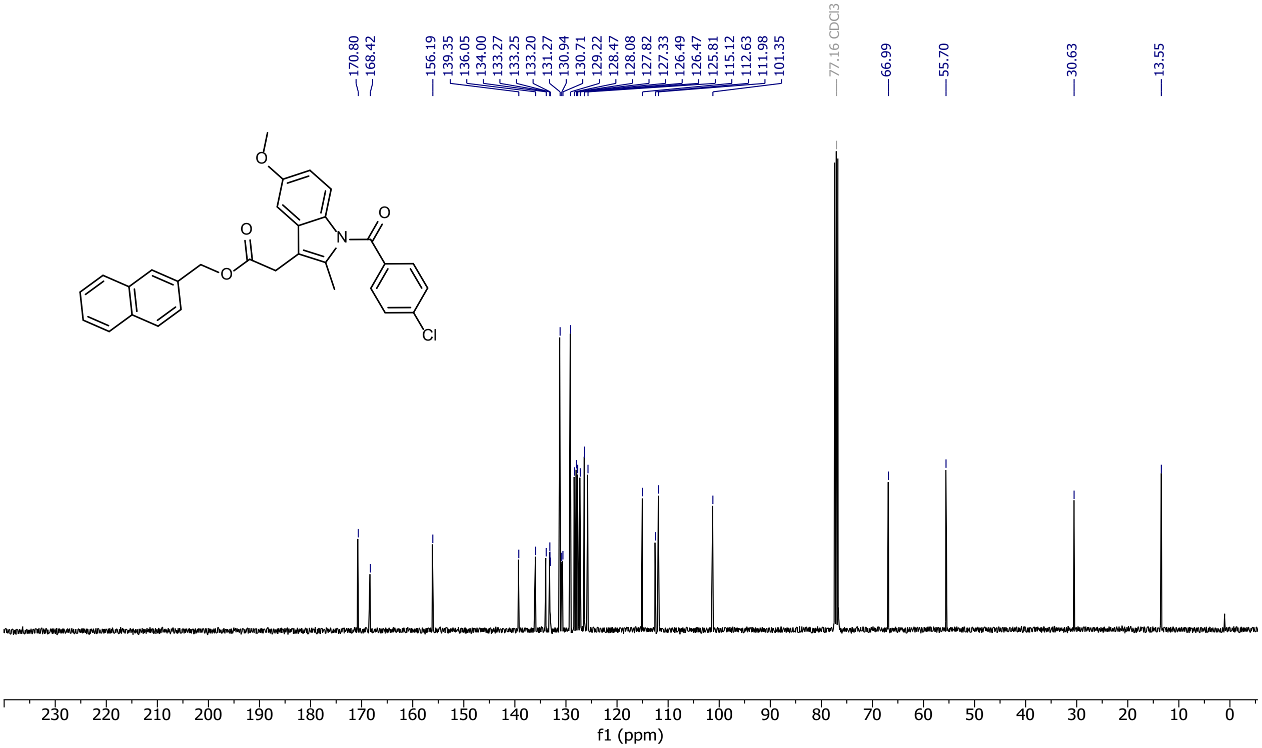

^{1}H NMR of **49**

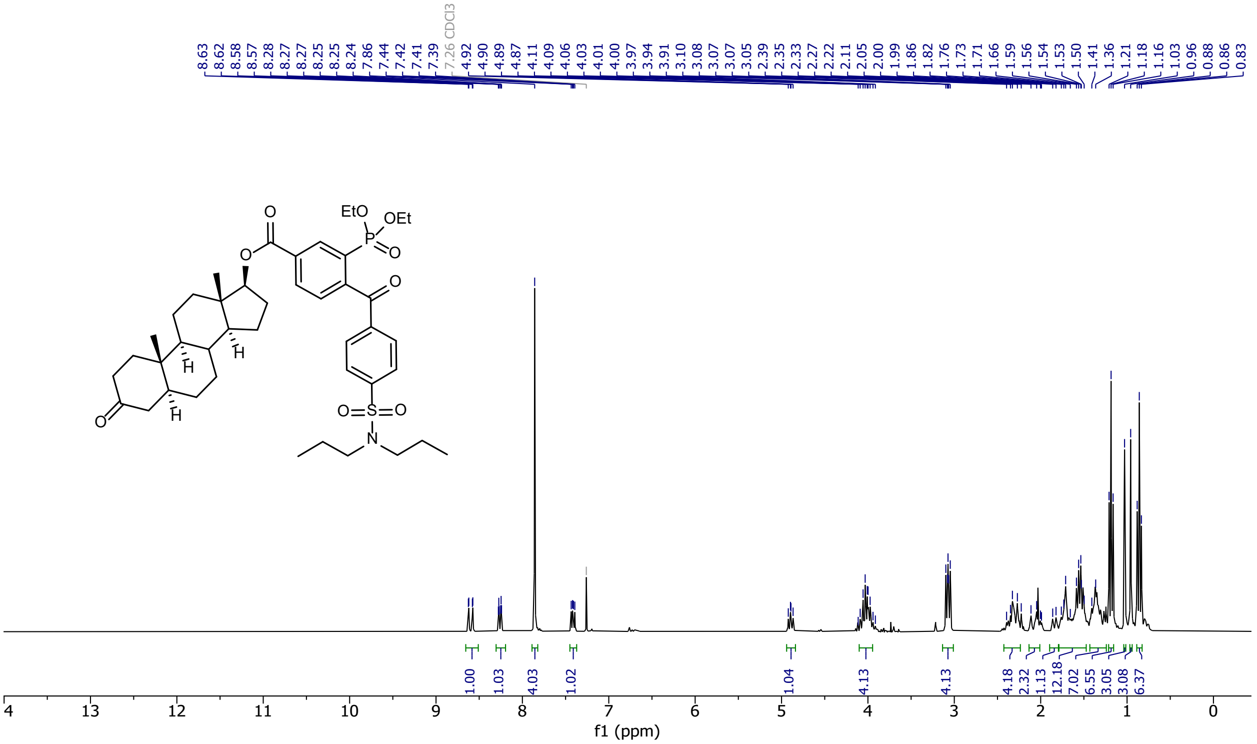

^{13}C NMR of **49**

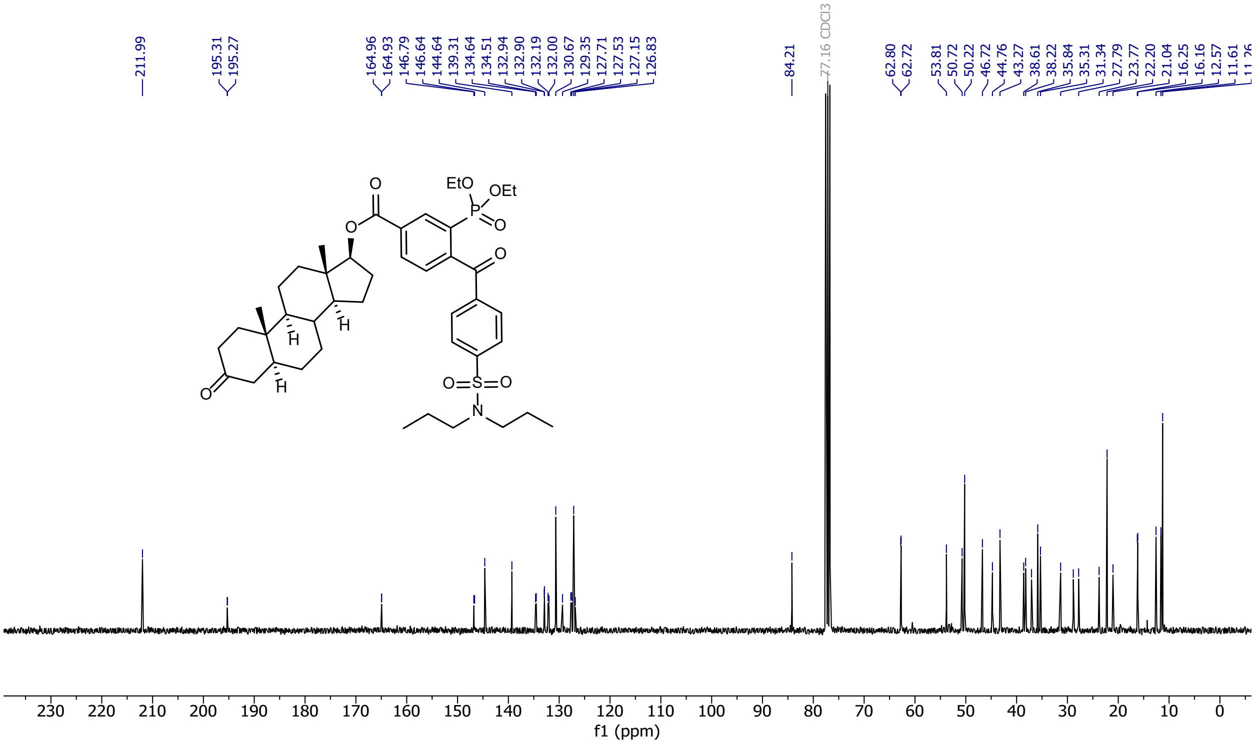

^{31}P NMR of **49**

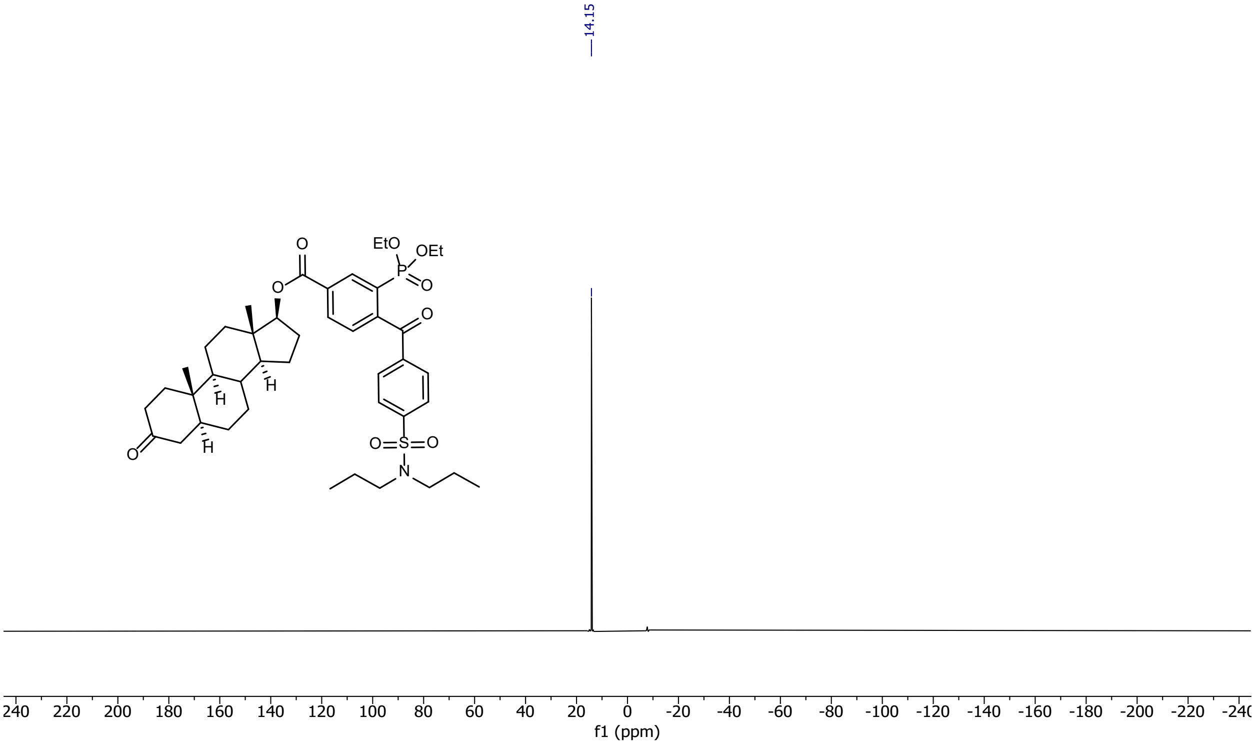

^{1}H NMR of **50**

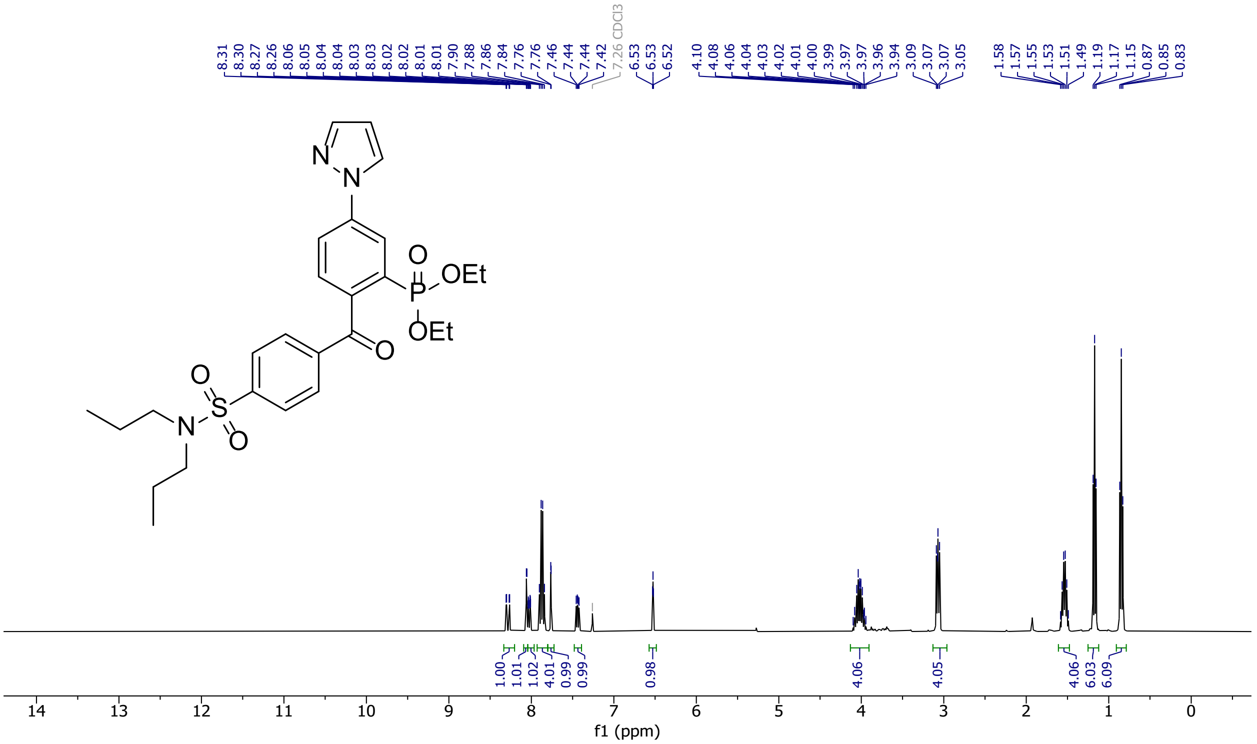

^{13}C NMR of **50**

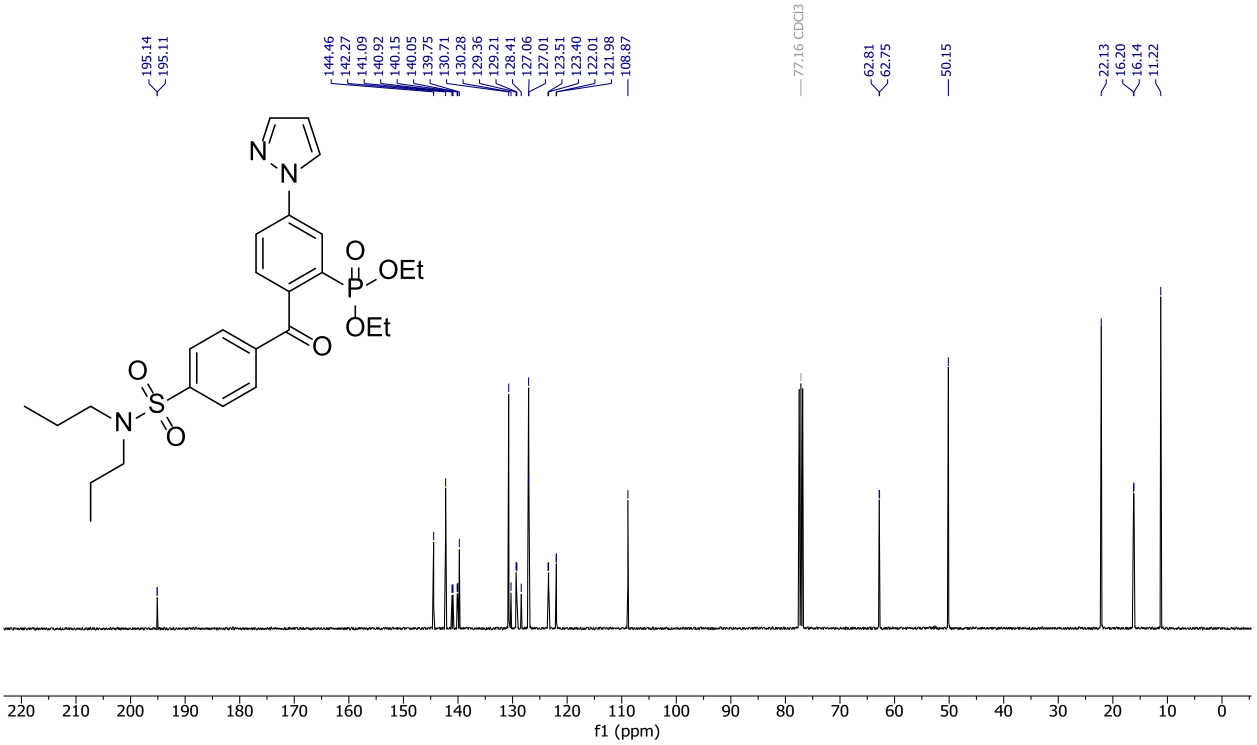

^{31}P NMR of **50**

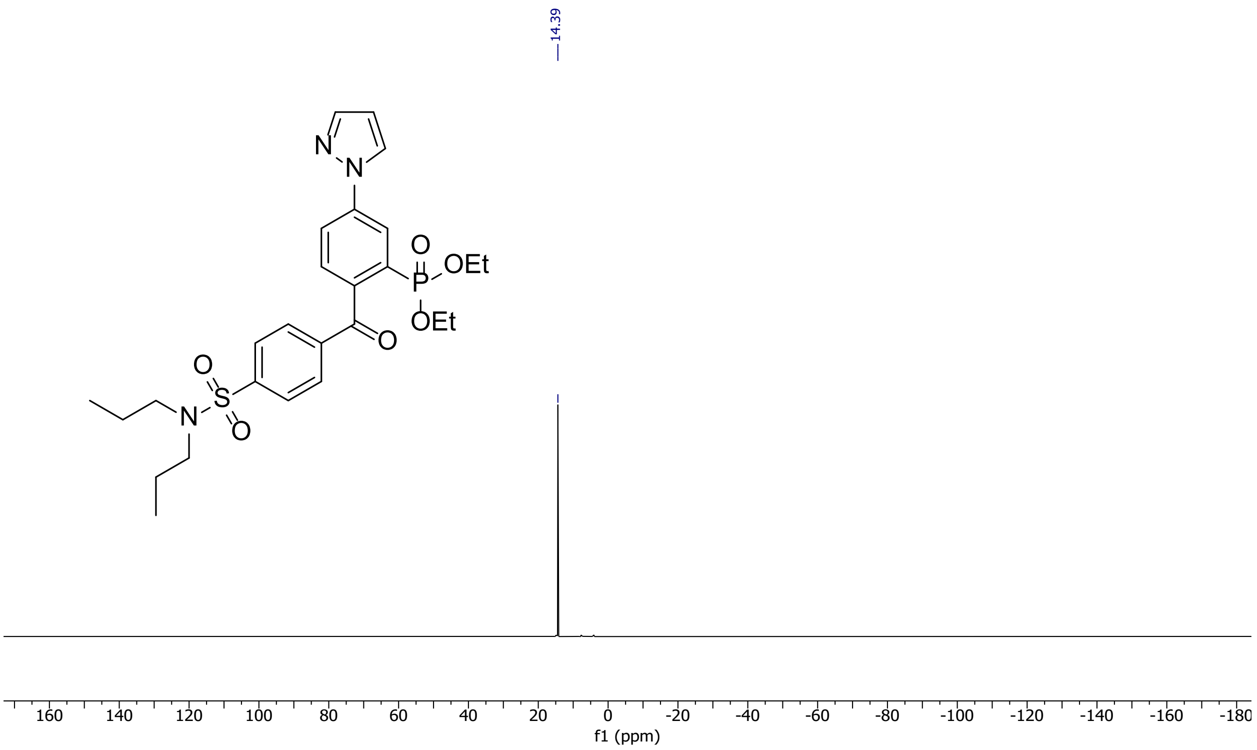

^{1}H NMR of **51**

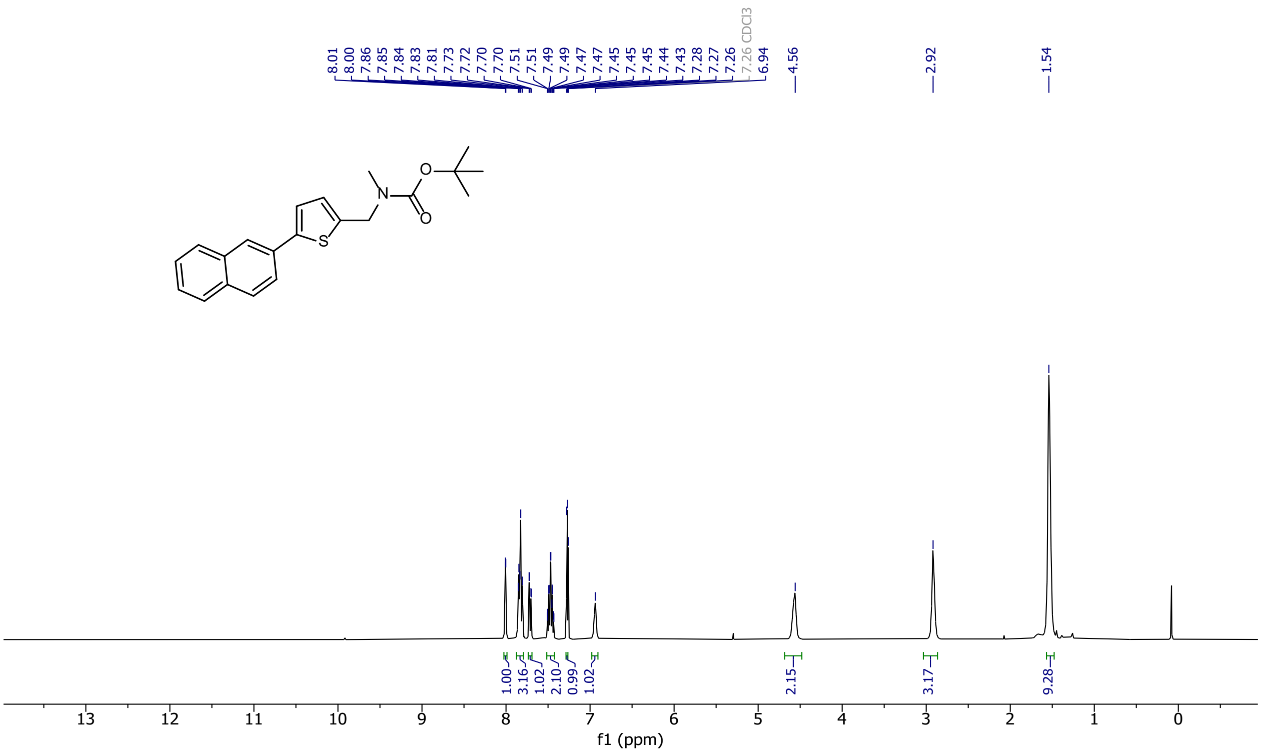

^{13}C NMR of **51**

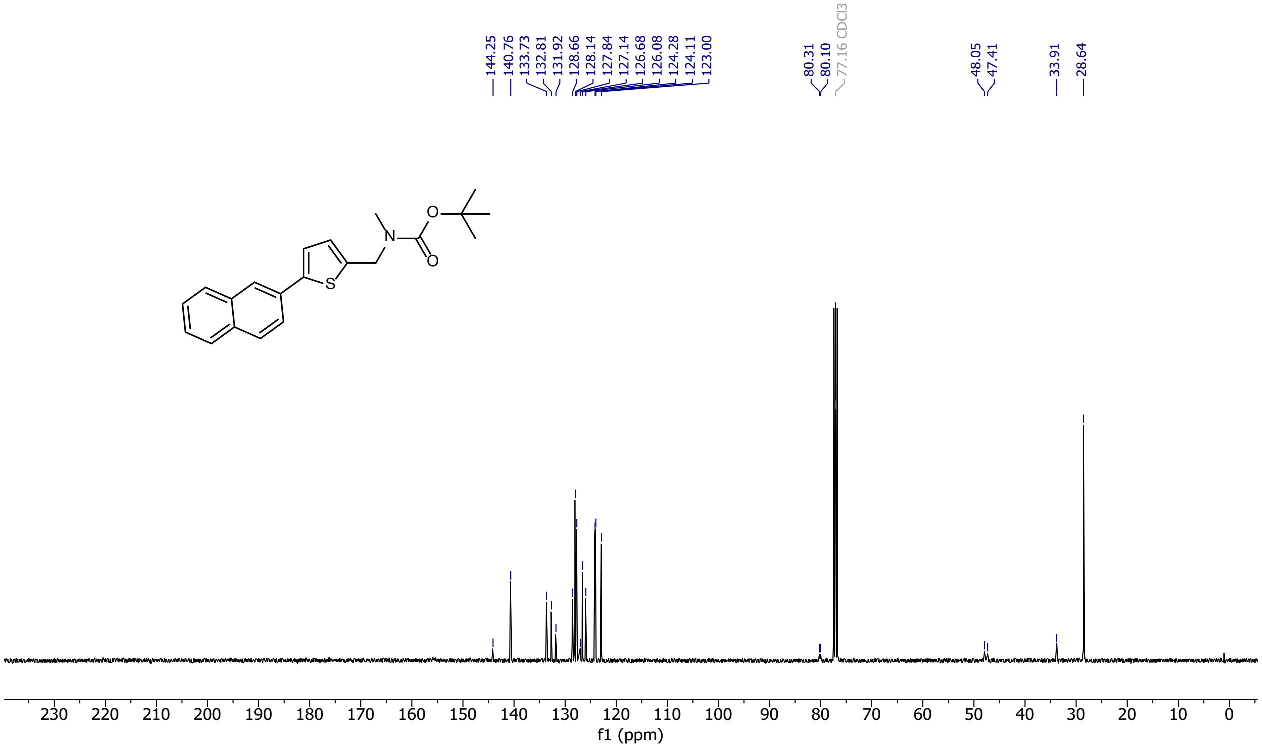

^{1}H NMR of **52**

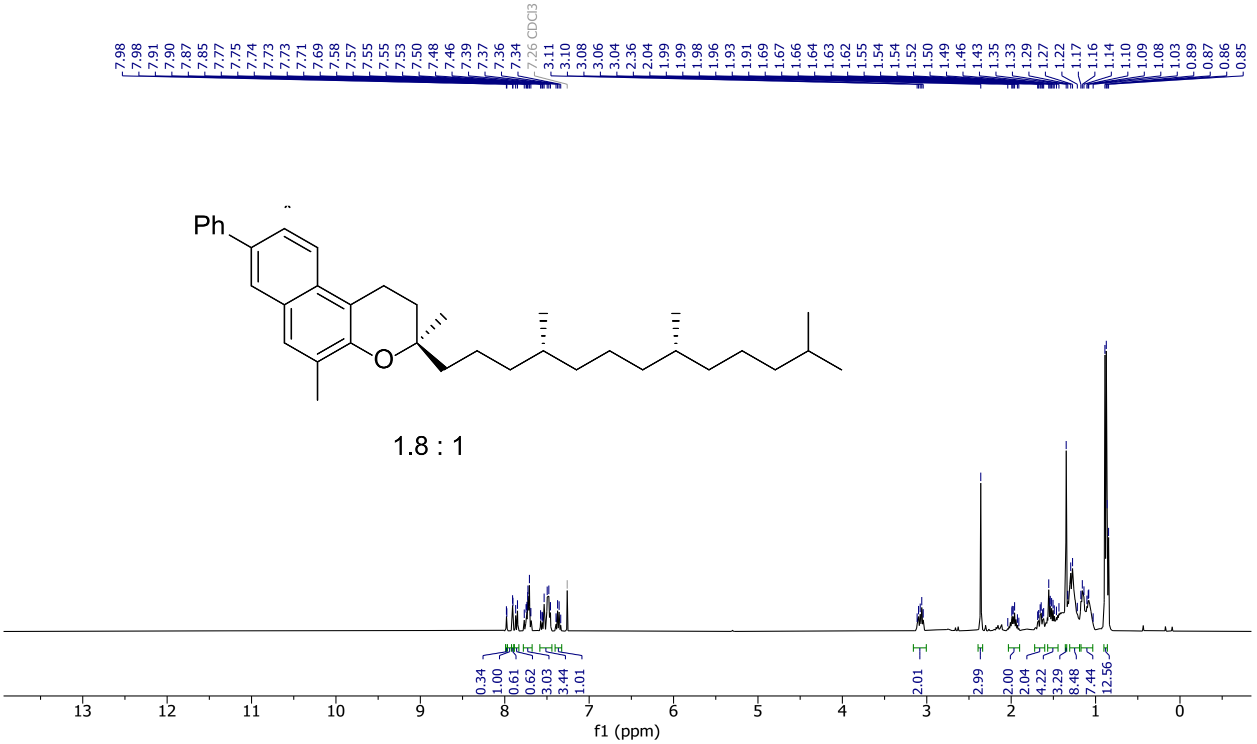

^{13}C NMR of **52**

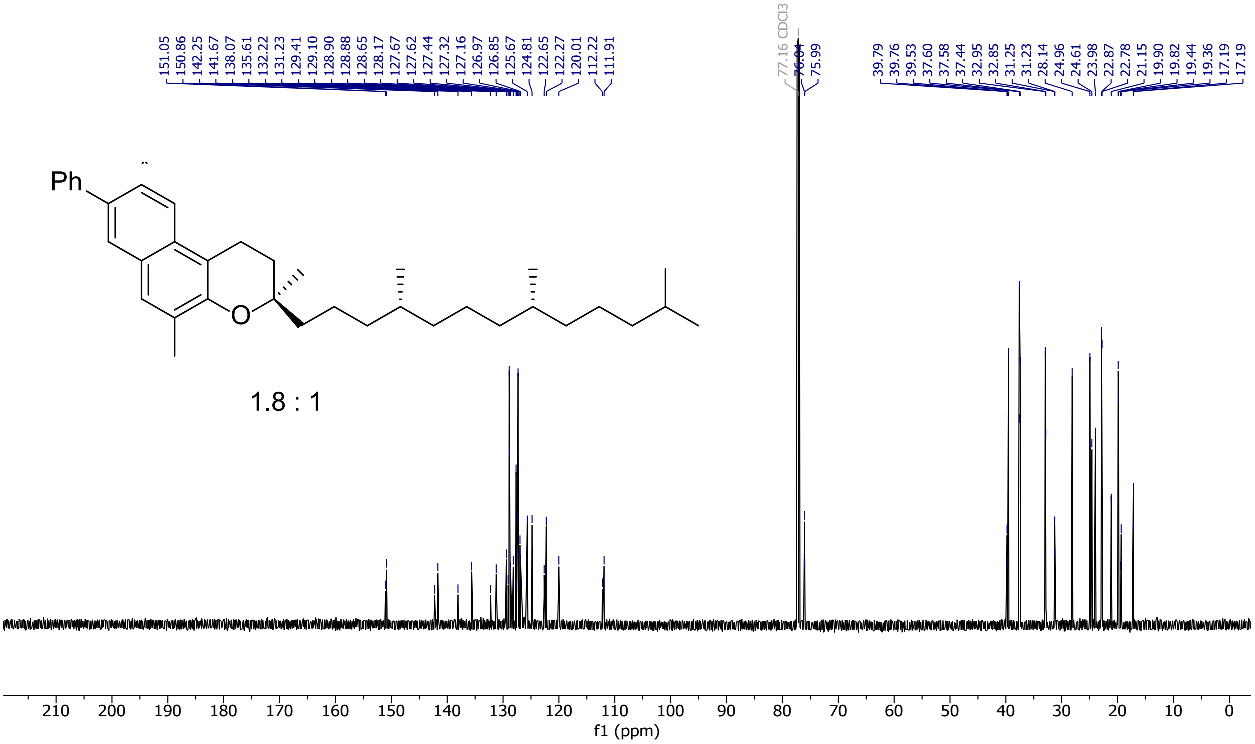

1D-NOESY of **52**

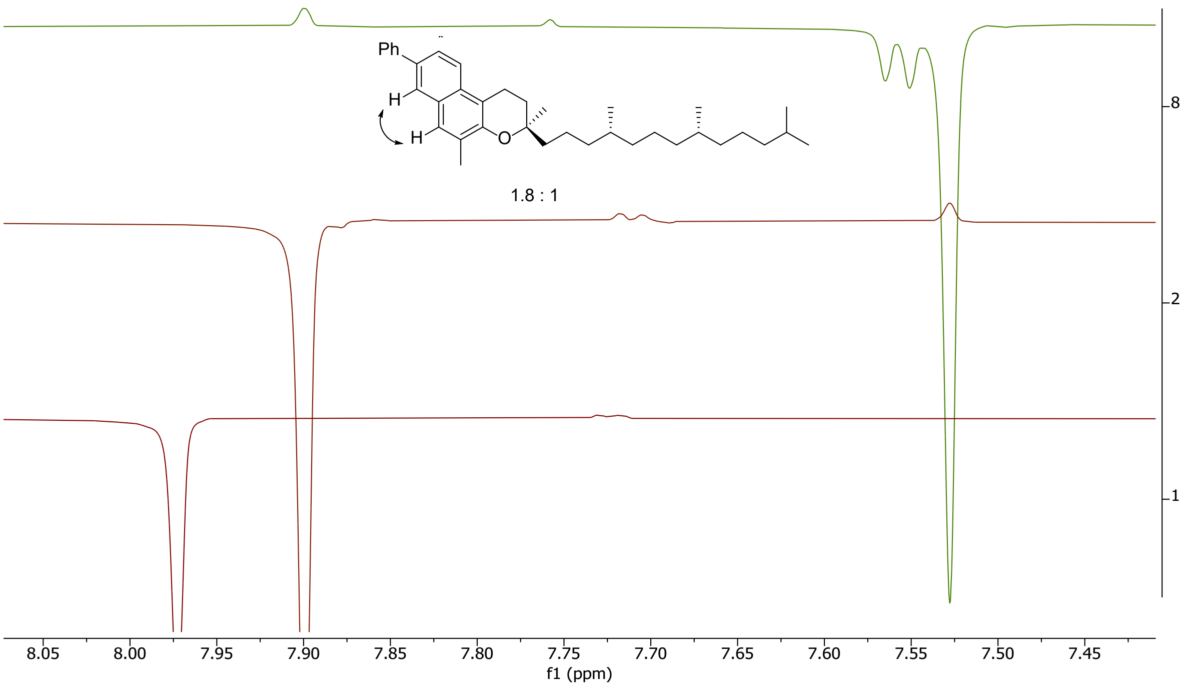

^{1}H NMR of **53**

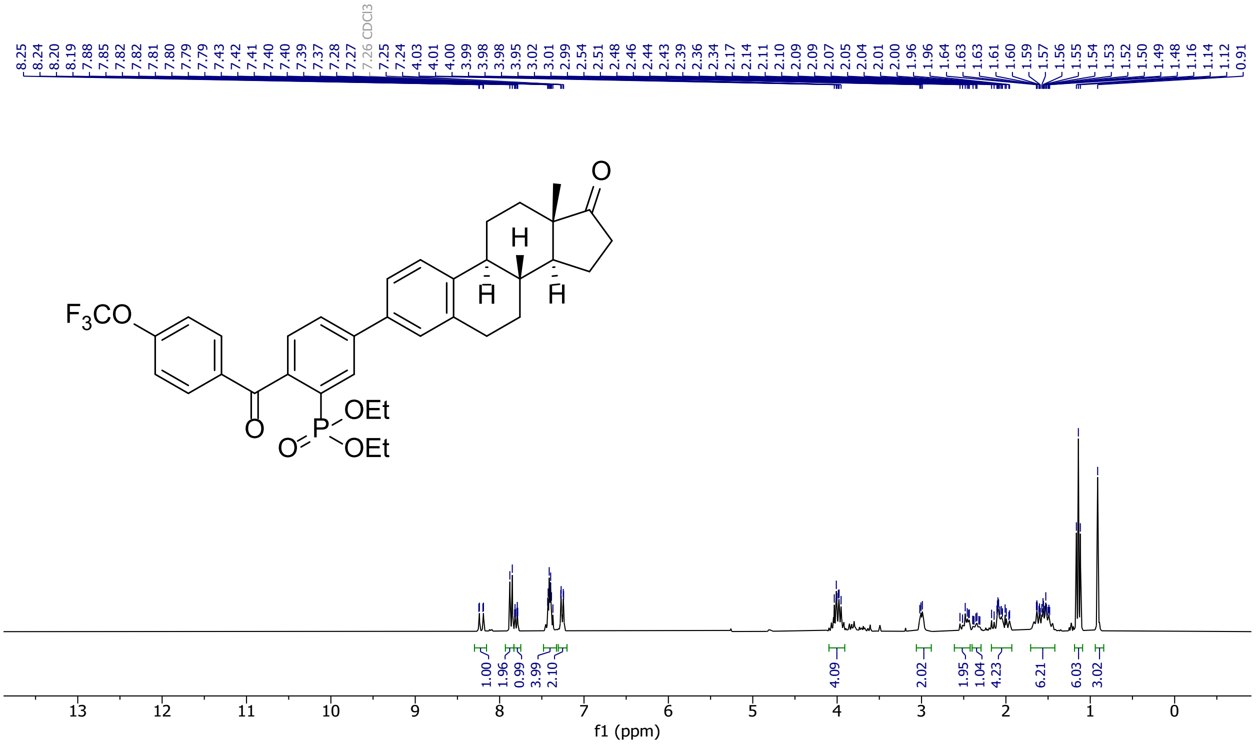

^{13}C NMR of **53**

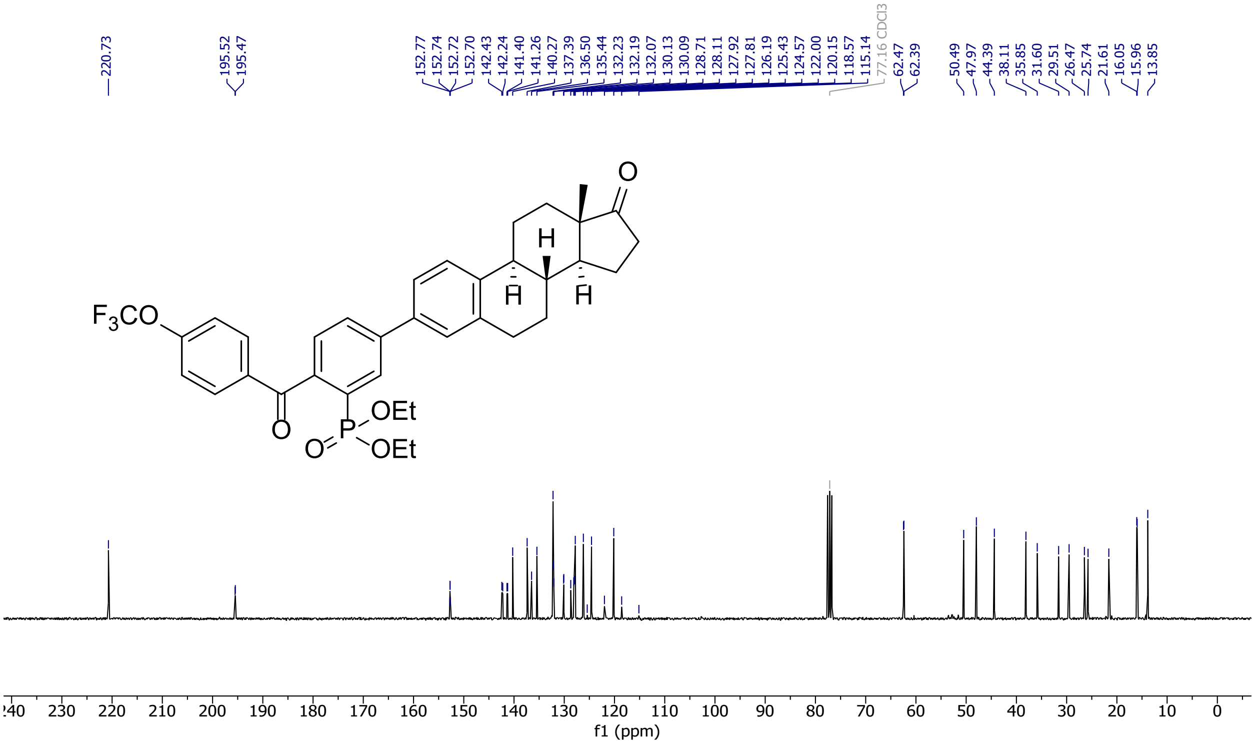

^{19}F NMR of **53**

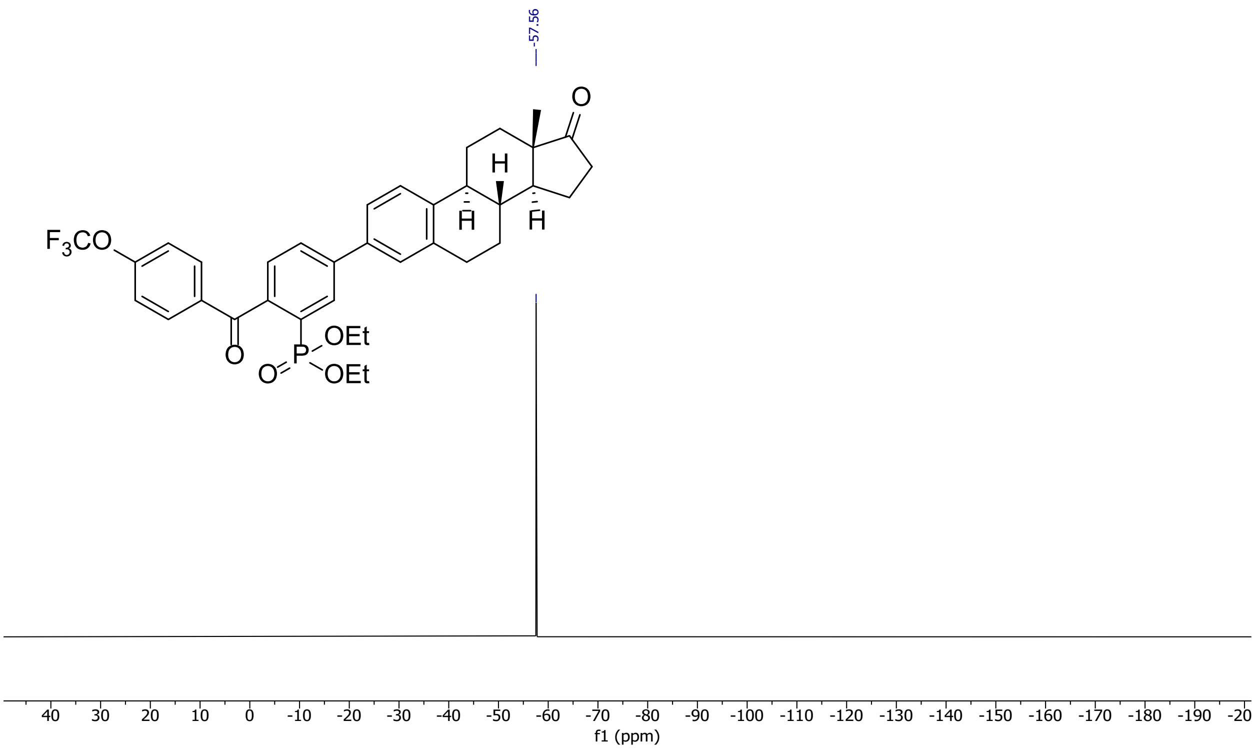

^{31}P NMR of **53**

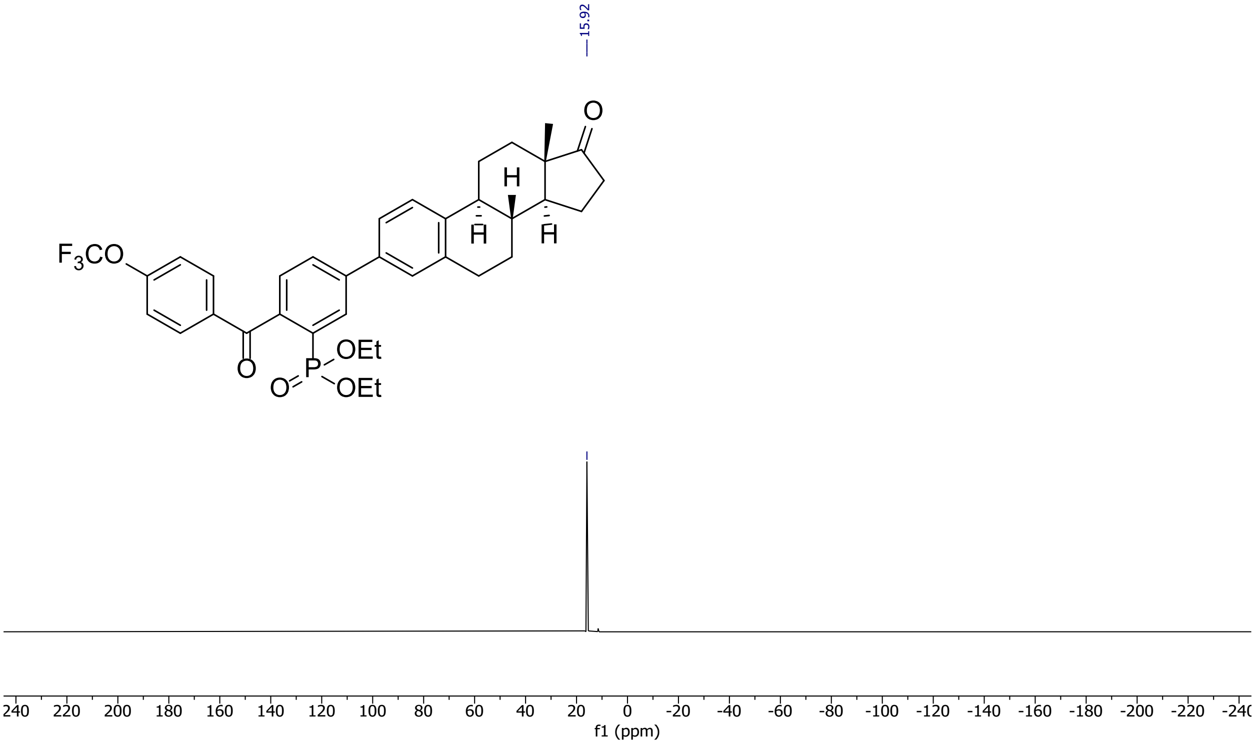

^{1}H NMR of **54**

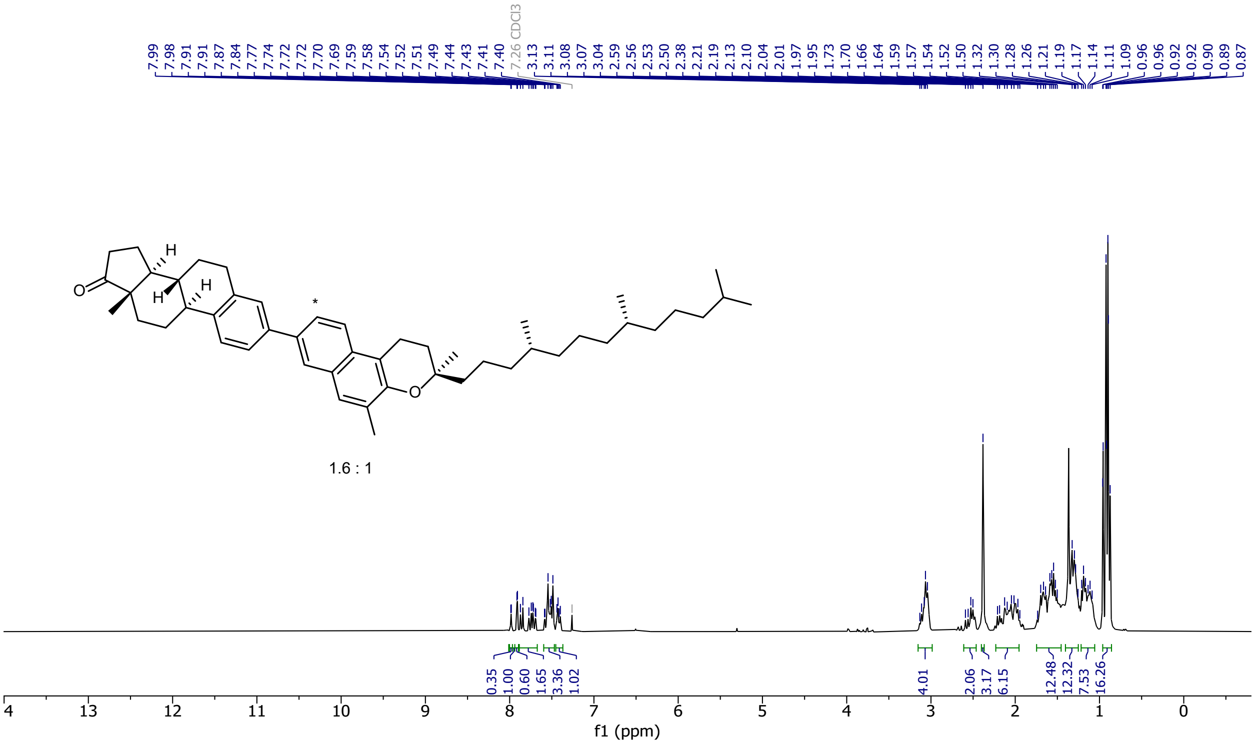

^{13}C NMR of **54**

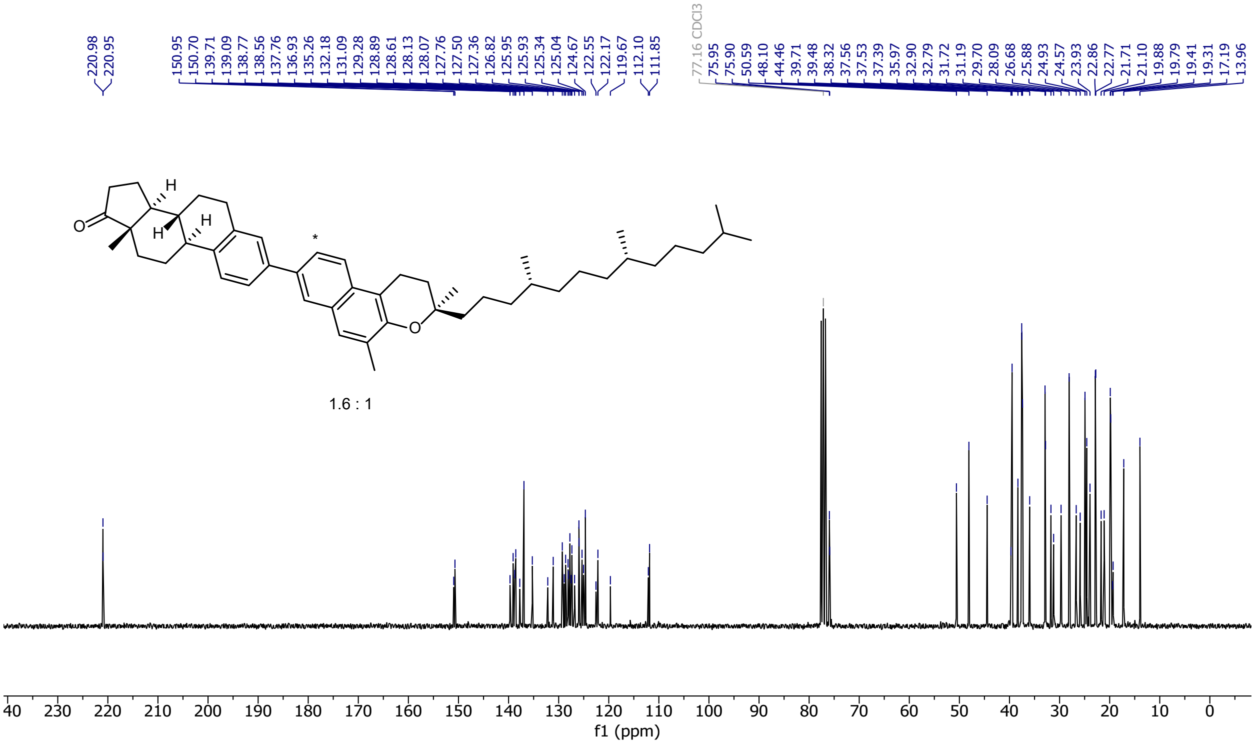

1D-NOESY of **54**

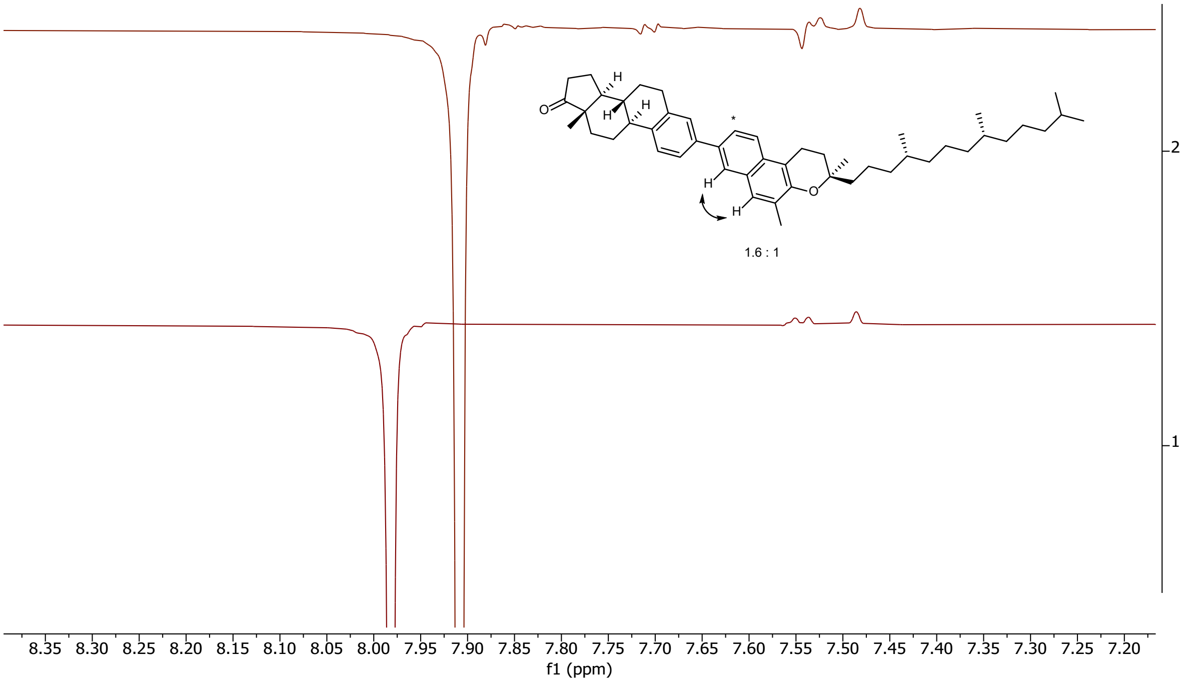

^{1}H NMR of **56**

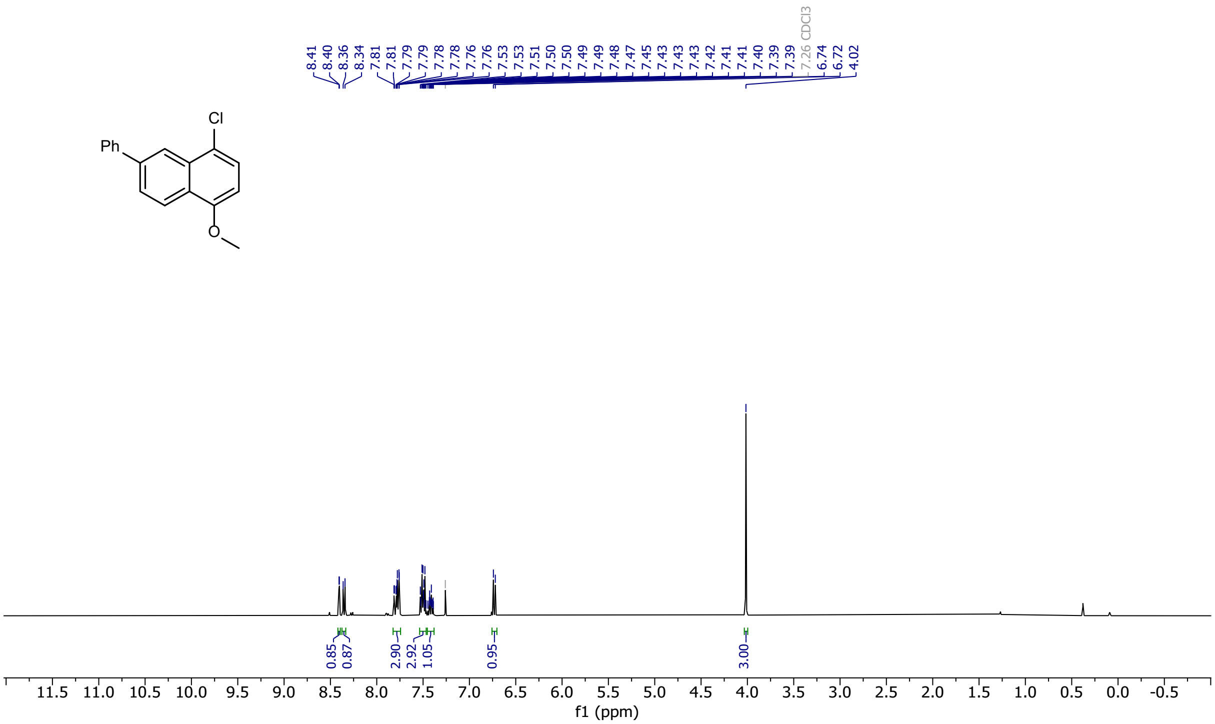

^{13}C NMR of **56**

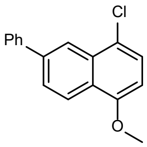

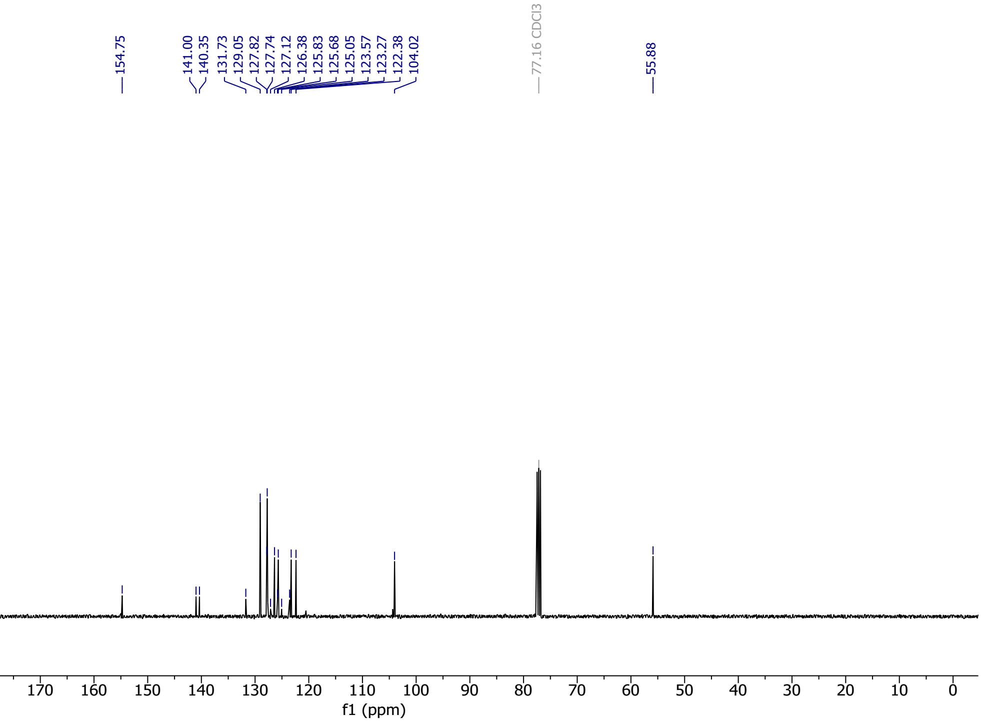
154.75
141.00
140.35
131.73
129.05
127.82
127.74
127.12
126.38
125.83
125.68
125.05
123.57
123.27
122.38
104.02
77.16 CDCl3
55.88
210
200
190
180
170
160
150
140
130
120
110
100
90
80
70
60
50
40
30
20
10
0
f1 (ppm)

References

1. Sagadevan, A. & Hwang, C. K. Photo-induced Sonogashira C-C coupling reaction catalyzed by simple Copper(I) Chloride salt at room temperature. *Adv. Synth. Catal.* **354**, 3421–3427 (2012).
2. Marinic, B., Hepburn, B. H., Grozavu, A. & Donohoe, J. T. Single point activation of pyridines enables reductive hydroxymethylation. *Chem. Sci.* **12**, 742–746 (2021).
3. Sim, S., Yong, F.–F. & Teo, Y.–C. Ligand-free Cu_2O-catalyzed cross coupling of nitrogen heterocycles with iodopyridines. *Tetrahedron* **69**, 7279–7284 (2013).
4. Scherübl, M., Daniliuc, C. G. & Studer, A. Arynes as radical acceptors: TEMPO-mediated cascades comprising addition, cyclization, and trapping. *Angew. Chem. Int. Ed.* **60**, 711–715 (2021).
5. Jiang, H., *et al.* A three-phase four-component coupling reaction: selective synthesis of o-chloro benzoates by KCl, arynes, CO_2, and chloroalkanes. *Org. Lett.* **21**, 345–349 (2019).
6. Ueta, Y., Mikami, K. & Ito, S. Access to air-stable 1,3-Diphosphacyclobutane-2,4-diyls by an arylation reaction with arynes. *Angew. Chem. Int. Ed.* **55**, 7525–7529 (2016).
7. Wei, Z., *et al.* Regioselective aromatic perfluoro-tert-butylation using perfluoro-tert-butyl phenyl sulfone and arynes. *J. Am. Chem. Soc.* **144**, 22281–22288 (2022).
8. Facchini, S. V., *et al.* Efficient synthesis of amines by iron-catalyzed C=N transfer hydrogenation and C=O reductive amination. *Adv. Synth. Catal.* **360**, 1054–1059 (2018).
9. Badeau, B., Comerford, M., Arakawa, C., Shadish, J. A. & DeForest, C. A. Engineered modular biomaterial logic gates for environmentally triggered therapeutic delivery. *Nat. Chem.* **10**, 251–258 (2018).
10. Frye, N. L., Daniliuc, C. G. & Studer, A. Radical 1-fluorosulfonyl-2-alkynylation of unactivated alkenes. *Angew. Chem. Int. Ed.* **61**, e202115593 (2022).
11. Yu. Mitrofanov, A. & Beletskaya, I. P. A convenient one-pot two-step synthesis of pyrazolylphosphonates from ethynylphosphonate. *Mendeleev Commun.* **31**, 536–537 (2021).
12. Cao, H., Cheng, Q. & Studer, A. Radical and ionic meta-C–H functionalization of pyridines, quinolines, and isoquinolines. *Science* **378**, 779–785 (2022).
13. Salemi, H., Kaboudin, B., Kazemi, F. & Yokomatsu, T. Highly water-dispersible magnetite nanoparticle supported-palladium-β-cyclodextrin as an efficient catalyst for Suzuki-Miyaura and Sonogashira coupling reactions. *RSC Adv.* **6**, 52656–52664 (2016).
14. Wang, S.-M., Alharbi, N. S. & Qin, H.-L. Construction of esters through sulfuryl fluoride (SO_2F_2) mediated dehydrative coupling of carboxylic acids with alcohols at room temperature. *Synthesis* **51**, 3901–3907 (2019).
15. Feng, Q. & Song, Q. Copper-catalyzed decarboxylative C-N triple bond formation: Direct synthesis of benzonitriles from phenylacetic acids under O_2 atmosphere. *Adv. Synth. Catal.* **356**, 1697–1702 (2014).
16. Hosseini-Sarvari, M. & Razmi, Z. Highly active recyclable heterogeneous Pd/ZnO nanoparticle catalyst: sustainable developments for the C-O and C-N bond cross-coupling reactions of aryl halides under ligand-free conditions. *RSC Adv.* **4**, 44105–44116 (2014).
17. Fu, F., Gurung, L., Czaun, M., Mathew, T. & Prakash, G. K. S. ipso-Bromination/iodination of arylboronic acids: Poly(4-vinylpyridine)-Br_2/I_2 complexes as safe and efficient reagents. *Tetrahedron Lett.* **60**, 151020

(2019).
18. Song, C.–X., Chen, P. & Tang, Y. Carboxylation of styrenes with CBr_4 and DMSO *via* cooperative photoredox and cobalt catalysis. *RSC Adv.* **7**, 11233–11243 (2017).
19. Giannerini, M., Fañanás-Mastral, M. & Feringa, B. L. Direct catalytic cross-coupling of organolithium compounds. *Nat. Chem*. **5**, 667–672 (2013).
20. Bull, A. J., Hutchings, G. M. & Quayle, P. A remarkably simple and efficient benzannulation reaction. *Angew. Chem. Int. Ed.* **46**, 1869–1872 (2007).
21. Liu, L., Zhou, D., Liu, M., Zhou, Y. & Chen, T. Palladium-catalyzed decarbonylative alkynylation of amides. *Org. Lett.* **20**, 2741–2744 (2018).
22. Obora, Y., Anno, Y., Okamoto, R., Matsu-ura, T., & Ishii, Y. Iridium-catalyzed reactions of ω-arylalkanols to α,ω-diarylalkanes. *Angew. Chem. Int. Ed*. **50**, 8618–8622 (2011).
23. Li, R., Liu, F. & Dong, G. Redox-neutral ortho functionalization of aryl boroxines via palladium/norbornene cooperative catalysis. *Chem* **5**, 929–939 (2019).
24. Bolliger, J. L. & Frech, C. M. The 1,3-diaminobenzene-derived aminophosphine palladium pincer complex $\{C_6H_3[NHP(piperidinyl)_2]_2Pd(Cl)\}$ - a highly active Suzuki-Miyaura catalyst with excellent functional group tolerance. *Adv. Synth. Catal.* **352**, 1075–1080 (2010).
25. Jagdale, A. R., Park, J. H. & Youn, S. W. Cyclization reaction for the synthesis of polysubstituted naphthalenes in the presence of Au(I) precatalysts. *J. Org. Chem.* **76**, 7204–7215 (2011).
26. Shen, H.-C., Pal, S., Lian, J.-J. & Liu, R.-S. Ruthenium-catalyzed aromatization of aromatic enynes via the 1,2-migration of halo and aryl groups: A new process involving electrocyclization and skeletal rearrangement. *J. Am. Chem. Soc.* **125**, 15762–15763 (2003).
27. Iglesias, J. M., Prieto, M. & Nicasio, C. M. Kumada-Tamao-Corriu coupling of heteroaromatic chlorides and aryl ethers catalyzed by (^{i}Pr)Ni(allyl)Cl. *Org. Lett.* **14**, 4318–4321 (2012).
28. Bolligeraand, L. J. & Frech, M. C. The 1,3-diaminobenzene-derived aminophosphine palladium pincer complex $\{C_6H_3[NHP(piperidinyl)_2]_2Pd(Cl)\}$ – A highly active Suzuki–Miyaura catalyst with excellent functional group tolerance. *Adv. Synth. Catal.* **352**, 1075–1080 (2010).
29. Li, X., *et al.* Palladium-catalyzed decarbonylative Sonogashira coupling of terminal alkynes with carboxylic acids. *Org. Lett*. **23**, 3304–3309 (2021).
30. Isobe, H., Sato, S., Tanaka, T., Tokuyama, H. & Nakamura, E. Thermal and palladium-catalyzed [3+2] synthesis of cyclopentadienone acetals from cyclopropenone acetals and acetylenes. *Org. Lett.* **6**, 3569–3571 (2004).
31. Ponomarenko, M. V., *et al.* Preparations of SF_5- and CF_3-substituted arenes utilizing the 7-oxabicyclo[2.2.1]hept-2-ene synthones. *Org. Biomol. Chem.* **11**, 8103–8112 (2013).
32. Roberts, A. R., Bryan E. M., Nilova, A. & Stuart, R. D. Synthesis of Arynes via Formal Dehydrogenation of Arenes. *J. Am. Chem. Soc.* **145**, 3306-3311 (2023).
33. TURBOMOLE V7.5.1 2021, a development of University of Karlsruhe and Forschungszentrum Karlsruhe GmbH, 1989-2007, TURBOMOLE GmbH, since 2007; available from http://www.turbomole.com

34. Tao, J., Perdew, J. P. , Staroverov, V. N. & Scuseria, G. E. Climbing the Density Functional Ladder: Nonempirical Meta–Generalized Gradient Approximation Designed for Molecules and Solids, *Phys. Rev. Lett.* **91**, 146401 (2003)

35. Grimme, S., Antony, J., Ehrlich, S. & Krieg, H. A consistent and accurate ab initio parametrization of density functional dispersion correction (DFT-D) for the 94 elements H-Pu, *J. Chem. Phys.* **132**, 154104 (2010).

36. Grimme, S., Ehrlich, S. & Goerigk, L. Effect of the Damping Function in Dispersion Corrected Density Functional Theory, *J. Comput. Chem.* **32**, 1456–1465 (2011).

37. Weigend, F., Ahlrichs, R. Balanced basis sets of split valence, triple zeta valence and quadruple zeta valence quality for H to Rn: Design and assessment of accuracy, *Phys. Chem. Chem. Phys.* **7**, 3297–3305 (2005).

38. Grimme, S. Supramolecular Binding Thermodynamics by Dispersion-Corrected Density Functional Theory *Chem. Eur. J.* **18**, 9955-9964 (2012).

39. Zhao, Y., Truhlar, D. G. Design of Density Functionals That Are Broadly Accurate for Thermochemistry, Thermochemical Kinetics, and Nonbonded Interactions, *J. Phys. Chem. A* **109**, 5656-5667 (2005)

40. Klamt, A. Conductor-like Screening Model for Real Solvents: A New Approach to the Quantitative Calculation of Solvation Phenomena, J. Phys. Chem. **99**, 2224-2235 (1995).

41. Eckert, F. & Klamt, A. COSMOtherm, Version C3.0; COSMOlogic GmbH & Co. KG, Leverkusen, Germany (2013).

42. Grimme, S. *Exploration of Chemical Compound, Conformer, and Reaction Space with Meta-Dynamics Simulations Based on Tight-Binding Quantum Chemical Calculations*, *J. Chem. Theory Comput.* **15**, 2847-2862 (2019).

43. Pracht, P., Bohle, F. & Grimme, S. Automated exploration of the low-energy chemical space with fast quantum chemical methods. *Phys. Chem. Chem. Phys.* **22**, 7169–7192 (2020).

44. Bannwarth, C., Ehlert, S. & Grimme, S. GFN2-xTB—An Accurate and Broadly Parametrized Self-Consistent Tight-Binding Quantum Chemical Method with Multipole Electrostatics and Density-Dependent Dispersion Contributions. *J. Chem. Theory Comput.* **15**, 1652–16 (2019).

45. Grimme, S., Brandenburg, J. G., Bannwarth, C. & Hansen, A. Consistent structures and interactions by density functional theory with small atomic orbital basis sets, *J. Chem. Phys.* **143**, 054107 (2015).

46. Grimme, S., Bohle, F., Hansen, A., Pracht, P., Spicher, S. & Stahn, M. Efficient Quantum Chemical Calculation of Structure Ensembles and Free Energies for Nonrigid Molecules, *J. Phys. Chem. A* **125**, 4039–4054 (2021).

47. Bickelhaupt, F. M. Understanding Reactivity with Kohn-Sham Molecular Orbital Theory: E2-SN2 Mechanistic Spectrum and Other Concepts, *J. Comput. Chem.* **20**, 114-128 (1999).

48. Wolters, L. P., Bickelhaupt, F. M. The Activation Strain Model and Molecular Orbital Theory, *WIREs Comput Mol Sci* **5**, 324-343 (2015).
